# Methodological Approach Based on Structural Parameters, Vibrational Frequencies, and MMFF94 Bond Charge Increments for Platinum-Based Compounds

**DOI:** 10.1021/acsomega.4c10141

**Published:** 2025-02-20

**Authors:** Gloria Castañeda-Valencia, Lucas F. Gama, Murugesan Panneerselvam, Viviane S. Vaiss, Isabella A. Guedes, Laurent E. Dardenne, Luciano T. Costa

**Affiliations:** † MolMod-CS, Institute of Chemistry, Fluminense Federal University, Campus Valonguinho, Centro, Niterói, Rio de Janeiro CEP 24020-141, Brazil; ‡ Laboratório Nacional de Computação Científica, Avenida Getúlio Vargas, 333, Quitandinha, Petrópolis, Rio de Janeiro CEP 25651-075, Brazil

## Abstract

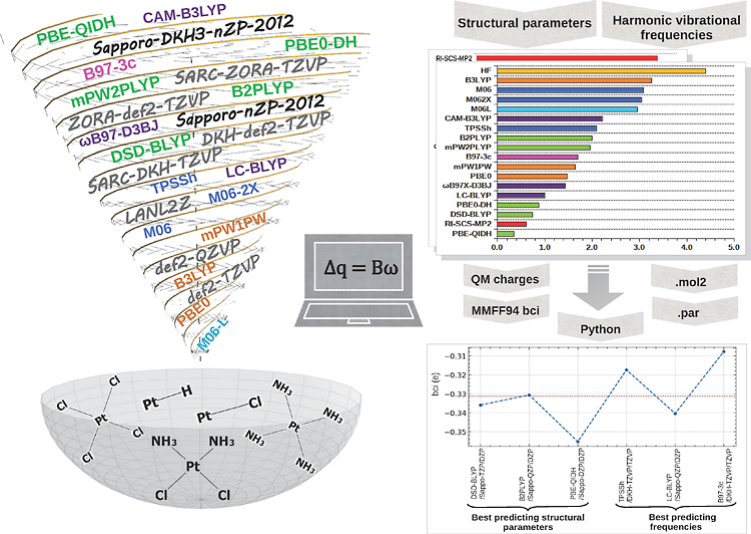

In this work, we performed a comprehensive benchmark study for the ground state of five small- and medium-sized platinum derivatives, PtH, PtCl, [PtCl_4_]^2–^, [Pt(NH_3_)_4_]^2+^, and *cis*-[Pt(NH_3_)_2_Cl_2_], in the gas phase and two cisplatin polymorphs in the solid phase. The benchmark encompassed 16 density functionals, including nonhybrids, hybrids, and double hybrids. Furthermore, Hartree–Fock (HF) and Post-HF by Møller–Plesset MP2 methods were also tested. Additionally, 11 basis sets were explored, comparing relativistic all-electron and RECP approaches. Our results indicate that the methodologies best suited for predicting structural parameters do not excel in predicting vibrational frequencies and vice versa. In the context of this theoretical framework, we also examine the derivation of partial atomic charges and bond charge increments (bci) as fundamental parameters within the MMFF94 classical force field. Our results show that the partial atomic charges of CHELPG present a slight charge fluctuation in Pt for all investigated levels of theory, and this behavior reproduces well the soft acid definition for Pt^2+^, giving the best description of the chemical environment of platinum in the cisplatin complex. The average calculated bci values effectively capture the atomic charge variations in the chemical environment of Pt in the investigated species. The developed bci optimization tool, based on MMFF94, was implemented using a Python code made available at https://github.com/molmodcs/bci_solver. This methodology will be further implemented in the DockThor receptor–ligand docking program, allowing future molecular docking validations involving ligand compounds containing Pt atoms.

## Introduction

Cisplatin, *cis*-[Pt(NH_3_)_2_Cl_2_], and its derivatives have been the target of many theoretical calculations over the years^1−3^ due to their anticancer activities.^4−6^ To study cisplatin-based compounds^7,8^ using classical methods such as molecular docking and molecular dynamics techniques, we need to develop accurate force field parameters, which is a challenge for transition metals such as platinum.^9,10^ Molecular Mechanics (MM) force field parameters are commonly developed for amino acids and nucleic acids such as CHARMM^11^ and GAFF,^12−14^ as well as organic compounds and small druglike molecules.^15−19^ The Universal Force Field is an example of a development applied to organometallic compounds, covering a broad range of transition metals.^20^ In this context, force field parametrization of metals is not an automatic procedure.^21,22^ Efforts in force field development for metals have frequently focused on common metallic ions found in complexes applied to biological systems.^21^

Merck Molecular Force Field (MMFF94) and its variation MMFF94s (for delocalized trigonal nitrogen centers with planar geometries) were developed originally by Halgren^17,23,24^ for MM-based methods.^25−27^ It is a robust and reliable force field that was thought to have a potential function describing the polarizability and the chemical environment of an atom in a molecule. MMFF94 has been implemented in many molecular tool kits (e.g., Babel^28^ and RDkit^29^), MM programs as Avogadro,^30^ and in receptor–ligand docking,^31−33^ and it has been also used as the main force field to obtain physical descriptors for binding affinity scoring functions.^33^ Thanks to its extensive list of parametrized atom types, MMFF94 is well suited for docking of drug-like compounds, as it eliminates the need for quantum mechanical calculations to derive partial atomic charges for small organic molecules. This is a great advantage for large-scale virtual screening of small-molecule libraries containing thousands, millions, or even billions of compounds. Moreover, the MMFF94 strategy for deriving partial atomic charges using bond-charge-increment (bci) parameters considers the chemical polarization context due to covalent chemical bonds. The use of MMFF94 for large-scale virtual screening is currently freely available^34^ through the DockThor VS Web server.^35^

However, a few metallic ions have parameters available for MMFF94, missing platinum (Pt, Z = 78), for instance. The parametrization of the empirical force field for platinum, in general, is based on optimized geometry and force constants^10^ of their complexes, where the charges may be derived from Restrained Electrostatic Potential Atomic Partial charges (RESP)^9^ and also from Electrostatic Potentials using a Grid-based charge method (CHELPG).^36,37^ The great challenge in the development of force fields is the transferability of the parameters that, in general, are derived for a specific task and chemical environment, as demonstrated by Jorgensen and coauthors^38^ in their recent work. For platinum-based complexes, such specific ways have been developed using Density Functional Theory (DFT) calculations.^10,39,40^ With this, and especially for transition metals, the selection of the level of theory implies enhancing the accuracy followed by higher computational cost related to the size of the chemical system.^41^

Cisplatin is the prototype molecule for platinum-based complex studies,^6,42^ while their derivatives are greater in size besides containing other elements such as nitrogen, chlorine, and hydrogen. In this work, in addition to cisplatin [Pt(NH_3_)_2_Cl_2_], we performed a comprehensive study for the ground state of four small and medium platinum derivatives, PtH, PtCl, [PtCl_4_]^2–^, and  in the gas phase and also for two polymorphs of cisplatin in the solid phase (see Figures 2 and 5).

**Figure 1:**
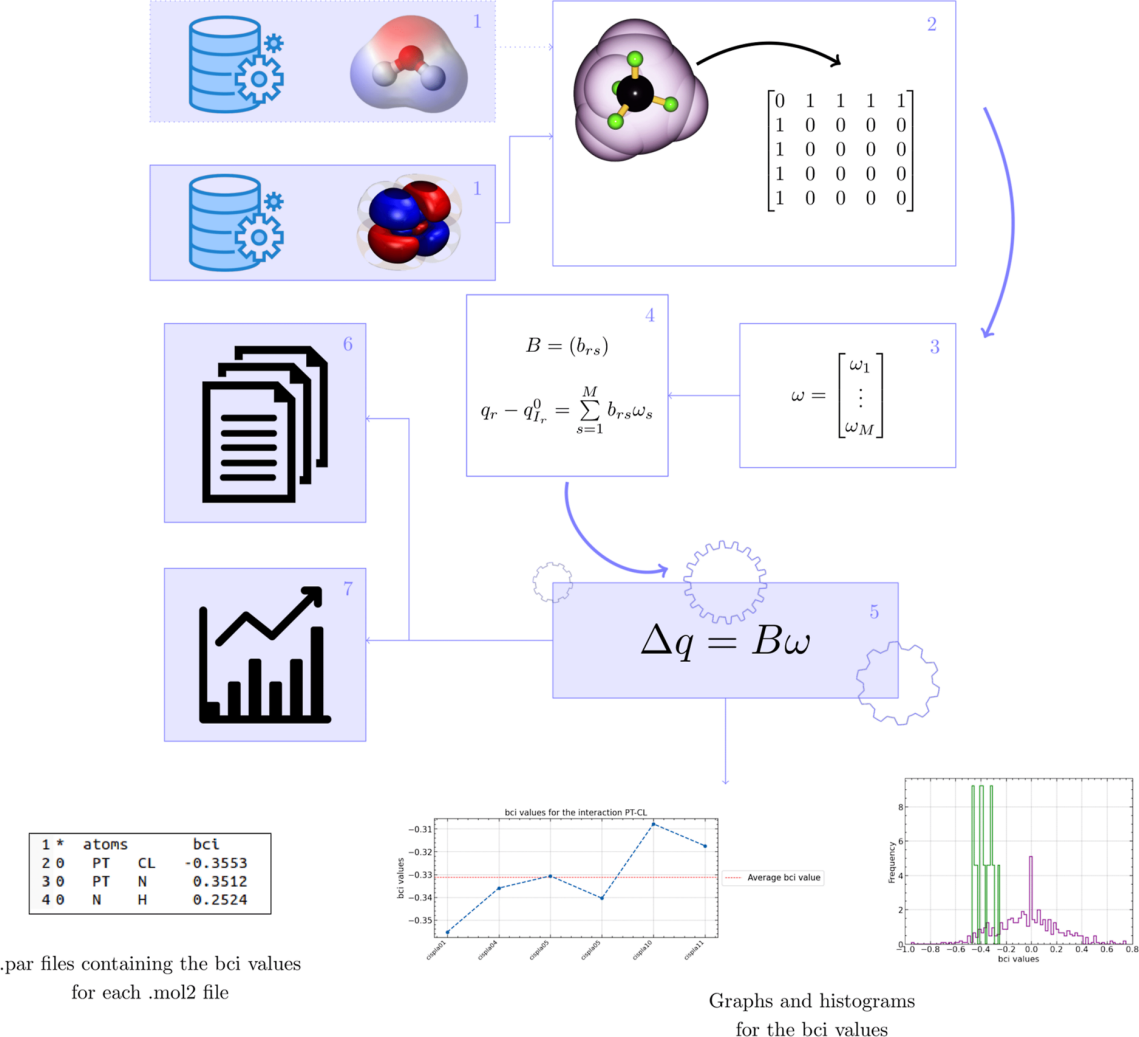
Flowchart for bci calculations: (1) The program accepts input from the user in the form of either a single.mol2 file or a folder containing.mol2 files, which store the connectivity information about the chemical structures. Optionally, the user can also provide an external file or folder containing charges, which will be utilized for computing the associated bci values. Initially, if charges are specified by an external source, the program will generate new.mol2 files with charges specified according to the provided source. (2) The program converts each.mol2 file to its associated adjacency matrix . (3) Array manipulations conducted by NumPy are performed to identify all possible bci pair interactions present in the structure. (4) Additional array manipulations are performed to construct matrix *B*, which contains the coefficients of each of the unknown bci values. (5) The linear equation, eq 4, is subsequently solved using the method of least-squares, employing the SciPy function scipy.linalg.lstsq. (6) Output files containing statistical data regarding the bci values for each distinct chemical bond are generated. (7) Finally, .par files are generated as output, containing the bci values for each.mol2 file through the calculations. Additionally, graphs and histograms depicting the variation between the bci values calculated across different structures or methods are also created.

**Figure 2:**
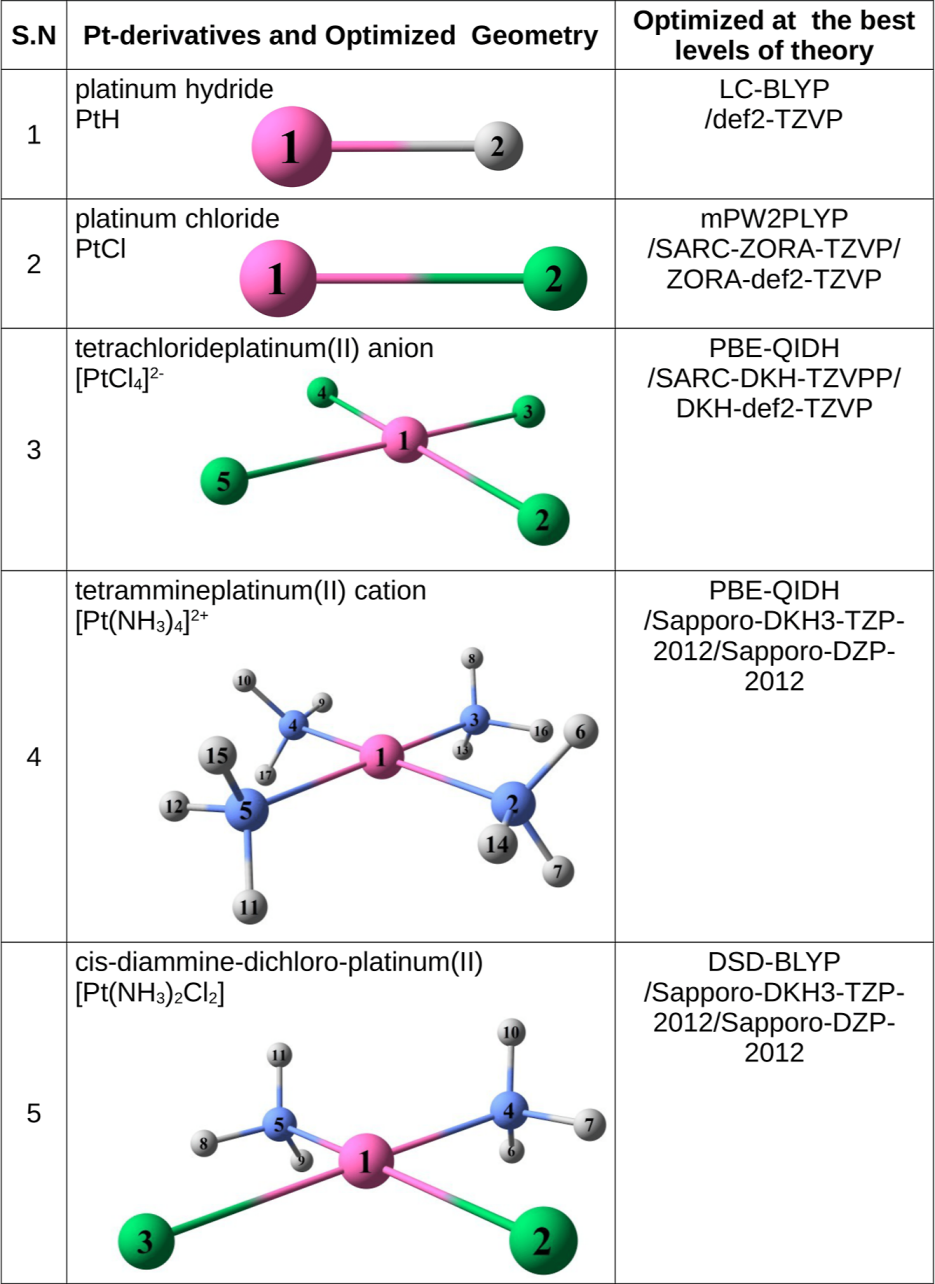
Optimized geometries of the selected Pt derivatives at the choice of the best functionals in the gas phase. The pink, blue, green, and gray balls correspond to Pt, N, Cl, and H atoms, respectively.

**Figure 3:**
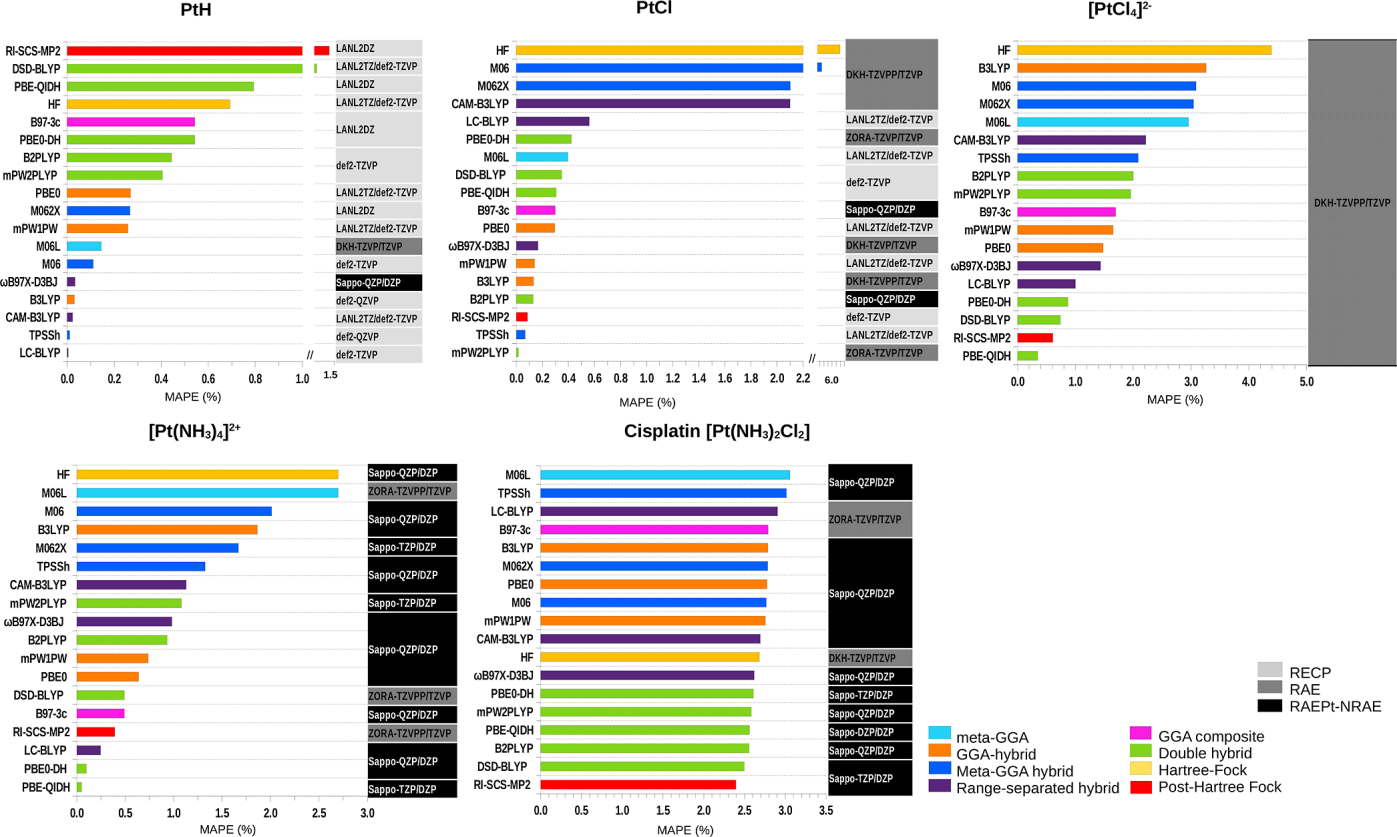
MAPE (%) calculation for Pt-derivatives PtH, PtCl, , , and cisplatin [Pt(NH_3_)_2_Cl_2_] with ground-state optimization in the gas phase. This is a selection from each method, along with its most optimal representative basis set, resulting from calculations at all levels of theory for predicting structural parameters, when compared with experimental refs (127–130 and 53), respectively. In this figure, we used short names for basis sets, full names are in Table 1.

**Figure 4:**
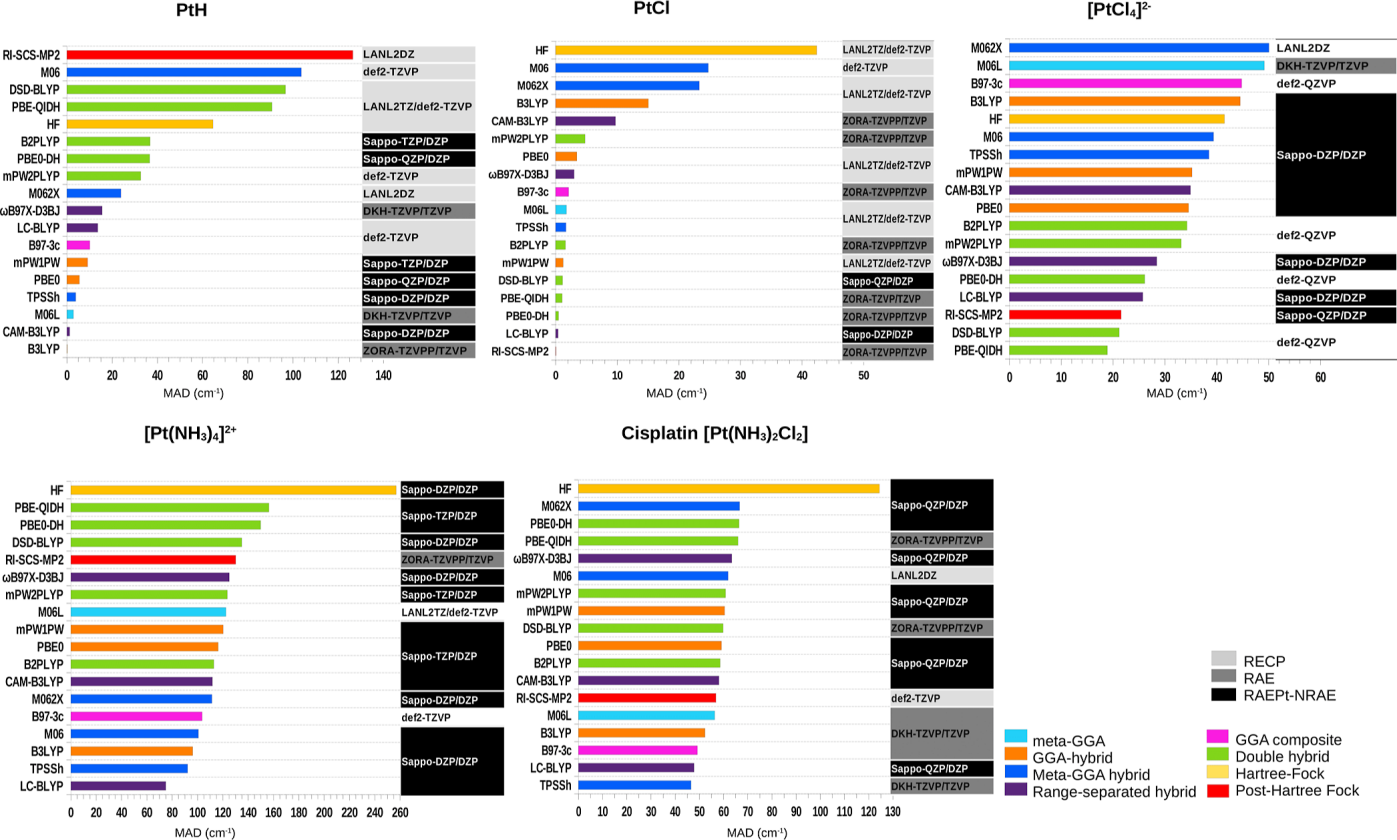
MAD calculation for Pt-derivatives PtH, PtCl, , , and [Pt(NH_3_)_2_Cl_2_] relative to the IR spectrum with ground-state optimization in the gas phase. This is a selection from each method, along with its most optimal representative basis set, resulting from calculations at all levels of theory for predicting harmonic vibrational frequencies, when compared with experimental refs (127, 136, 137, 130, and 39), respectively. In this figure, we used short names for basis sets, full names are in Table 1.

**Figure 5:**
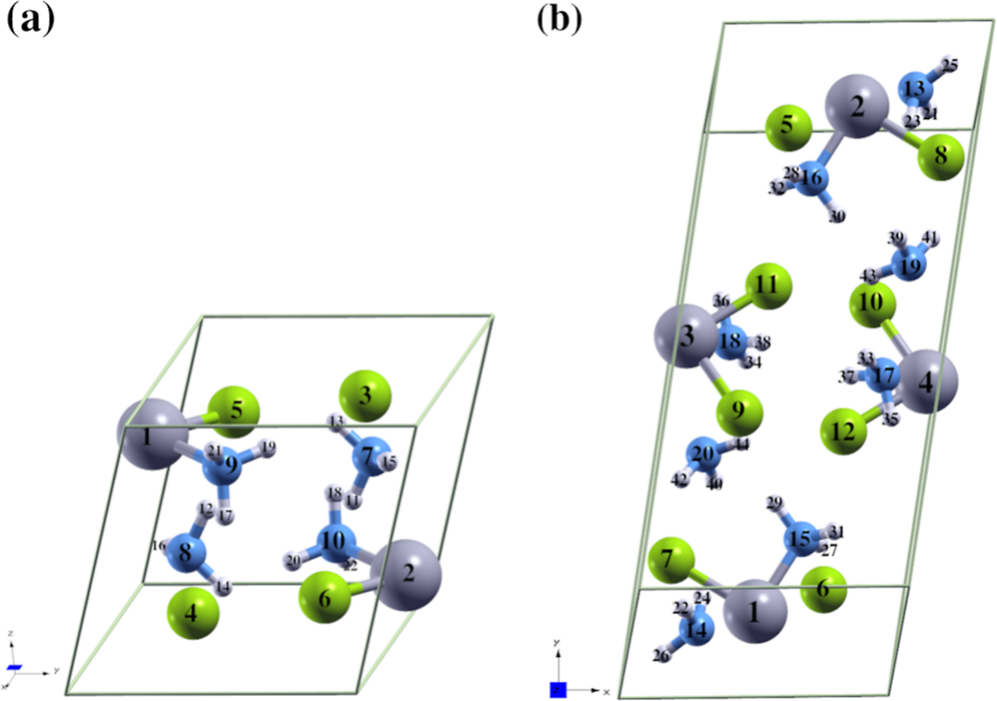
Unit cell of the polymorphs (a) α-Cisplatin and (b) β-Cisplatin. The gray, green, blue, and white balls correspond to the Pt, Cl, N, and H atoms, respectively.

The smallest and simplest Pt derivative is platinum hydride PtH, widely studied in experimental and theoretical work.^43−47^ Although this hydride has been studied for many years, its electronic states and the definition of the ground state were recently discussed in a theoretical study.^45^ Other derivatives are based on platinum halides, where platinum chloride is the smallest, PtCl.

It is important to highlight that, as described by Mardirossian and Head-Gordon^48^ in their review about the DFT and also investigated by Truhlar,^49^ the CCSD(T) method may not always be the gold standard reference for the density functional benchmark applied for the transition metal-based systems. This was also obtained by Süb et al. for both open-shell systems, such as PtH and PtCl.^40^ Then, we need to consider these previous results to justify our decision not to use CCSD(T) as the reference in our studies.

The other two compounds investigated have geometry similar to that of cisplatin.  has recently been studied in gas-phase calculations,^50^ and  can serve as a precursor compound for the synthesization of transplatin or cisplatin molecules.^51,52^ In addition to the five compounds, two cisplatin polymorphs (α and β) were investigated in the solid phase. Unlike the gas phase, the intermolecular hydrogen-bonding H···Cl was shown between neighboring cisplatin molecules due to orientations of the NH_3_ groups in each polymorph of the solid phase.^53^ The conversion of polymorphs affects the physical and chemical properties of pharmaceutical molecules such as cisplatin.^54^

Theoretical efforts have been made to study the influence of relativistic effects in heavy metals.^55−58^ For example, this implies an impact on the prediction of structural parameters, vibrational frequencies, and intramolecular force constants;^59^ this leads to the shortening of the bonds between the metal center and ligands;^60^ and this affects the dipole moment and molecular polarizability.^61^ Relativistic effects are not limited solely to heavy transition metals. Recent theoretical work demonstrated their influence on energy, geometry, harmonic vibrational frequencies, and polarizability in smaller systems composed of H to Xe atoms, including the first and second rows of transition metals.^62,63^

The Relativistic Effective Core Potential (RECP) approach, which incorporates relativistic effect through parametrization, has proven to be a popular choice for Pt-based complexes, particularly in large-sized cisplatin analogues.^8−10,41,64^ Their main goal is to reduce computational costs by avoiding explicit treatment of core–electrons while implicitly addressing major relativistic effects in the valence electron system.^65^ In the majority of the theoretical studies regarding anticancer Pt derivatives, the RECP basis set has been exclusively applied for the heavy metal, and then Pt is treated as a system with 18 electrons, while nonrelativistic all-electron basis sets were utilized for ligands containing nonmetal elements.^8,47,66−69^ On the other hand, the Zero-Order Regular Approximation (ZORA) and the approximate second-order Dirac equation derivated by Douglas–Kroll–Hess (DKH) Hamiltonian are considered the most popular Scalar Relativistic all-electron Hamiltonians with reduced complexity compared to the Dirac Hamiltonian.^70,71^ However, its utilization in Pt-based compounds appears to be less common.^60,72−74^

As the concept behind the parametrization of MMFF94 is the chemical environment and polarization of an atom in a molecule, which is described by the bci, this work aims to derive the bci values for platinum based on the MMFF94 approach. To achieve this goal, we compared different density functionals varying the basis sets to determine the best predictor levels of theory for structural parameters and harmonic vibrational frequencies. Ab-initio Hartree–Fock (HF) and Post-HF methods were also calculated. To derive the bci parameters from the atomic charges, an inverse problem was employed in a Python algorithm which is available free at https://github.com/molmodcs/bci_solver.

## Computational Methods

### Gas Phase

Quantum mechanical calculations were performed using Orca 5.0.3 software.^75−77^ All molecular structures were obtained with the Avogadro v.2.1 suite package.^30^ The analysis of the vibrational modes and infrared (IR) spectrum was performed with the ChemCraft.^78^ A systematic investigation on DFT methods was employed, and also we employed HF and Post-HF with the Perturbation Theory by the Møller–Plesset (MP2) approach in the same approach as used by Halgren’s work.^16^ Sixteen (16) different exchange–correlation functionals were examined, encompassing different steps from the Jacob's ladder.^79^ These functionals comprised the Minnesota meta-GGA functional M06L,^80^ the GGA hybrid functionals such as B3LYP,^81^ PBE0,^82^ and mPW1PW,^83^ the meta-GGA hybrid functionals like TPSSh,^84^ M06,^85^ and M062X,^85^ the range-separated hybrid functionals including LC-BLYP,^86,87^ ωB97X-D3BJ,^88^ and CAM-B3LYP,^89^ the GGA composite functional B97-3c,^90^ and double-hybrid functionals such as B2PLYP,^91^ mPW2PLYP,^92^ PBE0-DH,^93^ PBE-QIDH,^94^ and DSD-BLYP.^95^ Additionally, we tested the GGA functionals BP86 and PBE, as well as the GGA hybrids B3PW91 and B3P86, recommended by Neese and co-workers for the third row transition metal complexes,^69^ but only with the best basis set that resulted from the benchmark for each compound.

For Post-HF, the resolution of the identity RI-SCS-MP2 method,^96^ accelerated by the frozen core approximation, was chosen. The unrestricted HF method was applied for PtH and PtCl, while the restricted HF method was included for the remaining.

To accommodate various basis sets and relativistic effects, 11 distinct basis sets, including RAE, RECP, and RAEPt-NRAE, were considered (Table 1). Our basis set selection aims to compare three scenarios where relativistic effects are applied: first, only to the metal element though RECP; second, using fully scalar relativistic all-electron treatment with ZORA or DKH Hamiltonians (RAE) applied to both metal and nonmetal elements; and third, with relativistic all-electron treatment applied to the metal and nonrelativistic all-electron treatment for nonmetal elements (RAEPt-NRAE). Nonrelativistic basis sets for the metal were not considered since previous studies^10,47,55^ demonstrated the necessity of including such effects, especially for elements from the third transition series onward. However, the application of relativistic effects to nonmetal elements is still limited. Pople basis sets like the 6-311G family were excluded due to recent findings by Pitman et al.,^97^ concluding their inadequacy for applications necessitating precise electronic characterization, particularly for systems with significant dispersion and polarization effects. However, for comparison reasons, we only used the 6-31G(d,p) basis set since it was employed by Halgren in the original MMFF94 development. Levels of theory were organized from left to right, showcasing the method/basis set. For instance, TPSSh/SARC-ZORA-TZVP/ZORA-def2-TZVP represents a density functional/basis set for a Pt atom/basis set for nonmetal atoms. Another example, HF/def2-TZVP employs the ab initio method/only one basis set for all atoms.

**Table 1:** Basis Sets Used in This Work^f^

basis set full name	abbrev. name	type
RECP		
def2-TZVP^a^		Karlsruhe^98,99^
def2-QZVP^a^		
LANL2DZ^b^		Los Álamos^100−102^
LANL2TZ/def2-TZVP^c^		LA/Karl^98−100^
RAE		
SARC-ZORA-TZVP/ZORA-def2-TZVP^d^	ZORA-TZVP/TZVP	Van Lenthe^70,103^
SARC-ZORA-TZVPP/ZORA-def2-TZVP^d^	ZORA-TZVPP/TZVP	
SARC-DKH-TZVP/DKH-def2-TZVP^d^	DKH-TZVP/TZVP	Neese^71,103^
SARC-DKH-TZVPP/DKH-def2-TZVP^d^	DKH-TZVPP/TZVP	
RAEPt-NRAE		
Sapporo-DKH3-DZP-2012/Sapporo-DZP-2012^e^	Sappo-DZP/DZP	Sapporo^104,105^
Sapporo-DKH3-TZP-2012/Sapporo-DZP-2012^e^	Sappo-TZP/DZP	
Sapporo-DKH3-QZP-2012/Sapporo-DZP-2012^e^	Sappo-QZP/DZP	

a All-electron basis set with triple- and quadruple-ζ for H, Cl, N, and valence electrons of Pt; with the Stuttgart–Dresden def2-RECP for 60 core electrons of the Pt atom.

b All-electron Dunning D95 V double-ζ basis set for H, Cl, N, and valence electrons of Pt and Cl; with the Hay Wadt RECP for 60 core electrons for Pt and 10 core electrons for Cl atoms.

c Mixture of Los Alamos/Karlsruhe. The LANL2TZ triple-ζ for valence electrons with the Hay Wadt RECP replacing 60 core electrons of Pt. For N, Cl, and H atoms: def2-TZVP.

d For the Pt atom applied a segmented all-electron relativistically contracted basis set (SARC) for the scalar relativistic ZORA or DKH Hamiltonian, triple zeta TZVP added polarized functions TZVPP. For H, N, and Cl atoms: a relativistic recontracted all-electron Karlsruhe basis set for the ZORA or DKH Hamiltonian.

e For the Pt atom: a relativistic all-electron double-, triple-, and quadruple-ζ valence plus polarization function. For H, N, and Cl atoms: a nonrelativistic all-electron double-ζ basis set.

f Larger names were abbreviated (Abbrev. name).

A total of 18 levels (16-DFT + HF + MP2), each employing 11 basis sets, were investigated. An exception was MP2, which used 10 basis sets due to the absence of the def2-QZVP option. Furthermore, for comparison purposes, the level of theory HF/LANL2DZ/6-31G(d), used by Halgren in the parametrization of MMFF94,^17,23^ was also investigated. To facilitate the comparison of the benchmark values, color-coded tables (Heat Map) generated by the LibreOffice Calc program were utilized, with colors ranging from dark green (indicating higher accuracy) to dark red (indicating lower accuracy). Performance evaluation of each computational calculation was performed based on the relative deviation percentage (RD) (1) and mean absolute deviation percentage (MAPE) (2) for structural parameters and mean absolute deviation (MAD) (3) for harmonic vibration frequencies. In these evaluations, **N** represents the total number of parameters considered, **ref.value** denotes the experimental reference, and **calc.value** represents the calculated value. The closer the RD, MAPE, and MAD values are to zero, the more accurate the results will be when compared to experimental values.
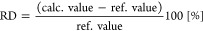
1
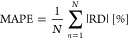
2
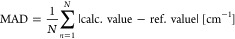
3

### Solid Phase

Bulk models for α- and β-Cisplatin polymorphs were built from the unit cells proposed by Ting et al.^53^ The crystalline system of both structures is triclinic and space group *P*1̅. The lattice parameters of the unit cell of the α-Cisplatin are *a* = 6.2424 Å, *b* = 6.5087 Å, *c* = 6.7451 Å, α = 69.896°, β = 83.710°, and γ = 87.348° and of the β-Cisplatin are *a* = 6.2846 Å, *b* = 13.4990 Å, *c* = 6.6839 Å, α = 69.444°, β = 85.586°, and γ = 82.229°.

Structural optimization calculations in the solid phase were performed using the Quantum ESPRESSO package,^106^ which is based on the DFT,^107,108^ with periodic boundary conditions. The Kohn–Sham one-electron states were expanded in a plane wave basis set until kinetic cutoff energies of 40 and 50 Ry (400 and 500 Ry for the density) for α- and β-Cisplatin, respectively. Monkhorst–Pack^109^ meshes of 4 × 4 × 4 and 4 × 2 × 4 *k*-point sampling in the first Brillouin zone were used for α- and β-Cisplatin, respectively. The effect of exchange–correlation potential was included with the Perdew–Burke–Ernzerhof (PBE) generalized gradient approximation.^110^ In addition, the Grimme scheme (D3) was used to correct van der Waals dispersion interactions.^111^ The internal electrons were represented with the projector augmented wave (PAW) method.^112^ For Pt, a relativistic PAW approach was used. The structures were allowed to relax until all residual force components were less than 10^–3^ Ry/Bohr, and the total energy difference was less than 10^–4^ Ry.

### Partial Atomic Charges

For the gas phase, the atomic charges were derived from CHELPG,^113^ Mulliken,^114^ and Hirshfeld^115^ methods. The reason for not using the CHELPG method for the solid phase is due to the unphysical results for periodic systems reported previously,^116^ which is explained by the fact that the electrostatic potential presents a constant shift at each point of the space.^117^ Therefore, Bader charges based on atoms in the molecule theory,^118,119^ Hirshfeld, and Mulliken methods were selected to study cisplatin polymorphs in the solid phase. Additionally, similar to partial atomic charges implemented in the standard MMFF94 approach, charges were calculated by the HF approximation with LANL2DZ as the basis set on Pt and 6-31G(d) Pople basis set on the nonmetal atoms.

We selected the three best levels of theory for the structural parameters and the harmonic vibrational frequencies for each compound in the gas phase. Two of our selected compounds, PtCl and PtH, are open-shell systems; therefore, we tested the stability of the wave function for the levels of theory selected. We found that all methods for PtCl yielded nonstable wave functions, except B2PLYP/Sappo-QZP/DZP, while for PtH, all resulted in stable wave functions (except M06L/DKH-TZVP/TZVP), as can be seen in the Supporting Information, Table S11.

For double-hybrid density functionals, we reported values obtained from the relaxed density for calculated first-order properties, such as charges and dipole moments, while second-order properties, such as polarizability, were obtained from the self-consistent field density.

CHELPG charges^113^ were fitted to reproduce the electrostatic potential calculated by the quantum mechanics procedure at selected points (“grid”). These charges aim to satisfactorily reproduce the electrostatic potential and dipole moment.

Mulliken population analyses were performed using the LOBSTER 4.1.0 package,^120^ and Bader–Hirshfeld population analyses were performed with the Critic2 package,^121,122^ in the solid phase. CHELPG, Mulliken, and Hirshfeld charges were obtained in populational analysis calculations with the Orca 5.0.3 program in the gas phase.

### bci Solver

We now describe the procedure taken by the bci solver Python tool to gather the necessary data and for solving this inverse problem
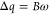
4for a given chemical structure, where *q* refers to the charges, *B* is a matrix that corresponds to the coefficients of each bci values, and ω refers to the bci.

This solver aims to recover information about the bci associated with the covalent bonds present in a chemical structure, using the quantum chemically derived charges. Halgren used this approach in his MMFF94, but it was also used to derive charges and to describe the polarization density between two bonded atoms in the classical force field functional forms applied to molecular simulations.^123−125^ MacKerrel et al. extended this idea to charge increments associated with angles and dihedrals for the CHARMM general force field (CGenFF) II.^125^ More precisely, given a chemical structure whose atoms are labeled, the partial atomic charge *q*_*j*_ of the *j*-th atom appearing in it is calculated through MMFF94^16,17,23,24^ as
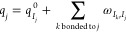
5

These values are defined as follows:

- *I*_*j*_ refers to the MMFF94 atom type of the *j*-th atom.
is the integral or fractional formal atomic charge associated with the atom type *I*_*j*_.
refers to the bci to atom *j* from the *j* – *k* bond with the atoms *j* and *k* of types *I*_*j*_ and *I*_*k*_, respectively.
- For any two given atom types *I*_*j*_ and *I*_*k*_, it holds that .

We follow Halgren’s previous approach^23,24^ and utilize a least-squares method to find approximate solutions to the above-mentioned linear system of equations. This procedure was implemented as a computer program written using Python and its libraries, NumPy, SciPy, and Matplotlib, for performing the necessary data manipulation, the least-squares method, and data visualization, respectively. The bci solver algorithms, files, and examples are available on the GitHub platform (https://github.com/molmodcs/bci_solver). Figure S13 in the Supporting Information describes details about the bci for the cisplatin structure, while Figures S24–S26show validated examples that reproduce well the bci values for few molecules in the MMFF94 database. Figure 1 depicts diagrammatically the functionality of the program, the necessary input files that it requires to run, and the generated output files.

For more information on using the bci solver and its documentation, check out the official GitHub page for the project.^126^ More details about this section are listed in the Supporting Information.

## Results and Discussion

Our findings are as follows: first, we evaluated the accuracy of different levels of theory, including DFT, HF, and Post-HF methods employing Perturbation Theory by Møller–Plesset (MP2), with respect to the agreement with experimental structural parameters of selected Pt complexes. Then, we calculated the RD percentage and MAPE for structural parameters and MAD for harmonic vibrational frequencies at the ground state in the gas phase. Moreover, we extended our investigation to include bulk-model analyses of the polymorphs of cisplatin in addition to conducting thorough calculations and examination of bci at the ground state in both gas and solid phases.

### Structural Parameters

Figure 2 shows the optimized geometries with the best level of theory for achieving the results. A comprehensive compilation of optimized bond lengths, bond angles, and dihedral angles is shown in the Supporting Information, Tables S1–S4, while the MAPE from experimental data is summarized in Figures S1–S5. The closer the MAPE values are to zero, the more accurate the results are when compared to experimental references.

We observe that the performance of the density functional methods and the basis set was closely related to the size and chemical environment of the investigated compounds. Figure 3 presents a subset of our findings, highlighting all investigated computational methods with a particular emphasis on the three most accurate density functionals within each class. Analysis across all groups, as depicted in Figure 3, indicates the ability to provide benchmark-quality data for platinum-based complexes. Among the employed methods, meta-GGA, GGA composite, Minnesota meta-GGA hybrid, and HF exhibit higher MAPEs compared to range-separated hybrid, GGA hybrid, and double-hybrid functionals for the majority of molecules considered. Specifically, double-hybrid functionals and Post-HF yield MAPEs exceeding 1.0% in the smallest PtH molecule, whereas the PtCl diatomic molecule showed the opposite trend. Nevertheless, double-hybrid functionals and Post-HF methods demonstrated minimal MAPEs when applied to the largest square-planar Pt(II) complexes such as , , and *cis*-[Pt(NH_3_)_2_Cl_2_].

From our results, mPW2PLYP/ZORA-TZVP/TZVP (double-hybrid functional) and LC-BLYP/def2-TZVP (range-separated hybrid functional) methods showed low MAPEs of 0.016% for PtCl and 0.005% for PtH, respectively, compared with experimental data.^127,128^ The meta-GGA hybrid nonlocal functional, “TPSSh”,^84^ also performed exceptionally well for both diatomic molecules, yielding MAPE values of 0.010% for PtH and 0.068% for PtCl, with def2-QZVP and LANL2TZ/def2-TZVP basis sets, respectively. The results with TPSSh showed superior predictive accuracy compared to previously reported data by Sub and co-workers.^40^

While the DSD-BLYP (double-hybrid functional) has shown a MAPE of 2.496% for cisplatin, the PBE-QIDH (another double-hybrid functional) exhibited low MAPEs of 0.348% and 0.049% for  and  with their corresponding basis sets over the methods, respectively. Interestingly, we observed a directly proportional relationship between the accuracy and the perturbative correlation MP2 nonlocal term (% MP2) of double-hybrid functionals for cisplatin, a phenomenon not observed for other molecules in this study. For instance, DSD-BLYP (60% MP2) predicted better than PBE0-DH (12.5% MP2). Our results highlight that all double-hybrid functionals presented good performance to predict the structural parameters.

The ionic complexes  and  exhibit a square-planar framework, where the metal center and ligands (Cl and N, respectively) lie in the same plane, with bond angles Cl–Pt–Cl and N–Pt–N ≈ 90°, respectively, compared to experimental data.^129,130^ Experimental Pt–Cl and Pt–N bond lengths for the solid state of the neutral species K_2_PtCl_4_^129^ and [Pt(NH_3_)_4_]Cl_2_^130^ were used for comparison, respectively. Nevertheless, Treviño and collaborators recently reported no significant counterion environmental effect on the Pt–Cl bond length.^50^

Additionally, most levels of theory calculated for cisplatin revealed a square-planar geometry corresponding to the *C*_2*v*_ symmetry, with hydrogen atoms H7 and H8 nearly lying within the molecular plane toward the chlorine atoms (see dihedral angles H–N–Pt–Cl ≈ 0° in Table S4 of the Supporting Information), consistent with the previous literature.^131^ This symmetry is characteristic of cisplatin in the gas phase, whereas the solid phase and aqueous solution disrupt the *C*_2*v*_ symmetry.^132^

Figure 3 shows that the Minnesota functionals, such as the meta-GGA M06-L and meta-GGA hybrids M06 and M06-2X, perform poorly for all square-planar Pt(II) compounds (, , and *cis*-[Pt(NH_3_)_2_Cl_2_]) of this study. This result is likely expected, as M06-2X was originally designed for nonmetal elements,^85^ and it has even been applied for transition-metal studies.^133^ M06-L,^80,85^ a local density functional, has been reported more efficient for the larger Pt-derivative carboplatin than for cisplatin.^133^ Nevertheless, of all meta-GGA hybrids used in this study, only TPSSh, the hybrid with the smallest HF exchange (10% HF_*x*_), provides more accurate predictions than M06 (27%) and M06-2X (54%) for the smallest diatomic molecules (PtH and PtCl).

The results shown in Figure 3 highlight the impact of relativistic effects on Pt derivatives for various basis sets. Among them, RECP shows superior accuracy for PtH and PtCl complexes compared with RAE and RAEPt-NRAE, as demonstrated by the MAPEs presented in Figure 3. The computed MAPEs for all Pt derivatives across all levels of theory are shown in Figures S1–S5, Supporting Information.

Figure 3 also shows that RECP basis sets have enhanced the efficiency of predicting the PtH and PtCl structural parameters. Another important conclusion is that the Karlsruhe basis set (i.e., def2-) ranks highly in terms of low MAPE values. However, RECP is less effective in predicting the optimized geometries of larger Pt(II) molecules, such as , , and *cis*-[Pt(NH_3_)_2_Cl_2_]. RAEPt-NRAE basis sets are useful for obtaining reliable geometrical analyses of larger systems, although RAE and RAEPt-NRAE become computationally expensive compared to RECP, particularly for square-planar frameworks of  and cisplatin.

Among the evaluated basis sets, the RAE basis set DKH-TZVPP/TZVP shows favorable accuracy with lower MAPEs across all functionals for the  system (see Figure 3). Recent literature has explored the application of the DKH basis set for tetrachlorinated-Pt complexes,^72^ while another DKH variant, DZP-DKH, has been used for all atoms in larger molecules.^134^ However, we observed no significant optimization when applying a scalar RAE basis set (i.e., DKH-TZVP/TZVP and DKH-TZVPP/TZVP) to the  complex, as illustrated in Figure S4 (see the Supporting Information).

RAEPt-NRAE Sapporo, Sappo-TZP/DZP, and Sappo-QZP/DZP basis sets, which include triple-ζ and quadruple-ζ valence plus polarization functions, improve the accuracy of structural parameter prediction for square-planar Pt(II) complexes with NH_3_ ligands, such as  and cisplatin. Specifically, Sappo-TZP/DZP provides good accuracy, yielding smaller MAPEs for the optimized structural parameters. This suggests that the presence of NH_3_ ligands bound to the Pt metal center is a critical factor in the performance of the basis sets for cisplatin rather than the chloride ligands. Previous studies on Sapporo basis sets^104,105^ have shown that the deviation for QZP is lower than for TZP when calculating bond lengths in smaller systems like diatomic hydrides and oxides. In our study, negligible deviation between TZP and QZP was observed when using these basis sets for larger systems, such as  and *cis*-[Pt(NH_3_)_2_Cl_2_].

Figure 3 also shows that the *cis*-[Pt(NH_3_)_2_Cl_2_] complex exhibits significant deviations (highest MAPE values) across all functionals when balancing accuracy and computational cost in larger systems. The compounds, arranged in an increasing order of their lowest MAPE (in %) values, are 0.005 (PtH) < 0.016 (PtCl) < 0.049 () < 0.348 () < 2.496 (cisplatin). This discrepancy is mainly due to the selection of the appropriate functional, which is heavily influenced by the molecular size. Our results show that cisplatin has the highest error among all molecules studied, a pattern that becomes more evident when comparing a monomer in the gas phase with a crystal in the solid phase.^132^ As noted, the solid phase of cisplatin exhibits distortions in symmetry and variations in the orientations of NH_3_ groups, which participate in the three-dimensional intermolecular bond network.^53^ However, recent studies reported smaller errors for cisplatin,^59^ with a 0.5% difference, although these studies used older reference data,^135^ which showed variations in bond angles compared to Ting’s work.^53^

The trend illustrated in Figure 3 generally follows the hierarchy of functionals in Perdew’s Jacob’s Ladder,^79^ where double hybrids outperform hybrids, and hybrids surpass meta-GGA functionals for cisplatin. The investigated functionals show moderately favorable performance in yielding accurate results. However, while the functionals largely confirm Perdew’s hierarchy for compounds like PtCl, , and , not all double hybrids consistently outperform hybrid functionals. Interestingly, for the smallest molecule, PtH, the trend diverges from Jacob’s Ladder, with hybrids and meta-GGA functionals yielding better results than double-hybrid functionals.

### Harmonic Vibrational Frequencies

The harmonic vibrational frequencies for all optimized geometries are shown in the Supporting Information, Tables S5–S8. MADs from experimental values were obtained to express the accuracy of the methods for PtH, PtCl, , , and cisplatin, all in their gas-phase ground state, as shown in Figure 4. The closer the MAD values are to zero, the more accurate the results are when compared with experimental references. We summarize the MAD values for each level of theory in Figures S6–S11.

The results are compared with the experimental IR spectra in the gas phase for PtCl^136^ and PtH^127^ and in the solid state for ,^137^,^130^ and cisplatin.^39^ Since no experimental vibrational frequencies are available for the ionic complexes, we used neutral species as references for comparison. Due to the broad range of functionals and basis sets used, we did not apply scaling factors to any of the molecular frequencies in this study, which is a tough work, and not all combinations of functionals and basis set used in our work were found in the reviewed literature.^138,139^

Diatomic molecules such as PtH and PtCl exhibit lower MADs, indicating better predictive accuracy, in line with their structural parameters. However, despite cisplatin showing large errors in structural predictions, the highest prediction errors for vibrational frequencies were observed in the  complex. Ranking the compounds by ascending MAD (in cm^–1^) values: 0.1 (PtH) < 0.4 (PtCl) < 18.9 () < 46.5 (cisplatin) < 74.9 (). The MADs increase with the size of the system. From the literature, it is possible to attribute the discrepancies in vibrational frequency predictions to systematic errors such as neglecting anharmonicity and incomplete electron correlation treatment.^140^ The lack of scaling factors in our analysis can also contribute to these discrepancies.

Figure 4 shows that the meta-GGA, Minnesota meta-GGA, and HF levels generally yield higher MADs compared to range-separated hybrids, GGA composites, and double-hybrid functionals for most compounds. In general, double-hybrid functionals perform better for predicting geometries than frequencies, with the exception for . The GGA composite functional B97-3c, known for including semiclassical corrections for dispersion,^90^ also performs well in predicting frequencies for cisplatin with an RAE basis set (DKH-TZVP/TZVP).

Popular GGA hybrid functional B3LYP and the Post-HF RI-SCS-MP2 method demonstrate minimal MADs, approaching zero, for PtH and PtCl, respectively, with their corresponding basis sets. However, neither B3LYP (20% exact exchange) nor PBE0 (25% exact exchange) was the most accurate for both geometry and frequency predictions in square-planar Pt(II) complexes.

The meta-GGA hybrid functional TPSSh shows better frequency predictions than for structural parameters when applied to square-planar Pt(II) complexes, such as  and cisplatin, using RAE basis sets. In contrast, for diatomic molecules such as PtH and PtCl, RECP basis sets provide significantly better structural predictions.

Figure 4 also highlights that RAE and RAEPt-NRAE basis sets outperform RECP basis sets in predicting frequencies for PtH, PtCl, , and cisplatin, except for , where RECP performs well with the def2-QZVP basis set. Among the tested methods, the scalar RAE DKH-TZVP/TZVP provides the best predictive accuracy for larger systems like cisplatin, while ZORA-TZVPP/TZVP excelled in smaller molecules like PtH and PtCl. For the  complex, the RAEPt-NRAE Sappo-DZP/DZP basis set proves to be the most accurate among the basis sets evaluated.

For the  complex with *D*_4*h*_ symmetry, which includes nine vibrational modes, three of which were selected, the double-hybrid PBE-QIDH (def2-QZVP) achieved the best frequency prediction with an MAD of 18.9 cm^–1^, outperforming previous ab initio HF/LANL2DZ results^141^ by 4.8 MAD points. Similarly, for the  complex, which has *D*_4*h*_ symmetry with *C*_3*v*_ symmetry for each NH_3_ group, and entails 45 vibrational modes, of which five were selected corresponding to NH_3_ groups, the range-separated hybrid LC-BLYP functional (Sappo-DZP/DZP) provided the smallest MAD (74.9 cm^–1^), despite differences in NH stretching modes compared to experimental solid-phase data.^142^

Cisplatin exhibits 27 vibrational modes; however, reports from the literature vary significantly, with some studies indicating 12^1,39^ or six^1,59^ vibrational modes in their experimental IR spectra. The primary discrepancy arises from whether the modes associated with the NH_3_ groups are included. To address this, we conducted a benchmark analysis both with and without these vibrational modes, as shown in Figures 4 and S12 of the Supporting Information.

Surprisingly, when NH_3_ modes are excluded, the RECP Los Alamos LANL2DZ basis set significantly improves IR predictions across the methods, except for MP2, as illustrated in Figure S12. This enhancement is not observed for the other investigated molecules. Conversely, when the NH_3_ modes are included, LANL2DZ performs poorly in predicting both geometry and frequency for all compounds, including cisplatin (see Figure 4). Recent work on LANL2DZ has emphasized geometric optimization for large cisplatin derivatives rather than detailed benchmark studies.^8,9,143^

Our results suggest that utilizing LANL2DZ as a single basis set for both metal and nonmetal elements may more effectively enhance IR spectrum predictions compared to structural parameters, especially when amine modes are excluded for cisplatin.

Moreover, our findings indicate that the highest errors occur when amine modes are included. These errors primarily stem from the symmetric and asymmetric stretching of the NH_3_ groups, likely linked to the dynamic rotation of NH_3_ around the Pt–N bond, as reported by Brieger and co-workers^130^ for the [Pt(NH_3_)_4_]Cl_2_ complex. This is also correlated to how the cisplatin molecule is packed in the solid phase as previously discussed here. A noteworthy observation is the comparison between results obtained from the best levels: the meta-GGA hybrid TPSSh/DKH-TZVP/TZVP yielded a MAD of 46.5 cm^–1^, three times higher than the range-separated hybrid LC-BLYP/LANL2DZ, which corresponds to the cases with and without NH_3_ modes, respectively.

In both scenarios, the functionals deviate from Jacob’s Ladder hierarchy concerning frequency, as hybrids tend to outperform double hybrids. However, they generally conform to the structural parameters of cisplatin. Our findings suggest that methods that perform well in predicting frequencies may not necessarily excel in predicting geometric parameters and vice versa. This observation aligns with previous works on cisplatin.^59,60^

Table 2 summarizes the results for the three optimal levels of theory for predicting structural parameters and harmonic vibrational frequencies across all methods and basis sets for the investigated species. It shows their MAD and MAPE values, including the results for the level of theory in the parametrization of MMFF94 by Halgren (Hartree–Fock/LANL2DZ/6-31G(d)).^17,23^ The data also highlights that good predictor functionals and basis sets for frequencies may not necessarily be good predictors of structural parameters, and vice versa, for all of the species in this work. Table 2 displays some functionals that accurately predict both parameters but require different basis sets, for instance, the range-separated hybrids CAM-B3LYP for PtH and LC-BLYP for  and double hybrids PBE-QIDH and DSD-BLYP for . Furthermore, double-hybrid functionals prove to be particularly effective in geometric screening, especially when dealing with square-planar Pt(II) derivatives. This also highlights the evident poor accuracy of the HF method for predicting geometries and frequencies in highly correlated systems. This is particularly important for this work since it proves that the standard method used by Halgren may not be suitable for platinum-based complex parametrization.

**Table 2:**
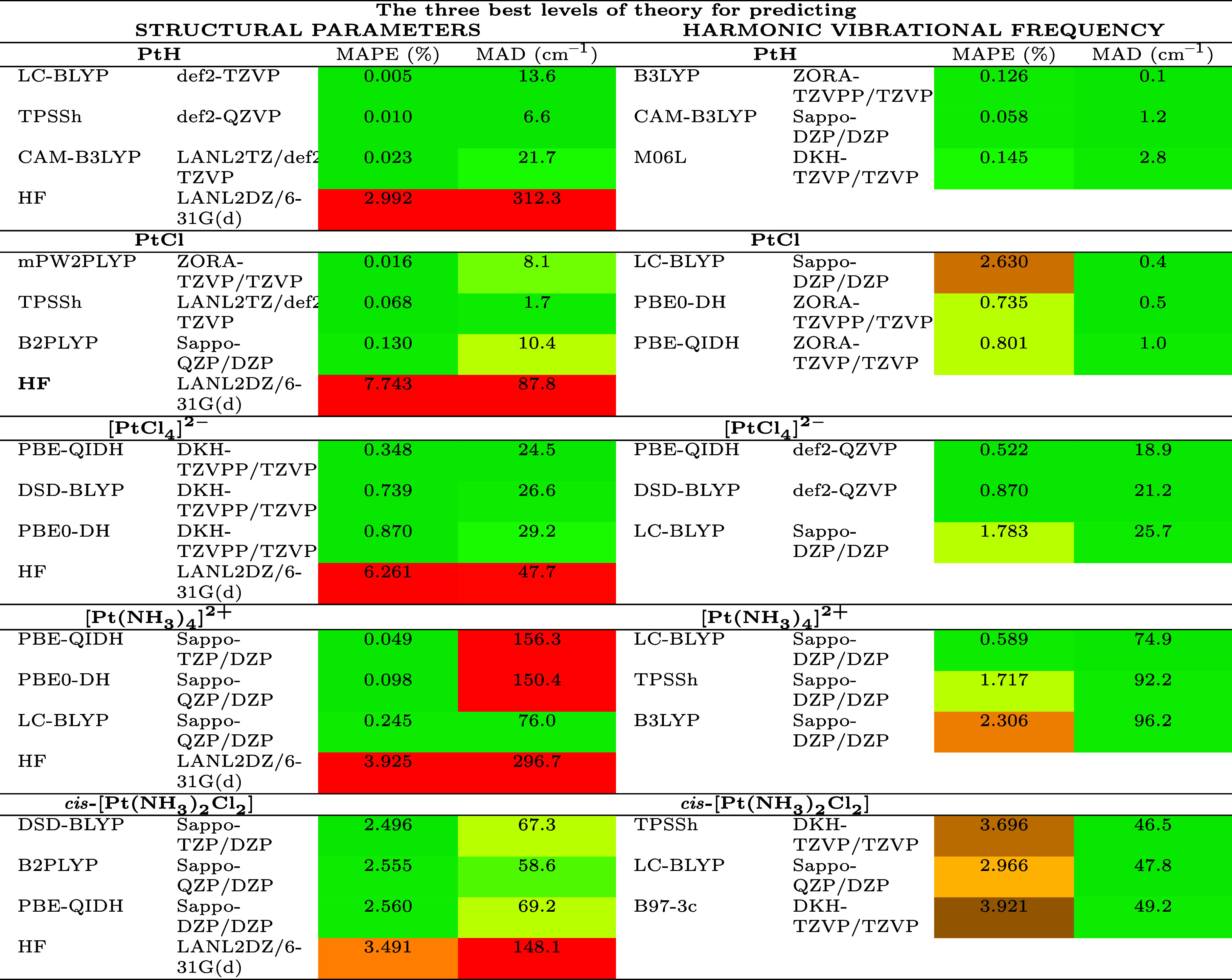
Selected Results from the Benchmark for the Prediction of Structural Parameters and Harmonic Vibrational Frequencies in the Gas Phase^a^

a The MAPE and MAD are included. The selected levels correspond to the three best predictors for structural parameters (left part) and the three for harmonic vibrational frequencies (right part). The level of theory used in standard MMFF94 (HF/LANL2DZ/···) is included. Color-coded tables generated by the LibreOffice Calc program are utilized for MAPE and MAD values, with colors ranging from dark green (indicating higher accuracy) to dark red (indicating lower accuracy). Abbreviated names for the basis sets are used here, with their full names listed in Table 1.

Specifically, for cisplatin geometries, the double-hybrid functionals play a predominant role in predicting the structure. However, it is important to note that the range-separated hybrid with dispersion-corrected ωB97X-D3BJ^88,144,145^ ranks as the next best functional after the double hybrids (as shown in Figure 3). This functional, belonging to the ωB97 family, has demonstrated effectiveness in capturing noncovalent interactions in benchmark studies.^48,146^ Additionally, it incorporates a damping function for treating dispersion interactions, making it an interesting choice for exploring larger Pt(II) and Pt(IV) complexes in future studies.

Our results for the GGA and hybrid GGA functionals such as BP86, PBE, B3P86, and B3PW91, with their respective basis sets (see Tables S1–S8 in the Supporting Information), were less predictive for structural parameters compared to our best levels of theory for all compounds in this work. The exception is the diatomic PtH, where PBE/def2-TZVP yielded a MAPE of 0.021%, which is comparable to our third-best level of theory (CAM-B3LYP/LANL2TZ/def2-TZVP). Our results differ from those reported by Neese and colleagues^69^ who conducted their study on other types of platinum compounds using different basis sets. Unlike these authors, we applied the suggested functionals to platinum derivatives of several sizes and incorporated a greater diversity of basis sets.

These findings suggest that using specific double-hybrid functionals rather than less computationally demanding hybrids effectively predicts structural parameters in medium-sized square-planar Pt(II) complexes. In contrast, hybrid functionals are more suitable for predicting harmonic vibrational frequencies across all Pt derivatives examined in this study (except for ), as detailed in the Supporting Information, see Figures S1–S11. Moreover, our work reaffirms the importance of utilizing basis sets with relativistic corrections, especially for platinum, in line with relevant studies reported in the literature.^56,59,147^ In summary, the choice of functionals and basis sets directly impacts the accuracy of molecular geometries and vibrational frequencies in computational chemistry calculations. With this, the atomic partial charges and the bci parameters were obtained and will be discussed in this work.

Finally, an important implication of our work is the inclusion of various types of double-hybrid functionals (B2PLYP, mPW2PLYP, PBE0-DH, PBE-QIDH, and DSD-BLYP) and the evaluation of their performance in predicting structural parameters and harmonic vibrational frequencies across platinum derivatives of different sizes. Relatively few studies have focused on these types of functionals for such systems.^39,40^ Consequently, our results contradict the functional hierarchy established by Jacob’s ladder in several cases, contrasting with major benchmark studies that did not include double-hybrid functionals but considered systems with metals other than platinum, adhering to Perdew’s approach.^48^

### Structural Parameters in the Solid Phase for the Cisplatin Molecule

Geometric optimizations were performed for the two polymorphs α- and β-Cisplatin used in this work. Figure 5 displays the optimized structures of both polymorphs, while Table 3 and the Supporting Information (Tables S9 and S10) present the structural parameters. The agreement between the experimental and calculated lattice parameters is very satisfactory, with the highest relative error found for the *b* parameter (1.43%) and the β parameter (1.95%) for α- and β-cisplatin, respectively.

**Table 3:** Comparison between the Experimental and Theoretical Lattice Parameters of the α- and β-Cisplatin^a^

lattice parameters	α-cisplatin			β-cisplatin		
	expt^1^	calc	RD (%)	expt^1^	calc	RD (%)
*a* (Å)	6.2424	6.2218	–0.330	6.2846	6.2901	0.088
*b* (Å)	6.5087	6.4154	–1.433	13.4990	13.3577	–1.047
*c* (Å)	6.7451	6.7697	0.365	6.6839	6.7008	0.253
α (deg)	69.896	69.4619	–0.621	69.444	69.918	0.683
β (deg)	83.710	82.9332	–0.928	85.586	83.917	–1.950
γ (deg)	87.348	87.3968	0.056	82.229	80.913	–1.600
MAPE			0.062			0.937

a Reference (53).

There is also good agreement between the calculated and experimental bond lengths and angles for both polymorphs. In general, except for some N–H distances and the angles associated with the hydrogen atoms (Pt–N–H) and (H–N–H), the bond lengths and angles are accurate to within 0.00–0.02 Å and 0.01–0.8°, respectively, for the two polymorphs. The angles Pt–N–H and H–N–H exhibit differences of up to 14.3° between the calculated and experimental data. Similar results were observed by Marques and collaborators.^148^ As stated previously by Berrêdo and Jorge,^147^ in the solid cisplatin structure, the intermolecular interactions become more important than the intramolecular hydrogen bonding, which is due to the steric repulsion between the neighboring molecules. This is one reason we decided to investigate the solid phase in order to see this effect on the charges and bci-derived values.

### Partial Atomic Charges in the Gas Phase

All of the partial atomic charges are shown in Tables 4, 5, and S12–S14 (in the Supporting Information). We have calculated the charges based on CHELPG, Hirshfeld, and Mulliken methods which are also described in the Supporting Information. For comparison reason, the CHELPG charges derived from the same level of theory employed by MMFF94 (HF/LANL2DZ/6-31G(d))^17,23,24^ are presented in Table 4 for cisplatin and the other compounds in their respective tables. We have used these charges to derive bci, which will be discussed in the next section.

**Table 4:** Partial Atomic Charges (e) on All Atoms of *cis*-[Pt(NH_3_)_2_Cl_2_] in the Gas Phase^a^

	3 best predicting structural parameters			3 best predicting frequencies		
	DSD-BLYP/Sappo-TZP/DZP			TPSSh/DKH-TZVP/TZVP		
atom	CHELPG	Hirshfeld	Mulliken	CHELPG	Hirshfeld	Mulliken
Pt	–0.02521	–0.04782	0.11478	–0.01859	–0.01809	0.33260
Cl2	–0.33530	–0.28769	–0.39988	–0.31571	–0.28467	–0.41383
Cl3	–0.33671	–0.28809	–0.40007	–0.31917	–0.28471	–0.41381
N4	–0.39325	–0.09487	–0.06204	–0.42569	–0.10053	–0.54101
N5	–0.40119	–0.09475	–0.06219	–0.42924	–0.10052	–0.54098
H6	0.26015	0.14114	0.12794	0.26935	0.13898	0.26306
H7	0.22269	0.12395	0.14823	0.21318	0.11606	0.26109
H8	0.22688	0.12423	0.14832	0.21666	0.11607	0.26109
H9	0.26098	0.14136	0.12844	0.26946	0.13919	0.26423
H10	0.25938	0.14135	0.12841	0.26952	0.13921	0.26435
H11	0.26158	0.14120	0.12806	0.27023	0.13901	0.26320

	B2PLYP/Sappo-QZP/DZP			LC-BLYP/Sappo-QZP/DZP		
	CHELPG	Hirshfeld	Mulliken	CHELPG	Hirshfeld	Mulliken
Pt	–0.03115	–0.03363	0.04672	0.00068	–0.00836	–0.00126
Cl2	–0.33038	–0.28982	–0.38598	–0.33933	–0.29960	–0.38242
Cl3	–0.33094	–0.29017	–0.38613	–0.34158	–0.29962	–0.38241
N4	–0.38386	–0.09671	–0.00725	–0.41305	–0.10756	0.01353
N5	–0.38906	–0.09660	–0.00741	–0.42555	–0.10753	0.01354
H6	0.25618	0.14016	0.11725	0.26640	0.14412	0.11815
H7	0.21849	0.12291	0.13510	0.22157	0.12303	0.13315
H8	0.22086	0.12314	0.13519	0.22834	0.12308	0.13316
H9	0.25692	0.14028	0.11760	0.26836	0.14414	0.11815
H10	0.25569	0.14027	0.11757	0.26592	0.14415	0.11819
H11	0.25724	0.14017	0.11735	0.26824	0.14415	0.11823

	PBE-QIDH/Sappo-DZP/DZP			B97-3c/DKH-TZVP/TZVP		
	CHELPG	Hirshfeld	Mulliken	CHELPG	Hirshfeld	Mulliken
Pt	0.00820	–0.01368	0.23319	–0.04994	–0.04916	0.30557
Cl2	–0.35439	–0.30410	–0.41797	–0.30689	–0.27593	–0.39662
Cl3	–0.35625	–0.30451	–0.41818	–0.30875	–0.27530	–0.39619
N4	–0.40284	–0.10171	–0.14706	–0.40158	–0.09666	–0.52287
N5	–0.40916	–0.10161	–0.14713	–0.40842	–0.09667	–0.52224
H6	0.26525	0.14363	0.14150	0.25712	0.14024	0.26430
H7	0.22441	0.12519	0.16509	0.20794	0.11835	0.25142
H8	0.22886	0.12545	0.16514	0.21211	0.11798	0.25084
H9	0.26541	0.14381	0.14189	0.26992	0.13845	0.25095
H10	0.26462	0.14383	0.14192	0.26970	0.13848	0.25082
H11	0.26589	0.14370	0.14162	0.25879	0.14021	0.26402

	standard MMFF94		
	HF/LANL2DZ/6-31G(d)		
	CHELPG	Hirshfeld	Mulliken
Pt	0.27629	0.14669	0.46497
Cl2	–0.45876	–0.37950	–0.38974
Cl3	–0.45994	–0.37839	–0.38944
N4	–0.52750	–0.10918	–1.14730
N5	–0.52860	–0.10912	–1.14646
H6	0.28595	0.14078	0.41987
H7	0.27662	0.13302	0.46412
H8	0.27918	0.13394	0.46376
H9	0.28548	0.13950	0.41731
H10	0.28604	0.14052	0.42003
H11	0.28525	0.14173	0.42288

a The selected levels of theory correspond to the three best predictors of structural parameters and the three for harmonic vibrational frequencies. The level of theory used in the parametrization of standard MMFF94 (HF/LANL2DZ/···) is included. Abbreviated names for the basis sets are used here, with their full names listed in Table 1.

**Table 5:** Partial Atomic Charges (e) on All Atoms of  in the Gas Phase^a^

	3 best predicting structural parameters			3 best predicting frequencies		
	PBE-QIDH/DKH-TZVPP/TZVP			PBE-QIDH/def2-QZVP		
atom	CHELPG	Hirshfeld	Mulliken	CHELPG	Hirshfeld	Mulliken
Pt	0.29999	–0.16918	0.28027	0.44168	–0.15493	0.85077
Cl2	–0.57397	–0.45771	–0.57007	–0.60937	–0.46117	–0.71269
Cl3	–0.57527	–0.45771	–0.57007	–0.61090	–0.46129	–0.71269
Cl4	–0.57510	–0.45771	–0.57007	–0.61038	–0.46117	–0.71270
Cl5	–0.57566	–0.45770	–0.57007	–0.61102	–0.46144	–0.71269

	DSD-BLYP/DKH-TZVPP/TZVP			DSD-BLYP/def2-QZVP		
atom	CHELPG	Hirshfeld	Mulliken	CHELPG	Hirshfeld	Mulliken
Pt	0.27217	–0.18711	0.26005	0.43299	–0.17046	0.81662
Cl2	–0.56743	–0.45324	–0.56501	–0.60819	–0.45741	–0.70416
Cl3	–0.56818	–0.45320	–0.56502	–0.60816	–0.45736	–0.70416
Cl4	–0.56830	–0.45324	–0.56501	–0.60846	–0.45741	–0.70416
Cl5	–0.56827	–0.45322	–0.56501	–0.60819	–0.45737	–0.70415

	PBE0-DH/DKH-TZVPP/TZVP			LC-BLYP/Sappo-DZP/DZP		
atom	CHELPG	Hirshfeld	Mulliken	CHELPG	Hirshfeld	Mulliken
Pt	0.30944	–0.15862	0.28267	0.31620	–0.11920	0.27365
Cl2	–0.57708	–0.46035	–0.57067	–0.57926	–0.47020	–0.56841
Cl3	–0.57736	–0.46034	–0.57067	–0.57899	–0.47021	–0.56841
Cl4	–0.57756	–0.46035	–0.57067	–0.57885	–0.47020	–0.56841
Cl5	–0.57745	–0.46034	–0.57067	–0.57910	–0.47020	–0.56841

	MMFF94		
	HF/LANL2DZ/6-31G(d)		
atom	CHELPG	Hirshfeld	Mulliken
Pt	0.58550	0.08519	0.26118
Cl2	–0.64618	–0.52143	–0.56530
Cl3	–0.64654	–0.52115	–0.56529
Cl4	–0.64632	–0.52143	–0.56530
Cl5	–0.64645	–0.52120	–0.56529

a The selected levels of theory correspond to the three best predictors of structural parameters and the three for harmonic vibrational frequencies. The level of theory used in the parametrization of standard MMFF94 (HF/LANL2DZ/···) is included. Abbreviated names for the basis sets are used here, with their full names listed in Table 1.

Our analysis, as presented in Table 4, reveals two distinct profiles for platinum charges across the three best levels of theory. The Mulliken method shows a significative positive charge on the Pt atom, while the CHELPG and Hirshfeld methods indicate a slightly negative charge. It is important to note that there are unique exceptions for the CHELPG charges obtained from LC-BLYP/Sappo-QZP/DZP and PBE-QIDH/Sappo-DZP/DZP, respectively.

Another characteristic of the CHELPG and Hirshfeld charges calculated for the Pt atom in **cisplatin** is their small magnitude, as shown in Table 4. This finding aligns with the classification of Pt(II) as soft acids,^149,150^ suggesting that the electron density transferred from the metal to the ligands is insufficient to fully neutralize the electron deficiency of Pt, resulting in a small negative charge. Similarly, the slightly positive values imply that the ligands donate nearly enough electron density to the metal, making it only slightly positive rather than neutral.

Both N and Cl^–^ ligands present negative charges across all selected levels of theory except when using LC-BLYP/Sappo-QZP/DZP, where Mulliken charges for N atoms were positive. This trend is in agreement with Lopes et al.^36^ (−0.41047e and −0.37495e) and de Berrêdo and Jorge^147^ (−0.39e and −0.48e) works, for N and Cl atoms, respectively. It is important to highlight that Lopes’s results were based on MP2/6-31G(d,p)/LANL2DZ (RECP) CHELPG charges, while de Berrêdo and Jorge derived Mulliken charges using mPW1PW/DZP (nonrelativistic basis set). de Berrêdo and Jorge also proposed a new contracted scalar relativistic basis (mPW1PW/DZP-DKH) set finding the charges −0.92e and −0.43e for N and Cl atoms for cisplatin, corroborating our results and indicating the importance of the relativistic effect to describe correctly the chemical environment around the central Pt atom.

In **cisplatin**, all H atoms from the amine ligands “NH_3_” present positive charges, displaying a charge transfer from hydrogens to the N atoms, which are much more electronegative (see Table 4). Across all selected functionals and basis sets, the H atoms exhibit higher positive charges than the central metal Pt in both CHELPG and Hirshfeld methods. However, one can see the inverse situation at certain levels of theory with Mulliken charges (TPSSh and B97-3c).

CHELPG and Hirshfeld charges on the hydrogens coplanar with Pt–N4–N5, specifically H7 and H8, are less positive than the respective charges on the hydrogens situated above and below the plane. However, the in-plane hydrogens H7 and H8 are more positively charged according to Mulliken charges in most cases (except for TPSSh and B97-3c).

Our results regarding charges on H atoms differ from those previously reported by de Berrêdo and Jorge,^147^ who showed no variations between H charges calculated using the Mulliken method in cisplatin with the mPW1PW/DZP-DKH level of theory. In contrast, our selected functionals correspond to double hybrids and long-range-correlated LC-BLYP with the Sapporo basis set. This suggests that differences in the selection of charge calculation methods, along with the choice of functional and basis sets, significantly influence the charge distribution, particularly in hydrogen atoms, within the cisplatin complex.

Table 5 presents the results for , and Tables S12–S14 show the results for , PtH, and PtCl, respectively. To summarize the discussion, details about the results for these species are given in the Supporting Information.

For , the calculated partial atomic charges reveal that Cl^–^ ligands consistently carry negative charges. Both CHELPG and Mulliken methods yielded similar, more negative charges for Cl^–^ compared with the Hirshfeld method. Naidoo and co-workers^151^ reported significant differences in the magnitude between the metal and chlorine ligands: +0.180e and −0.545e, respectively, for the dianion complex. They used the Merz–Kollman–Singh approach to derive their charges from semiempirical calculations. This trend appears for all charge methods with the double-hybrid functionals (PBE-QIDH, DSD-BLYP, and PBE0-DH with the DKH-TZVPP/TZVP basis set) that yield more accurate geometries and also with LC-BLYP/Sappo-DZP/DZP. Exceptions of this trend are the Mulliken charges related to PBE-QIDH and DSD-BLYP with the RECP basis set (def2-QZVP) that yield higher positive values for the metal. In a recent work, Treviño and Ermler^50^ reported similar magnitudes for the metal and chlorine ligands for the dianion complex obtained with B3LYP, HF, and MCSF.

Remarkably, as observed with the *cis*-[Pt(NH_3_)_2_Cl_2_] molecule, Hirshfeld charges yield negative values for the Pt atoms in  across all functional and basis set combination. In contrast, both CHELPG and Mulliken methods produced positive charges for Pt, with similar values across the three best-performing levels of theory for geometry optimization (PBE-QIDH, DSD-BLYP, and PBE0-DH), combined with scalar relativistic all-electron basis sets (DKH-TZVP/TZVP). But again, as for cisplatin, the Mulliken charges always overestimate the magnitude of the charges.

It is interesting and clear to note that the CHELPG charges present a slight charge fluctuation on Pt for all levels of theory investigated, and this behavior reproduces well the soft acid definition for Pt^2+^, as previously defined by Davies and Hartley.^150^ Another important conclusion is that if we used the same standard MMFF94 method developed by Halgren,^17^ all charges would be overestimated. However, the results that CHELPG charges give the best description of the chemical environment of the platinum in the cisplatin complex (see Table 4) are in agreement with the choice made by Halgren for MMFF94 development.

### Partial Atomic Charges for the Solid Phase of the Cisplatin Molecule

We have calculated partial atomic charges for the two **polymorphs** of cisplatin [Pt(NH_3_)_2_Cl_2_] using the Bader, Hirshfeld, and Mulliken methods, as presented in Tables 6 and S15 (in the Supporting Information). The results show that the Pt atoms consistently exhibit the highest positive charge across both Bader and Hirshfeld methods, indicating a strong tendency to receive electrons. In contrast, the Mulliken method assigns the highest positive charge to the H atoms.

**Table 6:** Partial Atomic Charges (e) of the α-Cisplatin^a^

atom	Bader	Hirshfeld	Mulliken
Pt1	0.54	0.10	0.19
Pt2	0.54	0.10	0.19
Cl3	–0.54	–0.14	–0.31
Cl4	–0.54	–0.14	–0.31
Cl5	–0.54	–0.15	–0.34
Cl6	–0.54	–0.15	–0.34
N7	–1.19	–0.16	–0.81
N8	–1.19	–0.16	–0.81
N9	–1.21	–0.15	–0.83
N10	–1.21	–0.15	–0.83
H11	0.49	0.08	0.34
H12	0.49	0.08	0.34
H13	0.49	0.08	0.34
H14	0.49	0.08	0.34
H15	0.48	0.08	0.35
H16	0.48	0.08	0.35
H17	0.50	0.08	0.36
H18	0.50	0.08	0.36
H19	0.51	0.08	0.34
H20	0.51	0.08	0.34
H21	0.46	0.09	0.36
H22	0.46	0.09	0.36

a Atoms are numbered according to Figure 5.

In the **α-cisplatin** polymorph (Table 6), all methods agree that the N atoms possess the most negative charge, signifying their potential as electron donors. However, for **β-cisplatin**, this trend is consistent only in the Bader and Mulliken methods, while the Hirshfeld method assigns the highest negative charge to the Cl atoms. It is interesting to note that this behavior and trend is in accordance with the charge distributions in the gas phase, indicating that the more negative charge is on the nitrogen atoms.

In both polymorphs, the Bader method presents the highest charges compared to the Hirshfeld and Mulliken methods across all atoms. Obtaining the bci from gas- and solid-phase calculations can give interesting insights about the chemical environment and polarity of the platinum–ligand bonds.

### Bond Charge Increment

It is important to highlight that, in the original work developed by Halgren for the parametrization of MMFF99,^17,23,24^ the bci values for metallic elements were obtained when they were in their ionic forms. The challenge here is that platinum does not exhibit a similar bonding or chemical environment to other metals studied in Halgren’s original work,^23^ which are mainly based on coordinated motifs.

**Figure 6:**
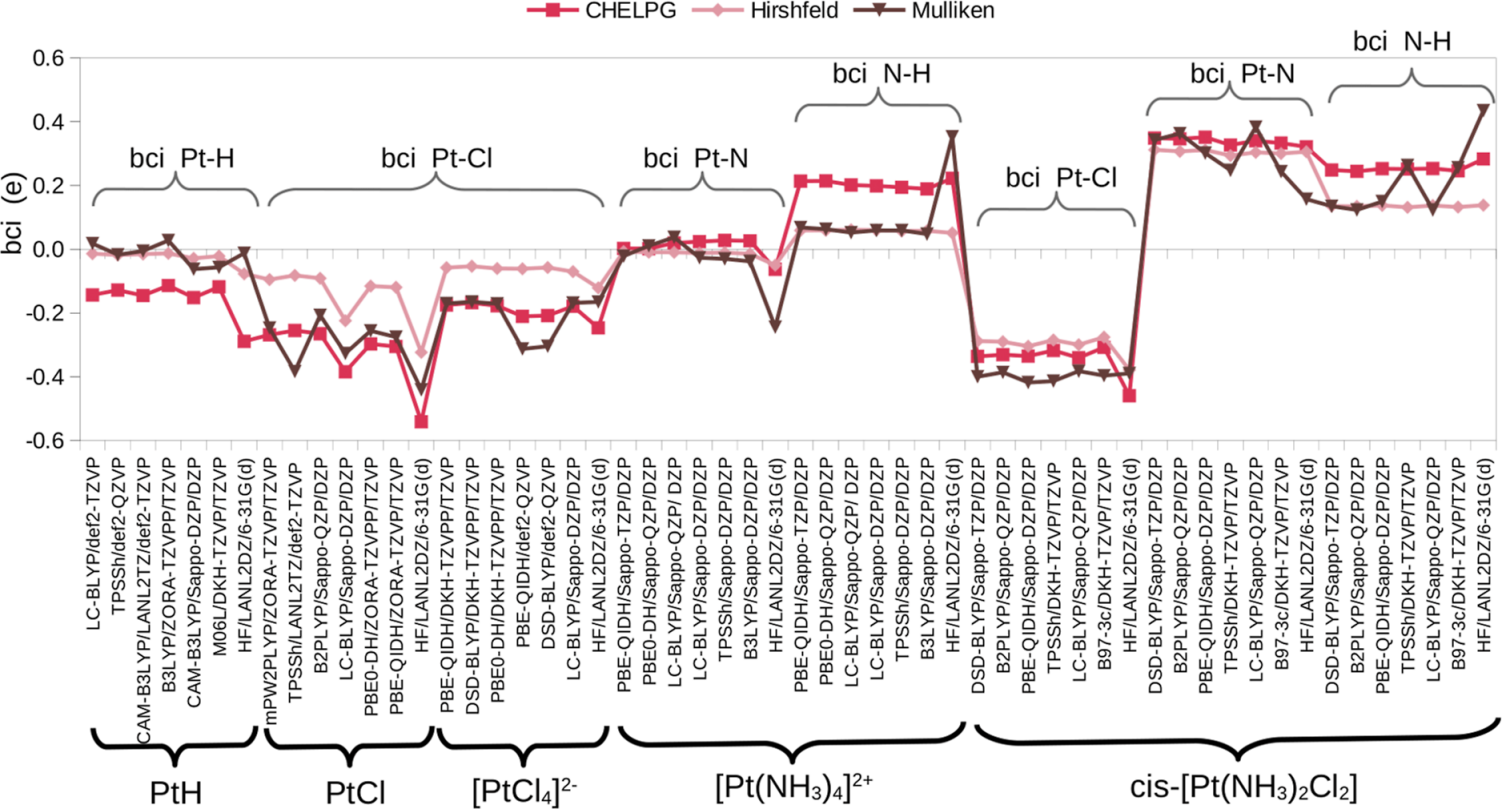
bci (e) for PtCl, PtH, , , and cisplatin [Pt(NH_3_)_2_Cl_2_] in the gas phase. The bci values are categorized by Pt–X (X = H, N, and Cl) and N–H bond types, as well as by the charge method used to derive them (CHELPG, Hirshfeld, and Mulliken). The selected levels of theory correspond to the three best predictors of structural parameters and the three for harmonic vibrational frequencies presented in Table 2. The level of theory used in the parametrization of standard MMFF94 (HF/LANL2DZ/···) is included. Abbreviated names for the basis sets are used here, with their full names listed in Table 1.

**Figure 7:**
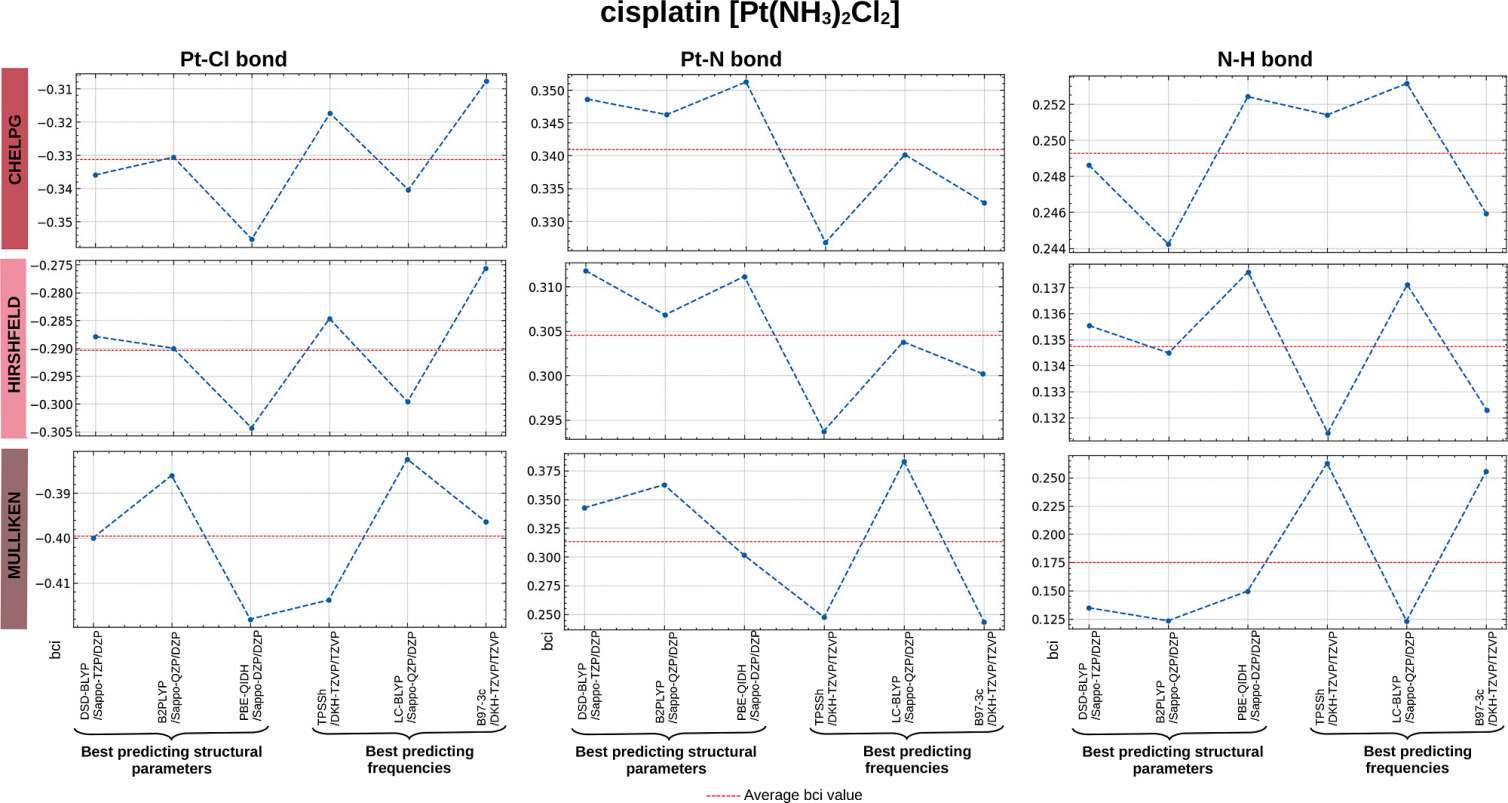
Computed bci (e) values for the Pt–Cl, Pt–N, and N–H bonds of the cisplatin molecule in the gas phase. The bci values are categorized by the charge method used to derive them (CHELPG, Hirshfeld, and Mulliken). The selected levels of theory correspond to the three best predictors of structural parameters and the three for harmonic vibrational frequencies presented in Table 2. Abbreviated names for the basis sets are used here, with their full names listed in Table 1.

**Figure 8:**
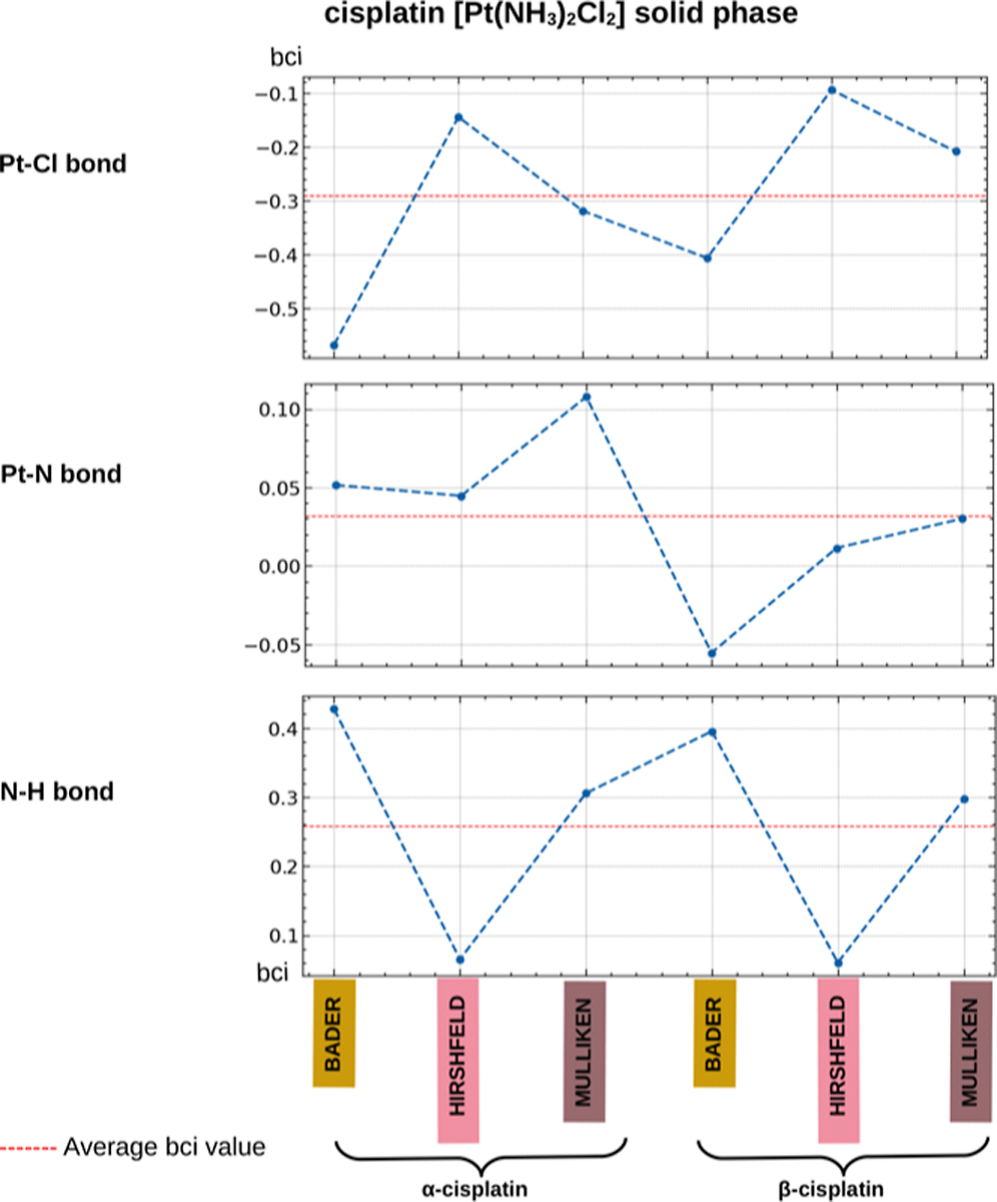
Computed bci (e) values for the Pt–Cl, Pt–N, and N–H bonds of α-cisplatin and β-cisplatin in the solid phase. The bci values are categorized by the charge method used to derive them (Bader, Hirshfeld, and Mulliken).

Using the bci solver tool, we generate the bci values from CHELPG, Hirshfeld, and Mulliken charges for all compounds in the gas phase and from Bader, Hirshfeld, and Mulliken for cisplatin in the solid phase. For clarity, we refer to each bci value derived from a specific charge method as “bci-charge-method” (e.g., bci-CHELPG). We report all results in Tables S16 and S17 (Supporting Information) and also in Figures 6–8 and S14–S17 (Supporting Information).

We summarize the calculated bci differentiated by the bond for all compounds in the gas phase, Figure 6. The results show different characteristics in the magnitude and sign of the bci values, depending on the bond type, charge method, and, to a lesser extent, the level of theory. For example, the bci values for the Pt–Cl and Pt–H bonds are negative, while the bci values for the Pt–N and N–H bonds are positive in all cases with few exceptions.

Therefore, we observe that the absence of correlation effects is reflected in the bci values, as shown by the higher magnitudes of the values using the Hartree–Fock method (HF/LANL2DZ/6-31G(d)) compared with those from DFT in most cases.

#### bci Pt–N

We present the bci for the Pt–N bond in two square-planar Pt(II) complexes: , which contains four identical ligands, and *cis*-[Pt(NH_3_)_2_Cl_2_], which has two different types of ligands. The NH_3_ ligand is neutral with N being more electronegative than H, leading to a polarized bond. However, the symmetry of the amine group (*C*_3*v*_) results in an overall nonpolar tetrahedral complex , with *D*_4*h*_ symmetry. In contrast, cisplatin, with its two additional anionic halide ligands, forms a molecule with *C*_2*v*_ symmetry and with significant polarity. This distinction affects the charge distribution within the bond of each complex.

Based on our geometry optimizations, we found that the Pt–N bond length in  is shorter than in *cis*-[Pt(NH_3_)_2_Cl_2_] across all levels of theory (see Tables S2 and S3 in the Supporting Information).

As shown in Figure 6, we observe higher and more positive bci Pt–N values in cisplatin, while  exhibits lower magnitude values, which could be either positive or negative.

All charge calculation methods (CHELPG, Hirshfeld, and Mulliken) yield positive bci Pt–N values for cisplatin. In contrast, negative values for all bci-Hirshfeld and some bci-Mulliken calculations for  were found. This behavior arises because, in , the magnitude of the CHELPG charges on Pt was much smaller than the magnitude of the charges on the N atoms, resulting in positive bci-CHELPG values. However, the Hirshfeld and Mulliken charges, which had much larger magnitudes for the Pt atom and smaller values for the N atoms, gave negative bci values. Furthermore, bci-CHELPG and bci-Hirshfeld have provided more consistent results across both complexes, whereas bci-Mulliken exhibits greater variability.

#### bci Pt–Cl

We also investigated the bci for the Pt–Cl bond type in two square-planar Pt(II) complexes ( and cisplatin [Pt(NH_3_)_2_Cl_2_]), as well as in linear PtCl. Each complex features four, two, and one Cl^–^ ligands, respectively. Theoretical studies have shown that the Pt–Cl bond exhibits covalent characteristics, as seen in carboplatin,^133^ a cisplatin derivative where chloride ligands are replaced by Pt–O bonds. Similarly, in cisplatin, theoretical data align with experimental findings, showing that the Pt–Cl covalent bond is stronger than the Pt–NH_3_ dative bond.^66^ It is also important to note that the relatively weak covalent nature of the Pt–Cl bond facilitates the hydrolysis of the Cl^–^ ligands, generating the active aquacomplexes within the cellular environment.^152^

All bci Pt–Cl values are negative with variations in magnitude influenced by differences in geometry. Despite these variations, we observe consistent trends in the calculated bci values across different theoretical levels and geometries. As seen in Figure 6, cisplatin displays the least dispersed bci Pt–Cl values, following the same trend as the bci for Pt–N. In contrast, one can see greater dispersion in  and PtCl, regardless of the charge calculation method. According to our calculations, the increasing order of the Pt–Cl bond length is PtCl < *cis*-[Pt(NH_3_)_2_Cl_2_] < , consistent across all levels of theory (see Tables S1–S3 in the Supporting Information). However, we found that the order of bci values is reversed, with cisplatin < PtCl <  for all bci-CHELPG, bci-Hirshfeld, and bci-Mulliken methods. In contrast, for systems where all four ligands are NH_3_ groups, as mentioned earlier, the bci Pt–N values for cisplatin exceeded those for . This difference could be influenced by the nature of the Cl^–^ ligands, which increase the polarity of the bond more than the NH_3_ ligands do.

#### bci Pt–H

We analyzed the bci for the Pt–H bond in platinum hydride. In all cases, negative values were obtained with bci-CHELPG showing the most negative values. The CHELPG partial atomic charges give a much larger magnitude for the Pt atom in PtH compared with other charge methods. We observe an exception of positive values by bci-Mulliken, corresponding to a negative sign for the Pt metal, especially with LC-BLYP/def2-TVZP and B3LYP/ZORA-TZVPP/TZVP, see Figure 6.

The two compounds selected for this study, PtH and platinum chloride, are referred to in the literature as possible ionic species.^128^ Our results show less negative bci values when Pt was bonded to the H^–^ across all charge calculation methods. This finding highlights how bci values differentiate species in single bonds with Pt, as H^–^ is less electronegative than the Cl^–^. In the case of Pt–halides, the bond is more polar than in the hydride.

#### bci N–H

The bci for the N–H bond type is present in all NH_3_ ligands of  and *cis*-[Pt(NH_3_)_2_Cl_2_]. In all cases, bci yields positive values with cisplatin showing a tendency toward more positive values across all selected levels of theory, as illustrated in Figure 6. Comparing each type of bci across different charge calculation methods (bci-CHELPG, bci-Hirshfeld, and bci-Mulliken) between both compounds, small differences in the values for the bci of the N–H bond length compared to the bonds involving the Pt metal (Pt–N and Pt–Cl) were observed. This result was expected, as the metal exerts a much stronger influence on the atoms directly bonded to it, affecting the charge density distribution.

As previously mentioned, we focus on bci-CHELPG values since it was the choice by MMFF94 development. We compile and arrange the bci values derived from the CHELPG method for the gas phase, ordering them as follows: For the **Pt–Cl** bond type, it was found: PtCl [−0.384 to −0.255] < *cis*-[Pt(NH_3_)_2_Cl_2_] [−0.341 to −0.308] <  [−0.210 to −0.168]. For **Pt–N**:  [0.002 to 0.028] < *cis*-[Pt(NH_3_)_2_Cl_2_] [0.327 to 0.351]. In addition, for **N–H**:  [0.189 to 0.214] < *cis*-[Pt(NH_3_)_2_Cl_2_] [0.244 to 0.253] and **Pt–H**: PtH [−0.152 to −0.114]. All calculated bci values are summarized in the Supporting Information (Table S16).

The obtained values for the selected tetracoordinate Pt(II) complexes are slightly lower in magnitude than those reported by Anastasi and Deeth.^34^ These authors reported bci from MM calculations based on MMFF94 and quantum mechanics (HF/LANL2DZ(f)/6-31G(d)). Smaller variations were observed when we obtained the MAD errors between our average bci-CHELPG values and those of the cited reference; for example, in cisplatin when the ligand is Cl^–^ (MAD 0.035e) and N (MAD 0.071e). The largest variations were obtained for  (MAD 0.177e) and  (MAD 0.395e), see Table S16 in the Supporting Information.

We note these variations in the bci of Pt–Cl and Pt–N bonds but not for Pt–H or for N–H across different systems. In this context, the bci-CHELPG and bci-Hirshfeld clearly highlight these differences, whereas bci-Mulliken does not in all cases, see Figures 7 and S14–S17 (Supporting Information).

Regarding the two polymorphs of cisplatin in the solid phase, significant variations in the bci of the **Pt–Cl** and **Pt–N** bond were found, while minimal variations were found in the bci of N–H between the α and β forms of cisplatin. Figure 8 illustrates smaller bci values for the Pt–Cl bond and larger values for Pt–N in **α-cisplatin** compared to **β-cisplatin**, regardless of the charge model (Bader, Hirshfeld, and Mulliken) used to derive the bci values. All bci values for the polymorphs of cisplatin are reported in Table S17 (Supporting Information).

Finally, we compared the bci values calculated in the **gas phase** and in the **solid phase** for cisplatin and its polymorphs from several charge methods, reported in Tables S16 and S17. In general, both phases yield negative bci values for Pt–Cl, while positive bci values for Pt–N and N–H bonds. One exception is bci-Bader in the solid phase of β-cisplatin with a negative bci for Pt–N.

Higher positive values for the bci of the **Pt–N** bond in the gas phase across all charge types (bci-CHELPG, bci-Hirshfeld, and bci-Mulliken) were observed. In contrast, for the **Pt–Cl** and **N–H** bonds, the bci from the gas phase present intermediate values compared to those in the solid phase. We highlight the good agreement of bci-CHELPG gas with the bci-Mulliken solid of α-cisplatin.

In summary, a crucial characteristic of square-planar Pt(II) complexes is the “dative” covalent interactions between the metal and ligands as shown by Obeng and Autschbach.^153^ In contrast, linear single-bond Pt systems, such as platinum hydride and platinum chloride, behave more like ionic compounds.^128^ In this context, the average calculated bci values effectively represent the chemical environment of Pt in the investigated species, falling within the expected bci value distribution of the standard MMFF94 protocol, as shown in Figures S18–S23 of the Supporting Information. It is also important to note that the partial atomic charge-derived bci values highlight that we do not need to perform all calculations for many species since the bci parameter captures the charge fluctuations between the bonded atoms in a molecule.

At this point, we emphasize that the bci solver can deliver optimized values from the atomic partial charges that have reasonable physical meaning in terms of the chemical environment of a bonding, as described by MMFF94 development.^16,23,24^ This investigation holds significant promise for future work, particularly in the new optimization needed for the bci solver developed in this study or even for new atom types in the MMFF94 approach. In the present work, we studied a few platinum complexes, developing a bci solver able to provide the bci parameters from the quantum chemistry-derived charges. Further DockThor implementation and tests should provide a validation and a more detailed understanding of these bci values for the Pt atom in molecular docking calculations and also can be used for a self-consistent optimization of the developed approach.

## Conclusions and Outlook

The benchmark included 16 density functionals, covering nonhybrids, hybrids, and double hybrids. In addition, the HF and Møller–Plesset perturbation theories at the MP2 level were evaluated. The study also examined 11 basis sets, contrasting relativistic all-electron methods with RECP approaches. We found that methods and basis sets that perform well in predicting vibrational frequencies may not necessarily excel in predicting geometric parameters and vice versa, and their performance is influenced by the size and chemical environment of the selected species. We show that DFT methods performed better than HF and Post-HF (MP2) methods for all of the compounds and parameters. Exceptions for the cisplatin geometry and for PtCl vibrational frequencies yield better results with the MP2 approach. We found that double-hybrid functionals proved to be effective in geometric screening, especially for medium-sized Pt(II) derivatives.

We have derived the bci values for the platinum atom based on the MMFF94 approach. Partial atomic charges were derived from benchmark density functional calculations for structural parameters and harmonic vibrational frequencies for the square-planar Pt(II) complexes , , cisplatin [Pt(NH_3_)_2_Cl_2_], and also for PtCl and PtH in the gas phase. On the other hand, the α and β cisplatin polymorphs were studied in the solid phase.

CHELPG partial atomic charges present a slight charge fluctuation on Pt for all of the levels of theory investigated, and this behavior reproduces well the soft acid definition for Pt^2+^, resulting in the best description of the chemical environment of the platinum in the cisplatin complex.

All of the bci values have shown narrow variations regardless of the methods and basis set used, particularly when calculated in the gas phase. A slight fluctuation was noted for the bci values of the Pt–Cl and Pt–N bonds, whereas the bci values of the N–H bond remained unaffected.

We found that the presence of NH_3_ ligands bound to the metal center is a critical factor in the cisplatin molecule: for the performance of the basis sets rather than the Cl^–^ ligands in geometry optimization, for the performance of levels of theory when including or excluding the NH_3_ vibrational modes, and for differentiation of the charges of the hydrogens coplanar with the coordinate plane in cisplatin.

We also show that the calculated bci yields different characteristics in the magnitude and sign of values depending on the bond type, charge method, and level of theory. These bci values reveal different bonding and chemical environments for the platinum compared with other metals in the original parametrization of MMFF94. We obtained individuals and average bci values for the Pt–H, Pt–Cl, Pt–N, and N–H bonds considered in this study. Our average bci values, in magnitude, for the square-planar Pt(II) complexes from CHELPG charges are 0.257 for Pt–Cl, 0.179 for Pt–N, and 0.226 for N–H. For the diatomic small-sized molecules: 0.133 for Pt–H and 0.296 for Pt–Cl.

The developed bci solver proved to be efficient to provide the bci parameters from quantum chemistry-derived charges. Further applications and optimizations will be important to validate the bci values for platinum in molecular docking using the DockThor program. We have provided an open-source implementation of all bci solver algorithms from Jupyter-Notebook and terminal applications, which is available on GitHub (https://github.com/molmodcs/bci_solver).

## Supporting Information Available

Structural parameters, harmonic vibrational frequencies, MAPE, and MAD for all compounds in the gas phase; structural parameters of the two cisplatin polymorphs in the solid phase; stability of the functional for the levels of theory for the two open-shell systems; partial atomic charges for all compounds in the gas phase; more details about the methodology, the matrix representation for cisplatin, bci values calculated for all compounds, and additional examples for small organic compounds for the bci solver; and electronic properties of all compounds in the gas phase (PDF)
Geometries of cisplatin, PtCl, [PtCl_4_]^2–^, PtH, and [Pt(NH_3_)_4_]^2+^ (ZIP)
