# Supplementary material for: Methodological Approach Based on Structural Parameters, Vibrational Frequencies, and MMFF94 Bond Charge Increments for Platinum-Based Compounds
Source: ACS Omega. 2025 Feb 20;10(8):8314–35. doi: 10.1021/acsomega.4c10141 (PMC11886664; doi:10.1021/acsomega.4c10141)
Supplement: Supplementary file 1 — ao4c10141_si_001.pdf [file ao4c10141_si_001.pdf]

SUPPORTING INFORMATION
Methodological Approach based on Structural Parameters, Vibrational Frequencies and MMFF94 Bond Charge Increments for Platinum-Based Compounds

Gloria Castañeda-Valencia,[†] Lucas F Gama,[†] Murugesan Panneerselvam,[†] Viviane Vaiss,[†] Isabella Guedes,[‡] Laurent Dardenne,[‡] and Luciano T. Costa[*,¶]

†*MolMod-CS, Institute of Chemistry, Fluminense Federal University, Campus Valonguinho,Centro, Niterói-RJ, CEP 24020-141*

‡*Laboratório Nacional de Computação Científica, Avenida Getúlio Vargas, 333, Quitandinha, Petrópolis - RJ, CEP 25651-075*

¶*MolMod-CS, Institute of Chemistry, Fluminense Federal Uniersity, Campus Valonguinho,Centro, Niterói-RJ, CEP 24020-141*

E-mail: ltcosta@id.uff.br

Structural parameters calculated with all methods and basis sets in the gas phase

PtH and PtCl

Table S1: Calculated Bond Lengths (in Å) for Pt-H and Pt-Cl in PtH and PtCl molecules, with Relative Deviation (RD in %) in parentheses. The level of theory used in the standard MMFF94 force field is included (HF-by-MMFF94). Abbreviated names for the basis sets are used here, with their full names listed in Table 1.

Method	Basis set	PtH	PtCl
Experimental	-	1.528 [1]	2.153 [2]
M06L	def2-TZVP	1.534 (0.41)	2.216 (2.94)
M06L	def2-QZVP	1.532 (0.24)	2.208 (2.56)
M06L	LANL2DZ	1.544 (1.02)	2.332 (8.32)
M06L	LANL2TZ/def2-TZVP	1.538 (0.68)	2.162 (0.40)
M06L	ZORA-TZVP/TZVP	1.535 (0.44)	-
M06L	ZORA-TZVPP/TZVP	1.534 (0.37)	2.202 (2.28)
M06L	DKH-TZVP/TZVP	1.526 (-0.14)	-
M06L	DKH-TZVPP/TZVP	1.525 (-0.18)	2.195 (1.96)
M06L	Sappo-DZP/DZP	1.536 (0.55)	2.231 (3.65)
M06L	Sappo-TZP/DZP	1.535 (0.47)	2.218 (3.03)
M06L	Sappo-QZP/DZP	1.531 (0.22)	2.216 (2.91)
B3LYP	def2-TZVP	1.532 (0.26)	2.228 (3.48)
B3LYP	def2-QZVP	1.528 (0.03)	2.222 (3.22)
B3LYP	LANL2DZ	1.543 (1.00)	2.342 (8.76)
B3LYP	LANL2TZ/def2-TZVP	1.532 (0.24)	2.173 (0.95)
B3LYP	ZORA-TZVP/TZVP	1.527 (-0.04)	2.170 (0.81)

to continued ...

Method	Basis set	PtH	PtCl
B3LYP	ZORA-TZVPP/TZVP	1.526 (-0.13)	2.164 (0.52)
B3LYP	DKH-TZVP/TZVP	1.518 (-0.67)	2.163 (0.48)
B3LYP	DKH-TZVPP/TZVP	1.516 (-0.76)	2.156 (0.13)
B3LYP	Sappo-DZP/DZP	1.530 (0,13)	2.194 (1.89)
B3LYP	Sappo-TZP/DZP	1.527 (-0.05)	2.179 (1.19)
B3LYP	Sappo-QZP/DZP	1.524 (-0.26)	2.176 (1.05)
mPW1PW	def2-TZVP	1.522 (-0.37)	2.203 (2.32)
mPW1PW	def2-QZVP	1.519 (-0.58)	2.198 (2.07)
mPW1PW	LANL2DZ	1.534 (0.38)	2.322 (7.86)
mPW1PW	LANL2TZ/def2-TZVP	1.524 (-0.26)	2.150 (-0.14)
mPW1PW	ZORA-TZVP/TZVP	1.519 (-0.61)	2.195 (1.94)
mPW1PW	ZORA-TZVPP/TZVP	1.517 (-0.70)	2.191 (1.78)
mPW1PW	DKH-TZVP/TZVP	1.509 (-1.24)	2.189 (1.65)
mPW1PW	DKH-TZVPP/TZVP	1.508 (-1.33)	2.184 (1.45)
mPW1PW	Sappo-DZP/DZP	1.521 (-0.47)	2.218 (3.04)
mPW1PW	Sappo-TZP/DZP	1.518 (-0.65)	2.204 (2.38)
mPW1PW	Sappo-QZP/DZP	1.515 (-0.83)	2.203 (2.32)
PBE0	def2-TZVP	1.522 (-0.37)	2.200 (2.18)
PBE0	def2-QZVP	1.519 (-0.59)	2.194 (1.91)
PBE0	LANL2DZ	1.535 (0.43)	2.320 (7.76)
PBE0	LANL2TZ/def2-TZVP	1.524 (-0.27)	2.147 (-0.30)
PBE0	ZORA-TZVP/TZVP	1.519 (-0.59)	2.192 (1.82)
PBE0	ZORA-TZVPP/TZVP	1.517 (-0.69)	2.189 (1.65)
PBE0	DKH-TZVP/TZVP	1.509 (-1.22)	2.186 (1.53)
PBE0	DKH-TZVPP/TZVP	1.508 (-1.32)	2.181 (1.32)

to continued ...

Method	Basis set	PtH	PtCl
PBE0	Sappo-DZP/DZP	1.521 (-0.48)	2.216 (2.95)
PBE0	Sappo-TZP/DZP	1.518 (-0.65)	2.202 (2.26)
PBE0	Sappo-QZP/DZP	1.515 (-0.82)	2.201 (2.22)
TPSSh	def2-TZVP	1.531 (0.17)	2.208 (2.53)
TPSSh	def2-QZVP	1.528 (-0.01)	2.202 (2.29)
TPSSh	LANL2DZ	1.539 (0.74)	2.329 (8.16)
TPSSh	LANL2TZ/def2-TZVP	1.530 (0.15)	2.152 (-0.07)
TPSSh	ZORA-TZVP/TZVP	1.526 (-0.14)	2.299 (6.79)
TPSSh	ZORA-TZVPP/TZVP	1.524 (-0.23)	2.196 (1.99)
TPSSh	DKH-TZVP/TZVP	1.516 (-0.78)	2.193 (1.85)
TPSSh	DKH-TZVPP/TZVP	1.515 (-0.86)	2.189 (1.66)
TPSSh	Sappo-DZP/DZP	1.528 (0.03)	2.222 (3.20)
TPSSh	Sappo-TZP/DZP	1.526 (-0.13)	2.208 (2.57)
TPSSh	Sappo-QZP/DZP	1.523 (-0.31)	2.207 (2.50)
M06	def2-TZVP	1.526 (-0.11)	2.231 (3.60)
M06	def2-QZVP	1.533 (0.31)	2.225 (3.33)
M06	LANL2DZ	1.552 (1.59)	2.334 (8.40)
M06	LANL2TZ/def2-TZVP	1.548 (1.31)	-
M06	ZORA-TZVP/TZVP	1.546 (1.19)	2.232 (3.65)
M06	ZORA-TZVPP/TZVP	1.545 (1.09)	2.228 (3.50)
M06	DKH-TZVP/TZVP	1.537 (0.56)	2.224 (3.28)
M06	DKH-TZVPP/TZVP	1.535 (0.46)	2.222 (3.23)
M06	Sappo-DZP/DZP	1.548 (1.33)	2.249 (4.47)
M06	Sappo-TZP/DZP	1.548 (1.28)	2.240 (4.03)
M06	Sappo-QZP/DZP	1.544 (1.05)	2.237 (3.91)

to continued ...

Method	Basis set	PtH	PtCl
M06-2X	def2-TZVP	1.507 (-1.38)	2.215 (2.86)
M06-2X	def2-QZVP	1.503 (-1.66)	2.208 (2.56)
M06-2X	LANL2DZ	1.524 (-0.27)	2.334 (8.40)
M06-2X	LANL2TZ/def2-TZVP	1.515 (-0.86)	2.219 (3.06)
M06-2X	ZORA-TZVP/TZVP	1.502 (-1.73)	2.209 (2.60)
M06-2X	ZORA-TZVPP/TZVP	1.500 (-1.86)	2.204 (2.38)
M06-2X	DKH-TZVP/TZVP	1.492 (-2.34)	2.204 (2.35)
M06-2X	DKH-TZVPP/TZVP	1.490 (-2.46)	2.198 (2.10)
M06-2X	Sappo-DZP/DZP	1.507 (-1.39)	2.242 (4.16)
M06-2X	Sappo-TZP/DZP	1.503 (-1.65)	2.220 (3.13)
M06-2X	Sappo-QZP/DZP	1.499 (-1.90)	2.223 (3.27)
LC-BLYP	def2-TZVP	1.528 (0.00)	2.196 (1.99)
LC-BLYP	def2-QZVP	1.525 (-0.20)	2.191 (1.79)
LC-BLYP	LANL2DZ	1.539 (0.74)	2.306 (7.11)
LC-BLYP	LANL2TZ/def2-TZVP	1.528 (-0.03)	2.141 (-0.56)
LC-BLYP	ZORA-TZVP/TZVP	1.523 (-0.30)	2.187 (1.59)
LC-BLYP	ZORA-TZVPP/TZVP	1.522 (-0.39)	2.183 (1.41)
LC-BLYP	DKH-TZVP/TZVP	1.514 (-0.92)	2.181 (1.29)
LC-BLYP	DKH-TZVPP/TZVP	1.513 (-1.01)	2.176 (1.08)
LC-BLYP	Sappo-DZP/DZP	1.525 (-0.17)	2.210 (2.63)
LC-BLYP	Sappo-TZP/DZP	1.523 (-0.36)	2.195 (1.97)
LC-BLYP	Sappo-QZP/DZP	1.520 (-0.55)	2.194 (1.92)
CAM-B3LYP	def2-TZVP	1.529 (0.09)	2.217 (2.98)
CAM-B3LYP	def2-QZVP	1.526 (-0.13)	2.212 (2.76)
CAM-B3LYP	LANL2DZ	1.540 (0.80)	2.327 (8.09)

to continued ...

Method	Basis set	PtH	PtCl
CAM-B3LYP	LANL2TZ/def2-TZVP	1.528 (0.02)	2.327 (8.09)
CAM-B3LYP	ZORA-TZVP/TZVP	1.525 (-0.22)	2.208 (2.56)
CAM-B3LYP	ZORA-TZVPP/TZVP	1.523 (-0.31)	2.204 (2.39)
CAM-B3LYP	DKH-TZVP/TZVP	1.515 (-0.84)	2.202 (2.30)
CAM-B3LYP	DKH-TZVPP/TZVP	1.514 (-0.92)	2.198 (2.10)
CAM-B3LYP	Sappo-DZP/DZP	1.527 (-0.06)	2.231 (3.64)
CAM-B3LYP	Sappo-TZP/DZP	1.524 (-0.23)	2.218 (3.00)
CAM-B3LYP	Sappo-QZP/DZP	1.521 (-0.43)	2.217 (2.95)
ωB97X-D3BJ	def2-TZVP	1.536 (0.53)	2.173 (0.92)
ωB97X-D3BJ	def2-QZVP	1.534 (0.39)	2.164 (0.52)
ωB97X-D3BJ	LANL2DZ	1.541 (0.86)	2.288 (6.29)
ωB97X-D3BJ	LANL2TZ/def2-TZVP	1.525 (-0.21)	2.208 (2.54)
ωB97X-D3BJ	ZORA-TZVP/TZVP	1.532 (0.23)	2.164 (0.50)
ωB97X-D3BJ	ZORA-TZVPP/TZVP	1.531 (0.19)	2.158 (0.21)
ωB97X-D3BJ	DKH-TZVP/TZVP	1.522 (-0.38)	2.157 (0.17)
ωB97X-D3BJ	DKH-TZVPP/TZVP	1.521 (-0.43)	2.149 (-0.18)
ωB97X-D3BJ	Sappo-DZP/DZP	1.532 (0.25)	2.187 (1.59)
ωB97X-D3BJ	Sappo-TZP/DZP	1.530 (0.11)	2.173 (0.91)
ωB97X-D3BJ	Sappo-QZP/DZP	1.527 (-0.03)	2.170 (0.77)
B97-3c	def2-TZVP	1.509 (-1.25)	2.136 (-0.79)
B97-3c	def2-QZVP	1.505 (-1.52)	2.125 (-1.32)
B97-3c	LANL2DZ	1.520 (-0.54)	2.284 (6.06)
B97-3c	LANL2TZ/def2-TZVP	1.510 (-1.20)	-
B97-3c	ZORA-TZVP/TZVP	1.505 (-1.53)	2.129 (-1.11)
B97-3c	ZORA-TZVPP/TZVP	1.503 (-1.63)	2.117 (-1.66)

to continued ...

Method	Basis set	PtH	PtCl
B97-3c	DKH-TZVP/TZVP	1.494 (-2.22)	-
B97-3c	DKH-TZVPP/TZVP	1.493 (-2.32)	2.105 (-2.22)
B97-3c	Sappo-DZP/DZP	1.240 (-18.86)	2.091 (-2.89)
B97-3c	Sappo-TZP/DZP	1.405 (-8.08)	-
B97-3c	Sappo-QZP/DZP	1.401 (-8.28)	2.147 (-0.30)
B2PLYP	def2-TZVP	1.521 (-0.44)	2.162 (0.41)
B2PLYP	def2-QZVP	1.513 (-0.96)	2.147 (-0.29)
B2PLYP	LANL2DZ	1.548 (1.29)	2.317 (7.62)
B2PLYP	LANL2TZ/def2-TZVP	1.521 (-0.45)	2.212 (2.72)
B2PLYP	ZORA-TZVP/TZVP	1.516 (-0.76)	2.149 (-0.20)
B2PLYP	ZORA-TZVPP/TZVP	1.515 (-0.88)	2.141 (-0.54)
B2PLYP	DKH-TZVP/TZVP	1.507 (-1.36)	2.141 (-0.57)
B2PLYP	DKH-TZVPP/TZVP	1.505 (-1.48)	2.132 (-0.97)
B2PLYP	Sappo-DZP/DZP	1.515 (-0.84)	2.177 (1.09)
B2PLYP	Sappo-TZP/DZP	1.512 (-1.05)	2.157 (0.21)
B2PLYP	Sappo-QZP/DZP	1.511 (-1.10)	2.150 (-0.13)
mPW2PLYP	def2-TZVP	1.522 (-0.41)	2.166 (0.62)
mPW2PLYP	def2-QZVP	1.514 (-0.90)	2.152 (-0.06)
mPW2PLYP	LANL2DZ	1.547 (1.24)	2.317 (7.62)
mPW2PLYP	LANL2TZ/def2-TZVP	1.521 (-0.47)	2.211 (2.71)
mPW2PLYP	ZORA-TZVP/TZVP	1.517 (-0.75)	2.153 (-0.02)
mPW2PLYP	ZORA-TZVPP/TZVP	1.515 (-0.87)	2.145 (-0.35)
mPW2PLYP	DKH-TZVP/TZVP	1.507 (-1.35)	2.145 (-0.37)
mPW2PLYP	DKH-TZVPP/TZVP	1.506 (-1.47)	2.136 (-0.77)
mPW2PLYP	Sappo-DZP/DZP	1.516 (-0.81)	2.180 (1.25)

to continued ...

Method	Basis set	PtH	PtCl
mPW2PLYP	Sappo-TZP/DZP	1.515 (-0.88)	2.161 (0.38)
mPW2PLYP	Sappo-QZP/DZP	1.512 (-1.08)	2.154 (0.06)
PBE0-DH	def2-TZVP	-	2.195 (1.96)
PBE0-DH	def2-QZVP	1.512 (-1.05)	2.188 (1.61)
PBE0-DH	LANL2DZ	1.536 (0.54)	2.326 (8.02)
PBE0-DH	LANL2TZ/def2-TZVP	1.518 (-0.67)	2.193 (1.88)
PBE0-DH	ZORA-TZVP/TZVP	1.514 (-0.95)	2.144 (-0.42)
PBE0-DH	ZORA-TZVPP/TZVP	1.512 (-1.06)	2.137 (-0.73)
PBE0-DH	DKH-TZVP/TZVP	1.504 (-1.55)	2.181 (1.32)
PBE0-DH	DKH-TZVPP/TZVP	1.503 (-1.67)	2.176 (1.08)
PBE0-DH	Sappo-DZP/DZP	1.513 (-0.95)	2.213 (2.81)
PBE0-DH	Sappo-TZP/DZP	1.512 (-1.05)	2.197 (2.06)
PBE0-DH	Sappo-QZP/DZP	1.509 (-1.22)	2.195 (1.93)
PBE-QIDH	def2-TZVP	-	2.146 (-0.31)
PBE-QIDH	def2-QZVP	1.501 (-1.74)	2.131 (-1.04)
PBE-QIDH	LANL2DZ	1.540 (0.79)	2.309 (7.26)
PBE-QIDH	LANL2TZ/def2-TZVP	1.512 (-1.07)	2.189 (1.67)
PBE-QIDH	ZORA-TZVP/TZVP	1.506 (-1.46)	2.136 (-0.80)
PBE-QIDH	ZORA-TZVPP/TZVP	1.504 (-1.59)	2.128 (-1.16)
PBE-QIDH	DKH-TZVP/TZVP	1.497 (-2.05)	2.127 (-1.19)
PBE-QIDH	DKH-TZVPP/TZVP	1.495 (-2.19)	2.118 (-1.62)
PBE-QIDH	Sappo-DZP/DZP	1.504 (-1.60)	2.165 (0.56)
PBE-QIDH	Sappo-TZP/DZP	1.503 (-1.63)	2.145 (-0.36)
PBE-QIDH	Sappo-QZP/DZP	1.500 (-1.82)	2.136 (-0.77)
DSD-BLYP	def2-TZVP	1.507 (-1.34)	2.146 (-0.35)

to continued ...

Method	Basis set	PtH	PtCl
DSD-BLYP	def2-QZVP	1.496 (-2.11)	2.126 (-1.24)
DSD-BLYP	LANL2DZ	1.547 (1.24)	2.327 (8.07)
DSD-BLYP	LANL2TZ/def2-TZVP	1.510 (-1.15)	2.196 (2.00)
DSD-BLYP	ZORA-TZVP/TZVP	1.502 (-1.71)	2.132 (-0.99)
DSD-BLYP	ZORA-TZVPP/TZVP	1.500 (-1.86)	2.123 (-1.39)
DSD-BLYP	DKH-TZVP/TZVP	1.493 (-2.30)	2.123 (-1.39)
DSD-BLYP	DKH-TZVPP/TZVP	1.491 (-2.45)	2.113 (-1.86)
DSD-BLYP	Sappo-DZP/DZP	1.499 (-1.93)	2.163 (0.46)
DSD-BLYP	Sappo-TZP/DZP	1.498 (-1.95)	2.141 (-0.58)
DSD-BLYP	Sappo-QZP/DZP	1.495 (-2.15)	2.130 (-1.08)
RI-SCS-MP2	def2-TZVP	1.494 (-2.20)	2.155 (0.09)
RI-SCS-MP2	def2-QZVP	-	-
RI-SCS-MP2	LANL2DZ	1.549 (1.40)	2.351 (9.21)
RI-SCS-MP2	LANL2TZ/def2-TZVP	1.501 (-1.80)	2.194 (1.90)
RI-SCS-MP2	ZORA-TZVP/TZVP	1.489 (-2.53)	2.141 (-0.56)
RI-SCS-MP2	ZORA-TZVPP/TZVP	1.487 (-2.68)	2.132 (-0.97)
RI-SCS-MP2	DKH-TZVP/TZVP	1.481 (-3.11)	2.133 (-0.93)
RI-SCS-MP2	DKH-TZVPP/TZVP	1.478 (-3.26)	2.123 (-1.41)
RI-SCS-MP2	Sappo-DZP/DZP	1.484 (-2.88)	2.176 (1.05)
RI-SCS-MP2	Sappo-TZP/DZP	1.485 (-2.82)	2.147 (-0.27)
RI-SCS-MP2	Sappo-QZP/DZP	1.481 (-3.05)	2.133 (-0.95)
HF	def2-TZVP	1.559 (2.05)	2.288 (6.29)
HF	def2-QZVP	1.558 (1.98)	2.282 (5.99)
HF	LANL2DZ	1.573 (2.97)	2.386 (10.84)
HF	LANL2TZ/def2-TZVP	1.539 (0.69)	2.263 (5.12)

to continued ...

Method	Basis set	PtH	PtCl
HF	ZORA-TZVP/TZVP	1.555 (1.78)	2.279 (5.87)
HF	ZORA-TZVPP/TZVP	1.554 (1.70)	2.274 (5.60)
HF	DKH-TZVP/TZVP	1.546 (1.19)	2.277 (5.75)
HF	DKH-TZVPP/TZVP	1.545 (1.11)	2.271 (5.47)
HF	Sappo-DZP/DZP	1.557 (1.88)	2.306 (7.10)
HF	Sappo-TZP/DZP	1.558 (1.97)	2.293 (6.50)
HF	Sappo-QZP/DZP	1.555 (1.80)	2.289 (6.34)
BP86	def2-TZVP	1.529 (0.08) MAPE 0.084%	-
PBE	def2-TZVP	1.528 (0.02) MAPE 0.021%	-
B3P86	def2-TZVP	1.525 (-0.23) MAPE 0.228%	-
B3PW91	def2-TZVP	1.524 (-0.28) MAPE 0.276%	-
BP86	ZORA-TZVP/TZVP	-	2.144 (-0.42) MAPE 0.42%
PBE	ZORA-TZVP/TZVP	-	2.148 (-0.24) MAPE 0.24%
B3P86	ZORA-TZVP/TZVP	-	2.147 (-0.29) MAPE 0.29%
B3PW91	ZORA-TZVP/TZVP	-	2.197 (2.02) MAPE 2.02%
HF-by-MMFF94	LANL2DZ/6-31G(d)	1.574 (2.99) MAPE 2.992%	2.320 (7.74) MAPE 7.743%

end.

$[PtCl_4]^{2-}$ and $[Pt(NH_3)_4]^{2+}$

Table S2: Calculated Bond Lengths (in Å) for Pt-Cl and Pt-N in $[PtCl_4]^{2-}$ and $[Pt(NH_3)_4]^{2+}$ complexes, with Relative Deviation (RD in %) in parentheses. All calculated bond angles Cl-Pt-Cl and N-Pt-N were ≈ 90 °. The level of theory used in the standard MMFF94 force field is included (HF-by-MMFF94). Abbreviated names for the basis sets are used here, with their full names listed in Table 1.

Method	Basis set	$[PtCl_4]^{2-}$	$[Pt(NH_3)_4]^{2+}$
Experimental	-	2.300[3]	2.038[4]
M06L	def2-TZVP	2.380 (3.48)	2.101 (3.09)
M06L	def2-QZVP	2.371 (3.09)	-
M06L	LANL2DZ	2.479 (7.78)	-
M06L	LANL2TZ/def2-TZVP	2.377 (3.35)	2.098 (2.94)
M06L	ZORA-TZVP/TZVP	2.372 (3.13)	2.094 (2.75)
M06L	ZORA-TZVPP/TZVP	2.371 (3.09)	2.093 (2.70)
M06L	DKH-TZVP/TZVP	2.369 (3.00)	-
M06L	DKH-TZVPP/TZVP	2.368 (2.96)	-
M06L	Sappo-DZP/DZP	2.392 (4.00)	-
M06L	Sappo-TZP/DZP	2.383 (3.61)	-
M06L	Sappo-QZP/DZP	2.384 (3.65)	-
B3LYP	def2-TZVP	2.385 (3.70)	2.097 (2.89)
B3LYP	def2-QZVP	2.379 (3.43)	2.095 (2.80)
B3LYP	LANL2DZ	2.472 (7.48)	2.109 (3.48)
B3LYP	LANL2TZ/def2-TZVP	2.383 (3.61)	2.096 (2.85)
B3LYP	ZORA-TZVP/TZVP	2.379 (3.43)	2.090 (2.55)
B3LYP	ZORA-TZVPP/TZVP	2.378 (3.39)	2.089 (2.50)
B3LYP	DKH-TZVP/TZVP	2.376 (3.30)	-
B3LYP	DKH-TZVPP/TZVP	2.375 (3.26)	-
B3LYP	Sappo-DZP/DZP	2.394 (4.09)	2.085 (2.31)

to continued ...

Method	Basis set	$[PtCl_4]^{2-}$	$[Pt(NH_3)_4]^{2+}$
B3LYP	Sappo-TZP/DZP	2.386 (3.74)	2.080 (2.06)
B3LYP	Sappo-QZP/DZP	2.386 (3.74)	2.076 (1.86)
mPW1PW	def2-TZVP	2.480 (7.83)	2.072 (1.67)
mPW1PW	def2-QZVP	2.342 (1.83)	2.071 (1.62)
mPW1PW	LANL2DZ	2.439 (6.04)	2.087 (2.40)
mPW1PW	LANL2TZ/def2-TZVP	2.347 (2.04)	2.071 (1.62)
mPW1PW	ZORA-TZVP/TZVP	2.343 (1.87)	2.066 (1.37)
mPW1PW	ZORA-TZVPP/TZVP	2.341 (1.78)	2.065 (1.32)
mPW1PW	DKH-TZVP/TZVP	2.339 (1.70)	-
mPW1PW	DKH-TZVPP/TZVP	2.338 (1.65)	-
mPW1PW	Sappo-DZP/DZP	2.359 (2.57)	2.062 (1.18)
mPW1PW	Sappo-TZP/DZP	2.350 (2.17)	2.057 (0.93)
mPW1PW	Sappo-QZP/DZP	2.350 (2.17)	2.053 (0.74)
PBE0	def2-TZVP	2.344 (1.91)	2.069 (1.52)
PBE0	def2-QZVP	2.338 (1.65)	2.068 (1.47)
PBE0	LANL2DZ	2.437 (5.96)	2.084 (2.26)
PBE0	LANL2TZ/def2-TZVP	2.343 (1.87)	2.068 (1.47)
PBE0	ZORA-TZVP/TZVP	2.339 (1.70)	2.063 (1.23)
PBE0	ZORA-TZVPP/TZVP	2.338 (1.65)	2.062 (1.18)
PBE0	DKH-TZVP/TZVP	2.336 (1.57)	-
PBE0	DKH-TZVPP/TZVP	2.334 (1.48)	-
PBE0	Sappo-DZP/DZP	2.355 (2.39)	2.060 (1.08)
PBE0	Sappo-TZP/DZP	2.346 (2.00)	2.054 (0.79)
PBE0	Sappo-QZP/DZP	2.346 (2.00)	2.051 (0.64)

to continued ...

Method	Basis set	$[PtCl_4]^{2-}$	$[Pt(NH_3)_4]^{2+}$
TPSSh	def2-TZVP	2.359 (2.57)	2.084 (2.26)
TPSSh	def2-QZVP	2.352 (2.26)	2.083 (2.21)
TPSSh	LANL2DZ	2.451 (6.57)	2.098 (2.94)
TPSSh	LANL2TZ/def2-TZVP	2.359 (2.57)	2.083 (2.21)
TPSSh	ZORA-TZVP/TZVP	2.353 (2.30)	2.078 (1.96)
TPSSh	ZORA-TZVPP/TZVP	2.352 (2.26)	2.077 (1.91)
TPSSh	DKH-TZVP/TZVP	2.350 (2.17)	-
TPSSh	DKH-TZVPP/TZVP	2.348 (2.09)	-
TPSSh	Sappo-DZP/DZP	2.368 (2.96)	2.073 (1.72)
TPSSh	Sappo-TZP/DZP	2.360 (2.61)	2.068 (1.47)
TPSSh	Sappo-QZP/DZP	2.360 (2.61)	2.065 (1.32)
M06	def2-TZVP	2.377 (3.35)	2.096 (2.85)
M06	def2-QZVP	2.371 (3.09)	-
M06	LANL2DZ	2.459 (6.91)	2.107* (3.39)
M06	LANL2TZ/def2-TZVP	2.377 (3.35)	2.096 (2.85)
M06	ZORA-TZVP/TZVP	2.376 (3.30)	2.092 (2.65)
M06	ZORA-TZVPP/TZVP	2.374 (3.22)	2.091 (2.60)
M06	DKH-TZVP/TZVP	2.373 (3.17)	-
M06	DKH-TZVPP/TZVP	2.371 (3.09)	-
M06	Sappo-DZP/DZP	2.389 (3.87)	2.085 (2.31)
M06	Sappo-TZP/DZP	2.382 (3.57)	2.082 (2.16)
M06	Sappo-QZP/DZP	2.382 (3.57)	2.079 (2.01)
M06-2X	def2-TZVP	2.377 (3.35)	2.089 (2.50)
M06-2X	def2-QZVP	2.377 (3.35)	2.088 (2.45)

to continued ...

Method	Basis set	$[PtCl_4]^{2-}$	$[Pt(NH_3)_4]^{2+}$
M06-2X	LANL2DZ	2.464 (7.13)	2.106 (3.34)
M06-2X	LANL2TZ/def2-TZVP	2.374 (3.22)	2.085 (2.31)
M06-2X	ZORA-TZVP/TZVP	2.374 (3.22)	2.082 (2.16)
M06-2X	ZORA-TZVPP/TZVP	2.373 (3.17)	2.081* (2.11)
M06-2X	DKH-TZVP/TZVP	2.371 (3.09)	-
M06-2X	DKH-TZVPP/TZVP	2.370 (3.04)	-
M06-2X	Sappo-DZP/DZP	2.393 (4.04)	2.080 (2.06)
M06-2X	Sappo-TZP/DZP	2.384 (3.65)	2.072 (1.67)
M06-2X	Sappo-QZP/DZP	2.387 (3.78)	-
LC-BLYP	def2-TZVP	2.333 (1.43)	2.064 (1.28)
LC-BLYP	def2-QZVP	2.329 (1.26)	2.062 (1.18)
LC-BLYP	LANL2DZ	2.419 (5.17)	2.075 (1.82)
LC-BLYP	LANL2TZ/def2-TZVP	2.332 (1.39)	2.063 (1.23)
LC-BLYP	ZORA-TZVP/TZVP	2.328 (1.22)	2.057 (0.93)
LC-BLYP	ZORA-TZVPP/TZVP	2.326 (1.13)	2.057 (0.93)
LC-BLYP	DKH-TZVP/TZVP	2.324 (1.04)	-
LC-BLYP	DKH-TZVPP/TZVP	2.323 (1.00)	-
LC-BLYP	Sappo-DZP/DZP	2.341 (1.78)	2.050 (0.59)
LC-BLYP	Sappo-TZP/DZP	2.332 (1.39)	2.046 (0.39)
LC-BLYP	Sappo-QZP/DZP	2.332 (1.39)	2.043 (0.25)
CAM-B3LYP	def2-TZVP	2.361 (2.65)	2.081 (2.11)
CAM-B3LYP	def2-QZVP	2.356 (2.43)	2.080 (2.06)
CAM-B3LYP	LANL2DZ	2.445 (6.30)	2.093 (2.70)
CAM-B3LYP	LANL2TZ/def2-TZVP	2.359 (2.57)	2.080 (2.06)

to continued ...

Method	Basis set	$[PtCl_4]^{2-}$	$[Pt(NH_3)_4]^{2+}$
CAM-B3LYP	ZORA-TZVP/TZVP	2.355 (2.39)	2.075 (1.82)
CAM-B3LYP	ZORA-TZVPP/TZVP	2.354 (2.35)	2.074 (1.77)
CAM-B3LYP	DKH-TZVP/TZVP	2.352 (2.26)	-
CAM-B3LYP	DKH-TZVPP/TZVP	2.351 (2.22)	-
CAM-B3LYP	Sappo-DZP/DZP	2.369 (3.00)	2.069 (1.52)
CAM-B3LYP	Sappo-TZP/DZP	2.361 (2.65)	2.064 (1.28)
CAM-B3LYP	Sappo-QZP/DZP	2.361 (2.65)	2.061 (1.13)
ωB97X-D3BJ	def2-TZVP	2.342 (1.83)	2.075 (1.82)
ωB97X-D3BJ	def2-QZVP	2.337 (1.61)	2.074 (1.77)
ωB97X-D3BJ	LANL2DZ	2.424 (5.39)	2.089 (2.50)
ωB97X-D3BJ	LANL2TZ/def2-TZVP	2.340 (1.74)	2.074 (1.77)
ωB97X-D3BJ	ZORA-TZVP/TZVP	2.337 (1.61)	2.070 (1.57)
ωB97X-D3BJ	ZORA-TZVPP/TZVP	2.336 (1.57)	2.069 (1.52)
ωB97X-D3BJ	DKH-TZVP/TZVP	2.334 (1.48)	-
ωB97X-D3BJ	DKH-TZVPP/TZVP	2.333 (1.43)	-
ωB97X-D3BJ	Sappo-DZP/DZP	2.353 (2.30)	2.066 (1.37)
ωB97X-D3BJ	Sappo-TZP/DZP	2.345 (1.96)	-
ωB97X-D3BJ	Sappo-QZP/DZP	2.345 (1.96)	2.058 (0.98)
B97-3c	def2-TZVP	2.353 (2.30)	2.084 (2.26)
B97-3c	def2-QZVP	2.345 (1.96)	2.083 (2.21)
B97-3c	LANL2DZ	2.451 (6.57)	2.098 (2.94)
B97-3c	LANL2TZ/def2-TZVP	2.352 (2.26)	2.083 (2.21)
B97-3c	ZORA-TZVP/TZVP	2.345 (1.96)	2.078 (1.96)
B97-3c	ZORA-TZVPP/TZVP	2.343 (1.87)	2.077 (1.91)

to continued ...

Method	Basis set	$[PtCl_4]^{2-}$	$[Pt(NH_3)_4]^{2+}$
B97-3c	DKH-TZVP/TZVP	2.341 (1.78)	-
B97-3c	DKH-TZVPP/TZVP	2.339 (1.70)	-
B97-3c	Sappo-DZP/DZP	-	2.073 (1.72)
B97-3c	Sappo-TZP/DZP	-	2.068 (1.47)
B97-3c	Sappo-QZP/DZP	-	2.065 (1.32)
B2PLYP	def2-TZVP	2.358 (2.52)	2.077 (1.91)
B2PLYP	def2-QZVP	2.350 (2.17)	-
B2PLYP	LANL2DZ	2.469 (7.35)	2.112 (3.63)
B2PLYP	LANL2TZ/def2-TZVP	2.363 (2.74)	2.078 (1.96)
B2PLYP	ZORA-TZVP/TZVP	2.353 (2.30)	2.071 (1.62)
B2PLYP	ZORA-TZVPP/TZVP	2.350 (2.17)	2.069 (1.52)
B2PLYP	DKH-TZVP/TZVP	2.350 (2.17)	-
B2PLYP	DKH-TZVPP/TZVP	2.346 (2.00)	-
B2PLYP	Sappo-DZP/DZP	2.373 (3.17)	-
B2PLYP	Sappo-TZP/DZP	2.360 (2.61)	2.062 (1.18)
B2PLYP	Sappo-QZP/DZP	2.356 (2.43)	2.057 (0.93)
mPW2PLYP	def2-TZVP	2.356 (2.43)	2.075 (1.82)
mPW2PLYP	def2-QZVP	2.349 (2.13)	-
mPW2PLYP	LANL2DZ	2.464 (7.13)	2.109 (3.48)
mPW2PLYP	LANL2TZ/def2-TZVP	2.360 (2.61)	2.076 (1.86)
mPW2PLYP	ZORA-TZVP/TZVP	2.351 (2.22)	2.070 (1.57)
mPW2PLYP	ZORA-TZVPP/TZVP	2.348 (2.09)	2.068 (1.47)
mPW2PLYP	DKH-TZVP/TZVP	2.348 (2.09)	-
mPW2PLYP	DKH-TZVPP/TZVP	2.345 (1.96)	-

to continued ...

Method	Basis set	$[PtCl_4]^{2-}$	$[Pt(NH_3)_4]^{2+}$
mPW2PLYP	Sappo-DZP/DZP	2.371 (3.09)	-
mPW2PLYP	Sappo-TZP/DZP	2.358 (2.52)	2.060 (1.08)
mPW2PLYP	Sappo-QZP/DZP	2.355 (2.39)	-
PBE0-DH	def2-TZVP	2.330 (1.30)	2.057 (0.93)
PBE0-DH	def2-QZVP	2.324 (1.04)	-
PBE0-DH	LANL2DZ	2.432 (5.74)	-
PBE0-DH	LANL2TZ/def2-TZVP	2.332 (1.39)	2.057 (0.93)
PBE0-DH	ZORA-TZVP/TZVP	2.326 (1.13)	2.052 (0.69)
PBE0-DH	ZORA-TZVPP/TZVP	2.324 (1.04)	2.051 (0.64)
PBE0-DH	DKH-TZVP/TZVP	2.322 (0.96)	-
PBE0-DH	DKH-TZVPP/TZVP	2.320 (0.87)	-
PBE0-DH	Sappo-DZP/DZP	2.344 (1.91)	2.050 (0.59)
PBE0-DH	Sappo-TZP/DZP	2.333 (1.43)	2.044 (0.29)
PBE0-DH	Sappo-QZP/DZP	2.332 (1.39)	2.040 (0.10)
PBE-QIDH	def2-TZVP	2.320 (0.87)	2.049 (0.54)
PBE-QIDH	def2-QZVP	2.312 (0.52)	-
PBE-QIDH	LANL2DZ	2.437 (5.96)	2.090 (2.55)
PBE-QIDH	LANL2TZ/def2-TZVP	2.326 (1.13)	2.050 (0.59)
PBE-QIDH	ZORA-TZVP/TZVP	2.316 (0.70)	2.045 (0.34)
PBE-QIDH	ZORA-TZVPP/TZVP	2.312 (0.53)	-
PBE-QIDH	DKH-TZVP/TZVP	2.312 (0.53)	-
PBE-QIDH	DKH-TZVPP/TZVP	2.308 (0.35)	-
PBE-QIDH	Sappo-DZP/DZP	2.337 (1.61)	2.043 (0.25)
PBE-QIDH	Sappo-TZP/DZP	2.323 (1.00)	2.037 (-0.05)

to continued ...

Method	Basis set	$[PtCl_4]^{2-}$	$[Pt(NH_3)_4]^{2+}$
PBE-QIDH	Sappo-QZP/DZP	2.319 (0.83)	-
DSD-BLYP	def2-TZVP	2.330 (1.30)	2.056 (0.88)
DSD-BLYP	def2-QZVP	2.320 (0.87)	-
DSD-BLYP	LANL2DZ	2.460 (6.96)	2.090 (2.55)
DSD-BLYP	LANL2TZ/def2-TZVP	2.341 (1.78)	2.059 (1.03)
DSD-BLYP	ZORA-TZVP/TZVP	2.325 (1.09)	2.051 (0.64)
DSD-BLYP	ZORA-TZVPP/TZVP	2.320 (0.87)	2.048 (0.49)
DSD-BLYP	DKH-TZVP/TZVP	2.322 (0.96)	-
DSD-BLYP	DKH-TZVPP/TZVP	2.317 (0.74)	-
DSD-BLYP	Sappo-DZP/DZP	2.349 (2.13)	2.049 (0.54)
DSD-BLYP	Sappo-TZP/DZP	2.332 (1.39)	-
DSD-BLYP	Sappo-QZP/DZP	2.326 (1.13)	-
RI-SCS-MP2	def2-TZVP	2.329 (1.26)	2.053 (0.74)
RI-SCS-MP2	def2-QZVP	-	-
RI-SCS-MP2	LANL2DZ	2.471 (7.43)	2.125 (4.27)
RI-SCS-MP2	LANL2TZ/def2-TZVP	2.346 (2.00)	2.059 (1.03)
RI-SCS-MP2	ZORA-TZVP/TZVP	2.324 (1.04)	2.049 (0.54)
RI-SCS-MP2	ZORA-TZVPP/TZVP	2.318 (0.78)	2.046 (0.39)
RI-SCS-MP2	DKH-TZVP/TZVP	2.321 (0.91)	-
RI-SCS-MP2	DKH-TZVPP/TZVP	2.314 (0.61)	-
RI-SCS-MP2	Sappo-DZP/DZP	2.351 (2.22)	-
RI-SCS-MP2	Sappo-TZP/DZP	2.330 (1.30)	-
RI-SCS-MP2	Sappo-QZP/DZP	2.320 (0.87)	-
HF	def2-TZVP	2.406 (4.61)	2.106 (3.34)

to continued ...

Method	Basis set	$[PtCl_4]^{2-}$	$[Pt(NH_3)_4]^{2+}$
HF	def2-QZVP	2.406 (4.61)	2.105 (3.29)
HF	LANL2DZ	2.482 (7.91)	2.122 (4.12)
HF	LANL2TZ/def2-TZVP	2.403 (4.48)	2.100 (3.04)
HF	ZORA-TZVP/TZVP	2.404 (4.52)	2.100 (3.04)
HF	ZORA-TZVPP/TZVP	2.403 (4.48)	2.100 (3.04)
HF	DKH-TZVP/TZVP	2.402 (4.43)	1.901 (-6.72)
HF	DKH-TZVPP/TZVP	2.401 (4.39)	-
HF	Sappo-DZP/DZP	2.419 (5.17)	2.102 (3.14)
HF	Sappo-TZP/DZP	2.413 (4.91)	-
HF	Sappo-QZP/DZP	2.412 (4.87)	2.093 (2.70)
BP86	DKH-TZVPP/TZVP	2.359 (2.55) MAPE 2.554%	-
PBE	DKH-TZVPP/TZVP	2.353 (2.31) MAPE 2.308%	-
B3P86	DKH-TZVPP/TZVP	2.343 (1.87) MAPE 1.873%	-
B3PW91	DKH-TZVPP/TZVP	2.344 (1.93) MAPE 1.926%	-
BP86	Sappo-TZP/DZP	-	2.071 (1.64) MAPE 1.642%
PBE	Sappo-TZP/DZP	-	2.068 (1.47) MAPE 1.468%
B3P86	Sappo-TZP/DZP	-	2.060 (1.08) MAPE 1.076%
B3PW91	Sappo-TZP/DZP	-	2.062 (1.17) MAPE 1.171%
HF-by-MMFF94	LANL2DZ/6-31G(d)	2.444 (6.26) MAPE 6.261%	2.118 (3.93) MAPE 3.925%

end.

Cisplatin $[Pt(NH_3)_2Cl_2]$

Table S3: Calculated Bond Lengths (in Å) Pt-Cl and Pt-N, and Bond Angles (°) Cl-Pt-Cl, Cl-Pt-N and N-Pt-N in cisplatin [$Pt(NH_3)_2Cl_2$], with Relative Deviation (RD in %) in parentheses. Atoms are numbered according to Figure 2. The level of theory used in the standard MMFF94 force field is included (HF-by-MMFF94). Abbreviated names for the basis sets are used here, with their full names listed in Table 1.

Method	Basis set	Pt-Cl	Pt-N	ClPtCl	ClPtN	NPtN
Experimental[5]	-	2.321	2.05	91.65	88.87	97.88
M06L	def2-TZVP	2.299 (-0.95)	2.119 (3.44)	95.91 (4.65)	82.35 (-7.34)	99.41 (1.57)
M06L	def2-QZVP	2.295 (-1.12)	2.116 (3.32)	95.88 (4.62)	82.47 (-7.20)	99.18 (1.33)
M06L	LANL2DZ	2.411 (3.88)	2.118 (3.42)	97.12 (5.97)	81.40 (-8.41)	100.10 (2.27)
M06L	LANL2TZ/def2-TZVP	2.299 (-0.95)	2.117 (3.37)	95.81 (4.54)	82.38 (-7.30)	99.44 (1.60)
M06L	ZORA-TZVP/TZVP	2.294 (-1.16)	2.107 (2.88)	95.98 (4.72)	82.57 (-7.09)	98.89 (1.04)
M06L	ZORA-TZVPP/TZVP	2.293 (-1.21)	2.106 (2.83)	95.99 (4.74)	82.55 (-7.11)	98.91 (1.06)
M06L	DKH-TZVP/TZVP	2.304 (-0.73)	2.006 (-2.05)	95.70 (4.42)	82.24 (-7.46)	99.82 (1.99)
M06L	DKH-TZVPP/TZVP	2.303 (-0.78)	2.000 (-2.34)	95.70 (4.42)	82.23 (-7.47)	99.85 (2.02)
M06L	Sappo-DZP/DZP	2.227 (-4.07)	2.099 (2.49)	95.70 (4.42)	83.05 (-6.54)	98.20 (0.33)
M06L	Sappo-TZP/DZP	2.303 (-0.78)	2.097 (2.39)	95.99 (4.74)	82.79 (-6.84)	98.44 (0.58)
M06L	Sappo-QZP/DZP	2.305 (-0.71)	2.093 (2.20)	96.02 (4.77)	82.72 (-6.91)	98.55 (0.69)
B3LYP	def2-TZVP	2.308 (-0.58)	2.112 (3.13)	95.57 (4.28)	83.02 (-6.58)	98.40 (0.54)
B3LYP	def2-QZVP	2.306 (-0.65)	2.108 (2.93)	95.67 (4.39)	83.06 (-6.53)	98.21 (0.34)

to continued ...

Method	Basis set	Pt-Cl	Pt-N	Cl-Pt-Cl	Cl-Pt-N	N-Pt-N
B3LYP	LANL2DZ	2.411 (3.88)	2.111 (3.08)	96.77 (5.59)	81.96 (-7.78)	99.33 (1.49)
B3LYP	LANL2TZ/def2-TZVP	2.308 (-0.56)	2.111 (3.08)	95.57 (4.28)	83.04 (-6.55)	98.34 (0.47)
B3LYP	ZORA-TZVP/TZVP	2.303 (-0.80)	2.103 (2.69)	95.51 (4.21)	83.11 (-6.48)	98.27 (0.40)
B3LYP	ZORA-TZVPP/TZVP	2.301 (-0.86)	2.102 (2.64)	95.53 (4.23)	83.09 (-6.50)	98.31 (0.44)
B3LYP	DKH-TZVP/TZVP	2.312 (-0.39)	2.004 (-2.15)	95.36 (4.05)	82.56 (-7.10)	99.53 (1.69)
B3LYP	DKH-TZVPP/TZVP	2.311 (-0.43)	1.997 (-2.49)	95.36 (4.05)	82.52 (-7.14)	99.61 (1.77)
B3LYP	Sappo-DZP/DZP	2.317 (-0.17)	2.093 (2.20)	96.20 (4.96)	82.85 (-6.77)	98.12 (0.25)
B3LYP	Sappo-TZP/DZP	2.311 (-0.43)	2.086 (1.86)	96.14 (4.90)	82.92 (-6.70)	98.04 (0.17)
B3LYP	Sappo-QZP/DZP	2.312 (-0.39)	2.082 (1.66)	96.14 (4.90)	82.88 (-6.74)	98.11 (0.24)
mPW1PW	def2-TZVP	2.281 (-1.74)	2.082 (1.66)	95.52 (4.22)	82.97 (-6.63)	98.54 (0.68)
mPW1PW	def2-QZVP	2.279 (-1.81)	2.078 (1.46)	95.73 (4.45)	82.91 (-6.70)	98.45 (0.59)
mPW1PW	LANL2DZ	2.386 (2.80)	2.085 (1.81)	96.27 (5.04)	82.27 (-7.43)	99.20 (1.35)
mPW1PW	LANL2TZ/def2-TZVP	2.282 (-1.68)	2.081 (1.61)	95.50 (4.20)	83.00 (-6.61)	98.52 (0.66)
mPW1PW	ZORA-TZVP/TZVP	2.276 (-1.94)	2.074 (1.27)	95.46 (4.16)	83.08 (-6.51)	98.38 (0.51)
mPW1PW	ZORA-TZVPP/TZVP	2.275 (-1.98)	2.073 (1.22)	95.80 (4.53)	83.06 (-6.53)	98.40 (0.54)
mPW1PW	DKH-TZVP/TZVP	2.287 (-1.46)	1.966 (-4.00)	95.19 (3.86)	82.36 (-7.32)	100.11 (2.28)
mPW1PW	DKH-TZVPP/TZVP	2.287 (-1.46)	1.956 (-4.49)	95.14 (3.81)	82.29 (-7.40)	100.30 (2.48)

to continued ...

Method	Basis set	Pt-Cl	Pt-N	Cl-Pt-Cl	Cl-Pt-N	N-Pt-N
mPW1PW	Sappo-DZP/DZP	2.291 (-1.29)	2.067 (0.93)	96.06 (4.81)	82.84 (-6.79)	98.28 (0.41)
mPW1PW	Sappo-TZP/DZP	2.285 (-1.55)	2.059 (0.54)	95.98 (4.72)	82.93 (-6.68)	98.16 (0.29)
mPW1PW	Sappo-QZP/DZP	2.286 (-1.51)	2.057 (0.44)	96.00 (4.75)	82.89 (-6.73)	98.22 (0.35)
PBE0	def2-TZVP	2.278 (-1.87)	2.079 (1.51)	95.55 (4.26)	82.92 (-6.69)	98.60 (0.74)
PBE0	def2-QZVP	2.276 (-1.94)	2.075 (1.32)	95.72 (4.44)	82.88 (-6.73)	98.51 (0.65)
PBE0	LANL2DZ	2.385 (2.74)	2.083 (1.71)	96.32 (5.10)	82.20 (-7.51)	99.29 (1.44)
PBE0	LANL2TZ/def2-TZVP	2.279 (-1.81)	2.077 (1.42)	95.52 (4.22)	82.94 (-6.67)	98.59 (0.73)
PBE0	ZORA-TZVP/TZVP	2.274 (-2.02)	2.071 (1.12)	95.48 (4.18)	83.04 (-6.56)	98.44 (0.58)
PBE0	ZORA-TZVPP/TZVP	2.272 (-2.11)	2.070 (1.07)	95.50 (4.20)	83.01 (-6.59)	98.48 (0.62)
PBE0	DKH-TZVP/TZVP	2.286 (-1.51)	1.959 (-4.35)	95.15 (3.82)	82.29 (-7.40)	100.30 (2.48)
PBE0	DKH-TZVPP/TZVP	2.286 (-1.53)	1.950 (-4.79)	95.12 (3.79)	82.23 (-7.47)	100.44 (2.62)
PBE0	Sappo-DZP/DZP	2.289 (-1.38)	2.064 (0.78)	96.09 (4.84)	82.81 (-6.82)	98.31 (0.44)
PBE0	Sappo-TZP/DZP	2.282 (-1.68)	2.056 (0.39)	96.01 (4.76)	82.90 (-6.72)	98.20 (0.33)
PBE0	Sappo-QZP/DZP	2.283 (-1.64)	2.054 (0.29)	96.04 (4.79)	82.86 (-6.76)	98.26 (0.39)
TPSSh	def2-TZVP	2.290 (-1.34)	2.093 (2.20)	95.79 (4.52)	82.57 (-7.09)	99.07 (1.22)
TPSSh	def2-QZVP	2.289 (-1.40)	2.090 (2.05)	95.92 (4.66)	82.54 (-7.12)	98.99 (1.14)
TPSSh	LANL2DZ	2.397 (3.27)	2.096 (2.34)	96.46 (5.25)	81.86 (-7.88)	99.82 (1.99)

to continued ...

Method	Basis set	Pt-Cl	Pt-N	Cl-Pt-Cl	Cl-Pt-N	N-Pt-N
TPSSh	LANL2TZ/def2-TZVP	2.292 (-1.25)	2.092 (2.15)	95.75 (4.47)	82.59 (-7.06)	99.08 (1.23)
TPSSh	ZORA-TZVP/TZVP	2.285 (-1.55)	2.085 (1.81)	95.70 (4.42)	82.67 (-6.97)	98.95 (1.10)
TPSSh	ZORA-TZVPP/TZVP	2.284 (-1.59)	2.084 (1.76)	95.72 (4.44)	82.65 (-6.99)	98.99 (1.14)
TPSSh	DKH-TZVP/TZVP	2.294 (-1.16)	1.984 (-3.13)	95.50 (4.20)	82.14 (-7.57)	100.24 (2.42)
TPSSh	DKH-TZVPP/TZVP	2.293 (-1.21)	1.977 (-3.49)	95.49 (4.19)	82.11 (-7.61)	100.31 (2.49)
TPSSh	Sappo-DZP/DZP	2.299 (-0.95)	2.077 (1.42)	96.33 (5.11)	82.46 (-7.21)	98.76 (0.90)
TPSSh	Sappo-TZP/DZP	2.293 (-1.21)	2.070 (1.05)	96.24 (5.01)	82.55 (-7.11)	98.67 (0.81)
TPSSh	Sappo-QZP/DZP	2.294 (-1.16)	2.066 (0.88)	96.24 (5.01)	82.52 (-7.15)	98.73 (0.87)
M06	def2-TZVP	2.301 (-0.86)	2.115 (3.27)	95.02 (3.68)	82.93 (-6.68)	99.13 (1.28)
M06	def2-QZVP	2.299 (-0.95)	2.111 (3.08)	95.22 (3.90)	82.93 (-6.68)	98.91 (1.06)
M06	LANL2DZ	2.400 (3.38)	2.113 (3.17)	96.12 (4.88)	82.19 (-7.51)	99.50 (1.66)
M06	LANL2TZ/def2-TZVP	2.303 (-0.80)	2.116 (3.32)	94.96 (3.61)	82.98 (-6.62)	99.07 (1.22)
M06	ZORA-TZVP/TZVP	2.301 (-0.86)	2.107 (2.88)	95.26 (3.94)	83.18 (-6.40)	98.39 (0.53)
M06	ZORA-TZVPP/TZVP	2.300 (-0.90)	2.106 (2.83)	95.27 (3.95)	83.15 (-6.43)	98.43 (0.57)
M06	DKH-TZVP/TZVP	2.309 (-0.52)	2.006 (-2.05)	95.13 (3.80)	82.51 (-7.16)	99.88 (2.05)
M06	DKH-TZVPP/TZVP	2.309 (-0.52)	1.999 (-2.39)	95.14 (3.81)	82.47 (-7.20)	99.95 (2.12)
M06	Sappo-DZP/DZP	2.314 (-0.32)	2.099 (2.49)	95.70 (4.42)	83.05 (-6.54)	98.20 (0.33)

to continued ...

Method	Basis set	Pt-Cl	Pt-N	Cl-Pt-Cl	Cl-Pt-N	N-Pt-N
M06	Sappo-TZP/DZP	2.309 (-0.52)	2.093 (2.20)	95.60 (4.31)	83.10 (-6.49)	98.20 (0.33)
M06	Sappo-QZP/DZP	2.310 (-0.50)	2.090 (2.05)	95.61 (4.32)	83.05 (-6.54)	98.29 (0.42)
M06-2X	def2-TZVP	2.289 (-1.38)	2.117 (3.37)	95.19 (3.86)	83.55 (-5.99)	97.72 (-0.16)
M06-2X	def2-QZVP	2.289 (-1.38)	2.111 (3.08)	95.64 (4.35)	83.30 (-6.27)	97.77 (-0.11)
M06-2X	LANL2DZ	2.403 (3.53)	2.108 (2.93)	97.06 (5.90)	82.11 (-7.60)	98.72 (0.86)
M06-2X	LANL2TZ/def2-TZVP	2.289 (-1.38)	2.109 (2.98)	95.12 (3.79)	83.59 (-5.94)	97.71 (-0.17)
M06-2X	ZORA-TZVP/TZVP	2.285 (-1.55)	2.106 (2.83)	95.15 (3.82)	83.63 (-5.90)	97.61 (-0.27)
M06-2X	ZORA-TZVPP/TZVP	2.283 (-1.64)	2.105 (2.78)	95.15 (3.82)	83.61 (-5.91)	97.64 (-0.24)
M06-2X	DKH-TZVP/TZVP	2.311 (-0.45)	1.957 (-4.44)	96.27 (5.04)	82.00 (-7.73)	99.73 (1.89)
M06-2X	DKH-TZVPP/TZVP	2.312 (-0.41)	1.945 (-5.03)	96.36 (5.14)	81.93 (-7.81)	99.78 (1.95)
M06-2X	Sappo-DZP/DZP	2.305 (-0.69)	2.097 (2.39)	95.92 (4.66)	83.29 (-6.28)	97.52 (-0.36)
M06-2X	Sappo-TZP/DZP	2.298 (-0.99)	2.086 (1.86)	95.81 (4.54)	83.40 (-6.16)	97.39 (-0.50)
M06-2X	Sappo-QZP/DZP	2.301 (-0.86)	2.084 (1.76)	95.89 (4.63)	83.33 (-6.23)	97.44 (-0.45)
LC-BLYP	def2-TZVP	2.273 (-2.07)	2.073 (1.22)	95.61 (4.32)	82.82 (-6.81)	98.75 (0.89)
LC-BLYP	def2-QZVP	2.273 (-2.07)	2.069 (1.03)	95.65 (4.36)	82.90 (-6.72)	98.57 (0.71)
LC-BLYP	LANL2DZ	2.273 (-2.07)	2.069 (1.03)	95.65 (4.36)	82.90 (-6.72)	98.57 (0.71)
LC-BLYP	LANL2TZ/def2-TZVP	2.274 (-2.02)	2.071 (1.12)	95.59 (4.30)	82.84 (-6.78)	98.73 (0.87)

to continued ...

Method	Basis set	Pt-Cl	Pt-N	Cl-Pt-Cl	Cl-Pt-N	N-Pt-N
LC-BLYP	ZORA-TZVP/TZVP	2.269 (-2.24)	2.064 (0.78)	95.47 (4.17)	82.98 (-6.62)	98.57 (0.71)
LC-BLYP	ZORA-TZVPP/TZVP	2.268 (-2.31)	2.063 (0.73)	95.49 (4.19)	82.96 (-6.65)	98.60 (0.74)
LC-BLYP	DKH-TZVP/TZVP	2.280 (-1.79)	1.955 (-4.54)	95.07 (3.73)	82.26 (-7.44)	100.46 (2.64)
LC-BLYP	DKH-TZVPP/TZVP	2.280 (-1.77)	1.945 (-5.03)	95.02 (3.68)	82.18 (-7.52)	100.65 (2.83)
LC-BLYP	Sappo-DZP/DZP	2.283 (-1.64)	2.052 (0.20)	96.36 (5.14)	82.56 (-7.10)	98.53 (0.67)
LC-BLYP	Sappo-TZP/DZP	2.277 (-1.90)	2.046 (-0.10)	96.27 (5.04)	82.64 (-7.01)	98.46 (0.60)
LC-BLYP	Sappo-QZP/DZP	2.278 (-1.87)	2.044 (-0.20)	96.27 (5.04)	82.60 (-7.05)	98.53 (0.67)
CAM-B3LYP	def2-TZVP	2.291 (-1.29)	2.094 (2.25)	95.51 (4.21)	83.15 (-6.43)	98.19 (0.32)
CAM-B3LYP	def2-QZVP	2.290 (-1.34)	2.090 (2.05)	95.64 (4.35)	83.17 (-6.41)	98.04 (0.17)
CAM-B3LYP	LANL2DZ	2.391 (3.02)	2.093 (2.20)	96.67 (5.48)	82.22 (-7.48)	98.89 (1.04)
CAM-B3LYP	LANL2TZ/def2-TZVP	2.291 (-1.29)	2.092 (2.15)	95.50 (4.20)	83.18 (-6.40)	98.15 (0.28)
CAM-B3LYP	ZORA-TZVP/TZVP	2.286 (-1.51)	2.085 (1.81)	95.44 (4.14)	83.27 (-6.30)	98.03 (0.16)
CAM-B3LYP	ZORA-TZVPP/TZVP	2.285 (-1.55)	2.084 (1.76)	95.46 (4.16)	83.24 (-6.33)	98.06 (0.19)
CAM-B3LYP	DKH-TZVP/TZVP	2.297 (-1.06)	1.981 (-3.27)	95.14 (3.81)	82.55 (-7.11)	99.79 (1.96)
CAM-B3LYP	DKH-TZVPP/TZVP	2.296 (-1.08)	1.974 (-3.61)	95.12 (3.79)	82.50 (-7.16)	99.90 (2.07)
CAM-B3LYP	Sappo-DZP/DZP	2.300 (-0.90)	2.076 (1.37)	96.14 (4.90)	82.96 (-6.65)	97.95 (0.08)
CAM-B3LYP	Sappo-TZP/DZP	2.295 (-1.12)	2.069 (1.03)	96.08 (4.83)	83.02 (-6.58)	97.87 (-0.01)

to continued ...

Method	Basis set	Pt-Cl	Pt-N	Cl-Pt-Cl	Cl-Pt-N	N-Pt-N
CAM-B3LYP	Sappo-QZP/DZP	2.296 (-1.08)	2.066 (0.88)	96.09 (4.84)	82.99 (-6.61)	97.93 (0.06)
ωB97X-D3BJ	def2-TZVP	2.277 (-1.90)	2.087 (1.90)	95.13 (3.80)	83.49 (-6.05)	97.90 (0.02)
ωB97X-D3BJ	def2-QZVP	2.276 (-1.94)	2.083 (1.71)	95.32 (4.00)	83.45 (-6.10)	97.79 (-0.09)
ωB97X-D3BJ	LANL2DZ	2.375 (2.33)	2.088 (1.95)	96.13 (4.89)	82.70 (-6.94)	98.47 (0.61)
ωB97X-D3BJ	LANL2TZ/def2-TZVP	2.277 (-1.90)	2.085 (1.81)	95.11 (3.78)	83.50 (-6.04)	97.89 (0.01)
ωB97X-D3BJ	ZORA-TZVP/TZVP	2.274 (-2.05)	2.080 (1.56)	95.10 (3.76)	83.59 (-5.94)	97.73 (-0.15)
ωB97X-D3BJ	ZORA-TZVPP/TZVP	2.272 (-2.11)	2.079 (1.51)	95.10 (3.76)	83.57 (-5.96)	97.77 (-0.11)
ωB97X-D3BJ	DKH-TZVP/TZVP	2.285 (-1.55)	1.972 (-3.71)	94.72 (3.35)	82.74 (-6.90)	99.84 (2.01)
ωB97X-D3BJ	DKH-TZVPP/TZVP	2.284 (-1.59)	1.964 (-4.10)	94.71 (3.34)	82.68 (-6.97)	99.97 (2.14)
ωB97X-D3BJ	Sappo-DZP/DZP	2.288 (-1.42)	2.073 (1.22)	95.69 (4.41)	83.33 (-6.23)	97.64 (-0.24)
ωB97X-D3BJ	Sappo-TZP/DZP	2.282 (-1.68)	2.065 (0.83)	95.62 (4.33)	83.41 (-6.14)	97.57 (-0.31)
ωB97X-D3BJ	Sappo-QZP/DZP	2.283 (-1.64)	2.062 (0.68)	95.63 (4.34)	83.38 (-6.18)	97.63 (-0.25)
B97-3c	def2-TZVP	2.280 (-1.77)	2.085 (1.81)	95.27 (3.95)	83.07 (-6.52)	98.59 (0.73)
B97-3c	def2-QZVP	2.277 (-1.90)	2.081 (1.61)	95.28 (3.96)	83.16 (-6.43)	98.41 (0.55)
B97-3c	LANL2DZ	2.392 (3.06)	2.087 (1.90)	96.26 (5.03)	82.05 (-7.67)	99.65 (1.81)
B97-3c	LANL2TZ/def2-TZVP	2.282 (-1.68)	2.084 (1.76)	95.25 (3.93)	83.09 (-6.50)	98.58 (0.72)
B97-3c	ZORA-TZVP/TZVP	2.273 (-2.09)	2.076 (1.37)	95.12 (3.79)	83.29 (-6.27)	98.30 (0.43)

to continued ...

Method	Basis set	Pt-Cl	Pt-N	Cl-Pt-Cl	Cl-Pt-N	N-Pt-N
B97-3c	ZORA-TZVPP/TZVP	2.272 (-2.11)	2.074 (1.27)	95.16 (3.83)	83.27 (-6.30)	98.32 (0.45)
B97-3c	DKH-TZVP/TZVP	2.289 (-1.38)	1.945 (-5.03)	94.58 (3.20)	82.44 (-7.24)	100.58 (2.76)
B97-3c	DKH-TZVPP/TZVP	2.289 (-1.38)	1.932 (-5.66)	94.54 (3.15)	82.36 (-7.33)	100.79 (2.98)
B97-3c	Sappo-DZP/DZP	2.177 (-6.20)	1.989 (-2.88)	100.21 (9.34)	81.94 (-7.80)	94.84 (-3.10)
B97-3c	Sappo-TZP/DZP	2.177 (-6.20)	1.896 (-7.42)	100.53 (9.69)	82.54 (-7.12)	94.39 (-3.56)
B97-3c	Sappo-QZP/DZP	2.207 (-4.91)	2.115 (3.27)	103.07 (12.46)	79.99 (-9.99)	92.43 (-5.56)
B2PLYP	def2-TZVP	-	-	-	-	
B2PLYP	def2-QZVP	2.284 (-1.59)	2.083 (1.71)	95.62 (4.33)	83.01 (-6.59)	98.36 (0.49)
B2PLYP	LANL2DZ	2.408 (3.75)	2.116 (3.30)	96.43 (5.22)	82.44 (-7.23)	98.70 (0.84)
B2PLYP	LANL2TZ/def2-TZVP	2.294 (-1.16)	2.089 (1.98)	95.42 (4.11)	83.07 (-6.53)	98.45 (0.59)
B2PLYP	ZORA-TZVP/TZVP	2.283 (-1.64)	2.083 (1.71)	95.21 (3.88)	83.26 (-6.31)	98.29 (0.42)
B2PLYP	ZORA-TZVPP/TZVP	2.280 (-1.77)	2.081 (1.61)	95.20 (3.87)	83.25 (-6.32)	98.30 (0.43)
B2PLYP	DKH-TZVP/TZVP	2.294 (-1.16)	1.980 (-3.34)	94.99 (3.64)	82.52 (-7.14)	99.99 (2.16)
B2PLYP	DKH-TZVPP/TZVP	2.292 (-1.27)	1.971 (-3.78)	94.95 (3.60)	82.49 (-7.18)	100.10 (2.27)
B2PLYP	Sappo-DZP/DZP	2.302 (-0.84)	2.076 (1.37)	95.85 (4.58)	83.13 (-6.45)	97.89 (0.01)
B2PLYP	Sappo-TZP/DZP	2.291 (-1.29)	2.068 (0.98)	95.66 (4.38)	83.25 (-6.32)	97.85 (-0.03)

to continued ...

Method	Basis set	Pt-Cl	Pt-N	Cl-Pt-Cl	Cl-Pt-N	N-Pt-N
B2PLYP	Sappo-QZP/DZP	2.289 (-1.38)	2.063 (0.73)	95.62 (4.33)	83.28 (-6.28)	97.83 (-0.05)
mPW2PLYP	def2-TZVP	2.286 (-1.51)	2.088 (1.93)	95.34 (4.03)	83.19 (-6.39)	98.27 (0.40)
mPW2PLYP	def2-QZVP	2.283 (-1.64)	2.081 (1.61)	95.66 (4.38)	83.05 (-6.54)	98.24 (0.37)
mPW2PLYP	LANL2DZ	2.404 (3.58)	2.112 (3.13)	96.45 (5.24)	82.49 (-7.17)	98.57 (0.71)
mPW2PLYP	LANL2TZ/def2-TZVP	2.292 (-1.25)	2.086 (1.86)	95.45 (4.15)	83.12 (-6.46)	98.31 (0.44)
mPW2PLYP	ZORA-TZVP/TZVP	2.281 (-1.72)	2.081 (1.61)	95.23 (3.91)	83.30 (-6.27)	98.17 (0.30)
mPW2PLYP	ZORA-TZVPP/TZVP	2.279 (-1.83)	2.079 (1.51)	95.23 (3.91)	83.30 (-6.27)	98.19 (0.32)
mPW2PLYP	DKH-TZVP/TZVP	2.293 (-1.23)	1.977 (-3.47)	95.01 (3.67)	82.53 (-7.13)	99.94 (2.11)
mPW2PLYP	DKH-TZVPP/TZVP	2.290 (-1.34)	1.968 (-3.91)	94.96 (3.61)	82.51 (-7.16)	100.04 (2.21)
mPW2PLYP	Sappo-DZP/DZP	2.300 (-0.90)	2.074 (1.27)	95.89 (4.63)	83.16 (-6.42)	97.79 (-0.09)
mPW2PLYP	Sappo-TZP/DZP	2.290 (-1.36)	2.066 (0.88)	95.71 (4.43)	83.28 (-6.28)	97.74 (-0.14)
mPW2PLYP	Sappo-QZP/DZP	2.288 (-1.42)	2.062 (0.68)	95.67 (4.39)	83.31 (-6.26)	97.72 (-0.16)
PBE0-DH	def2-TZVP	2.267 (-2.33)	2.066 (0.88)	95.40 (4.09)	83.16 (-6.43)	98.29 (0.42)
PBE0-DH	def2-QZVP	2.265 (-2.41)	2.061 (0.63)	95.75 (4.47)	82.98 (-6.63)	98.30 (0.43)
PBE0-DH	LANL2DZ	2.380 (2.52)	2.083 (1.71)	96.16 (4.92)	82.62 (-7.03)	98.60 (0.74)
PBE0-DH	LANL2TZ/def2-TZVP	2.271 (-2.18)	2.064 (0.78)	95.43 (4.12)	83.13 (-6.46)	98.32 (0.45)
PBE0-DH	ZORA-TZVP/TZVP	2.263 (-2.50)	2.060 (0.59)	95.35 (4.04)	83.27 (-6.30)	98.13 (0.26)

to continued ...

Method	Basis set	Pt-Cl	Pt-N	Cl-Pt-Cl	Cl-Pt-N	N-Pt-N
PBE0-DH	ZORA-TZVPP/TZVP	2.261 (-2.59)	2.058 (0.49)	95.35 (4.04)	83.25 (-6.32)	98.15 (0.28)
PBE0-DH	DKH-TZVP/TZVP	2.276 (-1.94)	1.947 (-4.93)	94.99 (3.64)	82.37 (-7.31)	100.29 (2.47)
PBE0-DH	DKH-TZVPP/TZVP	2.275 (-1.98)	1.934 (-5.57)	94.94 (3.59)	82.29 (-7.40)	100.50 (2.68)
PBE0-DH	Sappo-DZP/DZP	2.280 (-1.79)	2.055 (0.34)	95.85 (4.58)	83.12 (-6.46)	97.91 (0.03)
PBE0-DH	Sappo-TZP/DZP	2.271 (-2.15)	2.047 (-0.05)	95.72 (4.44)	83.23 (-6.35)	97.83 (-0.05)
PBE0-DH	Sappo-QZP/DZP	2.271 (-2.18)	2.044 (-0.20)	95.73 (4.45)	83.23 (-6.35)	97.82 (-0.06)
PBE-QIDH	def2-TZVP	2.259 (-2.67)	2.057 (0.44)	95.25 (3.93)	83.27 (-6.30)	98.22 (0.35)
PBE-QIDH	def2-QZVP	2.256 (-2.82)	2.050 (0.10)	95.71 (4.43)	82.99 (-6.62)	98.32 (0.45)
PBE-QIDH	LANL2DZ	2.383 (2.67)	2.090 (2.05)	96.08 (4.83)	82.87 (-6.75)	98.19 (0.32)
PBE-QIDH	LANL2TZ/def2-TZVP	2.267 (-2.35)	2.056 (0.39)	95.36 (4.05)	83.17 (-6.41)	98.32 (0.45)
PBE-QIDH	ZORA-TZVP/TZVP	2.255 (-2.84)	2.052 (0.20)	95.17 (3.84)	83.37 (-6.18)	98.09 (0.22)
PBE-QIDH	ZORA-TZVPP/TZVP	2.252 (-2.99)	2.049 (0.05)	95.14 (3.81)	83.39 (-6.17)	98.10 (0.23)
PBE-QIDH	DKH-TZVP/TZVP	-	-	-	-	-
PBE-QIDH	DKH-TZVPP/TZVP	2.267 (-2.33)	1.924 (-6.08)	94.82 (3.46)	82.30 (-7.39)	100.60 (2.78)
PBE-QIDH	Sappo-DZP/DZP	2.274 (-2.02)	2.048 (-0.02)	95.64 (4.35)	83.32 (-6.24)	97.72 (-0.16)
PBE-QIDH	Sappo-TZP/DZP	2.263 (-2.50)	2.040 (-0.39)	95.42 (4.11)	83.46 (-6.08)	97.66 (-0.22)

to continued ...

Method	Basis set	Pt-Cl	Pt-N	Cl-Pt-Cl	Cl-Pt-N	N-Pt-N
PBE-QIDH	Sappo-QZP/DZP	2.260 (-2.63)	2.035 (-0.63)	95.40 (4.09)	83.49 (-6.05)	97.62 (-0.26)
DSD-BLYP	def2-TZVP	2.267 (-2.33)	2.066 (0.85)	95.14 (3.81)	83.27 (-6.30)	98.32 (0.45)
DSD-BLYP	def2-QZVP	2.261 (-2.59)	2.058 (0.46)	95.56 (4.27)	83.02 (-6.58)	98.40 (0.54)
DSD-BLYP	LANL2DZ	2.401 (3.45)	2.112 (3.13)	96.24 (5.01)	82.81 (-6.82)	98.15 (0.28)
DSD-BLYP	LANL2TZ/def2-TZVP	2.278 (-1.85)	2.066 (0.88)	95.35 (4.04)	83.09 (-6.50)	98.47 (0.61)
DSD-BLYP	ZORA-TZVP/TZVP	2.261 (-2.59)	2.060 (0.59)	95.01 (3.67)	83.38 (-6.17)	98.23 (0.36)
DSD-BLYP	ZORA-TZVPP/TZVP	2.257 (-2.78)	2.058 (0.49)	94.98 (3.63)	83.41 (-6.14)	98.22 (0.35)
DSD-BLYP	DKH-TZVP/TZVP	2.275 (-2.00)	1.949 (-4.83)	94.72 (3.35)	82.45 (-7.22)	100.40 (2.58)
DSD-BLYP	DKH-TZVPP/TZVP	2.272 (-2.13)	1.936 (-5.47)	94.66 (3.28)	82.43 (-7.25)	100.51 (2.69)
DSD-BLYP	Sappo-DZP/DZP	2.284 (-1.62)	2.055 (0.32)	95.57 (4.28)	83.37 (-6.19)	97.70 (-0.18)
DSD-BLYP	Sappo-TZP/DZP	2.269 (-2.24)	2.047 (-0.05)	95.29 (3.97)	83.53 (-6.01)	97.67 (-0.21)
DSD-BLYP	Sappo-QZP/DZP	2.265 (-2.41)	2.041 (-0.34)	95.22 (3.90)	83.59 (-5.94)	97.61 (-0.27)
RI-SCS-MP2	def2-TZVP	2.265 (-2.41)	2.062 (0.68)	94.84 (3.48)	83.66 (-5.86)	97.85 (-0.03)
RI-SCS-MP2	def2-QZVP	-	-	-	-	-
RI-SCS-MP2	LANL2DZ	2.409 (3.79)	2.129 (3.96)	95.93 (4.67)	83.33 (-6.23)	97.41 (-0.48)
RI-SCS-MP2	LANL2TZ/def2-TZVP	2.282 (-1.68)	2.064 (0.76)	95.11 (3.78)	83.40 (-6.15)	98.09 (0.22)
RI-SCS-MP2	ZORA-TZVP/TZVP	2.261 (-2.61)	2.059 (0.51)	94.68 (3.31)	83.77 (-5.74)	97.80 (-0.08)

to continued ...

Method	Basis set	Pt-Cl	Pt-N	Cl-Pt-Cl	Cl-Pt-N	N-Pt-N
RI-SCS-MP2	ZORA-TZVPP/TZVP	2.254 (-2.91)	2.056 (0.37)	94.59 (3.21)	83.83 (-5.67)	97.76 (-0.12)
RI-SCS-MP2	DKH-TZVP/TZVP	2.275 (-1.98)	1.941 (-5.22)	94.99 (3.64)	82.47 (-7.20)	100.08 (2.25)
RI-SCS-MP2	DKH-TZVPP/TZVP	2.270 (-2.20)	1.930 (-5.79)	94.95 (3.60)	82.49 (-7.18)	100.08 (2.25)
RI-SCS-MP2	Sappo-DZP/DZP	2.285 (-1.57)	2.056 (0.37)	95.16 (3.83)	83.86 (-5.64)	97.13 (-0.76)
RI-SCS-MP2	Sappo-TZP/DZP	2.265 (-2.41)	2.048 (0.00)	94.75 (3.38)	84.06 (-5.41)	97.12 (-0.77)
RI-SCS-MP2	Sappo-QZP/DZP	2.258 (-2.71)	2.039 (-0.44)	94.65 (3.27)	84.17 (-5.28)	97.02 (-0.87)
HF	def2-TZVP	2.320 (-0.04)	2.121 (3.56)	95.80 (4.53)	84.34 (-5.10)	95.53 (-2.40)
HF	def2-QZVP	2.322 (0.04)	2.116 (3.32)	96.35 (5.13)	84.05 (-5.42)	95.56 (-2.37)
HF	LANL2DZ	2.415 (4.05)	2.126 (3.81)	97.15 (6.00)	83.53 (-6.00)	95.79 (-2.13)
HF	LANL2TZ/def2-TZVP	2.320 (-0.04)	2.114 (3.22)	95.82 (4.55)	84.34 (-5.10)	95.51 (-2.42)
HF	ZORA-TZVP/TZVP	2.317 (-0.19)	2.113 (3.17)	95.76 (4.48)	84.38 (-5.05)	95.49 (-2.44)
HF	ZORA-TZVPP/TZVP	2.316 (-0.24)	2.112 (3.13)	95.77 (4.50)	84.36 (-5.08)	95.53 (-2.40)
HF	DKH-TZVP/TZVP	2.325 (0.17)	2.013 (-1.71)	96.00 (4.75)	82.98 (-6.62)	98.03 (0.16)
HF	DKH-TZVPP/TZVP	2.325 (0.17)	2.008 (-1.95)	95.99 (4.74)	82.98 (-6.63)	98.06 (0.19)
HF	Sappo-DZP/DZP	-	-	-	-	-
HF	Sappo-TZP/DZP	2.327 (0.26)	2.107 (2.86)	96.15 (4.91)	84.33 (-5.11)	95.10 (-2.84)

to continued ...

Method	Basis set	Pt-Cl	Pt-N	Cl-Pt-Cl	Cl-Pt-N	N-Pt-N
HF	Sappo-QZP/DZP	2.327 (0.26)	2.103 (2.66)	96.17 (4.93)	84.32 (-5.11)	95.19 (-2.74)
BP86	Sappo-TZP/DZP	2.301 (-0.84)	2.072 (1.15)	96.37 (5.15)	82.29 (-7.99)	99.05 (1.20)
					MAPE	3.267
PBE	Sappo-TZP/DZP	2.297 (-1.01)	2.068 (0.96)	96.44 (5.22)	82.28 (-8.01)	99.00 (1.14)
					MAPE	3.269
B3P86	Sappo-TZP/DZP	2.289 (-1.37)	2.062 (0.71)	96.05 (4.80)	82.81 (-7.37)	98.37 (0.50)
					MAPE	2.950
B3PW91	Sappo-TZP/DZP	2.290 (-1.35)	2.064 (0.80)	96.01 (4.75)	82.88 (-7.28)	98.27 (0.40)
					MAPE	2.917
HF-by-MMFF94	LANL2DZ/6-31G(d)	2.360 (1.66)	2.127 (3.83)	95.70 (4.42)	84.50 (-4.91)	95.30 (-2.63)
					MAPE	3.491

end.

Dihedral angle Cl-Pt-N-H from cisplatin $[Pt(NH_3)_2Cl_2]$

Table S4: Calculated Dihedral Angle (in °) Cl-Pt-N-H in cisplatin $[Pt(NH_3)_2Cl_2]$ focused in H7 and H8 atoms. Atoms are numbered according to Figure 2. The level of theory used in the standard MMFF94 force field is included (HF-by-MMFF94). Abbreviated names for the basis sets are used here, with their full names listed in Table 1.

Method	Basis set	Cl2-Pt-N4-H7	Cl3-Pt-N5-H8
Experimental	-	-	-
M06L	def2-TZVP	0.03	0.07
M06L	def2-QZVP	-0.03	-0.03
M06L	LANL2DZ	-0.02	0.10
M06L	LANL2TZ/def2-TZVP	-0.01	0.11
M06L	ZORA-TZVP/TZVP	0.01	-0.02
M06L	ZORA-TZVPP/TZVP	0.00	-0.02
M06L	DKH-TZVP/TZVP	0.40	-0.02
M06L	DKH-TZVPP/TZVP	0.42	-0.07
M06L	Sappo-DZP/DZP	0.40	0.11
M06L	Sappo-TZP/DZP	0.12	-0.08
M06L	Sappo-QZP/DZP	0.16	-0.04
B3LYP	def2-TZVP	0.16	0.19
B3LYP	def2-QZVP	0.34	0.00
B3LYP	LANL2DZ	0.05	0.12
B3LYP	LANL2TZ/def2-TZVP	-0.04	0.02
B3LYP	ZORA-TZVP/TZVP	0.08	-2.08
B3LYP	ZORA-TZVPP/TZVP	0.07	-0.21
B3LYP	DKH-TZVP/TZVP	2.24	1.61
B3LYP	DKH-TZVPP/TZVP	4.16	3.30
B3LYP	Sappo-DZP/DZP	0.18	0.09

to continued ...

Method	Basis set	Cl2-Pt-N4-H7	Cl3-Pt-N5-H8
B3LYP	Sappo-TZP/DZP	0.15	0.07
B3LYP	Sappo-QZP/DZP	0.17	0.10
mPW1PW	def2-TZVP	0.08	0.11
mPW1PW	def2-QZVP	-0.02	0.26
mPW1PW	LANL2DZ	0.05	0.16
mPW1PW	LANL2TZ/def2-TZVP	0.08	0.11
mPW1PW	ZORA-TZVP/TZVP	0.10	-0.24
mPW1PW	ZORA-TZVPP/TZVP	0.15	-0.19
mPW1PW	DKH-TZVP/TZVP	-10.79	-10.68
mPW1PW	DKH-TZVPP/TZVP	-12.78	-12.62
mPW1PW	Sappo-DZP/DZP	0.74	0.11
mPW1PW	Sappo-TZP/DZP	0.20	0.17
mPW1PW	Sappo-QZP/DZP	0.17	0.15
PBE0	def2-TZVP	0.11	0.12
PBE0	def2-QZVP	0.32	-0.01
PBE0	LANL2DZ	0.04	0.14
PBE0	LANL2TZ/def2-TZVP	0.03	0.08
PBE0	ZORA-TZVP/TZVP	-0.24	0.08
PBE0	ZORA-TZVPP/TZVP	0.16	-0.16
PBE0	DKH-TZVP/TZVP	-13.19	-13.15
PBE0	DKH-TZVPP/TZVP	-14.31	-14.10
PBE0	Sappo-DZP/DZP	0.17	0.11
PBE0	Sappo-TZP/DZP	0.20	0.17
PBE0	Sappo-QZP/DZP	0.10	0.08

to continued ...

Method	Basis set	Cl2-Pt-N4-H7	Cl3-Pt-N5-H8
TPSSh	def2-TZVP	0.06	0.09
TPSSh	def2-QZVP	0.12	-0.02
TPSSh	LANL2DZ	0.04	0.12
TPSSh	LANL2TZ/def2-TZVP	0.03	0.06
TPSSh	ZORA-TZVP/TZVP	0.01	-0.09
TPSSh	ZORA-TZVPP/TZVP	0.03	-0.09
TPSSh	DKH-TZVP/TZVP	1.36	1.04
TPSSh	DKH-TZVPP/TZVP	3.05	2.62
TPSSh	Sappo-DZP/DZP	0.09	0.07
TPSSh	Sappo-TZP/DZP	0.11	0.09
TPSSh	Sappo-QZP/DZP	0.11	0.09
M06	def2-TZVP	0.49	0.50
M06	def2-QZVP	0.98	0.36
M06	LANL2DZ	0.55	0.73
M06	LANL2TZ/def2-TZVP	0.46	0.48
M06	ZORA-TZVP/TZVP	0.50	0.00
M06	ZORA-TZVPP/TZVP	0.49	0.00
M06	DKH-TZVP/TZVP	-14.34	-14.06
M06	DKH-TZVPP/TZVP	-14.78	-14.30
M06	Sappo-DZP/DZP	0.40	0.11
M06	Sappo-TZP/DZP	0.16	0.46
M06	Sappo-QZP/DZP	0.07	0.32
M06-2X	def2-TZVP	0.18	0.40
M06-2X	def2-QZVP	1.40	0.05

to continued ...

Method	Basis set	Cl2-Pt-N4-H7	Cl3-Pt-N5-H8
M06-2X	LANL2DZ	0.14	0.51
M06-2X	LANL2TZ/def2-TZVP	0.02	0.26
M06-2X	ZORA-TZVP/TZVP	0.61	-1.17
M06-2X	ZORA-TZVPP/TZVP	0.61	-1.12
M06-2X	DKH-TZVP/TZVP	-54.95	-59.65
M06-2X	DKH-TZVPP/TZVP	-54.93	-54.44
M06-2X	Sappo-DZP/DZP	0.65	0.03
M06-2X	Sappo-TZP/DZP	0.67	0.17
M06-2X	Sappo-QZP/DZP	0.66	0.15
LC-BLYP	def2-TZVP	0.06	-0.13
LC-BLYP	def2-QZVP	0.27	-0.09
LC-BLYP	LANL2DZ	-0.02	0.06
LC-BLYP	LANL2TZ/def2-TZVP	0.08	-0.14
LC-BLYP	ZORA-TZVP/TZVP	0.11	-0.21
LC-BLYP	ZORA-TZVPP/TZVP	0.10	-0.22
LC-BLYP	DKH-TZVP/TZVP	-14.86	-14.94
LC-BLYP	DKH-TZVPP/TZVP	-16.43	-16.86
LC-BLYP	Sappo-DZP/DZP	0.06	-0.06
LC-BLYP	Sappo-TZP/DZP	0.05	-0.07
LC-BLYP	Sappo-QZP/DZP	0.07	-0.07
CAM-B3LYP	def2-TZVP	0.02	-0.03
CAM-B3LYP	def2-QZVP	0.44	0.01
CAM-B3LYP	LANL2DZ	0.02	0.11
CAM-B3LYP	LANL2TZ/def2-TZVP	0.08	-0.02

to continued ...

Method	Basis set	Cl2-Pt-N4-H7	Cl3-Pt-N5-H8
CAM-B3LYP	ZORA-TZVP/TZVP	0.06	-0.39
CAM-B3LYP	ZORA-TZVPP/TZVP	0.05	-0.39
CAM-B3LYP	DKH-TZVP/TZVP	-14.39	-14.34
CAM-B3LYP	DKH-TZVPP/TZVP	-15.12	-14.99
CAM-B3LYP	Sappo-DZP/DZP	0.21	0.06
CAM-B3LYP	Sappo-TZP/DZP	0.11	-0.01
CAM-B3LYP	Sappo-QZP/DZP	0.25	0.12
ωB97X-D3BJ	def2-TZVP	0.06	-0.11
ωB97X-D3BJ	def2-QZVP	0.68	0.04
ωB97X-D3BJ	LANL2DZ	0.11	0.26
ωB97X-D3BJ	LANL2TZ/def2-TZVP	0.07	-0.13
ωB97X-D3BJ	ZORA-TZVP/TZVP	0.07	-0.53
ωB97X-D3BJ	ZORA-TZVPP/TZVP	0.17	-0.42
ωB97X-D3BJ	DKH-TZVP/TZVP	-20.00	-21.54
ωB97X-D3BJ	DKH-TZVPP/TZVP	-21.06	-22.24
ωB97X-D3BJ	Sappo-DZP/DZP	0.26	0.10
ωB97X-D3BJ	Sappo-TZP/DZP	0.28	0.13
ωB97X-D3BJ	Sappo-QZP/DZP	0.26	0.09
B97-3c	def2-TZVP	0.00	0.00
B97-3c	def2-QZVP	0.01	0.01
B97-3c	LANL2DZ	-0.03	-0.02
B97-3c	LANL2TZ/def2-TZVP	0.04	0.07
B97-3c	ZORA-TZVP/TZVP	0.14	0.17
B97-3c	ZORA-TZVPP/TZVP	0.02	0.02

to continued ...

Method	Basis set	Cl2-Pt-N4-H7	Cl3-Pt-N5-H8
B97-3c	DKH-TZVP/TZVP	-14.79	-14.21
B97-3c	DKH-TZVPP/TZVP	-15.81	-15.30
B97-3c	Sappo-DZP/DZP	-2.76	2.73
B97-3c	Sappo-TZP/DZP	-0.41	0.42
B97-3c	Sappo-QZP/DZP	17.71	-17.79
B2PLYP	def2-TZVP	-	-
B2PLYP	def2-QZVP	0.99	0.11
B2PLYP	LANL2DZ	0.01	0.21
B2PLYP	LANL2TZ/def2-TZVP	0.18	0.25
B2PLYP	ZORA-TZVP/TZVP	0.27	-0.76
B2PLYP	ZORA-TZVPP/TZVP	0.32	-0.69
B2PLYP	DKH-TZVP/TZVP	-14.79	-15.75
B2PLYP	DKH-TZVPP/TZVP	-15.70	-16.29
B2PLYP	Sappo-DZP/DZP	0.38	0.21
B2PLYP	Sappo-TZP/DZP	0.46	0.37
B2PLYP	Sappo-QZP/DZP	0.43	0.36
mPW2PLYP	def2-TZVP	0.27	0.37
mPW2PLYP	def2-QZVP	1.04	0.14
mPW2PLYP	LANL2DZ	0.11	0.32
mPW2PLYP	LANL2TZ/def2-TZVP	0.17	0.24
mPW2PLYP	ZORA-TZVP/TZVP	0.33	-0.79
mPW2PLYP	ZORA-TZVPP/TZVP	0.37	-0.72
mPW2PLYP	DKH-TZVP/TZVP	-15.87	-16.58
mPW2PLYP	DKH-TZVPP/TZVP	-16.53	-17.28

to continued ...

Method	Basis set	Cl2-Pt-N4-H7	Cl3-Pt-N5-H8
mPW2PLYP	Sappo-DZP/DZP	0.40	0.24
mPW2PLYP	Sappo-TZP/DZP	0.50	0.42
mPW2PLYP	Sappo-QZP/DZP	0.47	0.41
PBE0-DH	def2-TZVP	0.11	0.17
PBE0-DH	def2-QZVP	0.76	0.05
PBE0-DH	LANL2DZ	-0.03	0.21
PBE0-DH	LANL2TZ/def2-TZVP	0.10	0.16
PBE0-DH	ZORA-TZVP/TZVP	0.35	0.26
PBE0-DH	ZORA-TZVPP/TZVP	0.28	-0.56
PBE0-DH	DKH-TZVP/TZVP	-16.97	-18.59
PBE0-DH	DKH-TZVPP/TZVP	84	-21.95
PBE0-DH	Sappo-DZP/DZP	0.40	0.29
PBE0-DH	Sappo-TZP/DZP	0.35	0.26
PBE0-DH	Sappo-QZP/DZP	0.35	0.35
PBE-QIDH	def2-TZVP	0.27	0.28
PBE-QIDH	def2-QZVP	1.30	0.23
PBE-QIDH	LANL2DZ	0.14	0.46
PBE-QIDH	LANL2TZ/def2-TZVP	0.22	0.27
PBE-QIDH	ZORA-TZVP/TZVP	0.56	-0.95
PBE-QIDH	ZORA-TZVPP/TZVP	0.57	-0.92
PBE-QIDH	DKH-TZVP/TZVP	-	-
PBE-QIDH	DKH-TZVPP/TZVP	-21.81	-28.39
PBE-QIDH	Sappo-DZP/DZP	0.57	0.42
PBE-QIDH	Sappo-TZP/DZP	0.55	0.51

to continued ...

Method	Basis set	Cl2-Pt-N4-H7	Cl3-Pt-N5-H8
PBE-QIDH	Sappo-QZP/DZP	0.51	0.56
DSD-BLYP	def2-TZVP	0.30	0.37
DSD-BLYP	def2-QZVP	1.58	0.26
DSD-BLYP	LANL2DZ	0.12	0.47
DSD-BLYP	LANL2TZ/def2-TZVP	0.35	0.41
DSD-BLYP	ZORA-TZVP/TZVP	0.79	-1.51
DSD-BLYP	ZORA-TZVPP/TZVP	0.78	-1.01
DSD-BLYP	DKH-TZVP/TZVP	-20.18	-24.58
DSD-BLYP	DKH-TZVPP/TZVP	-20.79	-25.69
DSD-BLYP	Sappo-DZP/DZP	0.75	0.57
DSD-BLYP	Sappo-TZP/DZP	0.64	0.56
DSD-BLYP	Sappo-QZP/DZP	0.54	0.52
RI-SCS-MP2	def2-TZVP	0.65	0.67
RI-SCS-MP2	def2-QZVP	-	-
RI-SCS-MP2	LANL2DZ	0.61	0.90
RI-SCS-MP2	LANL2TZ/def2-TZVP	0.87	0.91
RI-SCS-MP2	ZORA-TZVP/TZVP	1.91	-2.57
RI-SCS-MP2	ZORA-TZVPP/TZVP	1.87	-2.53
RI-SCS-MP2	DKH-TZVP/TZVP	-33.05	-57.38
RI-SCS-MP2	DKH-TZVPP/TZVP	-35.32	-57.73
RI-SCS-MP2	Sappo-DZP/DZP	1.33	0.84
RI-SCS-MP2	Sappo-TZP/DZP	1.23	1.16
RI-SCS-MP2	Sappo-QZP/DZP	1.13	1.39
HF	def2-TZVP	0.89	0.32

to continued ...

Method	Basis set	Cl2-Pt-N4-H7	Cl3-Pt-N5-H8
HF	def2-QZVP	4.67	1.47
HF	LANL2DZ	0.47	1.17
HF	LANL2TZ/def2-TZVP	0.89	0.25
HF	ZORA-TZVP/TZVP	0.55	-5.24
HF	ZORA-TZVPP/TZVP	0.57	-5.21
HF	DKH-TZVP/TZVP	-55.49	-61.67
HF	DKH-TZVPP/TZVP	-55.45	-61.63
HF	Sappo-DZP/DZP	-	-
HF	Sappo-TZP/DZP	2.18	1.16
HF	Sappo-QZP/DZP	2.40	0.91
BP86	Sappo-TZP/DZP	-0.07	-0.05
PBE	Sappo-TZP/DZP	0.04	0.09
B3P86	Sappo-TZP/DZP	0.07	0.11
B3PW91	Sappo-TZP/DZP	0.06	0.11
HF-by-MMFF94	LANL2DZ/6-31G(d)	-1.85	-7.75

end.

Mean Absolute Percentage Erro (MAPE) calculated with all methods and basis sets for structural parameters in the gas phase

PtH

	RECP				RAE				RAEPt-NRAE		
Level / basis set	def2-TZVP	def2-QZVP	LANL2DZ	LANL2TZ/ def2-TZVP	ZORA-TZVP/ TZVP	ZORA-TZVPP/ TZVP	DKH-TZVP/ TZVP	DKH-TZVPP/TZVP	Sappo-DZP/ DZP	Sappo-TZP/ DZP	Sappo-QZP/ DZP
M06L	0.405	0.240	1.016	0.682	0.440	0.373	0.145	0.178	0.552	0.469	0.217
B3LYP	0.262	0.031	0.996	0.242	0.039	0.126	0.672	0.758	0.131	0.055	0.255
mPW1PW	0.372	0.583	0.378	0.259	0.609	0.704	1.241	1.334	0.474	0.649	0.830
PBE0	0.371	0.586	0.427	0.270	0.592	0.688	1.223	1.317	0.478	0.645	0.819
TPSSh	0.171	0.010	0.738	0.153	0.141	0.231	0.776	0.865	0.031	0.130	0.310
M06	0.111	0.314	1.593	1.311	1.186	1.088	0.558	0.459	1.326	1.276	1.054
M062X	1.375	1.658	0.267	0.865	1.734	1.861	2.336	2.459	1.387	1.652	1.902
LC-BLYP	0.005	0.196	0.740	0.026	0.305	0.392	0.924	1.009	0.169	0.359	0.553
CAM-B3LYP	0.088	0.126	0.800	0.023	0.218	0.307	0.837	0.924	0.058	0.233	0.431
ωB97X-D3BJ	0.528	0.391	0.860	0.207	0.230	0.186	0.383	0.428	0.249	0.106	0.033
B97-3c	1.255	1.521	0.543	1.204	1.530	1.630	2.217	2.316	18.857	8.080	8.279
B2PLYP	0.444	0.960	1.293	0.446	0.757	0.878	1.363	1.484	0.842	1.045	1.104
mPW2PLYP	0.405	0.898	1.241	0.471	0.745	0.865	1.353	1.471	0.812	0.878	1.078
PBE0-DH	not opt	1.048	0.543	0.668	0.945	1.058	1.554	1.668	0.954	1.045	1.217
PBE-QIDH	not opt	1.740	0.793	1.071	1.457	1.595	2.052	2.190	1.601	1.635	1.818
DSD-BLYP	1.344	2.111	1.236	1.149	1.708	1.864	2.296	2.452	1.929	1.948	2.151
RI-SCS-MP2	2.199	not opt	1.397	1.798	2.529	2.684	3.107	3.262	2.877	2.823	3.051
HF	2.045	1.984	2.965	0.692	1.781	1.697	1.194	1.107	1.876	1.971	1.796
**Minor MAPE**	**0.005**	0.010	0.267	0.023	0.039	0.126	0.145	0.178	0.031	0.055	0.033
	**LC-BLYP**	TPSSh	M062X	CAM-B3LYP	B3LYP	B3LYP	M06L	M06L	TPSSh	B3LYP	ωB97X-D3BJ

meta-GGA
GGA hybrid
meta-GGA hybrid
range-separated hybrid
GGA composite
Double hybrid
Hartree-Fock
Post-Hartree Fock

Figure S1: Calculated MAPE (in %) for optimized geometry of PtH molecule with all methods and basis sets in the gas phase. MAPEs were calculated from bond length Pt-H reported in Table S1. MAPEs with values lower or equal to 0.1 are colored dark green, and those higher than 0.1 vary toward dark red. Abbreviated names for the basis sets are used here, with their full names listed in Table 1. not opt: not geometric optimization.

PtCl

	RECP				RAE				RAEPt-NRAE		
Level / basis set	def2-TZVP	def2-QZVP	LANL2DZ	LANL2TZ/ def2-TZVP	ZORA-TZVP/ TZVP	ZORA-TZVPP/ TZVP	DKH-TZVP/ TZVP	DKH-TZVPP/ TZVP	Sappo-DZP/ DZP	Sappo-TZP/ DZP	Sappo-QZP/ DZP
M06L	2.941	2.556	8.316	0.396	not opt	2.281	not opt	1.960	3.646	3.034	2.910
B3LYP	3.476	3.224	8.759	0.950	0.808	0.518	0.477	0.132	1.889	1.192	1.053
mPW1PW	2.320	2.072	7.864	0.140	1.940	1.776	1.649	1.448	3.036	2.378	2.323
PBE0	2.176	1.914	7.765	0.295	1.820	1.654	1.527	1.323	2.947	2.263	2.215
TPSSh	2.532	2.294	8.158	0.068	6.785	1.987	1.849	1.659	3.203	2.573	2.497
M06	3.603	3.326	8.401	not opt	3.653	3.500	3.275	3.227	4.471	4.032	3.909
M062X	2.864	2.558	8.401	3.055	2.597	2.379	2.354	2.103	4.156	3.125	3.268
LC-BLYP	1.995	1.787	7.109	0.560	1.585	1.412	1.294	1.084	2.630	1.967	1.922
CAM-B3LYP	2.981	2.763	8.089	8.089	2.556	2.391	2.299	2.100	3.639	2.996	2.955
ωB97X-D3BJ	0.916	0.517	6.290	2.544	0.497	0.209	0.166	0.176	1.595	0.914	0.771
B97-3c	0.789	1.318	6.061	not opt	1.114	1.660	not opt	2.216	2.886	not opt	0.299
B2PLYP	0.409	0.290	7.619	2.720	0.205	0.543	0.569	0.972	1.092	0.207	0.130
mPW2PLYP	0.617	0.057	7.620	2.706	0.016	0.353	0.371	0.769	1.252	0.384	0.059
PBE0-DH	1.958	1.610	8.015	1.880	0.422	0.735	1.320	1.075	2.805	2.058	1.928
PBE-QIDH	0.306	1.042	7.265	1.670	0.801	1.158	1.192	1.620	0.565	0.360	0.773
DSD-BLYP	0.347	1.245	8.066	1.998	0.987	1.390	1.386	1.863	0.459	0.576	1.075
RI-SCS-MP2	0.086	not opt	9.212	1.904	0.558	0.968	0.928	1.415	1.050	0.269	0.950
HF	6.293	5.988	10.842	5.120	5.870	5.601	5.754	5.466	7.102	6.501	6.337
Minor MAPE	0.086 RI-SCS-MP2	0.057 mPW2PLYP	6.061 B97-3c	0.068 TPSSh	**0.016 mPW2PLYP**	0.209 ωB97X-D3BJ	0.166 ωB97X-D3BJ	0.132 B3LYP	0.459 DSD-BLYP	0.207 B2PLYP	0.059 mPW2PLYP

meta-GGA
GGA hybrid
meta-GGA hybrid
range-separated hybrid
GGA composite
Double hybrid
Hartree-Fock
Post-Hartree Fock

Figure S2: Calculated MAPE (in %) for optimized geometry of PtCl molecule with all methods and basis sets in the gas phase. MAPEs were calculated from bond length Pt-Cl reported in Table S1. MAPEs with values lower or equal to 0.1 are colored dark green, and those higher than 0.1 vary toward dark red. Abbreviated names for the basis sets are used here, with their full names listed in Table 1. not opt: not geometric optimization.

$[PtCl_4]^{2-}$

Level / basis set	RECP				RAE				RAEPt-NRAE		
	def2-TZVP	def2-QZVP	LANL2DZ	LANL2TZ/ def2-TZVP	ZORA-TZVP/ TZVP	ZORA-TZVPP/ TZVP	DKH-TZVP/ TZVP	DKH-TZVPP/ TZVP	Sappo-DZP/ DZP	Sappo-TZP/ DZP	Sappo-QZP/ DZP
M06L	3.48	3.09	7.78	3.35	3.13	3.09	3.00	2.96	4.00	3.61	3.65
B3LYP	3.70	3.43	7.48	3.61	3.43	3.39	3.30	3.26	4.09	3.74	3.74
mPW1PW	7.83	1.83	6.04	2.04	1.87	1.78	1.70	1.65	2.57	2.17	2.17
PBE0	1.91	1.65	5.96	1.87	1.70	1.65	1.57	1.48	2.39	2.00	2.00
TPSSh	2.57	2.26	6.57	2.57	2.30	2.26	2.17	2.09	2.96	2.61	2.61
M06	3.35	3.09	6.91	3.35	3.30	3.22	3.17	3.09	3.87	3.57	3.57
M062X	3.35	3.35	7.13	3.22	3.22	3.17	3.09	3.04	4.04	3.65	3.78
LC-BLYP	1.43	1.26	5.17	1.39	1.22	1.13	1.04	1.00	1.78	1.39	1.39
CAM-B3LYP	2.65	2.43	6.30	2.57	2.39	2.35	2.26	2.22	3.00	2.65	2.65
ωB97X-D3BJ	1.83	1.61	5.39	1.74	1.61	1.57	1.48	1.43	2.30	1.96	1.96
B97-3c	2.30	1.96	6.57	2.26	1.96	1.87	1.78	1.70	not opt	not opt	not opt
B2PLYP	2.52	2.17	7.35	2.74	2.30	2.17	2.17	2.00	3.17	2.61	2.43
mPW2PLYP	2.43	2.13	7.13	2.61	2.22	2.09	2.09	1.96	3.09	2.52	2.39
PBE0-DH	1.30	1.04	5.74	1.39	1.13	1.04	0.96	0.87	1.91	1.43	1.39
PBE-QIDH	0.87	0.52	5.96	1.13	0.70	0.52	0.52	0.35	1.61	1.00	0.83
DSD-BLYP	1.30	0.87	6.96	1.78	1.09	0.87	0.96	0.74	2.13	1.39	1.13
RI-SCS-MP2	1.26	not opt	7.43	2.00	1.04	0.78	0.91	0.61	2.22	1.30	0.87
HF	4.61	4.61	7.91	4.48	4.52	4.48	4.43	4.39	5.17	4.91	4.87
Minor MAPE	0.87 PBE-QIDH	0.52 PBE-QIDH	5.17 LC-BLYP	1.13 PBE-QIDH	0.70 PBE-QIDH	0.52 PBE-QIDH	0.52 PBE-QIDH	**0.35 PBE-QIDH**	1.61 PBE-QIDH	1.00 PBE-QIDH	0.83 PBE-QIDH

- meta-GGA
- GGA hybrid
- meta-GGA hybrid
- range-separated hybrid
- GGA composite
- Double hybrid
- Hartree-Fock
- Post-Hartree Fock

Figure S3: Calculated MAPE (in %) for optimized geometry of $[PtCl_4]^{2-}$ molecule with all methods and basis sets in the gas phase. MAPEs were calculated from bond length Pt-Cl reported in Table S2. MAPEs with values lower or equal to 1 are colored dark green, and those higher than 1 vary toward dark red. Abbreviated names for the basis sets are used here, with their full names listed in Table 1. not opt: not geometric optimization.

$[Pt(NH_3)_4]^{2+}$

	RECP				RAE				RAEPt-NRAE		
Level / basis set	def2-TZVP	def2-QZVP	LANL2DZ	LANL2TZ/ def2-TZVP	ZORA-TZVP/ TZVP	ZORA-TZVPP/ TZVP	DKH-TZVP/ TZVP	DKH-TZVPP/ TZVP	Sappo-DZP/ DZP	Sappo-TZP/ DZP	Sappo-QZP/ DZP
M06L	3.09	not opt	not opt	2.94	2.75	2.70	not opt	not opt	not opt	not opt	not opt
B3LYP	2.89	2.80	3.48	2.85	2.55	2.50	not opt	not opt	2.31	2.06	1.86
mPW1PW	1.67	1.62	2.40	1.62	1.37	1.32	not opt	not opt	1.18	0.93	0.74
PBE0	1.52	1.47	2.26	1.47	1.23	1.18	not opt	not opt	1.08	0.79	0.64
TPSSh	2.26	2.21	2.94	2.21	1.96	1.91	not opt	not opt	1.72	1.47	1.32
M06	2.85	not opt	3.39	2.85	2.65	2.60	not opt	not opt	2.31	2.16	2.01
M062X	2.50	2.45	3.34	2.31	2.16	2.11	not opt	not opt	2.06	1.67	not opt
LC-BLYP	1.28	1.18	1.82	1.23	0.93	0.93	not opt	not opt	0.59	0.39	0.25
CAM-B3LYP	2.11	2.06	2.70	2.06	1.82	1.77	not opt	not opt	1.52	1.28	1.13
ωB97X-D3BJ	1.82	1.77	2.50	1.77	1.57	1.52	not opt	not opt	1.37	not opt	0.98
B97-3c	1.77	1.67	2.55	1.77	1.37	1.32	not opt	not opt	3.19	6.18	0.49
B2PLYP	1.91	not opt	3.63	1.96	1.62	1.52	not opt	not opt	not opt	1.18	0.93
mPW2PLYP	1.82	not opt	3.48	1.86	1.57	1.47	not opt	not opt	not opt	1.08	not opt
PBE0-DH	0.93	not opt	not opt	0.93	0.69	0.64	not opt	not opt	0.59	0.29	0.10
PBE-QIDH	0.54	not opt	2.55	0.59	0.34	not opt	not opt	not opt	0.25	0.05	not opt
DSD-BLYP	0.88	not opt	2.55	1.03	0.64	0.49	not opt	not opt	0.54	not opt	not opt
RI-SCS-MP2	0.74	not opt	4.27	1.03	0.54	0.39	not opt	not opt	not opt	not opt	not opt
HF	3.34	3.29	4.12	3.04	3.04	3.04	6.72	not opt	3.14	not opt	2.70
Minor MAPE	0.54	1.18	1.82	0.59	0.34	0.39	6.72	-	0.25	**0.05**	0.10
	PBE-QIDH	LC-BLYP	LC-BLYP	PBE-QIDH	PBE-QIDH	RI-SCS-MP2	HF	-	PBE-QIDH	**PBE-QIDH**	PBE0-DH

meta-GGA
GGA hybrid
meta-GGA hybrid
range-separated hybrid
GGA composite
Double hybrid
Hartree-Fock
Post-Hartree Fock

Figure S4: Calculated MAPE (in %) for optimizes geometry of $[Pt(NH_3)_4]^{2+}$ molecule with all methods and basis sets in the gas phase. MAPEs were calculated from bond length Pt-N reported in Table S2 of Supporting Information. MAPEs with values lower or equal to 1 are colored dark green, and those higher than 1 vary toward dark red. Abbreviated names for the basis sets are used here, with their full names listed in Table 1. not opt: not geometric optimization.

Cisplatin [Pt(NH_3)$_2$$Cl_2$]

Level / basis set	def2-TZVP	def2-QZVP	LANL2DZ	LANL2TZ/ def2-TZVP	ZORA-TZVP/ TZVP	ZORA-TZVPP/ TZVP	DKH-TZVP/ TZVP	DKH-TZVPP/ TZVP	Sappo-DZP/ DZP	Sappo-TZP/ DZP	Sappo-QZP/ DZP
	RECP				RAE				RAEPt-NRAE		
M06L	3.59	3.52	4.79	3.55	3.38	3.39	3.33	3.41	3.57	3.06	3.06
B3LYP	3.02	2.97	4.36	2.99	2.91	2.94	3.07	3.18	2.87	2.81	2.79
mPW1PW	2.99	3.00	3.69	2.95	2.88	2.96	3.79	3.93	2.85	2.76	2.75
PBE0	3.27	3.27	4.15	3.23	3.17	3.18	3.70	3.80	3.12	3.04	3.01
TPSSh	3.27	3.27	4.15	3.23	3.17	3.18	3.70	3.80	3.12	3.04	3.01
M06	3.15	3.13	4.12	3.11	2.92	2.94	3.11	3.21	2.82	2.77	2.77
M062X	2.95	3.04	4.17	2.85	2.87	2.88	3.91	4.07	2.88	2.81	2.78
LC-BLYP	3.06	2.98	2.98	3.02	2.90	2.92	4.03	4.17	2.95	2.93	2.97
CAM-B3LYP	2.90	2.86	3.84	2.86	2.78	2.80	3.44	3.54	2.78	2.71	2.69
ωB97X-D3BJ	2.74	2.77	3.34	2.71	2.69	2.69	3.50	3.63	2.70	2.66	2.62
B97-3c	2.95	2.89	3.89	2.92	2.79	2.79	3.92	4.10	5.87	6.80	7.24
B2PLYP	not opt	2.94	4.07	2.87	2.79	2.80	3.49	3.62	2.65	2.60	2.55
mPW2PLYP	2.85	2.91	3.96	2.83	2.76	2.77	3.52	3.64	2.66	2.62	2.58
PBE0-DH	2.83	2.92	3.38	2.80	2.74	2.74	4.06	4.24	2.64	2.61	2.65
PBE-QIDH	2.74	2.88	3.33	2.73	2.66	2.65	not opt	4.41	2.56	2.66	2.73
DSD-BLYP	2.75	2.89	3.74	2.77	2.67	2.68	4.00	4.16	2.52	2.50	2.57
RI-SCS-MP2	2.49	not opt	3.83	2.52	2.45	2.45	4.06	4.20	2.43	2.39	2.52
HF	3.13	3.26	4.40	3.07	3.07	3.07	2.68	2.74	not opt	3.19	3.14
Minor MAPE	2.49<br>RI-SCS-MP2	2.77<br>ωB97X-D3BJ	2.98<br>LC-BLYP	2.52<br>RI-SCS-MP2	2.45<br>RI-SCS-MP2	2.45<br>RI-SCS-MP2	2.68<br>HF	2.74<br>HF	2.43<br>RI-SCS-MP2	**2.40**<br>**RI-SCS-MP2**	2.52<br>RI-SCS-MP2

meta-GGA
GGA hybrid
meta-GGA hybrid
range-separated hybrid
GGA composite
Double hybrid
Hartree-Fock
Post-Hartree Fock

Figure S5: Calculated MAPE (in %) for optimized geometry of cisplatin [Pt(NH_3)$_2$$Cl_2$] molecule with all methods and basis sets in the gas phase. MAPEs were calculated from five structural parameters: Pt-Cl, Pt-N, Cl-Pt-Cl, N-Pt-N, and Cl-Pt-N reported in Table S3. MAPEs with values lower or equal to 2.5 are colored dark green, and those higher than 2.5 vary toward dark red. Abbreviated names for the basis sets are used here, with their full names listed in Table 1. not opt: not geometric optimization.

Harmonic Vibrational frequencies calculated with all methods and basis sets in the gas phase

PtH and PtCl

Table S5: Calculated wavenumbers (in cm^{-1}) for Harmonic Vibrational Frequency of PtH and PtCl molecules with IR Intensities (in km/mol) in brackets. The level of theory used in the standard MMFF94 force field is included (HF-by-MMFF94). Abbreviated names for the basis sets are used here, with their full names listed in Table 1.

Method	Basis set	PtH	PtCl
Experimental	-	2378[1]	399[6]
M06L	def2-TZVP	2337 [31]	373 [16]
M06L	def2-QZVP	2328 [29]	376 [16]
M06L	LANL2DZ	2289 [57]	339 [16]
M06L	LANL2TZ/def2-TZVP	2343 [12]	397 [23]
M06L	ZORA-TZVP/TZVP	2365 [13]	-
M06L	ZORA-TZVPP/TZVP	2373 [22]	386 [16]
M06L	DKH-TZVP/TZVP	2375 [13]	-
M06L	DKH-TZVPP/TZVP	2375 [13]	382 [16]
M06L	Sappo-DZP/DZP	2348 [37]	369 [15]
M06L	Sappo-TZP/DZP	2330 [25]	376 [17]
M06L	Sappo-QZP/DZP	2332 [19]	374 [16]
B3LYP	def2-TZVP	2339 [29]	372 [14]
B3LYP	def2-QZVP	2344 [26]	371 [13]
B3LYP	LANL2DZ	2286 [35]	338 [11]
B3LYP	LANL2TZ/def2-TZVP	2370 [13]	384 [22]
B3LYP	ZORA-TZVP/TZVP	2374 [18]	380 [20]

to continued ...

Method	Basis set	PtH	PtCl
B3LYP	ZORA-TZVPP/TZVP	2378 [18]	383 [20]
B3LYP	DKH-TZVP/TZVP	2386 [18]	374 [21]
B3LYP	DKH-TZVPP/TZVP	2390 [18]	376 [21]
B3LYP	Sappo-DZP/DZP	2355 [23]	368 [22]
B3LYP	Sappo-TZP/DZP	2342 [28]	376 [23]
B3LYP	Sappo-QZP/DZP	2344 [25]	374 [22]
mPW1PW	def2-TZVP	2390 [34]	389 [15]
mPW1PW	def2-QZVP	2392 [30]	389 [14]
mPW1PW	LANL2DZ	2317 [48]	349 [13]
mPW1PW	LANL2TZ/def2-TZVP	2411 [17]	400 [25]
mPW1PW	ZORA-TZVP/TZVP	2417 [23]	393 [14]
mPW1PW	ZORA-TZVPP/TZVP	2421 [24]	394 [14]
mPW1PW	DKH-TZVP/TZVP	2429 [23]	389 [14]
mPW1PW	DKH-TZVPP/TZVP	2434 [24]	389 [14]
mPW1PW	Sappo-DZP/DZP	2401 [27]	383 [13]
mPW1PW	Sappo-TZP/DZP	2387 [34]	389 [14]
mPW1PW	Sappo-QZP/DZP	2387 [30]	386 [14]
PBE0	def2-TZVP	2389 [33]	391 [15]
PBE0	def2-QZVP	2391 [29]	390 [14]
PBE0	LANL2DZ	2315 [46]	351 [13]
PBE0	LANL2TZ/def2-TZVP	2411 [16]	403 [25]
PBE0	ZORA-TZVP/TZVP	2414 [23]	395 [14]
PBE0	ZORA-TZVPP/TZVP	2418 [23]	396 [14]
PBE0	DKH-TZVP/TZVP	2426 [23]	390 [14]
PBE0	DKH-TZVPP/TZVP	2431 [23]	390 [14]

to continued ...

Method	Basis set	PtH	PtCl
PBE0	Sappo-DZP/DZP	2398 [29]	382 [13]
PBE0	Sappo-TZP/DZP	2385 [34]	390 [14]
PBE0	Sappo-QZP/DZP	2383 [31]	387 [14]
TPSSh	def2-TZVP	2371 [29]	386 [13]
TPSSh	def2-QZVP	2371 [26]	386 [12]
TPSSh	LANL2DZ	2307 [45]	347 [11]
TPSSh	LANL2TZ/def2-TZVP	2395 [15]	401 [20]
TPSSh	ZORA-TZVP/TZVP	2398 [19]	390 [12]
TPSSh	ZORA-TZVPP/TZVP	2402 [19]	391 [12]
TPSSh	DKH-TZVP/TZVP	2411 [18]	386 [12]
TPSSh	DKH-TZVPP/TZVP	2414 [19]	386 [12]
TPSSh	Sappo-DZP/DZP	2382 [24]	381 [11]
TPSSh	Sappo-TZP/DZP	2368 [30]	386 [12]
TPSSh	Sappo-QZP/DZP	2367 [26]	384 [12]
M06	def2-TZVP	2274 [40]	374 [15]
M06	def2-QZVP	2267 [34]	374 [15]
M06	LANL2DZ	2229 [52]	344 [16]
M06	LANL2TZ/def2-TZVP	2249 [22]	-
M06	ZORA-TZVP/TZVP	2255 [43]	373 [17]
M06	ZORA-TZVPP/TZVP	2259 [44]	374 [17]
M06	DKH-TZVP/TZVP	2266 [44]	370 [17]
M06	DKH-TZVPP/TZVP	2270 [45]	369 [17]
M06	Sappo-DZP/DZP	2239 [48]	369 [14]
M06	Sappo-TZP/DZP	2215 [55]	371 [17]
M06	Sappo-QZP/DZP	2216 [46]	371 [15]

to continued ...

Method	Basis set	PtH	PtCl
M06-2X	def2-TZVP	2470 [35]	368 [23]
M06-2X	def2-QZVP	2487 [28]	365 [21]
M06-2X	LANL2DZ	2354 [57]	344 [16]
M06-2X	LANL2TZ/def2-TZVP	2437 [27]	376 [19]
M06-2X	ZORA-TZVP/TZVP	2484 [29]	367 [22]
M06-2X	ZORA-TZVPP/TZVP	2493 [29]	368 [22]
M06-2X	DKH-TZVP/TZVP	2495 [29]	362 [22]
M06-2X	DKH-TZVPP/TZVP	2505 [29]	363 [23]
M06-2X	Sappo-DZP/DZP	2438 [40]	352 [23]
M06-2X	Sappo-TZP/DZP	2436 [46]	360 [23]
M06-2X	Sappo-QZP/DZP	2442 [42]	353 [23]
LC-BLYP	def2-TZVP	2392 [51]	402 [18]
LC-BLYP	def2-QZVP	2393 [43]	400 [17]
LC-BLYP	LANL2DZ	2332 [57]	367 [16]
LC-BLYP	LANL2TZ/def2-TZVP	2423 [27]	415 [31]
LC-BLYP	ZORA-TZVP/TZVP	2423 [33]	406 [17]
LC-BLYP	ZORA-TZVPP/TZVP	2428 [33]	407 [17]
LC-BLYP	DKH-TZVP/TZVP	2433 [33]	401 [17]
LC-BLYP	DKH-TZVPP/TZVP	2438 [34]	402 [17]
LC-BLYP	Sappo-DZP/DZP	2408 [37]	399 [16]
LC-BLYP	Sappo-TZP/DZP	2394 [44]	404 [17]
LC-BLYP	Sappo-QZP/DZP	2396 [40]	401 [17]
CAM-B3LYP	def2-TZVP	2364 [57]	384 [18]
CAM-B3LYP	def2-QZVP	2366 [49]	383 [17]
CAM-B3LYP	LANL2DZ	2308 [64]	351 [15]

to continued ...

Method	Basis set	PtH	PtCl
CAM-B3LYP	LANL2TZ/def2-TZVP	2400 [29]	351 [15]
CAM-B3LYP	ZORA-TZVP/TZVP	2397 [39]	388 [17]
CAM-B3LYP	ZORA-TZVPP/TZVP	2401 [39]	389 [17]
CAM-B3LYP	DKH-TZVP/TZVP	2408 [39]	384 [17]
CAM-B3LYP	DKH-TZVPP/TZVP	2414 [39]	384 [17]
CAM-B3LYP	Sappo-DZP/DZP	2379 [45]	380 [16]
CAM-B3LYP	Sappo-TZP/DZP	2364 [51]	385 [17]
CAM-B3LYP	Sappo-QZP/DZP	2367 [47]	382 [17]
ωB97X-D3BJ	def2-TZVP	2331 [65]	385 [33]
ωB97X-D3BJ	def2-QZVP	2325 [56]	386 [32]
ωB97X-D3BJ	LANL2DZ	2267 [69]	353 [30]
ωB97X-D3BJ	LANL2TZ/def2-TZVP	2422 [82]	402 [19]
ωB97X-D3BJ	ZORA-TZVP/TZVP	2357 [47]	390 [31]
ωB97X-D3BJ	ZORA-TZVPP/TZVP	2356 [48]	393 [32]
ωB97X-D3BJ	DKH-TZVP/TZVP	2362 [46]	384 [32]
ωB97X-D3BJ	DKH-TZVPP/TZVP	2362 [47]	386 [32]
ωB97X-D3BJ	Sappo-DZP/DZP	2356 [43]	379 [35]
ωB97X-D3BJ	Sappo-TZP/DZP	2341 [53]	386 [34]
ωB97X-D3BJ	Sappo-QZP/DZP	2336 [51]	383 [33]
B97-3c	def2-TZVP	2388 [9]	389 [13]
B97-3c	def2-QZVP	2396 [7]	394 [12]
B97-3c	LANL2DZ	2318 [22]	339 [13]
B97-3c	LANL2TZ/def2-TZVP	2422 [9]	-
B97-3c	ZORA-TZVP/TZVP	2416 [4]	384 [18]
B97-3c	ZORA-TZVPP/TZVP	2421 [5]	401 [12]

to continued ...

Method	Basis set	PtH	PtCl
B97-3c	DKH-TZVP/TZVP	2431 [4]	-
B97-3c	DKH-TZVPP/TZVP	2435 [4]	393 [13]
B97-3c	Sappo-DZP/DZP	3204 [5]	434 [22]
B97-3c	Sappo-TZP/DZP	3575 [11]	-
B97-3c	Sappo-QZP/DZP	3441 [16]	432 [1]
B2PLYP	def2-TZVP	2418 [27]	386 [31]
B2PLYP	def2-QZVP	2425 [19]	392 [29]
B2PLYP	LANL2DZ	2261 [54]	334 [22]
B2PLYP	LANL2TZ/def2-TZVP	2419 [19]	393 [14]
B2PLYP	ZORA-TZVP/TZVP	2448 [12]	394 [29]
B2PLYP	ZORA-TZVPP/TZVP	2456 [12]	398 [29]
B2PLYP	DKH-TZVP/TZVP	2460 [12]	388 [29]
B2PLYP	DKH-TZVPP/TZVP	2468 [12]	391 [30]
B2PLYP	Sappo-DZP/DZP	2447 [22]	376 [32]
B2PLYP	Sappo-TZP/DZP	2415 [48]	389 [32]
B2PLYP	Sappo-QZP/DZP	2424 [20]	389 [31]
mPW2PLYP	def2-TZVP	2411 [34]	383 [32]
mPW2PLYP	def2-QZVP	2417 [26]	388 [30]
mPW2PLYP	LANL2DZ	2263 [61]	334 [23]
mPW2PLYP	LANL2TZ/def2-TZVP	2421 [22]	393 [15]
mPW2PLYP	ZORA-TZVP/TZVP	2443 [17]	391 [30]
mPW2PLYP	ZORA-TZVPP/TZVP	2451 [17]	394 [30]
mPW2PLYP	DKH-TZVP/TZVP	2455 [17]	385 [30]
mPW2PLYP	DKH-TZVPP/TZVP	2463 [17]	387 [31]
mPW2PLYP	Sappo-DZP/DZP	2440 [28]	374 [33]

to continued ...

Method	Basis set	PtH	PtCl
mPW2PLYP	Sappo-TZP/DZP	2414 [29]	387 [33]
mPW2PLYP	Sappo-QZP/DZP	2418 [26]	386 [32]
PBE0-DH	def2-TZVP	-	397 [18]
PBE0-DH	def2-QZVP	2424 [39]	397 [16]
PBE0-DH	LANL2DZ	2298 [76]	350 [17]
PBE0-DH	LANL2TZ/def2-TZVP	2439 [29]	407 [14]
PBE0-DH	ZORA-TZVP/TZVP	2444 [33]	397 [29]
PBE0-DH	ZORA-TZVPP/TZVP	2450 [33]	400 [30]
PBE0-DH	DKH-TZVP/TZVP	2456 [33]	396 [17]
PBE0-DH	DKH-TZVPP/TZVP	2461 [33]	396 [17]
PBE0-DH	Sappo-DZP/DZP	2437 [43]	387 [17]
PBE0-DH	Sappo-TZP/DZP	2415 [48]	395 [18]
PBE0-DH	Sappo-QZP/DZP	2415 [43]	393 [17]
PBE-QIDH	def2-TZVP	-	394 [37]
PBE-QIDH	def2-QZVP	2482 [35]	401 [35]
PBE-QIDH	LANL2DZ	2280 [97]	339 [27]
PBE-QIDH	LANL2TZ/def2-TZVP	2469 [32]	409 [19]
PBE-QIDH	ZORA-TZVP/TZVP	2500 [28]	400 [35]
PBE-QIDH	ZORA-TZVPP/TZVP	2508 [28]	403 [35]
PBE-QIDH	DKH-TZVP/TZVP	2512 [27]	394 [36]
PBE-QIDH	DKH-TZVPP/TZVP	2520 [28]	396 [36]
PBE-QIDH	Sappo-DZP/DZP	2503 [42]	381 [38]
PBE-QIDH	Sappo-TZP/DZP	2471 [44]	395 [37]
PBE-QIDH	Sappo-QZP/DZP	2474 [39]	395 [37]
DSD-BLYP	def2-TZVP	2511 [31]	393 [39]

to continued ...

Method	Basis set	PtH	PtCl
DSD-BLYP	def2-QZVP	2520 [20]	402 [37]
DSD-BLYP	LANL2DZ	2263 [94]	332 [26]
DSD-BLYP	LANL2TZ/def2-TZVP	2475 [23]	405 [19]
DSD-BLYP	ZORA-TZVP/TZVP	2540 [13]	402 [37]
DSD-BLYP	ZORA-TZVPP/TZVP	2551 [13]	406 [37]
DSD-BLYP	DKH-TZVP/TZVP	2553 [12]	395 [38]
DSD-BLYP	DKH-TZVPP/TZVP	2563 [13]	398 [38]
DSD-BLYP	Sappo-DZP/DZP	2550 [27]	380 [40]
DSD-BLYP	Sappo-TZP/DZP	2512 [26]	397 [40]
DSD-BLYP	Sappo-QZP/DZP	2518 [22]	398 [39]
RI-SCS-MP2	def2-TZVP	2618 [62]	388 [43]
RI-SCS-MP2	def2-QZVP	-	-
RI-SCS-MP2	LANL2DZ	2252 [212]	326 [24]
RI-SCS-MP2	LANL2TZ/def2-TZVP	2528 [36]	411 [26]
RI-SCS-MP2	ZORA-TZVP/TZVP	2628 [31]	395 [41]
RI-SCS-MP2	ZORA-TZVPP/TZVP	2640 [32]	399 [42]
RI-SCS-MP2	DKH-TZVP/TZVP	2642 [29]	389 [43]
RI-SCS-MP2	DKH-TZVPP/TZVP	2653 [30]	391 [44]
RI-SCS-MP2	Sappo-DZP/DZP	2653 [64]	372 [43]
RI-SCS-MP2	Sappo-TZP/DZP	2594 [56]	393 [44]
RI-SCS-MP2	Sappo-QZP/DZP	2605 [48]	395 [43]
HF	def2-TZVP	2149 [271]	328 [36]
HF	def2-QZVP	2131 [238]	325 [35]
HF	LANL2DZ	2078 [208]	309 [29]
HF	LANL2TZ/def2-TZVP	2313 [183]	357 [33]

to continued ...

Method	Basis set	PtH	PtCl
HF	ZORA-TZVP/TZVP	2166 [227]	329 [34]
HF	ZORA-TZVPP/TZVP	2167 [227]	329 [34]
HF	DKH-TZVP/TZVP	2173 [233]	325 [34]
HF	DKH-TZVPP/TZVP	2175 [232]	325 [34]
HF	Sappo-DZP/DZP	2149 [231]	324 [38]
HF	Sappo-TZP/DZP	2120 [240]	324 [37]
HF	Sappo-QZP/DZP	2120 [230]	323 [36]
BP86	def2-TZVP	2358 [7] MAD 19.5 cm^{-}	-
PBE	def2-TZVP	2361 [7] MAD 17.0 cm^{-}	-
B3P86	def2-TZVP	2381 [25] MAD 3.0 cm^{-}	-
B3PW91	def2-TZVP	2384 [27] MAD 6.0 cm^{-}	-
BP86	ZORA-TZVP/TZVP	-	398 [12] MAD 0.9 cm^{-}
PBE	ZORA-TZVP/TZVP	-	388 [17] MAD 11.6 cm^{-}
B3P86	ZORA-TZVP/TZVP	-	396 [20] MAD 2.9 cm^{-}
B3PW91	ZORA-TZVP/TZVP	-	391 [12] MAD 7.9 cm^{-}
HF-by-MMFF94	LANL2DZ/6-31G(d)	2066 [217] MAD 312.3 cm^{-}	311 [36] MAD 87.8 cm^{-}

end.

$[PtCl_4]^{2-}$

Table S6: Calculated wavenumbers (in cm^{-}) for Harmonic Vibrational Frequencies of $[PtCl_4]^{2-}$ with Infrared Intensity (in km/mol) in brackets. oop: A2u out-of-plane, bdd: Eu bending double degenerate, sdd: Eu stretching double degenerate. The level of theory used in the standard MMFF94 force field is included (HF-by-MMFF94). Abbreviated names for the basis sets are used here, with their full names listed in Table 1.

Method	Basis set	ν2 A2u oop	ν4 Eu bdd	ν7 Eu sdd
Experimental[7]	-	168	191	321
M06L	def2-TZVP	128.30 [6.82]	144.29 [0.01]	258.18 [108.00]
M06L	def2-QZVP	129.07 [7.69]	145.78 [0.01]	255.98 [105.70]
M06L	LANL2DZ	115.84 [7.62]	127.05 [0.24]	268.52 [90.04]
M06L	LANL2TZ/def2-TZVP	128.23 [6.09]	144.03 [0.01]	258.93 [101.49]
M06L	ZORA-TZVP/TZVP	129.12 [6.48]	145.23 [0.00]	258.20 [103.20]
M06L	ZORA-TZVPP/TZVP	129.24 [6.59]	145.30 [0.00]	257.57 [103.54]
M06L	DKH-TZVP/TZVP	129.82 [6.34]	145.93 [0.00]	256.83 [102.52]
M06L	DKH-TZVPP/TZVP	129.74 [6.45]	146.01 [0.00]	255.83 [102.83]
M06L	Sappo-DZP/DZP	130.29 [11.40]	141.59 [0.06]	260.44 [113.40]
M06L	Sappo-TZP/DZP	130.88 [10.48]	143.95 [0.03]	256.50 [109.77]
M06L	Sappo-QZP/DZP	130.23 [10.17]	143.25 [0.02]	255.05 [108.67]
B3LYP	def2-TZVP	129.70 [6.44]	146.08 [0.01]	267.12 [98.52]
B3LYP	def2-QZVP	130.11 [7.26]	146.47 [0.00]	263.95 [94.25]

to continued ...

Method	Basis set	$\nu 2$ A2u oop	$\nu 4$ Eu bdd	$\nu 7$ Eu sdd
B3LYP	LANL2DZ	118.35 [6.70]	130.34 [0.10]	277.72 [82.25]
B3LYP	LANL2TZ/def2-TZVP	129.32 [5.75]	145.35 [0.00]	265.45 [91.37]
B3LYP	ZORA-TZVP/TZVP	128.86 [6.27]	145.71 [0.00]	264.55 [93.79]
B3LYP	ZORA-TZVPP/TZVP	129.18 [6.38]	145.91 [0.00]	264.30 [94.01]
B3LYP	DKH-TZVP/TZVP	129.32 [6.17]	146.38 [0.01]	263.38 [92.93]
B3LYP	DKH-TZVPP/TZVP	129.65 [6.28]	146.63 [0.01]	262.95 [93.13]
B3LYP	Sappo-DZP/DZP	131.69 [9.87]	145.03 [0.03]	269.76 [99.95]
B3LYP	Sappo-TZP/DZP	132.54 [9.57]	146.56 [0.02]	266.15 [96.03]
B3LYP	Sappo-QZP/DZP	131.90 [9.23]	146.29 [0.01]	266.14 [94.35]
mPW1PW	def2-TZVP	134.60 [6.40]	151.45 [0.00]	286.08 [94.88]
mPW1PW	def2-QZVP	135.61 [7.16]	152.03 [0.00]	283.59 [89.03]
mPW1PW	LANL2DZ	122.56 [7.26]	134.62 [0.14]	295.35 [82.86]
mPW1PW	LANL2TZ/def2-TZVP	134.19 [5.68]	150.68 [0.00]	284.95 [88.51]
mPW1PW	ZORA-TZVP/TZVP	134.21 [6.37]	151.36 [0.00]	284.29 [90.17]
mPW1PW	ZORA-TZVPP/TZVP	134.58 [6.47]	151.59 [0.00]	284.13 [90.33]
mPW1PW	DKH-TZVP/TZVP	134.71 [6.25]	152.12 [0.00]	282.77 [89.19]
mPW1PW	DKH-TZVPP/TZVP	135.14 [6.35]	152.42 [0.00]	282.33 [89.31]
mPW1PW	Sappo-DZP/DZP	136.26 [9.59]	150.26 [0.04]	287.87 [97.60]

to continued ...

Method	Basis set	$\nu2$ A2u oop	$\nu4$ Eu bdd	$\nu7$ Eu sdd
mPW1PW	Sappo-TZP/DZP	137.18 [9.32]	151.77 [0.02]	284.64 [92.85]
mPW1PW	Sappo-QZP/DZP	136.63 [8.98]	151.46 [0.01]	283.93 [91.33]
PBE0	def2-TZVP	134.89 [6.23]	151.93 [0.01]	287.77 [94.03]
PBE0	def2-QZVP	135.94 [7.02]	152.59 [0.00]	285.31 [88.00]
PBE0	LANL2DZ	122.63 [7.11]	134.80 [0.13]	296.85 [82.63]
PBE0	LANL2TZ/def2-TZVP	134.56 [5.55]	151.17 [0.00]	286.76 [87.58]
PBE0	ZORA-TZVP/TZVP	134.44 [6.21]	151.77 [0.00]	285.87 [88.98]
PBE0	ZORA-TZVPP/TZVP	134.82 [6.32]	152.01 [0.00]	285.70 [89.13]
PBE0	DKH-TZVP/TZVP	134.96 [6.09]	152.55 [0.00]	284.30 [88.00]
PBE0	DKH-TZVPP/TZVP	135.39 [6.20]	152.85 [0.00]	283.86 [88.11]
PBE0	Sappo-DZP/DZP	136.56 [9.50]	150.60 [0.04]	289.23 [96.81]
PBE0	Sappo-TZP/DZP	137.55 [9.24]	152.14 [0.02]	286.06 [91.90]
PBE0	Sappo-QZP/DZP	136.98 [8.92]	151.82 [0.01]	285.21 [90.39]
TPSSH	def2-TZVP	132.00 [5.89]	146.86 [0.00]	282.20 [92.31]
TPSSH	def2-QZVP	132.89 [6.54]	147.48 [0.00]	280.54 [86.16]
TPSSH	LANL2DZ	120.00 [6.11]	130.74 [0.10]	291.82 [78.83]
TPSSH	LANL2TZ/def2-TZVP	131.39 [5.19]	145.77 [0.00]	280.34 [86.02]
TPSSH	ZORA-TZVP/TZVP	131.39 [5.89]	146.57 [0.00]	280.23 [88.51]

to continued ...

Method	Basis set	$\nu2$ A2u oop	$\nu4$ Eu bdd	$\nu7$ Eu sdd
TPSSH	ZORA-TZVPP/TZVP	131.74 [5.99]	146.78 [0.00]	280.07 [88.71]
TPSSH	DKH-TZVP/TZVP	131.89 [5.78]	147.32 [0.00]	278.78 [87.70]
TPSSH	DKH-TZVPP/TZVP	132.30 [5.88]	147.60 [0.00]	278.38 [87.86]
TPSSH	Sappo-DZP/DZP	133.87 [9.07]	145.94 [0.04]	284.78 [94.04]
TPSSH	Sappo-TZP/DZP	134.83 [8.84]	147.35 [0.02]	281.43 [90.14]
TPSSH	Sappo-QZP/DZP	134.24 [8.46]	147.08 [0.02]	280.70 [88.69]
M06	def2-TZVP	127.51 [6.52]	146.78 [0.01]	281.55 [99.83]
M06	def2-QZVP	127.96 [7.64]	147.23 [0.00]	278.32 [98.69]
M06	LANL2DZ	116.46 [7.48]	132.89 [0.11]	287.12 [82.26]
M06	LANL2TZ/def2-TZVP	126.59 [5.76]	145.83 [0.01]	279.50 [94.42]
M06	ZORA-TZVP/TZVP	127.65 [6.64]	147.10 [0.03]	281.01 [95.09]
M06	ZORA-TZVPP/TZVP	128.08 [6.76]	147.33 [0.03]	280.89 [95.19]
M06	DKH-TZVP/TZVP	128.60 [6.52]	147.87 [0.02]	280.00 [94.30]
M06	DKH-TZVPP/TZVP	128.37 [6.64]	148.01 [0.02]	279.40 [94.40]
M06	Sappo-DZP/DZP	130.46 [10.96]	146.42 [0.18]	285.07 [101.48]
M06	Sappo-TZP/DZP	130.33 [10.39]	147.47 [0.13]	282.96 [98.76]
M06	Sappo-QZP/DZP	129.99 [10.13]	147.22 [0.12]	283.01 [97.54]
M06-2X	def2-TZVP	127.84 [9.40]	144.32 [0.00]	240.85 [135.08]

to continued ...

Method	Basis set	ν2 A2u oop	ν4 Eu bdd	ν7 Eu sdd
M06-2X	def2-QZVP	128.45 [10,00]	144.29 [0.38]	230.82 [129.40]
M06-2X	LANL2DZ	120.74 [11.27]	132.12 [0.38]	277.04 [102.09]
M06-2X	LANL2TZ/def2-TZVP	128.13 [8.28]	144.72 [0.20]	241.76 [126.05]
M06-2X	ZORA-TZVP/TZVP	127.65 [9.11]	145.10 [0.20]	238.96 [124.57]
M06-2X	ZORA-TZVPP/TZVP	127.83 [9.25]	145.22 [0.22]	237.43 [125.19]
M06-2X	DKH-TZVP/TZVP	128.06 [9.02]	145.71 [0.23]	237.69 [123.21]
M06-2X	DKH-TZVPP/TZVP	128.28 [9.15]	145.87 [0.26]	235.97 [123.79]
M06-2X	Sappo-DZP/DZP	129.89 [13.61]	143.32 [0.00]	250.95 [135.37]
M06-2X	Sappo-TZP/DZP	130.44 [12.94]	144.70 [0.08]	238.26 [130.47]
M06-2X	Sappo-QZP/DZP	129.49 [12.86]	143.75 [0.06]	239.02 [127.88]
LC-BLYP	def2-TZVP	136.27 [6.32]	154.34 [0.00]	306.68 [96.18]
LC-BLYP	def2-QZVP	136.80 [7.35]	154.61 [0.00]	303.40 [90.70]
LC-BLYP	LANL2DZ	123.81 [7.18]	137.99 [0.18]	315.08 [83.22]
LC-BLYP	LANL2TZ/def2-TZVP	136.19 [5.68]	153.61 [0.00]	305.55 [89.46]
LC-BLYP	ZORA-TZVP/TZVP	135.48 [6.19]	154.18 [0.00]	305.10 [90.07]
LC-BLYP	ZORA-TZVPP/TZVP	135.94 [6.32]	154.44 [0.00]	304.91 [90.20]
LC-BLYP	DKH-TZVP/TZVP	136.00 [6.08]	155.01 [0.00]	303.36 [88.94]
LC-BLYP	DKH-TZVPP/TZVP	136.52 [6.20]	155.33 [0.00]	302.90 [89.04]

to continued ...

Method	Basis set	$\nu2$ A2u oop	$\nu4$ Eu bdd	$\nu7$ Eu sdd
LC-BLYP	Sappo-DZP/DZP	139.00 [9.57]	153.46 [0.07]	310.42 [96.07]
LC-BLYP	Sappo-TZP/DZP	139.45 [9.31]	154.86 [0.05]	307.40 [90.89]
LC-BLYP	Sappo-QZP/DZP	138.96 [9.04]	154.60 [0.04]	306.73 [89.32]
CAM-B3LYP	def2-TZVP	133.76 [7.20]	151.57 [0.00]	285.69 [102.05]
CAM-B3LYP	def2-QZVP	134.53 [8.12]	151.84 [0.00]	282.19 [97.28]
CAM-B3LYP	LANL2DZ	122.19 [8.27]	135.56 [0.21]	295.39 [87.26]
CAM-B3LYP	LANL2TZ/def2-TZVP	133.77 [6.53]	150.89 [0.00]	284.02 [95.41]
CAM-B3LYP	ZORA-TZVP/TZVP	133.33 [7.02]	151.35 [0.00]	283.36 [96.40]
CAM-B3LYP	ZORA-TZVPP/TZVP	133.69 [7.14]	151.56 [0.00]	283.12 [96.56]
CAM-B3LYP	DKH-TZVP/TZVP	133.79 [6.90]	152.07 [0.00]	282.01 [95.33]
CAM-B3LYP	DKH-TZVPP/TZVP	134.20 [7.02]	152.34 [0.00]	281.54 [95.46]
CAM-B3LYP	Sappo-DZP/DZP	136.04 [10.60]	150.47 [0.06]	288.73 [103.34]
CAM-B3LYP	Sappo-TZP/DZP	136.65 [10.26]	151.87 [0.04]	285.14 [98.48]
CAM-B3LYP	Sappo-QZP/DZP	136.12 [9.95]	151.63 [0.04]	284.82 [96.85]
ωB97X-D3BJ	def2-TZVP	135.79 [7.58]	154.71 [0.00]	300.72 [103.75]
ωB97X-D3BJ	def2-QZVP	136.69 [8.49]	155.15 [0.00]	297.80 [97.92]
ωB97X-D3BJ	LANL2DZ	124.04 [9.69]	138.39 [0.30]	311.10 [91.50]
ωB97X-D3BJ	LANL2TZ/def2-TZVP	135.68 [6.95]	154.13 [0.00]	299.68 [97.66]

to continued ...

Method	Basis set	$\nu2$ A2u oop	$\nu4$ Eu bdd	$\nu7$ Eu sdd
ωB97X-D3BJ	ZORA-TZVP/TZVP	135.34 [7.42]	154.62 [0.01]	299.10 [97.20]
ωB97X-D3BJ	ZORA-TZVPP/TZVP	135.71 [7.54]	154.84 [0.01]	298.88 [97.29]
ωB97X-D3BJ	DKH-TZVP/TZVP	135.84 [7.29]	155.36 [0.00]	297.58 [95.97]
ωB97X-D3BJ	DKH-TZVPP/TZVP	136.28 [7.41]	155.65 [0.00]	297.10 [96.02]
ωB97X-D3BJ	Sappo-DZP/DZP	137.73 [11.16]	153.33 [0.08]	303.73 [106.35]
ωB97X-D3BJ	Sappo-TZP/DZP	138.15 [10.71]	154.60 [0.06]	299.78 [100.49]
ωB97X-D3BJ	Sappo-QZP/DZP	137.61 [10.40]	154.26 [0.05]	298.93 [99.03]
B97-3c	def2-TZVP	129.12 [4.75]	148.97 [0.03]	267.40 [87.50]
B97-3c	def2-QZVP	130.03 [5.28]	149.95 [0.03]	265.73 [81.88]
B97-3c	LANL2DZ	117.66 [4.78]	131.22 [0.03]	277.94 [75.35]
B97-3c	LANL2TZ/def2-TZVP	129.03 [4.09]	148.17 [0.02]	267.04 [80.41]
B97-3c	ZORA-TZVP/TZVP	129.19 [4.72]	149.40 [0.01]	266.22 [83.29]
B97-3c	ZORA-TZVPP/TZVP	129.58 [4.82]	149.64 [0.02]	265.98 [83.47]
B97-3c	DKH-TZVP/TZVP	129.79 [4.62]	150.80 [0.02]	264.34 [82.55]
B97-3c	DKH-TZVPP/TZVP	130.23 [4.71]	150.59 [0.03]	263.85 [82.69]
B97-3c	Sappo-DZP/DZP	-	-	-
B97-3c	Sappo-TZP/DZP	-	-	-
B97-3c	Sappo-QZP/DZP	-	-	-

to continued ...

Method	Basis set	$\nu 2$ A2u oop	$\nu 4$ Eu bdd	$\nu 7$ Eu sdd
B2PLYP	def2-TZVP	131.77 [4.01]	156.48 [0.12]	285.75 [97.86]
B2PLYP	def2-QZVP	130.70 [4.05]	164.56 [0.14]	282.05 [84.86]
B2PLYP	LANL2DZ	122.34 [7.71]	133.76 [0.11]	285.54 [86.39]
B2PLYP	LANL2TZ/def2-TZVP	130.68 [5.51]	153.87 [0.04]	276.12 [90.54]
B2PLYP	ZORA-TZVP/TZVP	128.60 [5.61]	157.19 [0.07]	283.08 [94.39]
B2PLYP	ZORA-TZVPP/TZVP	128.62 [5.71]	157.38 [0.07]	283.49 [94.49]
B2PLYP	DKH-TZVP/TZVP	129.20 [5.51]	157.86 [0.08]	281.80 [93.37]
B2PLYP	DKH-TZVPP/TZVP	129.98 [5.70]	158.17 [0.08]	281.87 [93.46]
B2PLYP	Sappo-DZP/DZP	138.24 [9.41]	150.56 [0.00]	283.92 [103.18]
B2PLYP	Sappo-TZP/DZP	140.26 [8.93]	151.98 [0.00]	282.80 [98.29]
B2PLYP	Sappo-QZP/DZP	139.98 [8.32]	152.62 [0.00]	283.25 [96.03]
mPW2PLYP	def2-TZVP	133.90 [5.36]	157.60 [0.10]	287.36 [99.19]
mPW2PLYP	def2-QZVP	131.39 [4.54]	165.79 [0.12]	283.48 [68.39]
mPW2PLYP	LANL2DZ	122.38 [8.01]	134.70 [0.15]	288.24 [87.28]
mPW2PLYP	LANL2TZ/def2-TZVP	132.64 [5.76]	155.15 [0.03]	278.21 [93.59]
mPW2PLYP	ZORA-TZVP/TZVP	129.04 [5.95]	158.32 [0.05]	284.68 [95.49]
mPW2PLYP	ZORA-TZVPP/TZVP	129.03 [5.83]	158.50 [0.05]	285.00 [95.57]
mPW2PLYP	DKH-TZVP/TZVP	131.04 [6.30]	159.04 [0.06]	283.39 [94.44]

to continued ...

Method	Basis set	$\nu2$ A2u oop	$\nu4$ Eu bdd	$\nu7$ Eu sdd
mPW2PLYP	DKH-TZVPP/TZVP	130.12 [6.18]	159.30 [0.06]	283.34 [94.54]
mPW2PLYP	Sappo-DZP/DZP	140.14 [9.59]	151.42 [0.01]	285.82 [104.44]
mPW2PLYP	Sappo-TZP/DZP	136.83 [9.35]	152.73 [0.00]	284.55 [99.42]
mPW2PLYP	Sappo-QZP/DZP	140.21 [8.80]	153.60 [0.00]	284.91 [97.22]
PBE0-DH	def2-TZVP	136.58 [4.91]	161.74 [0.02]	298.90 [96.95]
PBE0-DH	def2-QZVP	135.83 [4.91]	169.36 [0.04]	296.57 [90.35]
PBE0-DH	LANL2DZ	127.25 [8.93]	138.49 [0.23]	302.87 [87.73]
PBE0-DH	LANL2TZ/def2-TZVP	138.15 [5.79]	160.23 [0.02]	294.72 [90.88]
PBE0-DH	ZORA-TZVP/TZVP	133.46 [6.63]	162.11 [0.01]	297.35 [91.73]
PBE0-DH	ZORA-TZVPP/TZVP	133.38 [6.73]	162.14 [0.01]	297.44 [91.79]
PBE0-DH	DKH-TZVP/TZVP	133.92 [6.50]	162.81 [0.01]	295.66 [90.78]
PBE0-DH	DKH-TZVPP/TZVP	134.04 [6.61]	162.90 [0.01]	295.42 [90.77]
PBE0-DH	Sappo-DZP/DZP	140.21 [10.16]	155.66 [0.02]	298.70 [101.57]
PBE0-DH	Sappo-TZP/DZP	140.43 [9.70]	156.40 [0.02]	296.60 [95.92]
PBE0-DH	Sappo-QZP/DZP	143.29 [9.10]	156.82 [0.01]	296.03 [94.06]
PBE-QIDH	def2-TZVP	140.61 [5.81]	166.30 [0.08]	307.07 [98.03]
PBE-QIDH	def2-QZVP	141.47 [5.50]	176.92 [0.16]	304.95 [90.37]
PBE-QIDH	LANL2DZ	125.85 [9.31]	139.92 [0.22]	303.47 [90.27]

to continued ...

Method	Basis set	$\nu2$ A2u oop	$\nu4$ Eu bdd	$\nu7$ Eu sdd
PBE-QIDH	LANL2TZ/def2-TZVP	137.60 [5.36]	163.14 [0.03]	297.21 [92.50]
PBE-QIDH	ZORA-TZVP/TZVP	133.98 [6.12]	166.87 [0.05]	305.39 [92.91]
PBE-QIDH	ZORA-TZVPP/TZVP	133.95 [6.10]	167.09 [0.04]	305.96 [92.90]
PBE-QIDH	DKH-TZVP/TZVP	134.36 [6.11]	167.62 [0.06]	303.66 [91.72]
PBE-QIDH	DKH-TZVPP/TZVP	134.56 [6.10]	167.96 [0.06]	303.86 [91.71]
PBE-QIDH	Sappo-DZP/DZP	142.89 [10.01]	158.57 [0.00]	304.42 [103.18]
PBE-QIDH	Sappo-TZP/DZP	146.97 [9.10]	159.69 [0.00]	304.00 [97.67]
PBE-QIDH	Sappo-QZP/DZP	146.35 [8.74]	160.68 [0.00]	304.16 [95.38]
DSD-BLYP	def2-TZVP	137.41 [5.62]	164.69 [0.16]	301.43 [100.85]
DSD-BLYP	def2-QZVP	139.98 [5.35]	176.62 [0.34]	299.94 [92.33]
DSD-BLYP	LANL2DZ	123.15 [8.56]	137.61 [0.12]	293.75 [91.39]
DSD-BLYP	LANL2TZ/def2-TZVP	132.78 [5.50]	160.22 [0.09]	287.77 [93.73]
DSD-BLYP	ZORA-TZVP/TZVP	131.63 [5.28]	165.74 [0.15]	300.78 [95.04]
DSD-BLYP	ZORA-TZVPP/TZVP	132.50 [5.19]	166.10 [0.15]	301.80 [95.04]
DSD-BLYP	DKH-TZVP/TZVP	132.05 [5.15]	166.30 [0.16]	299.21 [93.72]
DSD-BLYP	DKH-TZVPP/TZVP	133.27 [5.33]	166.95 [0.17]	299.84 [93.82]
DSD-BLYP	Sappo-DZP/DZP	143.27 [9.25]	156.66 [0.02]	297.52 [106.12]
DSD-BLYP	Sappo-TZP/DZP	141.74 [8.58]	158.63 [0.01]	299.10 [100.11]

to continued ...

Method	Basis set	$\nu 2$ A2u oop	$\nu 4$ Eu bdd	$\nu 7$ Eu sdd
DSD-BLYP	Sappo-QZP/DZP	142.04 [8.15]	160.23 [0.04]	299.99 [97.45]
RI-SCS-MP2	def2-TZVP	139.29 [5.61]	169.68 [0.18]	302.61 [109.10]
RI-SCS-MP2	def2-QZVP	-	-	-
RI-SCS-MP2	LANL2DZ	123.71 [9.43]	139.86 [0.12]	291.31 [97.50]
RI-SCS-MP2	LANL2TZ/def2-TZVP	131.84 [5.83]	162.92 [0.26]	281.65 [102.01]
RI-SCS-MP2	ZORA-TZVP/TZVP	132.05 [5.27]	170.97 [0.37]	301.79 [102.62]
RI-SCS-MP2	ZORA-TZVPP/TZVP	132.04 [5.39]	171.57 [0.38]	303.43 [102.65]
RI-SCS-MP2	DKH-TZVP/TZVP	132.36 [5.15]	171.65 [0.39]	300.45 [101.13]
RI-SCS-MP2	DKH-TZVPP/TZVP	132.61 [5.26]	172.47 [0.41]	301.66 [101.07]
RI-SCS-MP2	Sappo-DZP/DZP	143.75 [9.68]	159.22 [0.11]	296.62 [115.97]
RI-SCS-MP2	Sappo-TZP/DZP	143.65 [8.55]	161.90 [0.07]	300.47 [108.52]
RI-SCS-MP2	Sappo-QZP/DZP	148.60 [8.02]	164.56 [0.12]	302.28 [105.31]
HF	def2-TZVP	136.17 [14.32]	155.95 [0.20]	263.41 [128.14]
HF	def2-QZVP	136.68 [14.89]	155.50 [0.18]	256.99 [123.91]
HF	LANL2DZ	125.62 [19.85]	141.89 [2.07]	275.81 [105.30]
HF	LANL2TZ/def2-TZVP	136.13 [13.51]	155.30 [0.17]	257.74 [122.21]
HF	ZORA-TZVP/TZVP	135.93 [14.02]	155.43 [0.21]	258.55 [121.82]
HF	ZORA-TZVPP/TZVP	136.16 [14.17]	155.56 [0.21]	258.16 [121.87]

to continued ...

Method	Basis set	$\nu2$ A2u oop	$\nu4$ Eu bdd	$\nu7$ Eu sdd
HF	DKH-TZVP/TZVP	136.16 [13.89]	155.86 [0.20]	257.99 [120.62]
HF	DKH-TZVPP/TZVP	136.43 [14.04]	156.03 [0.19]	257.43 [120.65]
HF	Sappo-DZP/DZP	136.59 [18.78]	153.65 [0.54]	265.39 [134.30]
HF	Sappo-TZP/DZP	136.79 [17.95]	154.48 [0.43]	260.34 [128.06]
HF	Sappo-QZP/DZP	136.57 [17.52]	154.48 [0.42]	260.50 [126.33]
BP86	DKH-TZVPP/TZVP	128.64 [4.52]	144.76 [0.01]	267.35 [81.63] MAD 46.4 cm^{-}
PBE	DKH-TZVPP/TZVP	129.15 [4.47]	145.62 [0.01]	269.48 [80.42] MAD 45.25 cm^{-}
B3P86	DKH-TZVPP/TZVP	133.52 [5.91]	150.71 [0.00]	278.85 [88.29] MAD 38.97 cm^{-}
B3PW91	DKH-TZVPP/TZVP	133.80 [6.10]	150.79 [0.00]	277.35 [88.80] MAD 39.35 cm^{-}
HF-by-MMFF94	LANL2DZ/6-31G(d)	130.36 [16.62]	146.64 [0.56]	259.82 [122.51] MAD 47.73 cm^{-}

end.

$[Pt(NH_3)_4]^{2+}$

Table S7: Calculated wavenumbers (in cm^{-1}) for Harmonic Vibrational Frequencies of $[Pt(NH_3)_4]^{2+}$ complex with IR Intensities (in km/mol) in brackets. ρ: rocking, δ_s: symmetric deformation, δ_a: asymmetric deformation, ν_s: symmetric stretching, ν_a: asymmetric stretching. The level of theory used in the standard MMFF94 force field is included (HF-by-MMFF94). Abbreviated names for the basis sets are used here, with their full names listed in Table 1.

Method	Basis set	ρNH_3	$\delta_s NH_3$	$\delta_a NH_3$	ν_s(NH)	ν_a(NH)
Experimental	-	824.50	1313.60	1580.00	3174.00	3298.50
M06L	def2-TZVP	-	-	-	-	-
M06L	def2-QZVP	-	-	-	-	-
M06L	LANL2DZ	-	-	-	-	-
M06L	LANL2TZ/def2-TZVP	826.50	1394.37	1663.16	3418.44	3512.98
		[45.23]	[123.74]	[127.35]	[99.74]	[105.91]
M06L	ZORA-TZVP/TZVP	821.91	1399.65	1664.57	3419.12	3512.07
		[40.04]	[133.52]	[133.02]	[93.95]	[148.14]
M06L	ZORA-TZVPP/TZVP	821.73	1400.24	1665.61	3418.81	3511.32
		[43.02]	[133.66]	[132.27]	[93.16]	[147.95]
M06L	DKH-TZVP/TZVP	-	-	-	-	-
M06L	DKH-TZVPP/TZVP	-	-	-	-	-
M06L	Sappo-DZP/DZP	-	-	-	-	-
M06L	Sappo-TZP/DZP	-	-	-	-	-

to continued ...

Method	Basis set	ρNH_3	$\delta_s NH_3$	$\delta_a NH_3$	ν_s(NH)	ν_a(NH)
M06L	Sappo-QZP/DZP	-	-	-	-	-
B3LYP	def2-TZVP	831.35	1400.57	1658.83	3404.14	3480.77
		[51.80]	[177.65]	[150.50]	[113.43]	[243.19]
B3LYP	def2-QZVP	827.76	1399.13	1659.88	3412.52	3490.01
		[43.65]	[167.47]	[137.62]	[118.08]	[296.74]
B3LYP	LANL2DZ	885.97	1444.44	1709.57	3402.20	3517.08
		[65.94]	[317.92]	[215.07]	[73.72]	[169.61]
B3LYP	LANL2TZ/def2-TZVP	839.20	1402.61	1658.28	3403.54	3479.78
		[51.14]	[177.14]	[152.78]	[112.84]	[238.13]
B3LYP	ZORA-TZVP/TZVP	834.43	1402.72	1659.07	3402.82	3478.82
		[46.95]	[174.87]	[150.80]	[111.97]	[155.41]
B3LYP	ZORA-TZVPP/TZVP	834.13	1403.26	1659.25	3402.68	3478.46
		[47.21]	[175.25]	[150.67]	[111.24]	[173.43]
B3LYP	DKH-TZVP/TZVP	-	-	-	-	-
B3LYP	DKH-TZVPP/TZVP	-	-	-	-	-
B3LYP	Sappo-DZP/DZP	822.01	1376.55	1632.07	3380.99	3467.82
		[64.43]	[133.14]	[133.82]	[128.18]	[356.82]
B3LYP	Sappo-TZP/DZP	820.04	1379.13	1630.65	3381.81	3467.43

to continued ...

Method	Basis set	ρNH_3	$\delta_s NH_3$	$\delta_a NH_3$	ν_s(NH)	ν_a(NH)
		[51.12]	[139.02]	[131.63]	[123.29]	[333.95]
B3LYP	Sappo-QZP/DZP	821.63	1380.01	1629.96	3382.77	3468.60
		[49.83]	[140.04]	[131.69]	[122.51]	[333.93]
mPW1PW	def2-TZVP	842.59	1409.04	1663.44	3448.86	3531.61
		[54.01]	[189.22]	[152.38]	[116.54]	[212.38]
mPW1PW	def2-QZVP	837.89	1406.41	1663.64	3453.73	3537.83
		[44.85]	[179.19]	[140.24]	[120.38]	[194.09]
mPW1PW	LANL2DZ	902.72	1462.22	1726.16	3448.24	3569.65
		[66.36]	[339.46]	[223.24]	[74.37]	[337.36]
mPW1PW	LANL2TZ/def2-TZVP	850.23	1410.75	1662.84	3447.73	3530.17
		[52.41]	[188.90]	[153.86]	[114.40]	[326.32]
mPW1PW	ZORA-TZVP/TZVP	844.03	1410.26	1663.94	3447.51	3529.62
		[48.30]	[187.35]	[153.24]	[114.93]	[319.10]
mPW1PW	ZORA-TZVPP/TZVP	843.82	1410.87	1664.11	3447.32	3529.20
		[48.79]	[187.76]	[153.09]	[114.21]	[315.29]
mPW1PW	DKH-TZVP/TZVP	-	-	-	-	-
mPW1PW	DKH-TZVPP/TZVP	-	-	-	-	-
mPW1PW	Sappo-DZP/DZP	833.00	1388.11	1641.07	3423.23	3519.52

to continued ...

Method	Basis set	ρNH_3	$\delta_s NH_3$	$\delta_a NH_3$	$\nu_s(NH)$	$\nu_a(NH)$
		[60.44]	[146.95]	[139.92]	[132.64]	[362.82]
mPW1PW	Sappo-TZP/DZP	830.87	1390.88	1640.04	3423.95	3519.03
		[53.20]	[152.80]	[137.42]	[127.33]	[362.59]
mPW1PW	Sappo-QZP/DZP	832.50	1391.49	1639.68	3424.60	3520.20
		[51.38]	[153.32]	[137.54]	[125.62]	[314.03]
PBE0	def2-TZVP	840.77	1404.43	1657.13	3440.61	3524.01
		[53.34]	[190.08]	[153.51]	[117.94]	[318.33]
PBE0	def2-QZVP	836.17	1401.52	1657.05	3446.60	3531.56
		[44.36]	[179.75]	[140.56]	[120.96]	[159.83]
PBE0	LANL2DZ	901.72	1459.04	1720.97	3440.22	3561.34
		[65.48]	[342.60]	[224.77]	[75.79]	[347.29]
PBE0	LANL2TZ/def2-TZVP	848.55	1406.34	1656.53	3439.84	3522.88
		[52.06]	[190.30]	[154.74]	[115.29]	[318.17]
PBE0	ZORA-TZVP/TZVP	842.13	1405.79	1657.59	3439.64	3522.36
		[48.21]	[188.53]	[153.75]	[115.80]	[305.33]
PBE0	ZORA-TZVPP/TZVP	841.95	1406.41	1657.76	3439.43	3521.93
		[48.60]	[188.90]	[153.61]	[115.03]	[302.06]
PBE0	DKH-TZVP/TZVP	-	-	-	-	-

to continued ...

Method	Basis set	ρNH_3	$\delta_s NH_3$	$\delta_a NH_3$	ν_s(NH)	ν_a(NH)
PBE0	DKH-TZVPP/TZVP	-	-	-	-	-
PBE0	Sappo-DZP/DZP	831.47	1384.32	1635.49	3418.41	3515.51
		[66.50]	[149.06]	[141.16]	[134.46]	[329.23]
PBE0	Sappo-TZP/DZP	829.39	1387.05	1634.44	3419.27	3514.74
		[52.89]	[154.98]	[138.51]	[129.22]	[375.73]
PBE0	Sappo-QZP/DZP	830.52	1387.43	1633.88	3420.12	3515.69
		[51.36]	[155.75]	[138.62]	[128.12]	[352.96]
TPSSh	def2-TZVP	837.10	1409.20	1668.94	3395.19	3474.84
		[46.97]	[157.11]	[142.50]	[101.94]	[159.71]
TPSSh	def2-QZVP	831.92	1405.46	1668.90	3397.74	3478.23
		[38.01]	[148.93]	[131.90]	[106.35]	[200.89]
TPSSh	LANL2DZ	891.10	1451.94	1722.07	3396.26	3513.86
		[57.42]	[284.47]	[203.61]	[64.54]	[150.16]
TPSSh	LANL2TZ/def2-TZVP	844.40	1410.26	1668.16	3394.79	3474.17
		[45.06]	[156.82]	[144.36]	[101.53]	[169.93]
TPSSh	ZORA-TZVP/TZVP	838.17	1409.04	1669.12	3394.12	3473.35
		[42.13]	[154.95]	[143.50]	[101.93]	[220.54]
TPSSh	ZORA-TZVPP/TZVP	838.06	1409.72	1669.26	3393.98	3472.96

to continued ...

Method	Basis set	ρNH_3	$\delta_s NH_3$	$\delta_a NH_3$	ν_s(NH)	ν_a(NH)
		[42.16]	[155.10]	[142.91]	[101.06]	[181.13]
TPSSh	DKH-TZVP/TZVP	-	-	-	-	-
TPSSh	DKH-TZVPP/TZVP	-	-	-	-	-
TPSSh	Sappo-DZP/DZP	825.32	1385.25	1645.91	3357.97	3449.94
		[58.36]	[114.80]	[129.48]	[114.93]	[328.20]
TPSSh	Sappo-TZP/DZP	823.28	1387.52	1644.90	3359.00	3449.93
		[44.81]	[120.57]	[127.12]	[109.21]	[284.11]
TPSSh	Sappo-QZP/DZP	824.53	1387.92	1644.26	3359.83	3450.94
		[43.20]	[121.57]	[127.24]	[108.48]	[284.45]
M06	def2-TZVP	804.55	1371.10	1625.56	3407.80	3501.31
		[40.90]	[190.05]	[119.40]	[128.94]	[250.78]
M06	def2-QZVP	-	-	-	-	-
M06	LANL2DZ	877.87	1431.63	1713.26	3410.67	3548.78
		[67.04]	[345.23]	[166.31]	[84.86]	[222.44]
M06	LANL2TZ/def2-TZVP	810.82	1369.61	1623.27	3406.54	3499.92
		[47.50]	[186.94]	[119.87]	[127.72]	[156.44]
M06	ZORA-TZVP/TZVP	803.41	1375.42	1629.04	3409.85	3500.76
		[50.44]	[206.80]	[150.93]	[122.71]	[274.12]

to continued ...

Method	Basis set	ρNH_3	$\delta_s NH_3$	$\delta_a NH_3$	ν_s(NH)	ν_a(NH)
M06	ZORA-TZVPP/TZVP	802.85	1376.04	1628.15	3409.50	3500.01
		[44.30]	[207.50]	[150.53]	[122.51]	[259.94]
M06	DKH-TZVP/TZVP	-	-	-	-	-
M06	DKH-TZVPP/TZVP	-	-	-	-	-
M06	Sappo-DZP/DZP	796.55	1358.71	1606.48	3394.04	3495.11
		[74.63]	[169.36]	[142.90]	[144.64]	[351.01]
M06	Sappo-TZP/DZP	791.33	1358.94	1604.69	3394.87	3495.24
		[54.23]	[175.44]	[140.57]	[140.29]	[347.45]
M06	Sappo-QZP/DZP	793.30	1360.13	1604.75	3395.90	3496.53
		[53.26]	[176.03]	[140.24]	[139.09]	[384.82]
M06-2X	def2-TZVP	824.04	1407.81	1653.22	3433.66	3519.84
		[58.82]	[224.27]	[156.06]	[149.01]	[310.17]
M06-2X	def2-QZVP	818.54	1404.11	1654.54	3442.53	3527.37
		[47.29]	[211.80]	[142.08]	[155.96]	[333.49]
M06-2X	LANL2DZ	882.29	1460.79	1713.55	3434.23	3554.28
		[74.51]	[420.91]	[244.83]	[101.84]	[391.83]
M06-2X	LANL2TZ/def2-TZVP	834.41	1409.92	1652.76	3432.71	3518.63
		[53.91]	[222.57]	[156.91]	[147.32]	[369.37]

to continued ...

Method	Basis set	ρNH_3	$\delta_s NH_3$	$\delta_a NH_3$	ν_s(NH)	ν_a(NH)
M06-2X	ZORA-TZVP/TZVP	825.78	1408.71	1653.71	3432.39	3517.96
		[50.50]	[220.61]	[156.85]	[148.05]	[336.49]
M06-2X	ZORA-TZVPP/TZVP	825.40	1409.35	1653.99	3432.46	3518.02
		[49.78]	[220.96]	[155.59]	[147.37]	[335.77]
M06-2X	DKH-TZVP/TZVP	-	-	-	-	-
M06-2X	DKH-TZVPP/TZVP	-	-	-	-	-
M06-2X	Sappo-DZP/DZP	812.01	1384.02	1630.27	3408.01	3501.03
		[73.25]	[181.32]	[144.92]	[163.59]	[414.90]
M06-2X	Sappo-TZP/DZP	812.22	1386.16	1628.92	3408.83	3500.75
		[58.22]	[185.27]	[140.83]	[156.88]	[381.88]
M06-2X	Sappo-QZP/DZP	-	-	-	-	-
LC-BLYP	def2-TZVP	828.55	1390.02	1620.07	3377.17	3450.05
		[53.98]	[247.35]	[165.50]	[136.98]	[310.37]
LC-BLYP	def2-QZVP	823.12	1386.66	1620.51	3384.90	3458.39
		[46.21]	[232.39]	[150.60]	[142.54]	[352.46]
LC-BLYP	LANL2DZ	889.00	1442.64	1677.45	3386.44	3492.37
		[69.52]	[430.94]	[240.31]	[92.49]	[362.79]
LC-BLYP	LANL2TZ/def2-TZVP	836.01	1391.36	1619.39	3376.63	3448.67

to continued ...

Method	Basis set	ρNH_3	$\delta_s NH_3$	$\delta_a NH_3$	$\nu_s(NH)$	$\nu_a(NH)$
		[51.52]	[246.99]	[167.32]	[136.31]	[328.21]
LC-BLYP	ZORA-TZVP/TZVP	830.53	1390.67	1620.25	3376.46	3448.40
		[49.24]	[241.82]	[165.44]	[136.38]	[381.22]
LC-BLYP	ZORA-TZVPP/TZVP	830.10	1391.28	1620.59	3376.23	3448.69
		[49.28]	[242.11]	[164.97]	[133.89]	[282.33]
LC-BLYP	DKH-TZVP/TZVP	-	-	-	-	-
LC-BLYP	DKH-TZVPP/TZVP	-	-	-	-	-
LC-BLYP	Sappo-DZP/DZP	819.43	1364.47	1592.98	3354.30	3436.74
		[66.53]	[188.90]	[145.67]	[151.45]	[411.81]
LC-BLYP	Sappo-TZP/DZP	815.87	1365.35	1591.10	3355.61	3437.27
		[52.80]	[194.84]	[142.92]	[147.74]	[368.65]
LC-BLYP	Sappo-QZP/DZP	817.17	1366.04	1590.51	3356.84	3438.34
		[51.47]	[195.75]	[142.87]	[147.19]	[371.11]
CAM-B3LYP	def2-TZVP	838.39	1415.36	1661.45	3432.13	3507.78
		[56.32]	[220.47]	[158.40]	[125.52]	[210.94]
CAM-B3LYP	def2-QZVP	833.94	1413.18	1662.43	3440.42	3516.80
		[48.09]	[207.88]	[144.51]	[130.17]	[261.92]
CAM-B3LYP	LANL2DZ	896.28	1464.12	1715.62	3436.02	3547.10

to continued ...

Method	Basis set	ρNH_3	$\delta_s NH_3$	$\delta_a NH_3$	ν_s(NH)	ν_a(NH)
		[71.92]	[385.62]	[229.90]	[83.35]	[343.33]
CAM-B3LYP	LANL2TZ/def2-TZVP	846.37	1417.18	1661.01	3431.72	3506.35
		[55.19]	[219.18]	[160.33]	[124.98]	[347.39]
CAM-B3LYP	ZORA-TZVP/TZVP	841.00	1416.81	1661.86	3431.34	3505.93
		[40.41]	[216.03]	[158.46]	[125.22]	[340.56]
CAM-B3LYP	ZORA-TZVPP/TZVP	840.61	1417.31	1662.01	3431.18	3505.57
		[49.47]	[216.51]	[158.30]	[124.44]	[338.97]
CAM-B3LYP	DKH-TZVP/TZVP	-	-	-	-	-
CAM-B3LYP	DKH-TZVPP/TZVP	-	-	-	-	-
CAM-B3LYP	Sappo-DZP/DZP	829.16	1391.27	1635.57	3411.11	3496.79
		[69.01]	[169.89]	[141.52]	[142.26]	[387.04]
CAM-B3LYP	Sappo-TZP/DZP	826.44	1393.12	1633.97	3412.06	3496.62
		[55.17]	[175.63]	[139.05]	[138.13]	[372.73]
CAM-B3LYP	Sappo-QZP/DZP	827.91	1393.90	1633.35	3413.04	3497.77
		[53.80]	[176.48]	[139.13]	[137.19]	[367.78]
ωB97X-D3BJ	def2-TZVP	839.81	1417.94	1666.15	3454.00	3534.82
		[56.93]	[215.73]	[151.18]	[115.63]	[172.63]
ωB97X-D3BJ	def2-QZVP	834.95	1415.33	1666.67	3461.34	3542.37

to continued ...

Method	Basis set	ρNH_3	$\delta_s NH_3$	$\delta_a NH_3$	$\nu_s(NH)$	$\nu_a(NH)$
		[47.87]	[204.08]	[138.97]	[119.45]	[301.29]
ωB97X-D3BJ	LANL2DZ	898.94	1470.59	1726.26	3458.76	3574.62
		[71.32]	[380.37]	[224.35]	[72.08]	[159.62]
ωB97X-D3BJ	LANL2TZ/def2-TZVP	847.23	1419.53	1665.50	3453.63	3533.23
		[54.93]	[216.11]	[153.06]	[113.62]	[322.49]
ωB97X-D3BJ	ZORA-TZVP/TZVP	840.86	1418.79	1666.48	3453.52	3533.22
		[51.59]	[213.23]	[152.19]	[114.41]	[314.53]
ωB97X-D3BJ	ZORA-TZVPP/TZVP	840.78	1419.30	1666.90	3453.05	3533.29
		[51.47]	[213.54]	[151.42]	[113.23]	[179.86]
ωB97X-D3BJ	DKH-TZVP/TZVP	-	-	-	-	-
ωB97X-D3BJ	DKH-TZVPP/TZVP	-	-	-	-	-
ωB97X-D3BJ	Sappo-DZP/DZP	829.97	1396.51	1643.39	3432.83	3526.28
		[69.65]	[169.79]	[138.68]	[133.37]	[371.09]
ωB97X-D3BJ	Sappo-TZP/DZP	-	-	-	-	-
ωB97X-D3BJ	Sappo-QZP/DZP	828.81	1398.87	1641.68	3434.88	3527.56
		[54.07]	[176.01]	[136.1]	[127.44]	[352.40]
B97-3c	def2-TZVP	833.42	1382.63	1650.29	3382.25	3472.72
		[44.01]	[146.70]	[143.59]	[98.20]	[274.67]

to continued ...

Method	Basis set	ρNH_3	$\delta_s NH_3$	$\delta_a NH_3$	ν_s(NH)	ν_a(NH)
B97-3c	def2-QZVP	830.20	1381.89	1651.75	3384.76	3477.05
		[35.62]	[137.83]	[131.36]	[100.61]	[260.16]
B97-3c	LANL2DZ	887.73	1427.18	1706.30	3378.68	3507.31
		[55.23]	[266.10]	[204.22]	[58.63]	[207.15]
B97-3c	LANL2TZ/def2-TZVP	840.59	1385.13	1649.64	3380.96	3471.42
		[42.32]	[146.26]	[144.83]	[95.87]	[199.42]
B97-3c	ZORA-TZVP/TZVP	835.49	1383.07	1648.42	3387.36	3475.87
		[40.51]	[144.97]	[143.38]	[93.85]	[171.96]
B97-3c	ZORA-TZVPP/TZVP	835.40	1383.80	1648.62	3387.16	3475.45
		[40.88]	[145.19]	[143.23]	[93.12]	[172.61]
B97-3c	DKH-TZVP/TZVP	-	-	-	-	-
B97-3c	DKH-TZVPP/TZVP	-	-	-	-	-
B97-3c	Sappo-DZP/DZP	1121.35	1618.99	1713.32	3373.16	3423.53
		[125.52]	[57.16]	[33.07]	[70.87]	[223.55]
B97-3c	Sappo-TZP/DZP	1132.10	1609.00	1721.05	3345.71	3384.98
		[31.74]	[55.16]	[50.26]	[38.48]	[74.06]
B97-3c	Sappo-QZP/DZP	1163.73	1564.33	1705.23	3379.32	3450.70
		[97.95]	[71.47]	[48.70]	[83.42]	[230.16]

to continued ...

Method	Basis set	ρNH_3	$\delta_s NH_3$	$\delta_a NH_3$	ν_s(NH)	ν_a(NH)
B2PLYP	def2-TZVP	846.82	1415.96	1669.18	3412.82	3497.61
		[47.31]	[181.03]	[153.54]	[121.99]	[285.58]
B2PLYP	def2-QZVP	-	-	-	-	-
B2PLYP	LANL2DZ	888.41	1462.46	1725.19	3408.50	3530.91
		[64.56]	[321.71]	[212.64]	[87.53]	[193.23]
B2PLYP	LANL2TZ/def2-TZVP	847.85	1417.84	1668.62	3413.62	3496.90
		[47.86]	[185.04]	[155.40]	[118.83]	[189.45]
B2PLYP	ZORA-TZVP/TZVP	844.87	1414.46	1668.65	3410.98	3495.31
		[45.42]	[176.14]	[152.82]	[120.99]	[227.88]
B2PLYP	ZORA-TZVPP/TZVP	844.78	1415.02	1668.58	3410.71	3495.04
		[45.21]	[176.58]	[149.10]	[120.15]	[275.78]
B2PLYP	DKH-TZVP/TZVP	-	-	-	-	-
B2PLYP	DKH-TZVPP/TZVP	-	-	-	-	-
B2PLYP	Sappo-DZP/DZP	-	-	-	-	-
B2PLYP	Sappo-TZP/DZP	832.94	1392.47	1644.51	3401.76	3496.05
		[52.56]	[139.45]	[134.62]	[133.10]	[111.76]
B2PLYP	Sappo-QZP/DZP	834.15	1393.35	1644.00	3401.82	3496.86
		[48.55]	[140.16]	[132.15]	[131.45]	[281.03]

to continued ...

Method	Basis set	ρNH_3	$\delta_s NH_3$	$\delta_a NH_3$	ν_s(NH)	ν_a(NH)
mPW2PLYP	def2-TZVP	848.53	1423.26	1676.02	3431.62	3516.09
		[50.67]	[190.44]	[152.40]	[124.29]	[316.69]
mPW2PLYP	def2-QZVP	-	-	-	-	-
mPW2PLYP	LANL2DZ	894.07	1471.95	1732.67	3431.54	3550.25
		[66.38]	[339.69]	[216.95]	[87.65]	[301.97]
mPW2PLYP	LANL2TZ/def2-TZVP	850.74	1425.49	1675.17	3432.54	3515.23
		[50.56]	[194.48]	[154.05]	[121.06]	[209.07]
mPW2PLYP	ZORA-TZVP/TZVP	847.88	1422.19	1675.61	3429.98	3513.85
		[46.46]	[185.80]	[152.76]	[123.62]	[291.46]
mPW2PLYP	ZORA-TZVPP/TZVP	847.97	1422.84	1675.84	3429.71	3513.41
		[47.01]	[186.42]	[154.16]	[122.90]	[287.62]
mPW2PLYP	DKH-TZVP/TZVP	-	-	-	-	
mPW2PLYP	DKH-TZVPP/TZVP	-	-	-	-	-
mPW2PLYP	Sappo-DZP/DZP	-	-	-	-	-
mPW2PLYP	Sappo-TZP/DZP	835.20	1400.25	1651.72	3419.73	3514.37
		[51.43]	[148.27]	[136.42]	[134.76]	[172.22]
mPW2PLYP	Sappo-QZP/DZP	-	-	-	-	-
PBE0-DH	def2-TZVP	854.99	1429.42	1679.89	3483.91	3571.12

to continued ...

Method	Basis set	ρNH_3	$\delta_s NH_3$	$\delta_a NH_3$	$\nu_s(NH)$	$\nu_a(NH)$
		[52.77]	[205.51]	[154.84]	[126.89]	[245.51]
PBE0-DH	def2-QZVP	-	-	-	-	-
PBE0-DH	LANL2DZ	-	-	-	-	-
PBE0-DH	LANL2TZ/def2-TZVP	860.38	1431.44	1679.48	3483.11	3569.79
		[54.45]	[209.14]	[156.90]	[123.18]	[337.21]
PBE0-DH	ZORA-TZVP/TZVP	854.61	1429.12	1680.41	3481.84	3569.43
		[50.70]	[204.98]	[156.24]	[124.82]	[331.48]
PBE0-DH	ZORA-TZVPP/TZVP	854.59	1429.77	1680.54	3481.58	3569.12
		[50.61]	[205.46]	[154.94]	[122.90]	[302.07]
PBE0-DH	DKH-TZVP/TZVP	-	-	-	-	-
PBE0-DH	DKH-TZVPP/TZVP	-	-	-	-	-
PBE0-DH	Sappo-DZP/DZP	844.79	1409.92	1661.64	3467.86	3569.82
		[52.73]	[165.96]	[146.08]	[142.19]	[350.24]
PBE0-DH	Sappo-TZP/DZP	843.16	1411.94	1660.46	3467.68	3569.26
		[57.16]	[171.11]	[142.89]	[131.26]	[283.87]
PBE0-DH	Sappo-QZP/DZP	844.31	1412.25	1660.10	3468.83	3570.02
		[54.28]	[171.87]	[142.81]	[138.04]	[342.05]
PBE-QIDH	def2-TZVP	862.79	1437.47	1685.31	3482.55	3573.60

to continued ...

Method	Basis set	ρNH_3	$\delta_s NH_3$	$\delta_a NH_3$	ν_s(NH)	ν_a(NH)
		[54.13]	[205.63]	[155.53]	[129.49]	[153.09]
PBE-QIDH	def2-QZVP	-	-	-	-	-
PBE-QIDH	LANL2DZ	909.51	1494.93	1753.68	3482.69	3609.02
		[68.51]	[369.48]	[228.80]	[96.28]	[378.13]
PBE-QIDH	LANL2TZ/def2-TZVP	864.87	1439.25	1685.10	3482.69	3572.43
		[52.00]	[211.73]	[159.31]	[124.41]	[269.59]
PBE-QIDH	ZORA-TZVP/TZVP	860.50	1435.59	1685.18	3479.47	3571.19
		[49.37]	[204.88]	[156.57]	[127.18]	[288.65]
PBE-QIDH	ZORA-TZVPP/TZVP	-	-	-	-	-
PBE-QIDH	DKH-TZVP/TZVP	-	-	-	-	-
PBE-QIDH	DKH-TZVPP/TZVP	-	-	-	-	-
PBE-QIDH	Sappo-DZP/DZP	851.04	1417.23	1668.35	3472.98	3578.82
		[68.34]	[164.41]	[147.28]	[149.03]	[408.75]
PBE-QIDH	Sappo-TZP/DZP	849.91	1418.57	1666.92	3472.20	3577.62
		[54.77]	[169.57]	[144.63]	[143.17]	[313.30]
PBE-QIDH	Sappo-QZP/DZP	- [-	-	-	-	
DSD-BLYP	def2-TZVP	862.79	1437.47	1685.31	3482.55	3573.60
		[54.13]	[205.63]	[155.53]	[129.49]	[153.09]

to continued ...

Method	Basis set	ρNH_3	$\delta_s NH_3$	$\delta_a NH_3$	ν_s(NH)	ν_a(NH)
DSD-BLYP	def2-QZVP	-	-	-	-	-
DSD-BLYP	LANL2DZ	909.51	1494.93	1753.68	3482.69	3609.02
		[68.51]	[369.48]	[228.80]	[96.28]	[378.13]
DSD-BLYP	LANL2TZ/def2-TZVP	858.22	1432.40	1676.91	3438.03	3527.62
		[48.94]	[201.71]	[154.87]	[124.30]	[154.35]
DSD-BLYP	ZORA-TZVP/TZVP	857.42	1427.41	1676.49	3434.10	3525.46
		[45.26]	[189.03]	[152.75]	[130.11]	[176.66]
DSD-BLYP	ZORA-TZVPP/TZVP	857.71	1428.05	1676.60	3432.82	3524.63
		[45.90]	[188.92]	[153.76]	[129.03]	[135.37]
DSD-BLYP	DKH-TZVP/TZVP	-	-	-	-	-
DSD-BLYP	DKH-TZVPP/TZVP	-	-	-	-	-
DSD-BLYP	Sappo-DZP/DZP	846.67	1406.07	1657.37	3432.59	3535.43
		[66.88]	[145.57]	[139.55]	[146.14]	[393.06]
DSD-BLYP	Sappo-TZP/DZP	-	-	-	-	-
DSD-BLYP	Sappo-QZP/DZP	-	-	-	-	-
RI-SCS-MP2	def2-TZVP	864.74	1438.39	1675.45	3397.77	3492.69
		[49.68]	[188.09]	[145.73]	[121.71]	[195.91]
RI-SCS-MP2	def2-QZVP	-	-	-	-	-

to continued ...

Method	Basis set	ρNH_3	$\delta_s NH_3$	$\delta_a NH_3$	ν_s(NH)	ν_a(NH)
RI-SCS-MP2	LANL2DZ	882.68	1480.49	1732.34	3384.54	3507.74
		[61.97]	[311.88]	[203.11]	[80.38]	[156.00]
RI-SCS-MP2	LANL2TZ/def2-TZVP	856.38	1438.69	1674.73	3400.19	3492.60
		[45.86]	[197.41]	[147.34]	[118.01]	[188.69]
RI-SCS-MP2	ZORA-TZVP/TZVP	860.43	1434.23	1674.81	3395.37	3490.34
		[44.06]	[182.11]	[146.95]	[122.65]	[205.36]
RI-SCS-MP2	ZORA-TZVPP/TZVP	860.89	1434.60	1674.83	3394.05	3489.95
		[44.64]	[181.53]	[146.90]	[120.83]	[114.70]
RI-SCS-MP2	DKH-TZVP/TZVP	-	-	-	-	-
RI-SCS-MP2	DKH-TZVPP/TZVP	-	-	-	-	-
RI-SCS-MP2	Sappo-DZP/DZP	-	-	-	-	-
RI-SCS-MP2	Sappo-TZP/DZP	-	-	-	-	-
RI-SCS-MP2	Sappo-QZP/DZP	-	-	-	-	-
HF	def2-TZVP	873.37	1524.81	1785.98	3628.85	3709.76
		[80.06]	[290.36]	[158.79]	[118.37]	[287.92]
HF	def2-QZVP	869.55	1523.29	1788.70	3638.79	3720.77
		[69.41]	[272.57]	[145.68]	[122.78]	[265.70]
HF	LANL2DZ	929.95	1576.66	1845.83	3635.52	3754.23

to continued ...

Method	Basis set	ρNH_3	$\delta_s NH_3$	$\delta_a NH_3$	ν_s(NH)	ν_a(NH)
		[99.52]	[495.31]	[252.57]	[76.40]	[396.44]
HF	LANL2TZ/def2-TZVP	882.74	1526.45	1785.35	3628.39	3708.60
		[77.68]	[289.56]	[159.21]	[112.26]	[299.21]
HF	ZORA-TZVP/TZVP	874.05	1525.72	1786.58	3628.11	3708.85
		[73.40]	[286.63]	[160.60]	[117.88]	[311.48]
HF	ZORA-TZVPP/TZVP	873.43	1525.96	1786.69	3627.99	3708.51
		[73.65]	[287.35]	[160.47]	[117.40]	[309.18]
HF	DKH-TZVP/TZVP	1063.29	1608.09	1809.98	3647.86	3732.78
		[33.32]	[364.85]	[148.55]	[77.58]	[174.53]
HF	DKH-TZVPP/TZVP	-	-	-	-	-
HF	Sappo-DZP/DZP	864.09	1509.48	1772.09	3621.95	3720.23
		[93.31]	[246.50]	[137.02]	[138.06]	[365.62]
HF	Sappo-TZP/DZP	-	-	-	-	-
HF	Sappo-QZP/DZP	863.30	1511.73	1770.84	3623.04	3719.07
		[77.00]	[253.68]	[150.16]	[136.68]	[383.65]
BP86	Sappo-TZP/DZP	804.49	1335.57	1585.28	3292.49	3382.07
		[45.23]	[109.60]	[128.29]	[106.45]	[330.65] MAD 47.3 cm^{-}
PBE	Sappo-TZP/DZP	803.53	1332.79	1581.89	3300.75	3390.65

to continued ...

Method	Basis set	ρNH_3	$\delta_s NH_3$	$\delta_a NH_3$	ν_s(NH)	ν_a(NH)
		[43.59]	[111.18]	[127.57]	[106.71]	[183.89] MAD 49.6 cm^-
B3P86	Sappo-TZP/DZP	825.33	1378.96	1626.43	3389.35	3482.31
		[52.40]	[146.69]	[137.31]	[126.21]	[346.16] MAD 99.8 cm^-
B3PW91	Sappo-TZP/DZP	826.60	1382.08	1631.72	3402.05	3495.82
		[52.59]	[144.86]	[136.16]	[124.85]	[353.28] MAD 106.9 cm^-
HF-by-MMFF94	LANL2DZ/6-31G(d)	898.75	1568.39	1828.08	3642.88	3738.99
		[103.20]	[327.77]	[198.23]	[140.17]	[362.16] MAD 294.7 cm^-

end.

Cisplatin [$Pt(NH_3)_2Cl_2$]

Table S8: Calculated wavenumbers (in cm^{-1}) for twelve Harmonic Vibrational Frequency of cisplatin $[Pt(NH_3)_2Cl_2]$ with IR Intensity (in km/mol) in brackets. 1: in plane bending (Cl-Pt-Cl), (2): in plane bending (N-Pt-N), (3): ν_a(Pt-Cl), (4): ν_s(Pt-Cl), (5): ν_a(Pt-N), (6): ν_s(Pt-N), (7): ρNH_3, (8): δ_s(H-N-H), (9): δ_s(H-N-H), (10): δ_d(H-N-H), (11): $\nu_s NH_3$, (12): $\nu_a NH_3$. The level of theory used in the standard MMFF94 force field is included (HF-by-MMFF94). Abbreviated names for the basis sets are used here, with their full names listed in Table 1.

Method	Basis set	1	2	3	4	5	6	7	8	9	10	11	12
Experimental[8]	-	156	254	315	323	511	519	796	1299	1314	1625	3206	3285
M06L	def2-TZVP	149	222	331	341	418	431	763	1241	1247	1640	3362	3518
		[0]	[24]	[35]	[30]	[8]	[1]	[85]	[126]	[72]	[31]	[63]	[52]
M06L	def2-QZVP	150	222	330	340	418	432	760	1239	1244	1679	3386	3541
		[0]	[25]	[34]	[29]	[7]	[0]	[71]	[119]	[75]	[27]	[61]	[47]
M06L	LANL2DZ	134	228	311	321	454	459	850	1300	1305	1728	3337	3547
		[0]	[21]	[29]	[29]	[16]	[3]	[89]	[272]	[154]	[67]	[70]	[64]
M06L	LANL2TZ/def2-TZVP	149	221	332	343	422	435	773	1243	1248	1673	3361	3518
		[0]	[25]	[34]	[30]	[9]	[1]	[88]	[124]	[75]	[32]	[57]	[57]
M06L	ZORA-TZVP/TZVP	151	225	334	345	427	439	771	1249	1254	1641	3359	3515
		[0]	[24]	[34]	[30]	[6]	[0]	[85]	[123]	[77]	[30]	[68]	[46]
M06L	ZORA-TZVPP/TZVP	151	225	334	345	426	438	771	1249	1255	1641	3359	3514
		[0]	[24]	[34]	[30]	[6]	[0]	[85]	[123]	[77]	[30]	[69]	[44]
M06L	DKH-TZVP/TZVP	158	271	326	340	417	428	848	1287	1297	1624	3312	3542

to continued ...

Method	Basis set	1	2	3	4	5	6	7	8	9	10	11	12
		[0]	[21]	[36]	[38]	[3]	[0]	[83]	[99]	[88]	[32]	[156]	[32]
M06L	DKH-TZVPP/TZVP	159	274	325	339	413	423	852	1290	1301	1624	3308	3539
		[0]	[21]	[37]	[39]	[3]	[0]	[83]	[97]	[88]	[32]	[159]	[33]
M06L	Sappo-DZP/DZP	148	228	333	344	417	430	768	1247	1253	1660	3364	3529
		[0]	[25]	[36]	[32]	[8]	[0]	[94]	[107]	[68]	[38]	[64]	[79]
M06L	Sappo-TZP/DZP	149	228	332	343	422	436	769	1248	1254	1659	3367	3529
		[0]	[25]	[36]	[31]	[5]	[0]	[86]	[108]	[69]	[36]	[60]	[72]
M06L	Sappo-QZP/DZP	149	229	329	341	421	435	771	1249	1255	1658	3367	3530
		[0]	[25]	[36]	[31]	[6]	[0]	[84]	[109]	[71]	[36]	[60]	[72]
B3LYP	def2-TZVP	150	221	334	345	428	440	771	1246	1253	1669	3364	3499
		[0]	[26]	[29]	[26]	[7]	[1]	[73]	[172]	[100]	[36]	[69]	[59]
B3LYP	def2-QZVP	150	222	332	342	430	442	768	1248	1255	1668	3375	3508
		[0]	[26]	[29]	[25]	[7]	[1]	[63]	[152]	[98]	[31]	[62]	[61]
B3LYP	LANL2DZ	134	228	318	328	462	467	859	1300	1306	1711	3345	3523
		[0]	[24]	[25]	[25]	[14]	[2]	[82]	[296]	[182]	[70]	[61]	[73]
B3LYP	LANL2TZ/def2-TZVP	150	221	333	344	430	441	783	1251	1258	1668	3364	3498
		[0]	[26]	[28]	[25]	[8]	[1]	[75]	[169]	[105]	[39]	[68]	[62]
B3LYP	ZORA-TZVP/TZVP	151	224	334	345	435	447	776	1250	1256	1668	3362	3545

to continued ...

Method	Basis set	1	2	3	4	5	6	7	8	9	10	11	12
		[0]	[25]	[28]	[25]	[7]	[1]	[70]	[161]	[99]	[34]	[64]	[56]
B3LYP	ZORA-TZVPP/TZVP	151	224	334	345	434	446	776	1250	1257	1669	3361	3545
		[0]	[25]	[28]	[25]	[7]	[1]	[70]	[161]	[99]	[34]	[65]	[56]
B3LYP	DKH-TZVP/TZVP	158	265	327	342	427	438	848	1291	1302	1618	3312	3518
		[0]	[25]	[31]	[31]	[3]	[0]	[69]	[137]	[108]	[34]	[126]	[49]
B3LYP	DKH-TZVPP/TZVP	158	268	326	341	422	432	852	1295	1307	1618	3309	3516
		[0]	[25]	[31]	[32]	[3]	[0]	[69]	[135]	[107]	[35]	[119]	[47]
B3LYP	Sappo-DZP/DZP	149	227	335	346	437	449	773	1234	1240	1641	3331	3478
		[0]	[20]	[29]	[27]	[7]	[1]	[79]	[113]	[73]	[34]	[78]	[68]
B3LYP	Sappo-TZP/DZP	150	227	333	344	442	455	773	1236	1242	1639	3333	3478
		[0]	[27]	[29]	[26]	[5]	[0]	[73]	[113]	[74]	[32]	[75]	[61]
B3LYP	Sappo-QZP/DZP	150	228	332	343	443	457	776	1238	1244	1638	3334	3479
		[0]	[27]	[29]	[25]	[5]	[0]	[71]	[113]	[75]	[32]	[75]	[61]
mPW1PW	def2-TZVP	155	231	351	362	456	468	782	1253	1260	1673	3395	3546
		[0]	[29]	[29]	[24]	[6]	[0]	[83]	[180]	[106]	[36]	[79]	[64]
mPW1PW	def2-QZVP	154	232	348	359	458	471	779	1251	1258	1671	3402	3553
		[0]	[29]	[29]	[23]	[5]	[0]	[72]	[161]	[105]	[31]	[71]	[65]
mPW1PW	LANL2DZ	138	236	332	341	489	494	875	1323	1330	1727	3383	3573

to continued ...

Method	Basis set	1	2	3	4	5	6	7	8	9	10	11	12
		[0]	[26]	[26]	[25]	[13]	[2]	[89]	[312]	[194]	[74]	[93]	[81]
mPW1PW	LANL2TZ/def2-TZVP	154	230	350	361	458	470	795	1259	1265	1672	3395	3602
		[0]	[30]	[29]	[24]	[7]	[0]	[85]	[177]	[112]	[39]	[78]	[67]
mPW1PW	ZORA-TZVP/TZVP	155	233	351	362	462	474	786	1254	1262	1673	3394	3600
		[0]	[29]	[28]	[24]	[5]	[0]	[80]	[171]	[107]	[34]	[69]	[63]
mPW1PW	ZORA-TZVPP/TZVP	155	233	351	362	461	474	786	1255	1262	1673	3392	3599
		[0]	[29]	[28]	[24]	[5]	[0]	[80]	[171]	[107]	[34]	[70]	[63]
mPW1PW	DKH-TZVP/TZVP	163	284	342	357	457	461	868	1307	1322	1620	3349	3571
		[0]	[29]	[31]	[31]	[2]	[0]	[70]	[143]	[112]	[42]	[86]	[49]
mPW1PW	DKH-TZVPP/TZVP	164	290	341	355	451	454	875	1317	1332	1622	3349	3568
		[0]	[28]	[31]	[32]	[0]	[2]	[66]	[139]	[109]	[46]	[88]	[46]
mPW1PW	Sappo-DZP/DZP	154	236	351	362	459	472	784	1242	1249	1650	3363	3529
		[0]	[27]	[30]	[26]	[6]	[0]	[91]	[128]	[82]	[36]	[86]	[82]
mPW1PW	Sappo-TZP/DZP	-	236	349	361	464	479	784	1244	1251	1648	3366	3529
		-	[29]	[29]	[25]	[4]	[0]	[83]	[128]	[83]	[34]	[82]	[74]
mPW1PW	Sappo-QZP/DZP	154	237	347	359	465	480	786	1245	1251	1647	3366	3530
		[0]	[30]	[29]	[24]	[4]	[0]	[81]	[128]	[84]	[35]	[81]	[74]
PBE0	def2-TZVP	155	232	352	363	459	472	783	1248	1256	1667	3385	3596

to continued ...

Method	Basis set	1	2	3	4	5	6	7	8	9	10	11	12
		[0]	[29]	[29]	[24]	[5]	[0]	[83]	[180]	[107]	[36]	[81]	[65]
PBE0	def2-QZVP	154	233	349	360	461	474	780	1247	1254	1665	3392	3546
		[0]	[29]	[28]	[23]	[5]	[0]	[72]	[161]	[105]	[31]	[73]	[65]
PBE0	LANL2DZ	138	237	333	342	493	498	877	1321	1328	1722	3372	3638
		[0]	[26]	[26]	[25]	[13]	[2]	[88]	[311]	[196]	[74]	[96]	[81]
PBE0	LANL2TZ/def2-TZVP	154	232	351	362	461	473	796	1254	1261	1666	3385	3596
		[0]	[30]	[29]	[24	[6]	[0]	[85]	[177]	[113]	[39]	[82]	[69]
PBE0	ZORA-TZVP/TZVP	155	234	352	363	464	477	786	1250	1257	1666	3384	3594
		[0]	[29]	[28]	[24	[5]	[0]	[80]	[171]	[108]	[34]	[72]	[64]
PBE0	ZORA-TZVPP/TZVP	155	234	352	363	464	477	786	1251	1258	1667	3382	3593
		[0]	[29]	[28]	[24	[5]	[0]	[80]	[171]	[107]	[34]	[74]	[63]
PBE0	DKH-TZVP/TZVP	164	289	343	358	463	464	871	1310	1325	1615	3345	3563
		[0]	[29]	[31]	[31	[0]	[2]	[64]	[141]	[109]	[48]	[119]	[46]
PBE0	DKH-TZVPP/TZVP	164	294	342	356	452	458	878	1318	1334	1616	3343	3560
		[0]	[28]	[31]	[32	[0]	[2]	[61]	[138]	[106]	[49]	[111]	[44]
PBE0	Sappo-DZP/DZP	154	237	352	363	461	474	785	1238	1245	1644	3355	3524
		[0]	[16]	[30]	[26	[6]	[0]	[91]	[128]	[83]	[36]	[90]	[82]
PBE0	Sappo-TZP/DZP	154	238	350	362	467	482	784	1240	1247	1643	3357	3524

to continued ...

Method	Basis set	1	2	3	4	5	6	7	8	9	10	11	12
		[0]	[30]	[29]	[25	[4]	[0]	[83]	[129]	[84]	[34]	[86]	[74]
PBE0	Sappo-QZP/DZP	154	239	348	360	468	482	786	1241	1248	1641	3357	3525
		[0]	[31]	[29]	[24	[4]	[0]	[81]	[128]	[85]	[34]	[85]	[73]
TPSSh	def2-TZVP	151	229	345	355	453	464	777	1254	1261	1639	3329	3537
		[0]	[28]	[28]	[24	[6]	[0]	[80]	[152]	[87]	[31]	[96]	[49]
TPSSh	def2-QZVP	150	230	343	353	455	466	773	1252	1258	1639	3333	3540
		[0]	[28]	[27]	[23	[5]	[0]	[68]	[135]	[86]	[27]	[91]	[49]
TPSSh	LANL2DZ	135	233	327	336	484	487	860	1307	1313	1719	3314	3583
		[0]	[18]	[24]	[23	[12]	[2]	[88]	[270]	[160]	[63]	[109]	[62]
TPSSh	LANL2TZ/def2-TZVP	150	227	343	354	454	464	789	1259	1265	1675	3329	3537
		[0]	[29]	[27]	[23	[7]	[0]	[81]	[150]	[93]	[35]	[94]	[53]
TPSSh	ZORA-TZVP/TZVP	151	230	345	356	458	469	779	1254	1260	1639	3327	3534
		[0]	[28]	[27]	[23	[5]	[0]	[76]	[144]	[88]	[31]	[89]	[48]
TPSSh	ZORA-TZVPP/TZVP	151	231	345	356	457	469	779	1255	1261	1639	3325	3534
		[0]	[28]	[27]	[24]	[5]	[0]	[77]	[144]	[87]	[31]	[91]	[47]
TPSSh	DKH-TZVP/TZVP	159	277	337	352	447	456	856	1296	1308	1621	3267	3504
		[0]	[27]	[30]	[30]	[2]	[0]	[76]	[119]	[98]	[33]	[156]	[28]
TPSSh	DKH-TZVPP/TZVP	160	280	336	352	441	448	860	1301	1313	1621	3262	3501

to continued ...

Method	Basis set	1	2	3	4	5	6	7	8	9	10	11	12
		[0]	[27]	[30]	[31]	[2]	[0]	[76]	[116]	[97]	[33]	[157]	[35]
TPSSh	Sappo-DZP/DZP	150	234	346	357	457	468	776	1241	1247	1651	3282	3451
		[0]	[29]	[28]	[25]	[6]	[0]	[86]	[99]	[63]	[31]	[110]	[55]
TPSSh	Sappo-TZP/DZP	150	234	344	355	461	475	776	1242	1248	1608	3285	3451
		[0]	[29]	[28]	[24]	[4]	[0]	[79]	[100]	[64]	[29]	[106]	[49
TPSSh	Sappo-QZP/DZP	150	235	342	354	463	476	779	1243	1249	1648	3285	3452
		[0]	[29]	[27]	[24]	[4]	[0]	[77]	[100]	[64]	[29]	[105]	[49]
M06	def2-TZVP	150	218	342	353	428	440	748	1212	1219	1639	3362	3519
		[0]	[27]	[31]	[25]	[8]	[1]	[80]	[183]	[107]	[35]	[52]	[77]
M06	def2-QZVP	149	219	340	351	431	443	746	1213	1219	1643	3383	3534
		[0]	[26]	[31]	[25]	[7]	[1]	[68]	[161]	[107]	[31]	[47]	[72]
M06	LANL2DZ	137	222	325	336	462	469	839	1281	1288	1695	3350	3552
		[0]	[26]	[28]	[25]	[14]	[3]	[85]	[330]	[206]	[78]	[60]	[96]
M06	LANL2TZ/def2-TZVP	150	216	341	352	425	437	756	1213	1219	1638	3362	3519
		[0]	[27]	[31]	[25]	[9]	[1]	[83]	[179]	[109]	[39]	[53]	[83]
M06	ZORA-TZVP/TZVP	151	221	344	356	437	449	753	1220	1226	1640	3363	3518
		[0]	[26]	[30]	[26]	[6]	[1]	[83]	[183]	[117]	[33]	[53]	[70]
M06	ZORA-TZVPP/TZVP	151	222	344	356	436	449	753	1220	1226	1640	3362	3518

to continued ...

Method	Basis set	1	2	3	4	5	6	7	8	9	10	11	12
		[0]	[26]	[30]	[26]	[6]	[1]	[84]	[183]	[117]	[33]	[54]	[69]
M06	DKH-TZVP/TZVP	158	268	339	352	433	433	820	1265	1277	1591	3341	3556
		[0]	[27]	[32]	[30]	[3]	[0]	[65]	[159]	[120	[47]	[70]	[46]
M06	DKH-TZVPP/TZVP	159	272	338	351	426	428	825	1270	1282	1591	3338	3554
		[0]	[27]	[33]	[31]	[0]	[3]	[64]	[157]	[119]	[48]	[68]	[46]
M06	Sappo-DZP/DZP	150	222	345	357	436	449	751	1213	1219	1618	3351	3513
		[0]	[28]	[31]	[27]	[7]	[1]	[95]	[144]	[96]	[39]	[76]	[98]
M06	Sappo-TZP/DZP	151	222	344	356	440	453	749	1212	1218	1616	3354	3514
		[0]	[28]	[31]	[26]	[6]	[0]	[88]	[146]	[97]	[37]	[73]	[92]
M06	Sappo-QZP/DZP	150	224	343	355	443	456	752	1214	1220	1615	3354	3515
		[0]	[28]	[31]	[26]	[6]	[0]	[87]	[146]	[98]	[37]	[73]	[91]
M06-2X	def2-TZVP	152	221	327	337	391	411	764	1257	1265	1668	3425	3554
		[0]	[22]	[51]	[39]	[6]	[0]	[77]	[214]	[126]	[42]	[34]	[95]
M06-2X	def2-QZVP	151	224	321	330	392	413	764	1256	1264	1668	3430	3561
		[0]	[22]	[50]	[37]	[5]	[0]	[68]	[193]	[123]	[36]	[37]	[96]
M06-2X	LANL2DZ	135	235	320	331	450	458	874	1332	1341	1719	3398	3574
		[0]	[16]	[37]	[34]	[18]	[4]	[89]	[374]	[243]	[87]	[51]	[112]
M06-2X	LANL2TZ/def2-TZVP	153	223	330	340	402	420	782	1264	1272	1668	3422	3552

to continued ...

Method	Basis set	1	2	3	4	5	6	7	8	9	10	11	12
		[0]	[23]	[48]	[37]	[8]	[1]	[80]	[207]	[130]	[46]	[35]	[100]
M06-2X	ZORA-TZVP/TZVP	152	223	324	336	400	420	770	1260	1268	1668	3423	3552
		[0]	[22]	[47]	[36]	[5]	[1]	[72]	[203]	[124]	[40]	[33]	[93]
M06-2X	ZORA-TZVPP/TZVP	152	224	323	334	398	419	770	1260	1268	1669	3422	3551
		[0]	[22]	[48]	[37]	[5]	[0]	[72]	[203]	[124]	[40]	[33]	[92]
M06-2X	DKH-TZVP/TZVP	145	285	317	320	403	426	790	1376	1386	1653	3451	3537
		[0]	[24]	[50]	[37]	[1]	[3]	[22]	[161]	[75]	[77]	[19]	[101]
M06-2X	DKH-TZVPP/TZVP	144	291	314	315	396	421	792	1387	1398	1656	3448	3533
		[0]	[20]	[49]	[43]	[2]	[3]	[21]	[157]	[74]	[77]	[19]	[107]
M06-2X	Sappo-DZP/DZP	149	231	326	338	403	422	774	1253	1260	1645	3391	3531
		[0]	[22]	[50]	[39]	[8]	[1]	[82]	[156]	[98]	[42]	[44]	[102]
M06-2X	Sappo-TZP/DZP	150	232	319	331	407	428	776	1254	1261	1643	3392	3531
		[0]	[22]	[50]	[38]	[5]	[0]	[74]	[154]	[100]	[40]	[39]	[92]
M06-2X	Sappo-QZP/DZP	149	232	315	328	405	426	777	1255	1261	1642	3393	3531
		[0]	[22]	[49]	[38]	[5]	[0]	[73]	[154]	[100]	[41]	[39]	[92]
LC-BLYP	def2-TZVP	155	236	364	376	478	492	788	1241	1249	1633	3325	3479
		[0]	[30]	[31]	[25]	[5]	[0]	[86]	[225]	[140]	[39]	[74]	[81]
LC-BLYP	def2-QZVP	154	235	361	372	480	494	784	1241	1249	1631	3336	3487

to continued ...

Method	Basis set	1	2	3	4	5	6	7	8	9	10	11	12
		[0]	[30]	[31]	[23]	[4]	[0]	[74]	[199]	[137]	[35]	[75]	[83]
LC-BLYP	LANL2DZ	140	245	346	356	512	520	888	1319	1326	1682	3318	3509
		[0]	[27]	[28]	[25]	[11]	[1]	[91]	[368]	[249]	[81]	[92]	[96]
LC-BLYP	LANL2TZ/def2-TZVP	154	235	363	375	481	493	801	1247	1254	1633	3324	3479
		[0]	[31]	[31]	[24]	[6]	[0]	[88]	[221]	[148]	[43]	[62]	[84]
LC-BLYP	ZORA-TZVP/TZVP	155	237	364	376	484	498	792	1243	1251	1633	3325	3532
		[0]	[29]	[29]	[24]	[4]	[0]	[81]	[212]	[139]	[38]	[68]	[79]
LC-BLYP	ZORA-TZVPP/TZVP	155	237	364	376	484	498	792	1243	1251	1633	3324	3532
		[0]	[29]	[30]	[24]	[4]	[0]	[81]	[212]	[139]	[37]	[70]	[78]
LC-BLYP	DKH-TZVP/TZVP	164	293	356	370	476	481	874	1306	1321	1582	3297	3508
		[0]	[31]	[32]	[30]	[0]	[2]	[61]	[178]	[136]	[53]	[97]	[53]
LC-BLYP	DKH-TZVPP/TZVP	165	299	354	368	465	477	882	1316	1333	1584	3299	3505
		[0]	[30]	[32]	[31]	[0]	[2]	[56]	[174]	[130]	[57]	[73]	[48]
LC-BLYP	Sappo-DZP/DZP	154	245	366	377	489	503	796	1227	1233	1606	3288	3460
		[0]	[32]	[31]	[26]	[5]	[0]	[95]	[153]	[111]	[37]	[99]	[93]
LC-BLYP	Sappo-TZP/DZP	155	244	364	375	492	508	793	1227	1233	1604	3292	3461
		[0]	[32]	[30]	[25]	[3]	[0]	[87]	[154	[111]	[35]	[92]	[86]
LC-BLYP	Sappo-QZP/DZP	154	245	362	374	493	509	795	1228	1234	1602	3292	3462

to continued ...

Method	Basis set	1	2	3	4	5	6	7	8	9	10	11	12
		[0]	[32]	[30]	[24]	[3]	[0]	[85]	[154]	[111]	[35]	[92]	[86]
CAM-B3LYP	def2-TZVP	154	229	350	361	451	464	785	1265	1273	1675	3401	3534
		[0]	[28]	[32]	[27]	[6]	[1]	[80]	[206]	[124]	[39]	[54]	[76]
CAM-B3LYP	def2-QZVP	153	229	347	358	453	467	782	1266	1273	1674	3412	3543
		[0]	[27]	[32]	[25]	[6]	[1]	[70]	[183]	[122]	[35]	[53]	[78]
CAM-B3LYP	LANL2DZ	138	236	332	342	484	491	879	1330	1338	1721	3389	3562
		[0]	[25]	[28]	[26]	[14]	[3]	[89]	[343]	[221]	[79]	[57]	[90]
CAM-B3LYP	LANL2TZ/def2-TZVP	153	229	349	360	453	465	798	1271	1278	1675	3400	3533
		[0]	[28]	[32]	[26]	[7]	[1]	[83]	[202]	[130]	[43]	[53]	[79]
CAM-B3LYP	ZORA-TZVP/TZVP	154	231	350	361	457	470	790	1268	1275	1675	3400	3532
		[0]	[27]	[31]	[26]	[6]	[1]	[76]	[195]	[124]	[37]	[49]	[72]
CAM-B3LYP	ZORA-TZVPP/TZVP	155	231	350	361	457	470	789	1268	1276	1675	3399	3532
		[0]	[27]	[31]	[26]	[6]	[1]	[77]	[195]	[123]	[37]	[50]	[71]
CAM-B3LYP	DKH-TZVP/TZVP	162	279	342	356	455	456	863	1321	1335	1626	3375	3559
		[0]	[28]	[33]	[31]	[2]	[1]	[60]	[167]	[122]	[50]	[80]	[50]
CAM-B3LYP	DKH-TZVPP/TZVP	162	283	341	354	447	451	869	1327	1342	1627	3372	3556
		[0]	[27]	[34]	[32]	[0]	[3]	[59]	[164]	[120]	[51]	[79]	[48]
CAM-B3LYP	Sappo-DZP/DZP	153	235	351	362	459	472	789	1253	1260	1649	3371	3517

to continued ...

Method	Basis set	1	2	3	4	5	6	7	8	9	10	11	12
		[0]	[24]	[32]	[28]	[6]	[0]	[87]	[142]	[95]	[38]	[64]	[89]
CAM-B3LYP	Sappo-TZP/DZP	154	235	349	360	464	478	788	1254	1260	1646	3374	3518
		[0]	[29]	[32]	[26]	[5]	[0]	[80]	[143]	[96]	[36]	[60]	[82]
CAM-B3LYP	Sappo-QZP/DZP	153	236	347	359	466	480	791	1255	1262	1645	3375	3519
		[0]	[29]	[32]	[26]	[5]	[0]	[78]	[143]	[97]	[36]	[60]	[81]
ωB97X-D3BJ	def2-TZVP	156	231	363	373	461	474	788	1270	1278	1680	3429	3559
		[0]	[28]	[34]	[27]	[5]	[1]	[81]	[203]	[125]	[38]	[45]	[73]
ωB97X-D3BJ	def2-QZVP	156	232	360	370	463	476	786	1270	1277	1679	3436	3566
		[0]	[28]	[33]	[26]	[5]	[0]	[71]	[182]	[123]	[34]	[47]	[74]
ωB97X-D3BJ	LANL2DZ	140	238	345	354	493	499	883	1341	1350	1733	3421	3589
		[0]	[25]	[30]	[27]	[13]	[3]	[89]	[336]	[223]	[79]	[58]	[87]
ωB97X-D3BJ	LANL2TZ/def2-TZVP	156	231	362	373	463	475	801	1275	1283	1680	3429	3559
		[0]	[29]	[34]	[27]	[6]	[1]	[84]	[200]	[132]	[42]	[44]	[76]
ωB97X-D3BJ	ZORA-TZVP/TZVP	157	233	363	373	466	479	791	1272	1279	1680	3429	3558
		[0]	[28]	[32]	[26]	[5]	[1]	[77]	[194]	[126]	[37]	[42]	[69]
ωB97X-D3BJ	ZORA-TZVPP/TZVP	157	233	363	373	466	479	791	1272	1280	1681	3428	3557
		[0]	[28]	[32]	[26]	[5]	[1]	[78]	[193]	[125]	[37]	[42]	[68]
ωB97X-D3BJ	DKH-TZVP/TZVP	164	286	355	366	461	469	867	1335	1351	1633	3424	3587

to continued ...

Method	Basis set	1	2	3	4	5	6	7	8	9	10	11	12
		[0]	[29]	[35]	[30]	[0]	[3]	[47]	[166]	[113]	[61]	[32]	[39]
ωB97X-D3BJ	DKH-TZVPP/TZVP	164	291	354	365	452	464	874	1343	1359	1634	3423	3585
		[0]	[29]	[35]	[31]	[0]	[3]	[44]	[164]	[110]	[62]	[32]	[38]
ωB97X-D3BJ	Sappo-DZP/DZP	155	237	364	374	465	478	790	1261	1268	1658	3403	3547
		[0]	[29]	[35]	[28]	[6]	[0]	[88]	[146]	[97]	[38]	[51]	[91]
ωB97X-D3BJ	Sappo-TZP/DZP	156	237	361	372	469	484	790	1261	1268	1656	3405	3547
		[0]	[30]	[34]	[27]	[4]	[0]	[80]	[146]	[99]	[37]	[49]	[84]
ωB97X-D3BJ	Sappo-QZP/DZP	155	238	359	370	471	485	792	1262	1269	1655	3406	3548
		[0]	[30]	[33]	[27]	[4]	[0]	[78]	[145]	[99]	[37]	[49]	[84]
B97-3c	def2-TZVP	153	225	333	345	440	451	775	1221	1228	1616	3311	3533
		[0]	[27]	[24]	[23]	[6]	[0]	[67]	[140]	[85]	[33]	[100]	[46]
B97-3c	def2-QZVP	153	224	331	343	441	453	771	1223	1230	1619	3318	3536
		[0]	[27]	[24]	[21]	[5]	[0]	[57]	[121]	[82]	[28]	[103]	[45]
B97-3c	LANL2DZ	135	231	317	327	473	477	861	1278	1283	1700	3286	3572
		[0]	[24]	[23]	[24]	[11]	[1]	[73]	[253]	[160]	[63]	[112]	[57]
B97-3c	LANL2TZ/def2-TZVP	152	224	332	344	441	452	786	1227	1233	1653	3310	3532
		[0]	[27]	[24]	[22]	[6]	[0]	[68]	[139]	[90]	[35]	[102]	[50]
B97-3c	ZORA-TZVP/TZVP	154	227	334	346	446	457	776	1222	1229	1614	3314	3534

to continued ...

Method	Basis set	1	2	3	4	5	6	7	8	9	10	11	12
		[0]	[26]	[23]	[22]	[5]	[0]	[65]	[132]	[85]	[33]	[99]	[45]
B97-3c	ZORA-TZVPP/TZVP	154	228	334	346	445	457	776	1223	1230	1615	3312	3534
		[0]	[26]	[23]	[22]	[5]	[0]	[65]	[132]	[85]	[33]	[105]	[45]
B97-3c	DKH-TZVP/TZVP	164	285	325	341	435	441	874	1296	1313	1600	3264	3489
		[0]	[23]	[27]	[33]	[0]	[2]	[48]	[100]	[80]	[47]	[112]	[28]
B97-3c	DKH-TZVPP/TZVP	165	290	324	340	422	435	887	1308	1326	1601	3258	3482
		[0]	[19]	[28]	[38]	[0]	[2]	[45]	[95]	[77]	[48]	[119]	[27]
B97-3c	Sappo-DZP/DZP	139	261	314	345	463	474	1138	1474	1477	1679	3259	3454
		[2]	[24]	[6]	[1]	[17]	[17]	[55]	[29]	[33]	[21]	[142]	[21]
B97-3c	Sappo-TZP/DZP	150	277	378	387	421	465	1120	1479	1492	1679	3199	3348
		[0]	[22]	[10]	[6]	[11]	[15]	[70]	[16]	[14]	[23]	[172]	[32]
B97-3c	Sappo-QZP/DZP	123	211	386	393	418	453	1185	1403	1410	1724	3335	3517
		[2]	[12]	[21]	[17]	[6]	[1]	[38]	[49]	[54]	[25]	[62]	[28]
B2PLYP	def2-TZVP	-	-	-	-	-	-	-	-	-	-	-	-
		-	-	-	-	-	-	-	-	-	-	-	-
B2PLYP	def2-QZVP	160	230	348	357	457	470	781	1260	1267	1677	3394	3586
		[0]	[28]	[29]	[23]	[6]	[1]	[63]	[152]	[100]	[31]	[45]	[62]
B2PLYP	LANL2DZ	136	228	325	334	464	469	859	1323	1331	1731	3379	3546

to continued ...

Method	Basis set	1	2	3	4	5	6	7	8	9	10	11	12
		[0]	[24]	[25]	[23]	[15]	[4]	[79]	[300]	[187]	[72]	[44]	[81]
B2PLYP	LANL2TZ/def2-TZVP	154	229	344	354	451	461	803	1276	1283	1681	3380	3516
		[0]	[27]	[30]	[25]	[7]	[1]	[75]	[168]	[109]	[40]	[53]	[71]
B2PLYP	ZORA-TZVP/TZVP	157	230	351	361	459	470	788	1264	1271	1680	3379	3515
		[0]	[27]	[28]	[24]	[6]	[1]	[69]	[162]	[100]	[35]	[42]	[64]
B2PLYP	ZORA-TZVPP/TZVP	157	231	352	362	459	471	787	1264	1272	1680	3377	3514
		[0]	[27]	[28]	[24]	[6]	[1]	[69]	[162]	[100]	[34]	[43]	[63]
B2PLYP	DKH-TZVP/TZVP	164	282	343	355	456	458	862	1318	1332	1633	3354	3539
		[0]	[28]	[31]	[29]	[0]	[3]	[54]	[137]	[98]	[50]	[49]	[44]
B2PLYP	DKH-TZVPP/TZVP	165	286	343	355	449	454	867	1324	1338	1634	3351	3537
		[0]	[27]	[31]	[30]	[0]	[3]	[52]	[135]	[97]	[52]	[54]	[42]
B2PLYP	Sappo-DZP/DZP	156	234	348	359	456	468	786	1254	1261	1659	3367	3511
		[0]	[28]	[30]	[26]	[7]	[1]	[78]	[114]	[73]	[36]	[49]	[75]
B2PLYP	Sappo-TZP/DZP	157	235	349	360	462	475	785	1253	1259	1657	3368	3511
		[0]	[28]	[29]	[25]	[5]	[0]	[71]	[115]	[74]	[34]	[46]	[65]
B2PLYP	Sappo-QZP/DZP	157	236	348	359	464	477	787	1254	1261	1656	3368	3511
		[0]	[28]	[29]	[24]	[5]	[0]	[69]	[114]	[74]	[34]	[46]	[64]
mPW2PLYP	def2-TZVP	157	230	352	362	457	468	791	1275	1283	1689	3403	3538

to continued ...

Method	Basis set	1	2	3	4	5	6	7	8	9	10	11	12
		[0]	[28]	[30]	[25]	[7]	[1]	[73]	[178]	[105]	[38]	[48]	[69]
mPW2PLYP	def2-QZVP	161	231	350	359	459	472	785	1269	1276	1686	3416	3606
		[0]	[28]	[30]	[24]	[6]	[1]	[64]	[159]	[105]	[32]	[44]	[64]
mPW2PLYP	LANL2DZ	137	229	328	336	468	473	865	1334	1342	1739	3401	3567
		[0]	[24]	[26]	[24]	[15]	[4]	[80]	[310]	[195]	[74]	[43]	[85]
mPW2PLYP	LANL2TZ/def2-TZVP	154	229	346	356	454	464	806	1284	1291	1689	3402	3536
		[0]	[28]	[31]	[25]	[7]	[1]	[76]	[175]	[114]	[41]	[51]	[75]
mPW2PLYP	ZORA-TZVP/TZVP	158	231	353	363	461	473	792	1273	1280	1688	3401	3535
		[0]	[27]	[29]	[24]	[6]	[1]	[70]	[169]	[105]	[35]	[41]	[68]
mPW2PLYP	ZORA-TZVPP/TZVP	158	232	353	363	461	473	791	1273	1280	1689	3400	3535
		[0]	[27]	[29]	[24]	[6]	[1]	[70]	[169]	[105]	[35]	[42]	[64]
mPW2PLYP	DKH-TZVP/TZVP	164	284	345	357	458	461	866	1328	1342	1642	3379	3561
		[0]	[28]	[32]	[30]	[0]	[3]	[53]	[144]	[102]	[52]	[47]	[45]
mPW2PLYP	DKH-TZVPP/TZVP	165	288	344	356	451	457	872	1334	1349	1642	3377	3558
		[0]	[27]	[32]	[31]	[0]	[3]	[51]	[141]	[100]	[53]	[48]	[43]
mPW2PLYP	Sappo-DZP/DZP	156	235	350	361	458	470	790	1262	1270	1667	3389	3532
		[0]	[28]	[31]	[26]	[7]	[1]	[79]	[120]	[78]	[37]	[47]	[80]
mPW2PLYP	Sappo-TZP/DZP	158	236	351	362	464	478	789	1261	1268	1665	3390	3531

to continued ...

Method	Basis set	1	2	3	4	5	6	7	8	9	10	11	12
		[0]	[28]	[30]	[25]	[5]	[0]	[71]	[122]	[79]	[35]	[44]	[68]
mPW2PLYP	Sappo-QZP/DZP	158	237	350	361	466	480	792	1263	1270	1664	3390	3532
		[0]	[28]	[30]	[25]	[5]	[0]	[70]	[121]	[79]	[35]	[45]	[64]
PBE0-DH	def2-TZVP	161	238	363	373	475	488	798	1279	1287	1693	3441	3590
		[0]	[31]	[31]	[25]	[5]	[0]	[85]	[192]	[114]	[39]	[58]	[79]
PBE0-DH	def2-QZVP	164	239	360	370	478	492	794	1273	1281	1690	3449	3600
		[0]	[30]	[30]	[23]	[5]	[0]	[75]	[173]	[115]	[34]	[55]	[72]
PBE0-DH	LANL2DZ	141	239	339	348	497	502	886	1355	1364	1752	3439	3619
		[0]	[25]	[28]	[25]	[14]	[3]	[89]	[332]	[213]	[78]	[61]	[92]
PBE0-DH	LANL2TZ/def2-TZVP	159	238	360	370	476	487	813	1286	1294	1693	3441	3589
		[0]	[31]	[31]	[25]	[6]	[0]	[87]	[189]	[125]	[43]	[59]	[80]
PBE0-DH	ZORA-TZVP/TZVP	161	239	363	373	479	493	799	1277	1285	1693	3441	3588
		[0]	[29]	[29]	[24]	[4]	[0]	[81]	[185]	[117]	[37]	[50]	[74]
PBE0-DH	ZORA-TZVPP/TZVP	161	240	363	374	479	493	799	1277	1285	1693	3440	3588
		[0]	[30]	[29]	[24]	[4]	[0]	[82]	[186]	[117]	[36]	[54]	[73]
PBE0-DH	DKH-TZVP/TZVP	168	299	354	366	475	482	887	1344	1361	1643	3422	3618
		[0]	[30]	[32]	[30]	[0]	[2]	[55]	[155]	[111]	[57]	[50]	[47]
PBE0-DH	DKH-TZVPP/TZVP	168	306	353	364	462	478	898	1358	1377	1644	3418	3615

to continued ...

Method	Basis set	1	2	3	4	5	6	7	8	9	10	11	12
		[0]	[29]	[33]	[33]	[0]	[2]	[46]	[151]	[104]	[61]	[46]	[42]
PBE0-DH	Sappo-DZP/DZP	159	243	361	372	473	487	798	1269	1277	1675	3425	3585
		[0]	[27]	[32]	[26]	[5]	[0]	[92]	[142]	[91]	[40]	[60]	[100]
PBE0-DH	Sappo-TZP/DZP	160	243	361	372	480	495	797	1269	1276	1673	3427	3585
		[0]	[31]	[31]	[25]	[4]	[0]	[83]	[143]	[92]	[38]	[57]	[88]
PBE0-DH	Sappo-QZP/DZP	160	245	360	371	481	496	799	1270	1277	1672	3427	3585
		[0]	[31]	[30]	[24]	[4]	[0]	[81]	[142]	[93]	[38]	[56]	[89]
PBE-QIDH	def2-TZVP	163	242	371	380	488	500	810	1291	1299	1699	3446	3593
		[0]	[31]	[31]	[24]	[5]	[0]	[84]	[192]	[117]	[37]	[48]	[84]
PBE-QIDH	def2-QZVP	169	243	368	378	490	505	800	1280	1288	1695	3456	3609
		[0]	[30]	[30]	[23]	[5]	[0]	[74]	[171]	[114]	[34]	[50]	[80]
PBE-QIDH	LANL2DZ	141	237	341	349	492	497	884	1365	1374	1761	3449	3622
		[0]	[26]	[28]	[25]	[15]	[4]	[87]	[331]	[215]	[79]	[43]	[97]
PBE-QIDH	LANL2TZ/def2-TZVP	160	240	363	373	484	495	824	1300	1307	1699	3445	3592
		[0]	[31]	[32]	[25]	[5]	[0]	[87]	[189]	[127]	[43]	[50]	[84]
PBE-QIDH	ZORA-TZVP/TZVP	163	242	371	381	490	503	806	1285	1293	1699	3446	3592
		[0]	[30]	[30]	[24]	[4]	[0]	[80]	[183]	[116]	[38]	[41]	[78]
PBE-QIDH	ZORA-TZVPP/TZVP	164	242	372	382	491	504	805	1285	1293	1659	3444	3591

to continued ...

Method	Basis set	1	2	3	4	5	6	7	8	9	10	11	12
		[0]	[30]	[30]	[24]	[4]	[0]	[80]	[183]	[115]	[35]	[42]	[77]
PBE-QIDH	DKH-TZVP/TZVP	-	-	-	-	-	-	-	-	-	-	-	-
		-											
PBE-QIDH	DKH-TZVPP/TZVP	171	312	361	370	469	492	913	1377	1400	1647	3431	3553
	-	[0]	[29]	[33]	[32]	[0]	[2]	[29]	[148]	[96]	[52]	[38]	[43]
PBE-QIDH	Sappo-DZP/DZP	162	245	367	377	482	496	805	1279	1287	1683	3440	3595
	-	[0]	[30]	[33]	[26]	[6]	[0]	[88]	[139]	[90]	[41]	[47]	[98]
PBE-QIDH	Sappo-TZP/DZP	163	246	368	379	489	504	803	1277	1285	1681	3441	3595
		[0]	[30]	[31]	[25]	[4]	[0]	[80]	[141]	[91]	[40]	[44]	[88]
PBE-QIDH	Sappo-QZP/DZP	164	248	368	379	491	506	806	1278	1286	1680	3441	3595
		[0]	[31]	[31]	[24]	[4]	[0]	[78]	[139]	[91]	[39]	[44]	[86]
DSD-BLYP	def2-TZVP	162	238	367	376	482	492	809	1285	1294	1691	3406	3548
		[0]	[29]	[30]	[24]	[6]	[1]	[76]	[179]	[109]	[36]	[40]	[80]
DSD-BLYP	def2-QZVP	167	239	364	374	484	497	796	1272	1280	1685	3420	3569
		[0]	[29]	[30]	[23]	[5]	[0]	[66]	[159]	[106]	[33]	[45]	[78]
DSD-BLYP	LANL2DZ	139	230	334	342	471	476	866	1347	1356	1748	3413	3577
		[0]	[25]	[27]	[24]	[15]	[4]	[81]	[312]	[200]	[75]	[33]	[90]
DSD-BLYP	LANL2TZ/def2-TZVP	157	235	356	365	473	482	822	1296	1303	1691	3406	3547

to continued ...

Method	Basis set	1	2	3	4	5	6	7	8	9	10	11	12
		[0]	[30]	[31]	[25]	[5]	[0]	[79]	[177]	[120]	[42]	[47]	[80]
DSD-BLYP	ZORA-TZVP/TZVP	162	238	368	377	484	495	803	1279	1287	1691	3403	3545
		[0]	[28]	[29]	[23]	[5]	[1]	[71]	[169]	[107]	[38]	[37]	[72]
DSD-BLYP	ZORA-TZVPP/TZVP	163	239	369	379	484	497	802	1279	1286	1652	3401	3544
		[0]	[29]	[29]	[24]	[5]	[1]	[71]	[169]	[106]	[35]	[37]	[71]
DSD-BLYP	DKH-TZVP/TZVP	168	298	359	369	474	488	886	1350	1368	1642	3389	3512
		[0]	[30]	[32]	[29]	[0]	[3]	[38]	[141]	[93[]	[61]	[37]	[40]
DSD-BLYP	DKH-TZVPP/TZVP	170	304	359	368	465	484	896	1359	1378	1642	3386	3508
		[0]	[29]	[32]	[30]	[0]	[3]	[34]	[138]	[90[]	[59]	[37]	[40]
DSD-BLYP	Sappo-DZP/DZP	160	241	362	372	476	489	802	1271	1279	1673	3404	3552
		[0]	[29]	[32]	[26]	[7]	[1]	[79]	[121]	[78]	[38]	[41]	[77]
DSD-BLYP	Sappo-TZP/DZP	163	243	365	375	483	498	800	1268	1275	1670	3405	3606
		[0]	[29]	[30]	[24]	[5]	[0]	[71]	[122]	[79]	[37]	[38]	[70]
DSD-BLYP	Sappo-QZP/DZP	163	245	365	376	486	500	803	1269	1276	1669	3404	3606
		[0]	[29]	[30]	[24]	[5]	[0]	[69]	[121]	[79]	[37]	[38]	[70]
RI-SCS-MP2	def2-TZVP	164	237	371	379	485	494	808	1301	1309	1690	3385	3516
		[0]	[29]	[34]	[26]	[6]	[1]	[72]	[172]	[104]	[38]	[29]	[71]
RI-SCS-MP2	def2-QZVP	-	-	-	-	-	-	-	-	-	-	-	-

to continued ...

Method	Basis set	1	2	3	4	5	6	7	8	9	10	11	12
		-	-	-	-	-	-	-	-	-	-	-	-
RI-SCS-MP2	LANL2DZ	140	225	334	341	458	462	854	1351	1360	1744	3386	3537
		[0]	[25]	[29]	[24]	[16]	[5]	[78]	[292]	[189]	[72]	[21]	[71]
RI-SCS-MP2	LANL2TZ/def2-TZVP	157	235	353	362	471	478	824	1312	1319	1690	3387	3515
		[0]	[30]	[36]	[28]	[5]	[0]	[75]	[170]	[115]	[42]	[29]	[75]
RI-SCS-MP2	ZORA-TZVP/TZVP	164	237	372	380	486	496	803	1292	1300	1691	3383	3514
		[0]	[28]	[33]	[25]	[6]	[1]	[68]	[164]	[102]	[37]	[27]	[68]
RI-SCS-MP2	ZORA-TZVPP/TZVP	165	238	374	383	487	499	802	1291	1299	1690	3382	3514
		[0]	[28]	[32]	[25]	[6]	[1]	[68]	[164]	[102]	[36]	[27]	[68]
RI-SCS-MP2	DKH-TZVP/TZVP	165	299	366	369	470	498	817	1380	1407	1674	3395	3512
		[1]	[30]	[35]	[27]	[0]	[3]	[18]	[126]	[77]	[45]	[12]	[47]
RI-SCS-MP2	DKH-TZVPP/TZVP	165	305	366	368	463	492	818	1390	1415	1677	3395	3508
		[1]	[30]	[34]	[29]	[0]	[3]	[18]	[124]	[74]	[51]	[11]	[46]
RI-SCS-MP2	Sappo-DZP/DZP	162	241	364	373	475	487	802	1288	1297	1678	3400	3582
		[0]	[28]	[36]	[28]	[7]	[1]	[73]	[113]	[71]	[38]	[26]	[62]
RI-SCS-MP2	Sappo-TZP/DZP	165	243	370	379	484	496	800	1283	1291	1676	3399	3581
		[0]	[28]	[34]	[26]	[5]	[1]	[65]	[115]	[72]	[36]	[23]	[60]
RI-SCS-MP2	Sappo-QZP/DZP	166	246	371	381	487	501	804	1285	1293	1675	38	3581

to continued ...

Method	Basis set	1	2	3	4	5	6	7	8	9	10	11	12
		[0]	[28]	[33]	[25]	[5]	[1]	[6393]	[114]	[73]	[36]	[23]	[59]
HF	def2-TZVP	158	232	340	351	424	441	816	1390	1399	1808	3646	3751
		[0]	[20]	[48]	[40]	[7]	[1]	[88]	[246]	[162]	[49]	[37]	[87]
HF	def2-QZVP	157	235	336	346	427	444	817	1391	1400	1809	3655	3761
		[0]	[14]	[47]	[38]	[6]	[1]	[79]	[225]	[158]	[42]	[37]	[80]
HF	LANL2DZ	142	235	323	332	450	462	911	1459	1471	1861	3643	3788
		[0]	[20]	[38]	[35]	[15]	[5]	[102]	[391]	[278]	[100]	[47]	[109]
HF	LANL2TZ/def2-TZVP	158	234	336	348	429	444	831	1397	1405	1808	3645	3749
		[0]	[23]	[48]	[40]	[7]	[1]	[94]	[239]	[168]	[52]	[38]	[92]
HF	ZORA-TZVP/TZVP	159	234	339	350	428	445	819	1391	1400	1808	3646	3751
		[0]	[19]	[45]	[38]	[6]	[1]	[84]	[238]	[162]	[47]	[33]	[87]
HF	ZORA-TZVPP/TZVP	159	235	339	350	428	444	818	1391	1400	1808	3645	3750
		[0]	[18]	[46]	[38]	[6]	[1]	[84]	[238]	[162]	[47]	[33]	[87]
HF	DKH-TZVP/TZVP	157	277	337	346	430	446	883	1460	1469	1785	3663	3757
		[1]	[26]	[47]	[36]	[0]	[4]	[23]	[216]	[115]	[78]	[26]	[101]
HF	DKH-TZVPP/TZVP	157	279	336	345	425	443	844	1464	1473	1787	3662	3755
		[1]	[26]	[47]	[36]	[0]	[4]	[33]	[215]	[115]	[78]	[31]	[116]
HF	Sappo-DZP/DZP	-	-	-	-	-	-	-	-	-	-	-	-

to continued ...

Method	Basis set	1	2	3	4	5	6	7	8	9	10	11	12
		-	-	-	-	-	-	-	-	-	-	-	-
HF	Sappo-TZP/DZP	157	237	337	349	427	444	817	1388	1396	1793	3642	3760
		[0]	[22]	[48]	[39]	[6]	[1]	[82]	[197]	[137]	[49]	[49]	[89]
HF	Sappo-QZP/DZP	157	238	336	348	431	448	820	1390	1398	1792	3642	3761
		[0]	[23]	[47]	[38]	[6]	[1]	[80]	[195]	[137]	[49]	[49]	[89]
BP86	Sappo-TZP/DZP	148	230	333	345	450	463	710	1182	1188	1541	3179	3435
		[0]	[30]	[24]	[23]	[4]	[0]	[12]	[88]	[59]	[29]	[160]	[46]
												$MAD_{withNH3}$	$65cm^{-1}$
												MAD_{notNH3}	$31cm^{-1}$
PBE	Sappo-TZP/DZP	149	231	335	347	452	466	710	1179	1185	1537	3187	3445
		[0]	[30]	[23]	[22]	[3]	[0]	[11]	[87]	[60]	[29]	[160]	[47]
												$MAD_{withNH3}$	$66cm^{-1}$
												MAD_{notNH3}	$31cm^{-1}$
B3P86	Sappo-TZP/DZP	153	235	345	357	462	476	780	1231	1238	1632	3320	3489
		[0]	[30]	[28]	[24]	[4]	[0]	[82]	[122]	[79]	[33]	[95]	[67]
												$MAD_{withNH3}$	$55cm^{-1}$
												MAD_{notNH3}	$30cm^{-1}$
B3PW91	Sappo-TZP/DZP	153	234	345	356	458	472	732	1233	1240	1638	3338	3503

to continued ...

Method	Basis set	1	2	3	4	5	6	7	8	9	10	11	12
		[0]	[28]	[28]	[24]	[4]	[0]	[15]	[122]	[78]	[34]	[91]	[69]
												$MAD_{withNH3}$	$63cm^{-1}$
												MAD_{notNH3}	$31cm^{-1}$
HF-by-MMFF94	LANL2DZ/6-31G(d)	152	231	327	338	427	442	862	1461	1470	1854	3662	3778
		[0]	[20]	[43]	[38]	[10]	[4]	[95]	[258]	[174]	[73]	[56]	[89]
												$MAD_{withNH3}$	$148cm^{-1}$
												MAD_{notNH3}	$36cm^{-1}$

end.

Mean Absolute Deviation (MAD) calculated with all methods and basis sets for harmonic vibrational frequencies in the gas phase

PtH

Level / basis set	RECP def2-TZVP	RECP def2-QZVP	RECP LANL2DZ	RECP LANL2TZ/ def2-TZVP	RAE ZORA-TZVP/ TZVP	RAE ZORA-TZVPP/ TZVP	RAE DKH-TZVP/ TZVP	RAE DKH-TZVPP/ TZVP	RAEPt-NRAE Sappo-DZP/ DZP	RAEPt-NRAE Sappo-TZP/ DZP	RAEPt-NRAE Sappo-QZP/ DZP
M06L	40.9	49.7	89.4	34.8	12.84	5.5	2.8	3.4	30.1	48.2	46.4
B3LYP	39.0	33.6	92.3	8.2	4.29	0.1	8.1	12.4	23.5	36.4	33.9
mPW1PW	11.9	13.9	60.8	32.9	38.82	43.5	51.1	55.6	22.9	9.1	8.9
PBE0	10.9	13.2	63.4	32.8	35.71	40.3	48.1	52.6	20.0	6.5	5.5
TPSSh	6.9	6.6	70.7	17.1	20.14	24.2	32.5	36.5	3.8	10.3	11.1
M06	103.6	111.0	149.4	128.7	123.17	118.9	112.0	108.1	138.7	162.6	162.1
M062X	91.8	109.1	23.9	59.3	105.57	115.5	117.3	127.5	60.5	58.0	63.8
LC-BLYP	13.6	14.8	46.0	44.9	44.95	49.8	55.2	60.1	30.3	16.4	18.3
CAM-B3LYP	13.8	12.1	70.5	21.7	18.86	23.4	29.9	36.4	1.2	13.6	11.3
ωB97X-D3BJ	47.2	53.3	110.6	44.2	21.41	21.6	15.6	15.8	22.4	37.2	42.0
B97-3c	10.1	17.8	59.9	43.9	37.66	42.8	52.5	57.4	825.7	1196.6	1063.1
B2PLYP	39.8	47.0	116.8	40.8	70.05	77.7	82.3	89.6	68.9	36.7	45.9
mPW2PLYP	32.6	38.8	114.8	43.2	65.25	72.6	77.5	84.5	62.4	35.8	39.8
PBE0-DH	not opt	45.6	80.1	60.6	65.92	71.9	77.8	83.3	59.2	36.8	36.6
PBE-QIDH	not opt	103.8	97.6	90.7	121.52	130.1	133.6	141.7	125.2	93.3	95.9
DSD-BLYP	133.1	142.0	115.0	96.7	162.00	172.9	174.9	185.0	172.4	134.0	140.4
RI-SCS-MP2	239.7	not opt	126.4	149.9	250.15	261.9	264.4	275.0	274.8	216.3	227.1
HF	228.7	247.5	300.0	64.6	212.08	210.7	204.6	203.2	229.1	258.5	258.3
Minor MAD	6.9 TPSSh	6.6 TPSSh	23.9 M062X	8.2 B3LYP	4.30 B3LYP	0.1 B3LYP	8.1 B3LYP	3.4 M06L	1.2 CAM-B3LYP	6.5 PBE0	5.5 PBE0

meta-GGA
GGA hybrid
meta-GGA hybrid
range-separated hybrid
GGA composite
Double hybrid
Hartree-Fock
Post-Hartree Fock

Figure S6: Calculated MAD (in cm^{-1}) for harmonic vibrational frequencies of PtH relative to Infrared spectrum with all methods and basis sets in the gas phase. MADs were calculated from wavenumbers reported in Table S5. MADs with values lower or equal to 10 are colored dark green, and those higher than 10 vary toward dark red. Abbreviated names for the basis sets are used here, with their full names listed in Table 1. not opt: not geometric optimization.

PtCl

Level / basis set	RECP def2-TZVP	RECP def2-QZVP	RECP LANL2DZ	RECP LANL2TZ/ def2-TZVP	RAE ZORA-TZVP/ TZVP	RAE ZORA-TZVPP/ TZVP	RAE DKH-TZVP/ TZVP	RAE DKH-TZVPP/ TZVP	RAEPt-NRAE Sappo-DZP/ DZP	RAEPt-NRAE Sappo-TZP/ DZP	RAEPt-NRAE Sappo-QZP/ DZP
M06L	26.14	22.83	60.58	1.74	not opt	13.20	not opt	17.58	29.81	23.53	24.98
B3LYP	27.53	27.77	60.87	15.04	19.19	16.25	24.75	22.82	31.18	22.99	25.38
mPW1PW	10.05	10.45	49.85	1.21	5.78	4.84	10.15	9.99	16.36	10.42	13.00
PBE0	8.37	8.71	48.55	3.43	4.50	3.57	8.94	8.80	16.85	9.46	12.28
TPSSh	13.13	13.22	52.09	1.67	8.87	8.01	13.10	13.01	18.51	13.13	15.38
M06	24.77	24.96	54.67	not opt	25.80	25.41	29.54	29.84	30.44	27.89	27.96
M062X	31.54	33.95	54.67	23.31	31.79	30.88	36.66	36.39	46.76	38.81	46.23
LC-BLYP	2.82	1.18	32.16	15.85	6.95	8.09	2.11	2.40	0.39	4.51	1.76
CAM-B3LYP	15.01	16.27	48.47	48.47	10.66	9.71	15.02	14.78	18.93	14.43	16.84
ωB97X-D3BJ	13.69	12.92	46.50	2.98	8.91	6.47	14.72	13.37	20.29	13.49	16.17
B97-3c	9.80	4.87	60.17	not opt	14.84	2.09	not opt	5.92	34.96	not opt	33.30
B2PLYP	13.24	7.57	65.11	6.33	5.07	1.58	10.81	8.49	22.82	9.77	10.41
mPW2PLYP	16.36	11.53	64.90	5.64	8.12	4.76	13.84	11.65	24.70	12.34	13.34
PBE0-DH	2.03	2.25	49.05	7.96	2.37	0.46	3.40	2.93	11.93	4.43	6.20
PBE-QIDH	4.66	1.53	60.17	10.39	1.04	4.36	5.13	3.10	18.50	4.13	4.59
DSD-BLYP	5.75	3.11	67.23	6.03	2.44	6.69	3.85	0.98	19.06	1.67	1.11
RI-SCS-MP2	11.24	not opt	73.40	11.97	3.94	0.03	10.58	8.01	27.11	5.94	4.19
HF	71.02	73.73	89.97	42.38	70.59	69.75	74.03	73.70	75.05	75.17	76.13
Minor MAD	2.03<br>PBE0-DH	1.18<br>LC-BLYP	32.16<br>LC-BLYP	1.21<br>mPW1PW	1.04<br>PBE-QIDH	**0.03**<br>**RI-SCS-MP2**	2.11<br>LC-BLYP	0.98<br>DSD-BLYP	0.39<br>LC-BLYP	1.67<br>DSD-BLYP	1.11<br>DSD-BLYP

meta-GGA
GGA hybrid
meta-GGA hybrid
range-separated hybrid
GGA composite
Double hybrid
Hartree-Fock
Post-Hartree Fock

Figure S7: Calculated MAD (in cm^{-1}) for harmonic vibrational frequencies of PtCl relative to Infrared spectrum with all methods and basis sets in the gas phase. MADs were calculated from wavenumbers reported in Table S5. MADs with values lower or equal to 5 are colored dark green, and those higher than 5 vary toward dark red. Abbreviated names for the basis sets are used here, with their full names listed in Table 1. not opt: not geometric optimization.

$[PtCl_4]^{2-}$

Level / basis set	RECP: def2-TZVP	RECP: def2-QZVP	RECP: LANL2DZ	RECP: LANL2TZ/ def2-TZVP	RAE: ZORA-TZVP/ TZVP	RAE: ZORA-TZVPP/ TZVP	RAE: DKH-TZVP/ TZVP	RAE: DKH-TZVPP/ TZVP	RAEPt-NRAE: Sappo-DZP/ DZP	RAEPt-NRAE: Sappo-TZP/ DZP	RAEPt-NRAE: Sappo-QZP/ DZP
**M06L**	49.7	49.7	56.2	49.6	49	49.3	49.1	49.5	49.2	49.6	50.5
**B3LYP**	45.7	46.5	51.2	46.6	47	46.9	47.0	46.9	44.5	44.9	45.2
**mPW1PW**	36.0	36.3	42.5	36.7	37	36.6	36.8	36.7	35.2	35.5	36.0
**PBE0**	35.1	35.4	41.9	35.8	36	35.8	36.1	36.0	34.5	34.8	35.3
**TPSSh**	39.6	39.7	45.8	40.8	41	40.5	40.7	40.6	38.5	38.8	39.3
**M06**	41.4	42.2	47.8	42.7	41	41.2	41.2	41.4	39.4	39.7	39.9
**M062X**	55.7	58.8	50.0	55.1	56	56.5	56.2	56.6	51.9	55.5	55.9
**LC-BLYP**	27.6	28.4	34.4	28.2	28	28.2	28.5	28.4	25.7	26.1	26.6
**CAM-B3LYP**	36.3	37.1	42.3	37.1	37	37.2	37.4	37.3	34.9	35.4	35.8
**ωB97X-D3BJ**	29.6	30.1	35.5	30.2	30	30.2	30.4	30.3	28.4	29.2	29.7
**B97-3c**	44.8	44.8	51.1	45.3	45	44.9	45.0	45.1	not opt	not opt	not opt
**B2PLYP**	35.3	34.2	46.1	39.8	37	36.8	37.0	36.7	35.8	35.0	34.7
**mPW2PLYP**	33.7	33.1	44.9	38.0	36	35.8	35.5	35.7	34.2	35.3	33.8
**PBE0-DH**	27.6	26.1	37.1	29.0	29	29.0	29.2	29.2	28.5	28.9	28.0
**PBE-QIDH**	22.0	18.9	36.9	27.4	25	24.3	24.8	24.5	24.7	23.1	22.9
**DSD-BLYP**	25.5	21.2	41.8	33.1	27	26.5	27.5	26.6	27.5	26.8	25.9
**RI-SCS-MP2**	22.8	not opt	41.7	34.5	25	24.3	25.2	24.4	26.8	24.7	21.5
**HF**	41.5	43.6	45.6	43.6	43	43.4	43.3	43.4	41.5	42.8	42.8
**Minor MAD**	22.0 PBE-QIDH	**18.9 PBE-QIDH**	34.4 LC-BLYP	27.4 PBE-QIDH	25 PBE-QIDH	24.3 RI-SCS-MP2	24.8 PBE-QIDH	24.0 RI-SCS-MP2	24.7 PBE-QIDH	23.1 PBE-QIDH	21.5 RI-SCS-MP2

meta-GGA
GGA hybrid
meta-GGA hybrid
range-separated hybrid
GGA composite
Double hybrid
Hartree-Fock
Post-Hartree Fock

Figure S8: Calculated MAD (in cm^{-1}) for harmonic vibrational frequencies of $[PtCl_4]^{2-}$ relative to Infrared spectrum with all methods and basis sets in the gas phase. Three vibrational modes were selected for MADs calculations. MADs were calculated from wavenumbers reported in Table S6. MADs with values lower or equal to 5 are colored dark green, and those higher than 5 vary toward dark red. Abbreviated names for the basis sets are used here, with their full names listed in Table 1. not opt: not geometric optimization.

$[Pt(NH_3)_4]^{2+}$

	RECP				RAE				RAEPt-NRAE		
Level / basis set	def2-TZVP	def2-QZVP	LANL2DZ	LANL2TZ/ def2-TZVP	ZORA-TZVP/ TZVP	ZORA-TZVPP/ TZVP	DKH-TZVP/ TZVP	DKH-TZVPP/ TZVP	Sappo-DZP/ DZP	Sappo-TZP/ DZP	Sappo-QZP/ DZP
M06L	124.2	not opt	not opt	122.4	123.8	123.9	not opt	not opt	not opt	not opt	not opt
B3LYP	114.4	117.1	151.1	116.0	114.9	114.8	not opt	not opt	96.2	96.9	97.0
mPW1PW	138.4	139.2	181.1	139.6	138.4	138.3	not opt	not opt	120.3	120.2	121.0
PBE0	132.7	133.9	175.9	134.1	132.8	132.8	not opt	not opt	116.3	116.3	116.8
TPSSh	116.3	115.7	154.3	117.6	116.0	116.1	not opt	not opt	92.2	92.7	92.8
M06	109.3	not opt	155.7	106.8	111.4	111.3	not opt	not opt	100.6	101.6	101.9
M062X	127.2	131.1	168.3	129.0	127.0	127.1	not opt	not opt	111.3	111.6	not opt
LC-BLYP	92.5	94.5	136.9	93.7	92.5	92.7	not opt	not opt	74.9	75.8	76.0
CAM-B3LYP	130.3	132.6	171.1	131.8	130.7	130.6	not opt	not opt	112.1	111.7	112.5
ωB97X-D3BJ	141.8	143.4	185.1	143.1	141.9	141.9	not opt	not opt	125.1	not opt	125.6
B97-3c	103.5	104.4	140.7	104.8	105.3	105.4	not opt	not opt	209.3	197.8	211.9
B2PLYP	127.8	not opt	162.4	128.2	126.1	126.1	not opt	not opt	not opt	112.8	113.3
mPW2PLYP	138.4	not opt	175.4	139.1	137.2	137.2	not opt	not opt	not opt	123.5	not opt
PBE0-DH	163.1	not opt	not opt	164.1	162.4	162.4	not opt	not opt	150.1	149.8	150.4
PBE-QIDH	167.6	not opt	209.2	168.1	165.7	not opt	not opt	not opt	157.0	156.3	not opt
DSD-BLYP	167.6	not opt	209.2	145.9	143.5	143.2	not opt	not opt	134.9	not opt	not opt
RI-SCS-MP2	133.1	not opt	156.8	131.8	130.3	130.1	not opt	not opt	not opt	not opt	not opt
HF	263.8	267.5	307.7	265.6	263.9	263.8	331.7	not opt	256.8	not opt	256.9
Minor MAD	92.5	94.5	136.9	93.7	92.5	92.7	331.7		**74.9**	75.8	76.0
	LC-BLYP	LC-BLYP	LC-BLYP	LC-BLYP	LC-BLYP	LC-BLYP	HF	-	**LC-BLYP**	LC-BLYP	LC-BLYP

- meta-GGA
- GGA hybrid
- meta-GGA hybrid
- range-separated hybrid
- GGA composite
- Double hybrid
- Hartree-Fock
- Post-Hartree Fock

Figure S9: Calculated MAD (in cm^{-1}) for harmonic vibrational frequencies of $[Pt(NH_3)_4]^{2+}$ relative to Infrared spectrum with all methods and basis sets in the gas phase. Five vibrational modes were selected for MADs calculations. MADs were calculated from wavenumbers reported in Table S7 of Supporting Information. MADs with values lower or equal to 100 are colored dark green, and those higher than 100 vary toward dark red. Abbreviated names for the basis sets are used here, with their full names listed in Table 1. not opt: not geometric optimization.

Cisplatin [$Pt(NH_3)_2Cl_2$]

	RECP				RAE				RAEPt-NRAE		
Level / basis set	def2-TZVP	def2-QZVP	LANL2DZ	LANL2TZ/ def2-TZVP	ZORA-TZVP/ TZVP	ZORA-TZVPP/ TZVP	DKH-TZVP/ TZVP	DKH-TZVPP/ TZVP	Sappo-DZP/ DZP	Sappo-TZP/ DZP	Sappo-QZP/ DZP
M06L	68.1	75.5	60.9	69.3	64.4	64.5	56.3	56.4	69.6	68.1	67.5
B3LYP	66.3	67.2	57.4	64.0	67.7	67.5	52.3	52.5	59.8	58.1	57.1
mPW1PW	68.1	68.8	66.9	70.2	70.8	70.4	61.0	64.2	62.9	66.7	60.4
PBE0	71.1	67.0	70.3	68.7	69.3	68.9	61.1	64.2	61.6	59.9	59.1
TPSSh	59.2	60.0	58.4	60.4	57.9	57.7	46.5	47.0	50.7	48.8	48.5
M06	74.6	77.2	61.9	74.2	71.8	71.5	64.0	64.4	71.0	71.0	69.9
M062X	78.8	78.5	74.0	74.4	75.4	75.3	77.4	80.8	69.3	66.8	66.6
LC-BLYP	52.7	53.7	50.6	51.2	55.3	55.2	54.6	58.7	48.5	48.5	47.8
CAM-B3LYP	66.2	67.1	68.4	64.1	64.0	63.8	63.7	66.1	60.3	59.1	58.1
ωB97X-D3BJ	70.2	70.7	76.9	68.5	68.5	68.3	74.7	77.6	65.7	64.3	63.4
B97-3c	63.1	63.3	57.6	62.7	62.3	62.0	49.2	52.5	91.3	92.1	118.7
B2PLYP	not opt	70.2	67.3	60.6	61.9	61.8	60.4	62.7	60.5	59.7	58.6
mPW2PLYP	65.0	72.4	73.4	63.5	64.3	64.1	67.1	69.5	62.8	61.9	60.8
PBE0-DH	70.3	71.6	83.4	69.5	69.7	69.5	80.1	84.7	68.2	66.9	66.4
PBE-QIDH	69.4	71.5	87.8	68.5	69.5	66.0	not opt	86.0	69.2	68.4	67.8
DSD-BLYP	63.2	66.2	78.6	62.0	63.2	59.8	69.6	73.1	63.2	67.3	66.7
RI-SCS-MP2	56.9	not opt	74.8	58.3	57.3	57.3	72.6	75.9	63.7	63.7	63.0
HF	127.3	127.6	147.9	127.9	126.7	126.7	143.0	141.0	not opt	124.7	124.4
Minor MAD	52.7 LC-BLYP	53.7 LC-BLYP	50.6 LC-BLYP	51.2 LC-BLYP	55.3 LC-BLYP	55.0 LC-BLYP	**46.5 TPSSh**	47.0 TPSSh	48.5 LC-BLYP	48.5 LC-BLYP	47.8 LC-BLYP

meta-GGA
GGA hybrid
meta-GGA hybrid
range-separated hybrid
GGA composite
Double hybrid
Hartree-Fock
Post-Hartree Fock

Figure S10: Calculated MAD (in cm^{-1}) for harmonic vibrational frequencies of Cisplatin [$Pt(NH_3)_2Cl_2$] at different methods and basis sets in gas phase. Twelve vibrational modes were selected for MADs calculations. MADs were calculated from wavenumbers reported in Table S8 of Supporting Information. MADs with values lower or equal to 50 are colored dark green, and those higher than 50 vary toward dark red. Abbreviated names for the basis sets are used here, with their full names listed in Table 1. not opt: not geometric optimization.

Cisplatin $[Pt(NH_3)_2Cl_2]$

	RECP				RAE				RAEPt-NRAE		
Level / basis set	def2-TZVP	def2-QZVP	LANL2DZ	LANL2TZ/ def2-TZVP	ZORA-TZVP/ TZVP	ZORA-TZVPP/ TZVP	DKH-TZVP/ TZVP	DKH-TZVPP/ TZVP	Sappo-DZP/ DZP	Sappo-TZP/ DZP	Sappo-QZP/ DZP
**M06L**	42.4	41.8	28.5	41.8	39.9	40.2	38.7	40.4	42.8	40.3	39.8
**B3LYP**	40.0	38.6	25.9	39.5	37.6	37.6	34.9	37.0	37.0	34.2	33.2
**mPW1PW**	34.1	32.4	19.5	33.3	31.8	31.8	35.0	37.9	32.5	35.2	28.7
**PBE0**	33.3	31.6	18.5	32.6	31.1	31.1	34.8	38.0	31.8	28.9	28.0
**TPSSh**	34.2	33.0	21.2	34.0	32.5	32.5	34.2	36.8	32.6	30.0	29.0
**M06**	43.4	41.7	28.9	44.5	40.9	40.7	38.8	41.3	41.0	39.3	37.8
**M062X**	48.5	45.5	29.2	45.9	44.5	44.5	41.4	45.2	43.3	39.2	38.9
**LC-BLYP**	30.0	28.5	15.3	29.5	27.8	27.8	34.5	37.7	25.6	23.7	22.8
**CAM-B3LYP**	35.7	34.0	21.2	35.0	33.4	33.3	35.0	37.5	32.6	30.0	28.9
**ωB97X-D3BJ**	36.2	34.2	21.9	35.2	34.1	34.1	37.3	39.8	34.3	31.5	30.5
**B97-3c**	35.2	34.3	21.9	35.0	33.1	33.0	36.9	40.7	23.5	49.8	62.9
**B2PLYP**	not opt	33.2	27.4	34.3	33.3	33.4	35.3	37.9	32.7	30.7	29.6
**mPW2PLYP**	34.4	32.9	26.4	33.7	32.9	33.1	35.3	37.9	32.3	30.4	29.2
**PBE0-DH**	30.9	29.4	18.4	29.6	29.3	29.3	35.3	38.7	30.0	27.5	26.6
**PBE-QIDH**	29.2	27.6	20.6	27.9	28.4	28.4	not opt	39.3	28.8	27.0	26.0
**DSD-BLYP**	30.5	29.1	26.9	29.4	30.2	30.2	35.5	38.9	29.4	28.3	27.1
**RI-SCS-MP2**	31.4	not opt	31.9	29.6	31.3	31.5	35.3	38.7	30.8	30.3	29.2
**HF**	40.3	37.1	28.0	37.5	38.5	38.6	37.2	38.6	not opt	37.5	35.9
**Minor MAD**	29.2 PBE-QIDH	27.6 PBE-QIDH	**15.3 LC-BLYP**	27.9 PBE-QIDH	27.8 LC-BLYP	27.8 LC-BLYP	34.2 TPSSh	36.8 TPSSh	23.5 B97-3c	23.7 LC-BLYP	22.8 LC-BLYP

Legend: meta-GGA; GGA hybrid; meta-GGA hybrid; range-separated hybrid; GGA composite; Double hybrid; Hartree-Fock; Post-Hartree Fock

Figure S11: Calculated MAD (in cm^{-1}) for harmonic vibrational frequencies excluding NH_3 modes for Cisplatin $[Pt(NH_3)_2Cl_2]$ at different methods and basis sets in gas phase. Six harmonic vibrational modes were selected for MADs calculations. MADs were calculated from wavenumbers reported in Table S8. MADs with values lower or equal to 20 are colored dark green, and those higher than 20 vary toward dark red. Abbreviated names for the basis sets are used here, with their full names listed in Table 1. not opt: not geometric optimization.

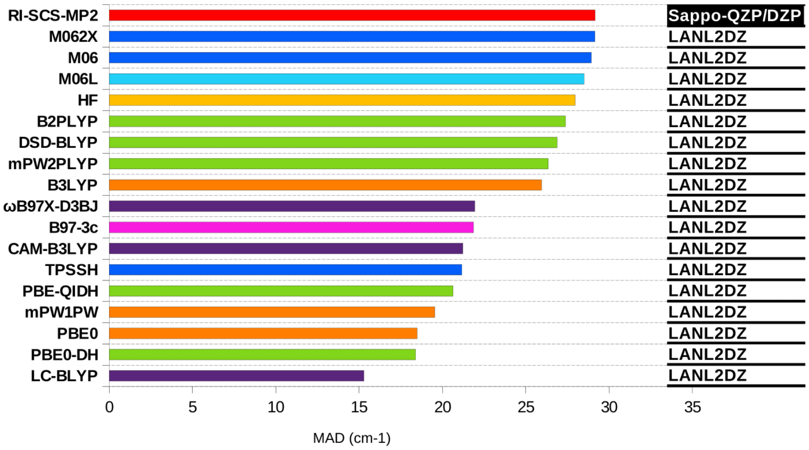

Figure S12: Calculated MAD (in cm^{-1}) excluding NH_3 modes for cisplatin in the gas phase. Six harmonic vibrational modes with Infrared Activity considered. This is a selection from each method, along with its most optimal representative basis set, resulting from calculations at all levels of theory for predicting harmonic vibrational frequencies. MADs values were compared with experimental reference.[8] All MAD values are shown in **Figure S11** of the Supporting Information. All the color bars in this Figure follow the same description for the DFT methods shown in Figure 4. In this figure we used short name for basis sets, full name are in Table 1.

Structural parameters for cisplatin polymorphs in the solid phase

α-cisplatin

Table S9: Experimental and calculated bond lengths (Å) and angles (°) for α-cisplatin. Atoms are numbered according to Figure 5.

Bond/Angle	*Expt.*[1]	*Calc.*	Bond/Angle	*Expt.*[1]	*Calc.*
Pt1–N9	2.047	2.063	N7–Pt1–Cl5	178.37	177.66
Pt1–N7	2.049	2.060	Cl3–Pt1–Cl5	91.65	91.24
Pt1–Cl3	2.321	2.332	Pt1–N7–H11	110.0	108.75
Pt1–Cl5	2.322	2.336	Pt1–N7–H13	110.2	111.47
N7–H11	0.990	1.033	Pt1–N7–H15	115.0	114.13
N7–H13	1.047	1.031	H11–N7–H13	113.3	108.20
N7–H15	0.936	1.030	H11–N7–H15	105.7	106.84
N9–H17	1.046	1.030	H13–N7–H15	102.5	107.21
N9–H19	0.988	1.033	Pt1–N9–H17	113.1	111.18
N9–H21	0.976	1.028	Pt1–N9–H19	109.1	110.20
N9–Pt1–N7	90.62	91.09	Pt1–N9–H21	112.3	111.48
N9–Pt1–Cl3	179.71	179.57	H17–N9–H19	109.0	108.85
N7–Pt1–Cl3	89.18	89.15	H17–N9–H21	112.4	108.34
N9–Pt1–Cl5	88.55	88.54	H19–N9–H21	100.1	106.64

[a]Reference[5]

β-cisplatin

Table S10: Experimental and calculated bond lengths (Å) and angles (°) for β-cisplatin. Atoms are numbered according to Figure 5.

Bond/Angle	*Expt.*[1]	*Calc.*	Bond/Angle	*Expt.*[1]	*Calc.*
Pt1–N15	2.046	2.058	N19–Pt3–Cl9	179.3	177.7
Pt1–N13	2.058	2.061	N17–Pt3–Cl11	178.7	178.6
Pt1–Cl5	2.3180	2.333	N19–Pt3–Cl11	88.1	88.0
Pt1–Cl7	2.3196	2.340	Cl9–Pt3–Cl11	92.05	92.1
Pt3–N17	2.042	2.062	Pt1–N13–H21	101.2	109.5
Pt3–N19	2.050	2.063	Pt1–N13–H23	98.8	111.3
Pt3–Cl9	2.314	2.329	Pt1–N13–H25	120.8	113.9
Pt3–Cl11	2.317	2.335	H21–N13–H23	113	108

to continued ...

Bond/Angle	*Expt.*[1]	*Calc.*	Bond/Angle	*Expt.*[1]	*Calc.*
N13–H21	0.98	1.03	H21–N13–H25	112	107
N13–H23	0.99	1.03	H23–N13–H25	110	107
N13–H25	1.01	1.03	Pt1–N15–H27	121.0	110
N15–H27	0.95	1.03	Pt1–N15–H29	104.0	111
N15–H29	1.08	1.03	Pt1–N15–H31	110.2	112
N15–H31	1.04	1.03	H27–N15–H29	107	109
N17–H33	0.95	1.03	H27–N15–H31	109	109
N17–H35	1.03	1.03	H29–N15–H31	104	106
N17–H37	1.01	1.03	Pt3–N17–H33	113.6	110
N19–H39	1.05	1.03	Pt3–N17–H35	125.3	111
N19–H41	0.91	1.03	Pt3–N17–H37	104.3	114
N19–H43	1.10	1.03	H33–N17–H35	105	106
N15–Pt1–N13	90.5	90.2	H33–N17–H37	106	109
N15–Pt1–Cl7	88.2	88.3	H35–N17–H37	100	107
N13–Pt1–Cl7	178.4	177.6	Pt3–N19–H39	118.4	109
N15–Pt1–Cl5	179.8	179.2	Pt3–N19–H41	100.6	112
N13–Pt1–Cl5	89.3	89.4	Pt3–N19–H43	116.8	111
Cl7–Pt1–Cl5	92.0	92.1	H39–N19–H41	120	108
N17–Pt3–N19	90.6	91.2	H39–N19–H43	101	108
N17–Pt3–Cl9	89.2	88.7	H41–N19–H43	99	108

end.

Stability of the wave function for the levels of theory for the open-shell systems PtH and PtH

Table S11: Self Consistent Field (SCF) stability analysis for the open-shell systems PtH, and PtCl. Calculations were with ORCA 5.0.3 quantum program. For scalar relativistic basis sets (such as DKH, ZORA, and Sapporo) we used the one-center approximation. The selected levels of theory correspond to the three best predictors of structural parameters and the three for harmonic vibrational frequencies, as shown in Table 2. Abbreviated names for the basis sets are used here, with their full names listed in Table 1.

	Level of theory	Result	type of functional/basis set
**PtH**	**Best predicting levels for structural parameters**		
	LC-BLYP/def2-TZVP	stable	range-separated/RECP
	TPSSh/def2-QZVP	stable	meta-GGA hybrid/RECP
	CAM-B3LYP/LANL2TZ/def2-TZVP	stable	range-separated/RECP
	**Best predicting levels for frequencies**		
	B3LYP/ZORA-TZVPP/TZVP	stable	GGA hybrid/RAE
	CAM-B3LYP/Sappo-DZP/DZP	stable	range-separated/RAEPt-NRAE
	M06L/DKH-TZVP/TZVP	nonstable	meta-GGA/RAE
**PtCl**	**Best predicting for structural parameters**		
	mPW2PLYP/ZORA-TZVP/TZVP	nonstable	double hybrid/RAE
	TPSSh/LANL2TZ/def2-TZVP	nonstable	meta-GGA hybrid/RECP
	B2PLYP/Sappo-QZP/DZP	stable	double hybrid/ RAEPt-NRAE
	**Best predicting for frequencies**		
	LC-BLYP/Sappo-DZP/DZP	nonstable	
	PBE0-DH/ZORA-TZVPP/TZVP	nonstable	double hybrid/RAE
	PBE-QIDH/ZORA-TZVP/TZVP	nonstable	double hybrid/RAE

Partial atomic charges calculated for Platinum derivatives in the gas phase

Table S12 shows our results for the $[\mathbf{Pt(NH_3)_4}]^{2+}$ complex. We observe positive values for the Pt metal center and H atoms, while N atoms exhibited negative values across all levels

of theory and charge method. The CHELPG and Hirshfeld charges methods reveal higher positive values for the H atoms. In contrast, the Mulliken charges do not show a similar trend across all tested levels.

Table S12: Partial Atomic Charges (e) on all atoms of $[Pt(NH_3)_4]^{2+}$ in the gas phase. The selected levels of theory correspond to the three best predictors of structural parameters and the three for harmonic vibrational frequencies. The level of theory used in the parametrization of the standard MMFF94 force field (HF/LANL2DZ/...) is included. Abbreviated names for the basis sets are used here, with their full names listed in Table 1.

	**3 best predicting structural parameters**			**3 best predicting frequencies**		
	**PBE-QIDH/Sappo-TZP/DZP**			**LC-BLYP/Sappo-DZP/DZP**		
**Atom**	**CHELPG**	**Hirshfeld**	**Mulliken**	**CHELPG**	**Hirshfeld**	**Mulliken**
**Pt**	0.10749	0.14720	0.20432	0.02213	0.16475	0.22500
**N2**	-0.51526	-0.06639	-0.10857	-0.45107	-0.07646	-0.08586
**N3**	-0.52135	-0.06642	-0.10868	-0.45142	-0.07647	-0.08584
**N4**	-0.52264	-0.06643	-0.10868	-0.45762	-0.07646	-0.08594
**N5**	-0.52088	-0.06640	-0.10868	-0.45849	-0.07645	-0.08589
**H6**	0.32842	0.17719	0.18909	0.31512	0.17949	0.18186
**H7**	0.32887	0.17796	0.19170	0.31490	0.17953	0.18202
**H8**	0.33039	0.17796	0.19168	0.31517	0.17953	0.18207
**H9**	0.33395	0.17454	0.17680	0.31775	0.17630	0.16575
**H10**	0.33078	0.17724	0.18936	0.31672	0.17951	0.18204
**H11**	0.33021	0.17719	0.18912	0.31705	0.17952	0.18195
**H12**	0.33321	0.17444	0.17665	0.31838	0.17624	0.16574

...

	3 best predicting structural parameters			3 best predicting frequencies		
**H13**	0.33022	0.17719	0.18925	0.31508	0.17945	0.18173
**H14**	0.33195	0.17447	0.17678	0.31623	0.17624	0.16572
**H15**	0.33031	0.17798	0.19184	0.31697	0.17949	0.18198
**H16**	0.33345	0.17446	0.17660	0.31598	0.17627	0.16574
**H17**	0.33085	0.17781	0.19141	0.31714	0.17951	0.18192

	PBE0-DH/Sappo-QZP/DZP			TPSSh/Sappo-DZP/DZP		
**Atom**	**CHELPG**	**Hirshfeld**	**Mulliken**	**CHELPG**	**Hirshfeld**	**Mulliken**
**Pt**	0.10986	0.15522	0.07396	0.00692	0.16020	0.23830
**N2**	-0.52032	-0.06980	-0.06008	-0.43363	-0.06442	-0.08944
**N3**	-0.52184	-0.06980	-0.06012	-0.43802	-0.06442	-0.08954
**N4**	-0.52535	-0.06981	-0.06017	-0.43910	-0.06445	-0.08954
**N5**	-0.52456	-0.06979	-0.06009	-0.43895	-0.06443	-0.08946
**H6**	0.32990	0.17791	0.18517	0.31042	0.17575	0.18114
**H7**	0.32985	0.17806	0.18553	0.31051	0.17581	0.18125
**H8**	0.33103	0.17802	0.18541	0.31166	0.17576	0.18122
**H9**	0.33381	0.17502	0.17096	0.31311	0.17282	0.16755
**H10**	0.33194	0.17805	0.18550	0.31189	0.17578	0.18120
**H11**	0.33164	0.17802	0.18538	0.31155	0.17578	0.18119

...

	3 best predicting structural parameters			3 best predicting frequencies		
**H12**	0.33390	0.17496	0.17079	0.31334	0.17283	0.16754
**H13**	0.33062	0.17799	0.18539	0.31142	0.17577	0.18115
**H14**	0.33354	0.17504	0.17098	0.31176	0.17283	0.16756
**H15**	0.33104	0.17800	0.18539	0.31204	0.17578	0.18118
**H16**	0.33311	0.17496	0.17079	0.31288	0.17286	0.16756
**H17**	0.33184	0.17794	0.18522	0.31219	0.17577	0.18115

	LC-BLYP/Sappo-QZP/ DZP			B3LYP/Sappo-DZP/DZP		
**Atom**	**CHELPG**	**Hirshfeld**	**Mulliken**	**CHELPG**	**Hirshfeld**	**Mulliken**
**Pt**	0.03957	0.15664	-0.03152	0.01261	0.17508	0.26981
**N2**	-0.46327	-0.07438	-0.00194	-0.42006	-0.06979	-0.06278
**N3**	-0.46407	-0.07438	-0.00193	-0.42227	-0.06981	-0.06288
**N4**	-0.46940	-0.07439	-0.00201	-0.42597	-0.06982	-0.06288
**N5**	-0.47118	-0.07439	-0.00195	-0.42603	-0.06980	-0.06282
**H6**	0.31772	0.17946	0.17592	0.30523	0.17625	0.16967
**H7**	0.31721	0.17953	0.17610	0.30551	0.17635	0.16988
**H8**	0.31763	0.17949	0.17613	0.30597	0.17628	0.16980
**H9**	0.32046	0.17622	0.15780	0.30814	0.17342	0.15582
**H10**	0.31902	0.17949	0.17603	0.30708	0.17632	0.16984

...

	3 best predicting structural parameters			3 best predicting frequencies		
**H11**	0.31975	0.17950	0.17603	0.30686	0.17629	0.16975
**H12**	0.32132	0.17625	0.15782	0.30833	0.17343	0.15581
**H13**	0.31778	0.17945	0.17587	0.30599	0.17629	0.16977
**H14**	0.31914	0.17623	0.15782	0.30680	0.17345	0.15585
**H15**	0.31951	0.17947	0.17594	0.30733	0.17630	0.16981
**H16**	0.31915	0.17628	0.15784	0.30720	0.17346	0.15582
**H17**	0.31967	0.17952	0.17604	0.30730	0.17629	0.16972

	Standard MMFF94 force field		
	HF/LANL2DZ/6-31G(d)		
**Atom**	**CHELPG**	**Hirshfeld**	**Mulliken**
**Pt**	0.36871	0.31717	1.09469
**N2**	-0.61393	-0.08562	-1.18613
**N3**	-0.60539	-0.08563	-1.18595
**N4**	-0.62537	-0.08563	-1.18603
**N5**	-0.60649	-0.08565	-1.18584
**H6**	0.33804	0.16948	0.47169
**H7**	0.33888	0.17012	0.47408
**H8**	0.33739	0.17009	0.47387

...

	3 best predicting structural parameters			3 best predicting frequencies
**H9**	0.34704	0.16673	0.46652	
**H10**	0.34170	0.16955	0.47193	
**H11**	0.33642	0.16954	0.47193	
**H12**	0.34222	0.16667	0.46635	
**H13**	0.33616	0.16957	0.47212	
**H14**	0.34411	0.16676	0.46656	
**H15**	0.33712	0.17010	0.47398	
**H16**	0.34144	0.16667	0.46633	
**H17**	0.34197	0.17008	0.47391	

end.

Tables S13 and **S14** present the calculated charges for the small-sized compounds, PtH and PtCl. The Pt atom exhibits a positive charge in both cases, while the H^- and Cl^- anions show negative values across all selected methods. We found exceptions in the Mulliken charges calculated with LC-BLYP/def2-TZVP and B3LYP/ZORA-TZVPP/TZVP where the metal showed small values. The CHELPG charges have larger magnitudes than those from Hirshfeld and Mulliken in both molecules, except with TPSSh/LANL2TZ/def2-TZVP for PtCl. We observe two distinct trends for both compounds: the CHELPG and Mulliken charges exhibit similar values for PtCl, while the Hirshfeld and Mulliken charges are comparable for PtH.

A more in-depth analysis of the charge methods is beyond the scope of this study, but our findings offer a comparative insight into these trends.

Table S13: Partial Atomic Charges (e) on all atoms of PtH in the gas phase. The selected levels of theory correspond to the three best predictors of structural parameters and the three for harmonic vibrational frequencies. The level of theory used in the parametrization of the standard MMFF94 force field (HF/LANL2DZ/...) is included. Abbreviated names for the basis sets are used here, with their full names listed in Table 1.

	**3 best predicting structural parameters**			**3 best predicting frequencies**		
	**LC-BLYP/def2-TZVP**			**B3LYP/ZORA-TZVPP/TZVP**		
**Atom**	**CHELPG**	**Hirshfeld**	**Mulliken**	**CHELPG**	**Hirshfeld**	**Mulliken**
**Pt**	0.14342	0.01382	-0.01789	0.11387	0.01312	-0.02752
**H**	-0.14342	-0.01382	0.01789	-0.11387	-0.01312	0.02752
	**TPSSh/def2-QZVP**			**CAM-B3LYP/Sappo-DZP/DZP**		
**Atom**	**CHELPG**	**Hirshfeld**	**Mulliken**	**CHELPG**	**Hirshfeld**	**Mulliken**
**Pt**	0.12814	0.01796	0.01745	0.15193	0.02860	0.06238
**H**	-0.12814	-0.01796	-0.01745	-0.15193	-0.02860	-0.06238
	**CAM-B3LYP/LANL2TZ/def2-TZVP**			**M06L/DKH-TZVP/TZVP**		
**Atom**	**CHELPG**	**Hirshfeld**	**Mulliken**	**CHELPG**	**Hirshfeld**	**Mulliken**
**Pt**	0.14519	0.01538	0.00572	0.11814	0.02172	0.05673
**H**	-0.14519	-0.01538	-0.00572	-0.11814	-0.02172	-0.05673
	**Standard MMFF94 force field**					
	**HF/LANL2DZ/6-31G(d)**					
**Atom**	**CHELPG**	**Hirshfeld**	**Mulliken**			
**Pt**	0.28894	0.07724	0.01248			
**H**	-0.28894	-0.07724	-0.01248			

Table S14: Partial Atomic Charges (e) on all atoms of PtCl in the gas phase. The selected levels of theory correspond to the three best predictors of structural parameters and the three for harmonic vibrational frequencies. The level of theory used in the parametrization of the standard MMFF94 force field (HF/LANL2DZ/...) is included. Abbreviated names for the basis sets are used here, with their full names listed in Table 1.

	**3 best predicting structural parameters**			**3 best predicting frequencies**		
	**mPW2PLYP/ZORA-TZVP/TZVP**			**LC-BLYP/Sappo-DZP/DZP**		
**Atom**	**CHELPG**	**Hirshfeld**	**Mulliken**	**CHELPG**	**Hirshfeld**	**Mulliken**
**Pt**	0.26856	0.09508	0.24720	0.38441	0.22431	0.32665
**Cl**	-0.26856	-0.09508	-0.24720	-0.38441	-0.22431	-0.32665
	**TPSSh/LANL2TZ/def2-TZVP**			**PBE0-DH/ZORA-TZVPP/TZVP**		
**Atom**	**CHELPG**	**Hirshfeld**	**Mulliken**	**CHELPG**	**Hirshfeld**	**Mulliken**
**Pt**	0.25495	0.08205	0.38349	0.29873	0.11554	0.25577
**Cl**	-0.25495	-0.08205	-0.38349	-0.29873	-0.11553	-0.25577
	**B2PLYP/Sappo-QZP/DZP**			**PBE-QIDH/ZORA-TZVP/TZVP**		
**Atom**	**CHELPG**	**Hirshfeld**	**Mulliken**	**CHELPG**	**Hirshfeld**	**Mulliken**
**Pt**	0.26503	0.09127	0.20685	0.30498	0.11933	0.27553
**Cl**	-0.26503	-0.09127	-0.20685	-0.30498	-0.11933	-0.27553
	**Standard MMFF94 force field**					
	**HF/LANL2DZ/6-31G(d)**					
**Atom**	**CHELPG**	**Hirshfeld**	**Mulliken**			
**Pt**	0.54138	0.32340	0.44098			
**Cl**	-0.54138	-0.32340	-0.44098			

Partial atomic charges in the solid phase

Table S15: Partial atomic charges (e) of the β-Cisplatin. Atoms are numbered according to Figure 5.

Atom	Bader	Hirshfeld	Mulliken
Pt1	0.54	0.10	0.20
Pt2	0.54	0.10	0.20
Pt3	0.55	0.10	0.21
Pt4	0.55	0.10	0.21
Cl5	-0.54	-0.18	-0.35
Cl6	-0.54	-0.18	-0.35

to continued ...

Atom	Bader	Hirshfeld	Mulliken
Cl7	-0.55	-0.15	-0.34
Cl8	-0.55	-0.15	-0.34
Cl9	-0.55	-0.14	-0.32
Cl10	-0.54	-0.14	-0.32
Cl11	-0.55	-0.16	-0.36
Cl12	-0.55	-0.16	-0.36
N13	-1.12	-0.15	-0.81
N14	-1.12	-0.15	-0.81
N15	-1.16	-0.15	-0.84
N16	-1.16	-0.15	-0.84
N17	-1.13	-0.15	-0.83
N18	-1.13	-0.15	-0.83
N19	-1.09	-0.15	-0.82
N20	-1.09	-0.15	-0.82
H21	0.46	0.08	0.34
H22	0.46	0.08	0.34
H23	0.47	0.08	0.35
H24	0.47	0.08	0.35
H25	0.47	0.09	0.36
H26	0.47	0.09	0.36
H27	0.50	0.08	0.36
H28	0.50	0.08	0.36
H29	0.45	0.08	0.35
H30	0.45	0.08	0.35

to continued ...

Atom	Bader	Hirshfeld	Mulliken
H31	0.48	0.09	0.36
H32	0.48	0.09	0.36
H33	0.48	0.09	0.35
H34	0.48	0.09	0.35
H35	0.47	0.10	0.37
H36	0.47	0.10	0.37
H37	0.47	0.09	0.37
H38	0.47	0.09	0.37
H39	0.46	0.09	0.35
H40	0.46	0.09	0.35
H41	0.45	0.09	0.36
H42	0.45	0.09	0.36
H43	0.45	0.09	0.35
H44	0.45	0.09	0.35

end.

Bond charge increment (bci)

More details about the methodology of the bci solver

Once we have knowledge of all the bci values and q^0 for the types of atoms present in a given structure, it is possible to obtain the atomic charges for any of its constituent atoms. In contrast, it is natural to ask if it is possible to regenerate within a good degree of accuracy the bci values ω_{I_k,I_j} for a given enumerated structure with specified charges q_j and $q^0_{I_j}$.

As a first insight, we notice that if we have any structure consisting of N atoms, the

equation (6) determines a system of N linear equations and M unknown values, which correspond to the M unique bci values appearing in the structure.

$$q_j - q^0_{I_j} = \sum_{k \text{ bonded to } j} \omega_{I_k,I_j} \tag{1}$$

Thus, our proposed problem is equivalent to finding solutions to the following linear equation

$$\Delta q = B\omega \tag{2}$$

Where Δq and ω are vectors, Δq being the vector in $\mathbb{R}^N$ whose coordinates are specified by the difference of charges $q_j - q^0_{I_j}$ and ω being the vector in $\mathbb{R}^M$ of unknown bci values ω_{I_k,I_j}. We convention that $I_k < I_j$ and entries are sorted by lexicographic order, meaning that a bci ω_{I_j,I_k} has precedence over a bci ω_{I_r,I_s} as an entry of ω if, and only if,

$$I_j < I_r \text{ or } I_j = I_r \text{ and } I_k < I_s.$$

Moreover, B is the following $N \times M$ matrix,

$$B = (b_{rs}),\ 1 \leq r \leq N \text{ and } 1 \leq s \leq M \tag{3}$$

consisting of coefficients for each bci ω_{I_k,I_j} which are obtained from connectivity information about the chemical structure. As such, by equation (2), the matrix B is meant to be so that for each r, the following equation holds:

$$q_r - q^0_{I_r} = \sum_{s=1}^{M} b_{rs}\omega_s \tag{4}$$

Where ω_s denotes the s-th component of the vector $\omega = (\omega_s) \in \mathbb{R}^M$, that is, it is the s-th bci occurring in the chemical structure according to the lexicographic order introduced previously. That is, the entry b_{rs} in the matrix B is an integer value that counts, up to a

sign, the number of atoms neighboring the r-th atom for which the bci of the corresponding interaction coincides with ω_s. The sign appears due to the skew-symmetry of the bci-values and also due to our convention that ω should consist only of those bci-values ω_{I_k,I_j} for which $I_k < I_j$. Explicitly:

$$b_{rs} = \begin{cases} \#\{k : k \text{ is bonded to } r \text{ and } \omega_{I_k,I_r} = \omega_s\}, & \text{if } I_k < I_r \\ -\#\{k : k \text{ is bonded to } r \text{ and } \omega_{I_k,I_r} = -\omega_s\}, & \text{if } I_k > I_r \end{cases} \tag{5}$$

Where # denotes the number of elements in the set. In other words, equation (4) is obtained from (**??**) by counting all repeated occurrences of a particular bci ω_s and recording it as a signed coefficient b_{rs} for this bci, where the sign is specified by whether or not the atom type I_r of the r-th atom is greater than the other atom type defining the bci ω_s.

In further detail, we now describe the procedure taken by the bci solver script to gather the necessary data and organize it in order to solve the equation:

$$\Delta q = B\omega \tag{6}$$

for a given chemical structure. We begin by first noticing that there are 4 pieces of data necessary to implement equation (6), these being:

- Complete information about the connectivity of the chemical structure;
- The charges q_j of each atom;
- The atom types I_j of each atom;
- The integral or fractional formal atomic charges $q^0_{I_j}$ for each atom type I_j in the structure.

About the first item, this information can be neatly implemented by thinking of a chemical structure as a labeled graph whose vertices correspond to a given atom and whose

edges correspond to the existence of a bond between two atoms in the structure. Since labeled graphs are the same thing as boolean matrices, by thinking of a labeled graph as its corresponding adjacency matrix, we are in turn led to implement these labeled graphs in the program through NumPy arrays. Namely, the adjacency matrix for a labeled chemical structure is:

$$a_{ij} = \begin{cases} 1, & \text{if atom } i \text{ is bonded to atom } j \\ 0, & \text{else} \end{cases} \tag{7}$$

For practical reasons, though, what the user actually inputs is not the NumPy array corresponding to the adjacency matrix of the graph representing the chemical structure of interest, but a .mol2 file containing this same information, which is then converted to a NumPy array. This is done since the necessary manipulations to construct the corresponding matrix B representing the linear system of equations are most easily performed via array operations.

Then, the user needs to specify the partial atomic charges for each atom appearing in the chemical structure of interest. These can be readily specified in the .mol2 file provided by the user or available in an external file containing these charges. The format for these external files containing the partial atomic charges is described in the reference.[9]

It's also important to specify the formal atomic charges and atom types of each atom in order to guarantee the well-posedness of the problem. In the current version of the script, however, these can't be directly provided by the user. The formal atomic charge parameters are all set to zero by default, and atoms are not distinguished by the atom types by default.

In any case, once these data are specified by the user, we have all the necessary information to implement and solve equation (6).

The script is organized into four modules:

1. bci_solver_main module, which the user actually interacts with to provide the information we described previously necessary for computations.

2. bci_solver_mol2tools module, responsible for performing conversions between the rele-

vant file formats and data structures as well as generating the output files.

3. bci_solver_optimization, which is responsible for setting up and solving the linear system from the information provided by the user, i.e., constructing the matrix B from the chemical structure and, in turn, using it to find the corresponding bci values.

4. bci_solver_visualization to generate graphs and histograms plotting the computed bci values across different chemical structures.

Matrix representation for cisplatin

For the enumerated cisplatin molecule shown below,

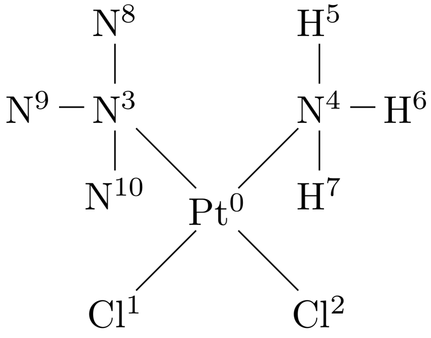

Figure S13: Enumerated cisplatin molecule for matrix calculation.

Its adjacency matrix is:

$$
\begin{bmatrix}
0 & 1 & 1 & 1 & 1 & 0 & 0 & 0 & 0 & 0 & 0 \\
1 & 0 & 0 & 0 & 0 & 0 & 0 & 0 & 0 & 0 & 0 \\
1 & 0 & 0 & 0 & 0 & 0 & 0 & 0 & 0 & 0 & 0 \\
1 & 0 & 0 & 0 & 0 & 0 & 0 & 0 & 1 & 1 & 1 \\
1 & 0 & 0 & 0 & 0 & 1 & 1 & 1 & 0 & 0 & 0 \\
0 & 0 & 0 & 0 & 1 & 0 & 0 & 0 & 0 & 0 & 0 \\
0 & 0 & 0 & 0 & 1 & 0 & 0 & 0 & 0 & 0 & 0 \\
0 & 0 & 0 & 0 & 1 & 0 & 0 & 0 & 0 & 0 & 0 \\
0 & 0 & 0 & 1 & 0 & 0 & 0 & 0 & 0 & 0 & 0 \\
0 & 0 & 0 & 1 & 0 & 0 & 0 & 0 & 0 & 0 & 0 \\
0 & 0 & 0 & 1 & 0 & 0 & 0 & 0 & 0 & 0 & 0
\end{bmatrix}
$$

And its array of atom types is given by:

$$
\begin{bmatrix} 100 & 90 & 90 & 8 & 8 & 23 & 23 & 23 & 23 & 23 & 23 \end{bmatrix}
$$

The unknown bci values in this case are $\omega_{8,23}$, $\omega_{8,100}$ and $\omega_{90,100}$ and the system of equations

obtained for the cisplatin molecule is the following:

$$\begin{bmatrix} q_0 - q_{100}^0 \\ q_1 - q_{90}^0 \\ q_2 - q_{90}^0 \\ q_3 - q_8^0 \\ q_4 - q_8^0 \\ q_5 - q_{23}^0 \\ q_6 - q_{23}^0 \\ q_7 - q_{23}^0 \\ q_8 - q_{23}^0 \\ q_9 - q_{23}^0 \\ q_{10} - q_{23}^0 \end{bmatrix} = \begin{bmatrix} 0 & 2 & 2 \\ 0 & 0 & -1 \\ 0 & 0 & -1 \\ -3 & -1 & 0 \\ -3 & -1 & 0 \\ 1 & 0 & 0 \\ 1 & 0 & 0 \\ 1 & 0 & 0 \\ 1 & 0 & 0 \\ 1 & 0 & 0 \\ 1 & 0 & 0 \end{bmatrix} \begin{bmatrix} \omega_{8,23} \\ \omega_{8,100} \\ \omega_{90,100} \end{bmatrix}$$

Calculated bci for Platinum derivatives of this study: PtH, PtCl, $[PtCl_4]^{2-}$, $[Pt(NH_3)_4]^{2+}$, and cisplatin $[Pt(NH_3)_2Cl_2]$

Table S16: Bond charge increment (bci, e) calculated for the Pt-H, Pt-Cl, Pt-N, and N-H bonds in Platinum derivatives in the gas phase. The bci values are categorized by the charge method used to derive them (CHELPG, Hirshfeld, and Mulliken). Additionally, we present the bci values when grouping the square-planar Pt(II) complexes of this study: $[PtCl_4]^{2-}$, $[Pt(NH_3)_4]^{2+}$, and cisplatin $[Pt(NH_3)_2Cl_2]$). The selected levels of theory correspond to the three best predictors of structural parameters and the three for harmonic vibrational frequencies, as can be seen in Table 2 of the main text. The level of theory used in the standard parametrization of the MMFF94 force field (HF/LANL2DZ/...) is included. Abbreviated names for the basis sets are used here, with their full names listed in Table 1.

compound	level of theory	bci	**CHELPG**	**Hirshfeld**	**Mulliken**
PtH	**LC-BLYP/def2-TZVP**	Pt-H	-0.143	-0.014	0.018
	**TPSSh/def2-QZVP**	Pt-H	-0.128	-0.018	-0.017
	**CAM-B3LYP/LANL2TZ/def2-TZVP**	Pt-H	-0.145	-0.015	-0.006
	**B3LYP/ZORA-TZVPP/TZVP**	Pt-H	-0.114	-0.013	0.028
	**CAM-B3LYP/Sappo-DZP/DZP**	Pt-H	-0.152	-0.029	-0.062
	**M06L/DKH-TZVP/TZVP**	Pt-H	-0.118	-0.022	-0.057
	**arithmetic average**		**-0.133**	**-0.018**	**-0.016**
	**standard deviation**		**0.016**	**0.006**	**0.037**
	**HF/LANL2DZ/6-31G(d)**	Pt-H	-0.289	-0.077	-0.013
PtCl	**mPW2PLYP/ZORA-TZVP/TZVP**	Pt-Cl	-0.269	-0.095	-0.247
	**TPSSh/LANL2TZ/def2-TZVP**	Pt-Cl	-0.255	-0.082	-0.384
	**B2PLYP/Sappo-QZP/DZP**	Pt-Cl	-0.265	-0.091	-0.207

...

compound	level of theory	bci	**CHELPG**	**Hirshfeld**	**Mulliken**
	**LC-BLYP/Sappo-DZP/DZP**	Pt-Cl	-0.384	-0.224	-0.327
	**PBE0-DH/ZORA-TZVPP/TZVP**	Pt-Cl	-0.297	-0.116	-0.256
	**PBE-QIDH/ZORA-TZVP/TZVP**	Pt-Cl	-0.305	-0.119	-0.276
	**arithmetic average**		**-0.296**	**-0.121**	**-0.283**
	**standard deviation**		**0.048**	**0.052**	**0.063**
	**HF/LANL2DZ/6-31G(d)**	Pt-Cl	-0.541	-0.323	-0.441
	MAD (e) compared with ref.[10]	Pt-Cl	0.067	0.242	0.081
$[PtCl_4]^{2-}$	**PBE-QIDH/DKH-TZVPP/TZVP**	Pt-Cl	-0.175	-0.058	-0.170
	**DSD-BLYP/DKH-TZVPP/TZVP**	Pt-Cl	-0.168	-0.053	-0.165
	**PBE0-DH/DKH-TZVPP/TZVP**	Pt-Cl	-0.177	-0.060	-0.171
	**PBE-QIDH/def2-QZVP**	Pt-Cl	-0.210	-0.061	-0.313
	**DSD-BLYP/def2-QZVP**	Pt-Cl	-0.208	-0.057	-0.304
	**LC-BLYP/Sappo-DZP/DZP**	Pt-Cl	-0.179	-0.070	-0.168
	**arithmetic average**		**-0.186**	**-0.060**	**-0.215**
	**standard deviation**		**0.018**	**0.006**	**0.072**
	**HF/LANL2DZ/6-31G(d)**	Pt-Cl	-0.246	-0.121	-0.165
	MAD (e) compared with ref.[10]	Pt-Cl	0.177	0.303	0.148
$[Pt(NH_3)_4]^{2+}$	**PBE-QIDH/Sappo-TZP/DZP**	Pt-N	0.003	-0.007	-0.022

...

compound	level of theory	bci	**CHELPG**	**Hirshfeld**	**Mulliken**
	**PBE0-DH/Sappo-QZP/DZP**	Pt-N	0.002	-0.009	0.011
	**LC-BLYP/Sappo-QZP/ DZP**	Pt-N	0.020	-0.010	0.037
	**LC-BLYP/Sappo-DZP/DZP**	Pt-N	0.024	-0.012	-0.027
	**TPSSh/Sappo-DZP/DZP**	Pt-N	0.028	-0.011	-0.030
	**B3LYP/Sappo-DZP/DZP**	Pt-N	0.026	-0.014	-0.038
	**arithmetic average**		**0.017**	**-0.011**	**-0.011**
	**standard deviation**		**0.012**	**0.002**	**0.029**
	**HF/LANL2DZ/6-31G(d)**	Pt-N	-0.063	-0.050	-0.244
	MAD (e) compared with ref.[10]	Pt-N	0.395	0.402	0.401
$[Pt(NH_3)_4]^{2+}$	**PBE-QIDH/Sappo-TZP/DZP**	N-H	0.213	0.059	0.068
	**PBE0-DH/Sappo-QZP/DZP**	N-H	0.214	0.059	0.063
	**LC-BLYP/Sappo-QZP/ DZP**	N-H	0.201	0.061	0.052
	**LC-BLYP/Sappo-DZP/DZP**	N-H	0.199	0.060	0.059
	**TPSSh/Sappo-DZP/DZP**	N-H	0.194	0.057	0.059
	**B3LYP/Sappo-DZP/DZP**	N-H	0.189	0.058	0.048
	**arithmetic average**		**0.202**	**0.059**	**0.058**
	**standard deviation**		**0.010**	**0.001**	**0.007**

...

compound	level of theory	bci	**CHELPG**	**Hirshfeld**	**Mulliken**
	**HF/LANL2DZ/6-31G(d)**	N-H	0.223	0.051	0.353
cisplatin	**DSD-BLYP/Sappo-TZP/DZP**	Pt-Cl	-0.336	-0.288	-0.400
	**B2PLYP/Sappo-QZP/DZP**	Pt-Cl	-0.331	-0.290	-0.386
	**PBE-QIDH/Sappo-DZP/DZP**	Pt-Cl	-0.335	-0.304	-0.418
	**TPSSh/DKH-TZVP/TZVP**	Pt-Cl	-0.317	-0.285	-0.414
	**LC-BLYP/Sappo-QZP/DZP**	Pt-Cl	-0.3405	-0.300	-0.382
	**B97-3c/DKH-TZVP/TZVP**	Pt-Cl	-0.308	-0.276	-0.396
	**arithmetic average**		**-0.328**	**-0.290**	**-0.399**
	**standard deviation**		**0.013**	**0.010**	**0.014**
	**HF/LANL2DZ/6-31G(d)**	Pt-Cl	-0.460	-0.379	-0.390
	MAD (e) compared with ref.[10]	Pt-Cl	0.035	0.073	0.036
cisplatin	**DSD-BLYP/Sappo-TZP/DZP**	Pt-N	0.349	0.312	0.343
	**B2PLYP/Sappo-QZP/DZP**	Pt-N	0.346	0.307	0.363
	**PBE-QIDH/Sappo-DZP/DZP**	Pt-N	0.351	0.311	0.302
	**TPSSh/DKH-TZVP/TZVP**	Pt-N	0.327	0.294	0.248
	**LC-BLYP/Sappo-QZP/DZP**	Pt-N	0.340	0.304	0.383
	**B97-3c/DKH-TZVP/TZVP**	Pt-N	0.333	0.300	0.244
	**arithmetic average**		**0.341**	**0.305**	**0.313**

...

compound	level of theory	bci	**CHELPG**	**Hirshfeld**	**Mulliken**
	**standard deviation**		**0.010**	**0.007**	**0.059**
	**HF/LANL2DZ/6-31G(d)**	Pt-N	0.321	0.306	0.157
	MAD (e) compared with ref.[10]	Pt-N	0.071	0.108	0.099
cisplatin	**DSD-BLYP/Sappo-TZP/DZP**	N-H	0.249	0.136	0.135
	**B2PLYP/Sappo-QZP/DZP**	N-H	0.244	0.135	0.123
	**PBE-QIDH/Sappo-DZP/DZP**	N-H	0.252	0.138	0.150
	**TPSSh/DKH-TZVP/TZVP**	N-H	0.251	0.131	0.263
	**LC-BLYP/Sappo-QZP/DZP**	N-H	0.253	0.137	0.123
	**B97-3c/DKH-TZVP/TZVP**	N-H	0.246	0.132	0.255
	**arithmetic average**		**0.249**	**0.135**	**0.175**
	**standard deviation**		**0.004**	**0.003**	**0.066**
	**HF/LANL2DZ/6-31G(d)**	N-H	0.283	0.138	0.435
Compilated	**For square-planar Pt(II)**	**Pt-Cl arith. av.**	-0.257	-0.175	-0.307
bci		**Pt-Cl st. dev.**	0.075	0.121	0.108
values	**For square-planar Pt(II)**	**Pt-N arith. av.**	0.179	0.147	0.151
		**Pt-N st. dev.**	0.169	0.165	0.175
	**For square-planar Pt(II)**	**N-H arith. av.**	0.226	0.097	0.116

...

compound	level of theory	bci	**CHELPG**	**Hirshfeld**	**Mulliken**
		**N-H st. dev.**	0.026	0.040	0.076

end.

Table S17: Bond charge increment (bci, e) calculated for the Pt-Cl, Pt-N, and N-H bonds in the two polymorphs of cisplatin in the solid phase. The bci are categorized by the charge methods used to derive them (Bader, Hirshfeld, and Mulliken). Additionally, we present the bci values when grouping the two polymorphs of cisplatin.

polymorph	Level of theory	bci	**Bader**	**Hirshfeld**	**Mulliken**
α-cisplatin	PBE-D3/PAW	Pt-Cl	-0.568	-0.145	-0.319
		Pt-N	0.052	0.045	0.108
		N-H	0.428	0.065	0.306
β-cisplatin	PBE-D3/PAW	Pt-Cl	-0.407	-0.094	-0.209
		Pt-N	-0.056	0.011	0.030
		N-H	0.395	0.060	0.297
Compilated	**arithmetic average**	Pt-Cl	-0.487	-0.119	-0.264
bci values	**standard deviation**		0.114	0.036	0.078
for both	**arithmetic average**	Pt-N	-0.002	0.028	0.069
polymorphs	**standard deviation**		0.076	0.024	0.055
	**arithmetic average**	N-H	0.411	0.063	0.301
	**standard deviation**		0.023	0.003	0.006

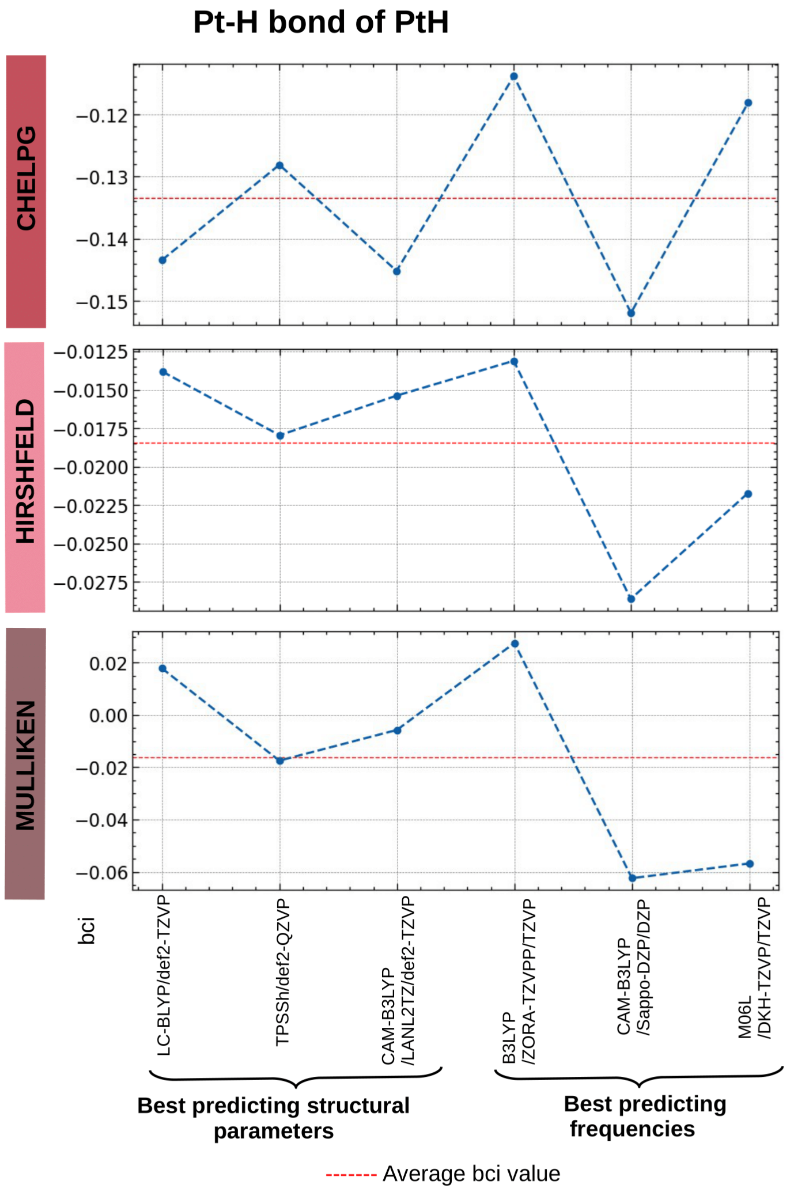

Figure S14: Computed bci (e) values for the Pt-H bond of PtH in the gas phase. The bci are categorized by the charge methods used to derive them (CHELPG, Hirshfeld, and Mulliken). The selected levels of theory correspond to the three best predictors of structural parameters and the three for harmonic vibrational frequencies. Abbreviated names for the basis sets are used here, with their full names listed in Table 1.

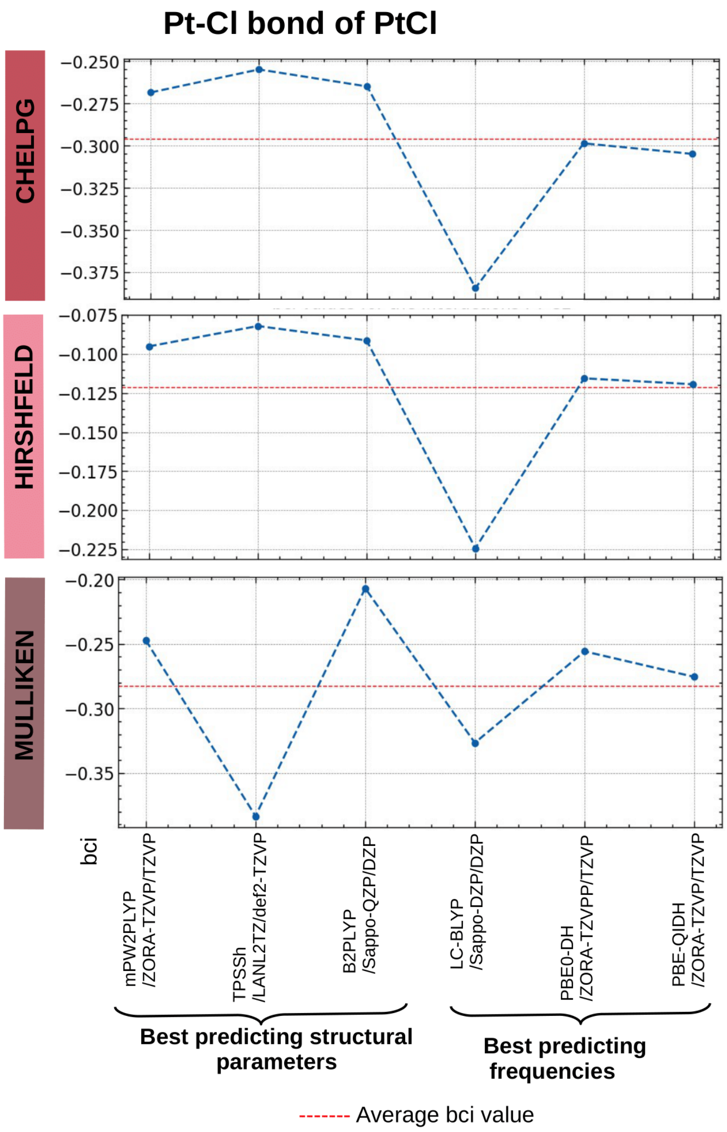

Figure S15: Computed bci (e) values for the Pt-Cl bond of PtCl in the gas phase. The bci are categorized by the charge methods used to derive them (CHELPG, Hirshfeld, and Mulliken). The selected levels of theory correspond to the three best predictors of structural parameters and the three for harmonic vibrational frequencies. Abbreviated names for the basis sets are used here, with their full names listed in Table 1.

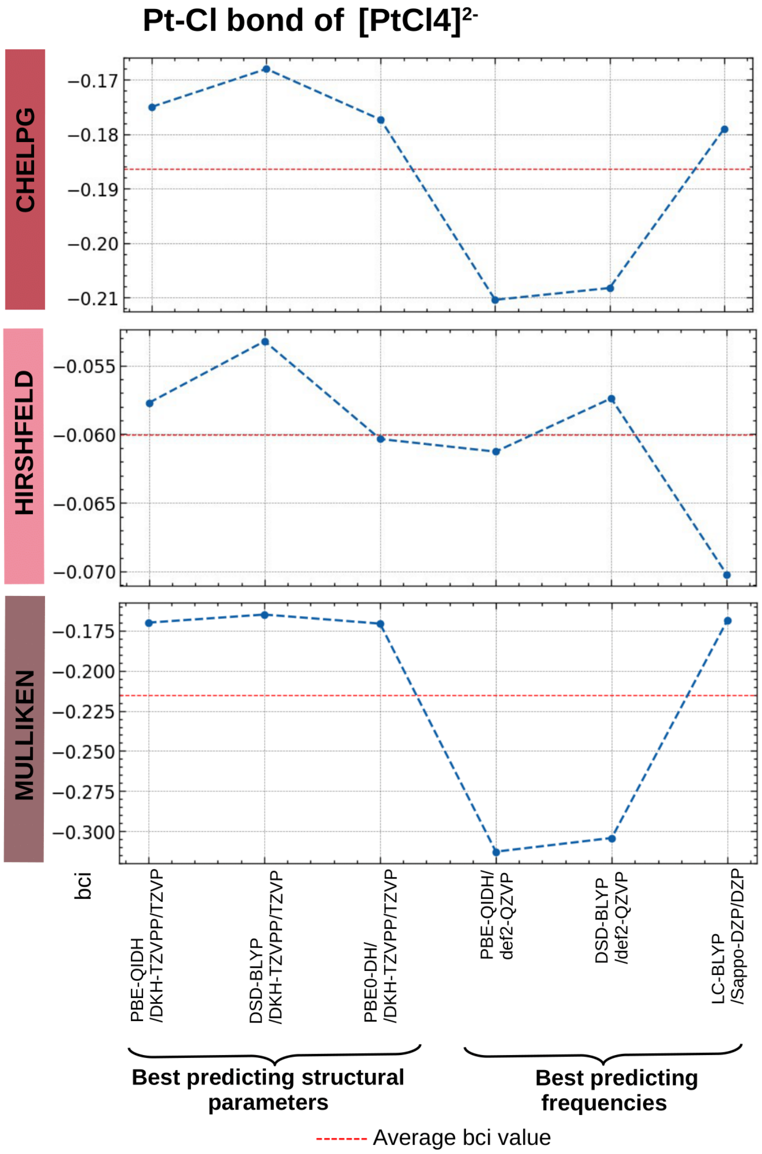

Figure S16: Computed bci (e) values for the Pt-Cl bond of $[PtCl_4]^{2-}$ in the gas phase. The bci are categorized by the charge methods used to derive them (CHELPG, Hirshfeld, and Mulliken). The selected levels of theory correspond to the three best predictors of structural parameters and the three for harmonic vibrational frequencies. Abbreviated names for the basis sets are used here, with their full names listed in Table 1.

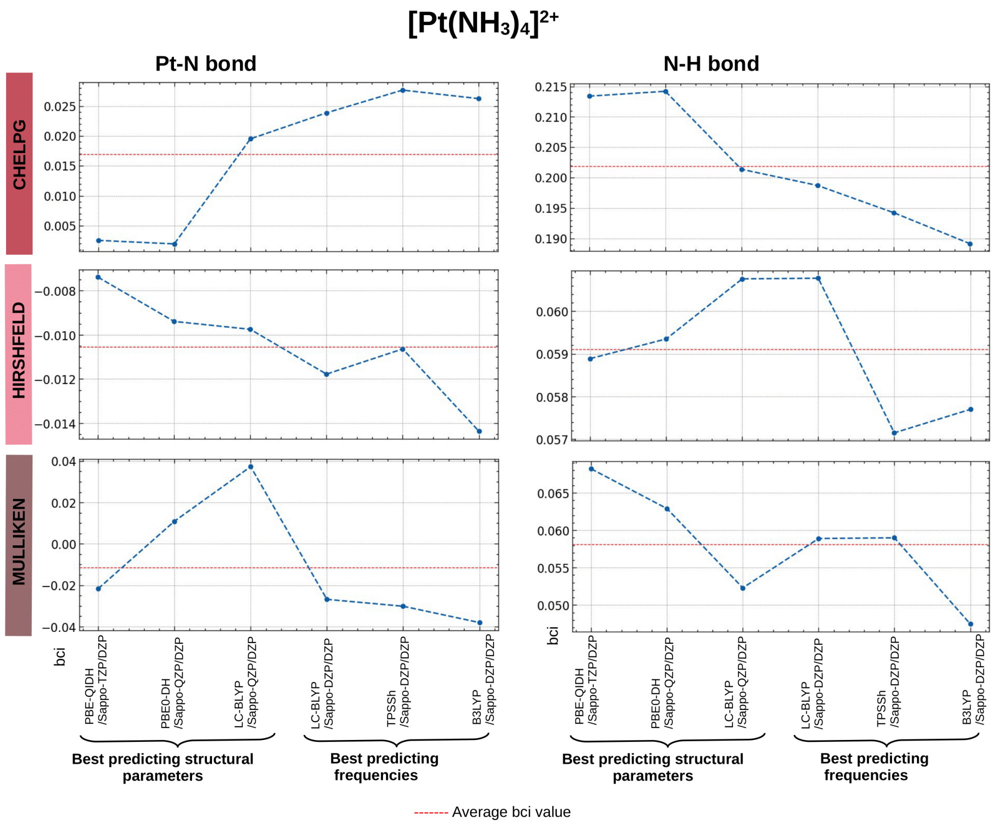

Figure S17: Computed bci (e) values for the Pt-N, and N-H bonds of $[Pt(NH_3)_4]^{2+}$ in the gas phase. The bci are categorized by the charge methods used to derive them (CHELPG, Hirshfeld, and Mulliken). The selected levels of theory correspond to the three best predictors of structural parameters and the three for harmonic vibrational frequencies. Abbreviated names for the basis sets are used here, with their full names listed in Table 1.

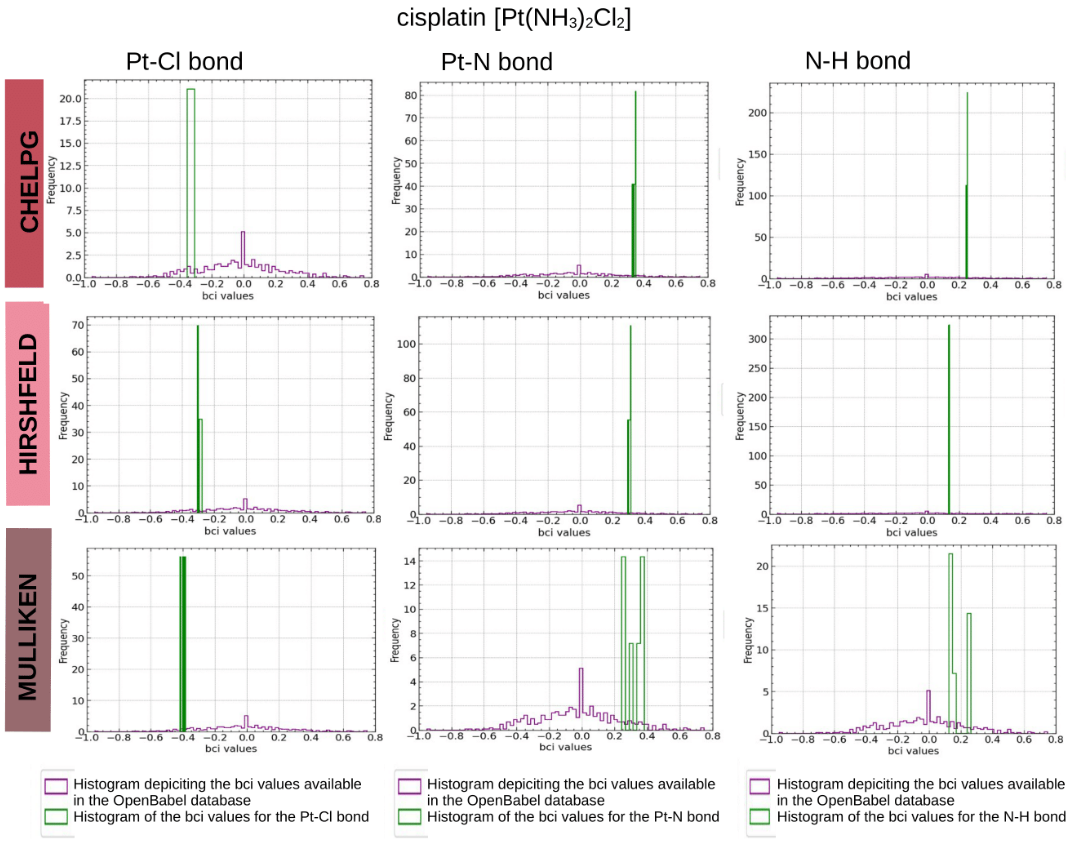

Figure S18: Computed bci histograms for the Pt-Cl, Pt-N, and N-H bonds of cisplatin molecule in the gas phase across different basis sets. The bci (e) are categorized by the charge methods used to derive them (CHELPG, Hirshfeld, and Mulliken).

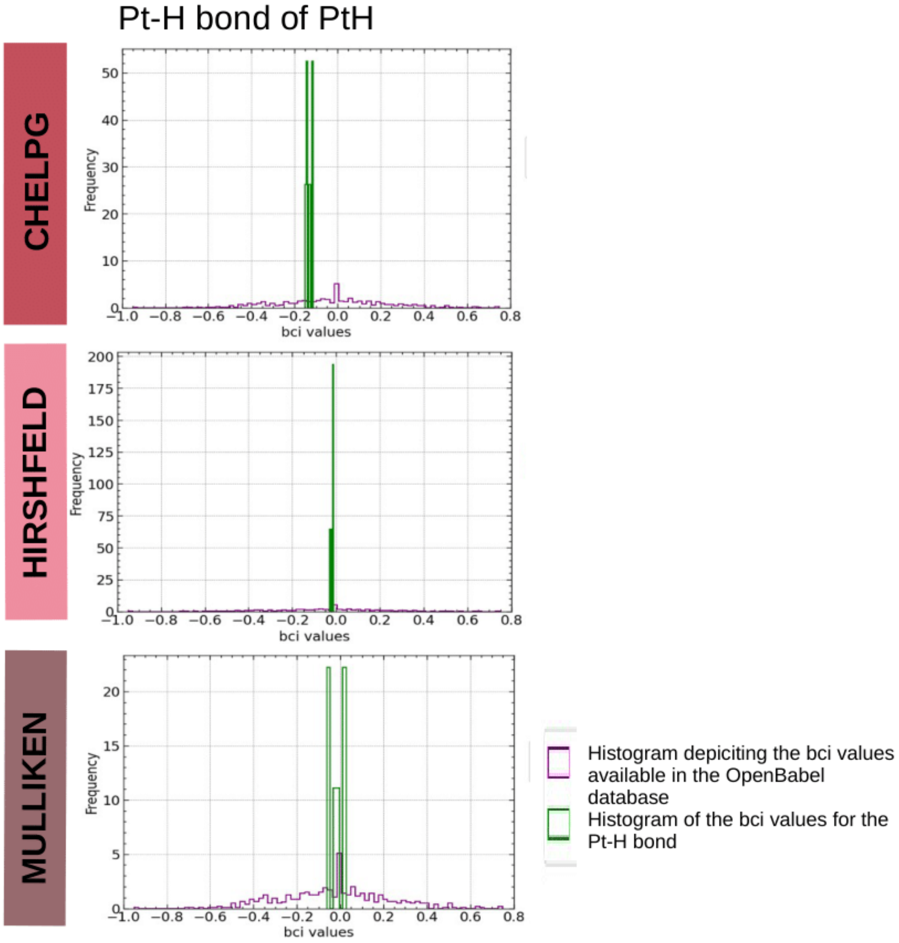

Figure S19: Computed bci histograms for the Pt-H bond of PtH in the gas phase across different basis sets. The bci (e) are categorized by the charge methods used to derive them (CHELPG, Hirshfeld, and Mulliken).

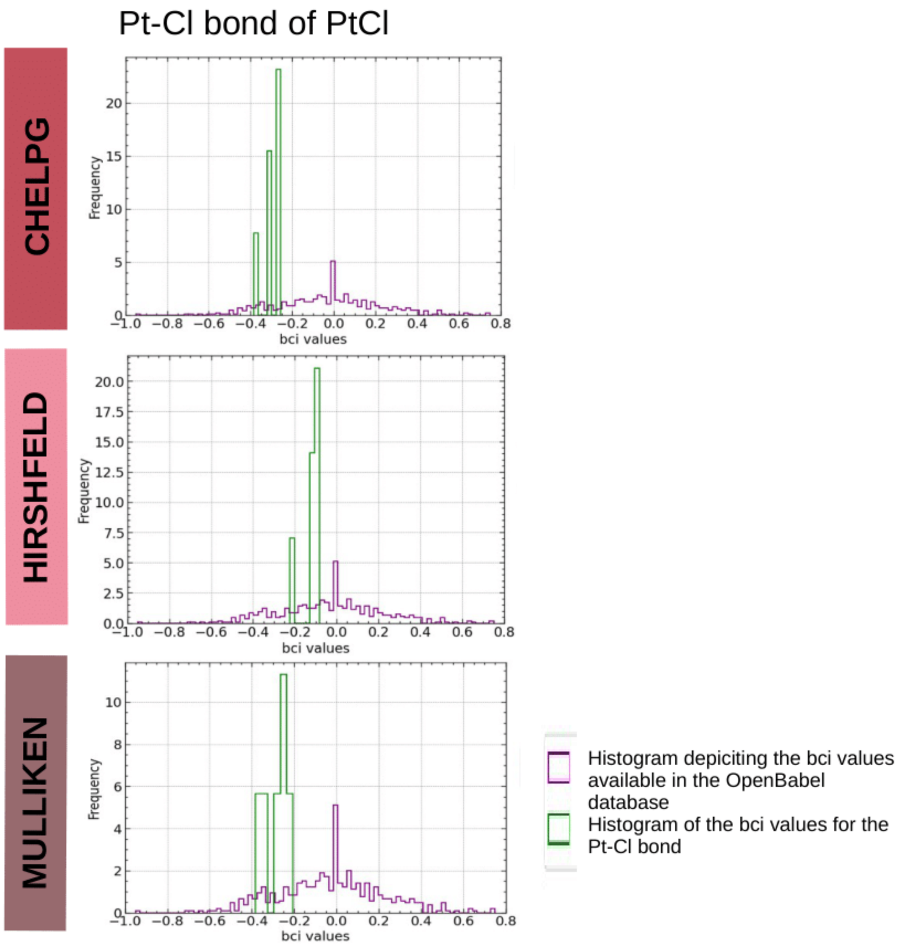

Figure S20: Computed bci histograms for the Pt-Cl bond of PtCl in the gas phase across different basis sets. The bci (e) are categorized by the charge methods used to derive them (CHELPG, Hirshfeld, and Mulliken).

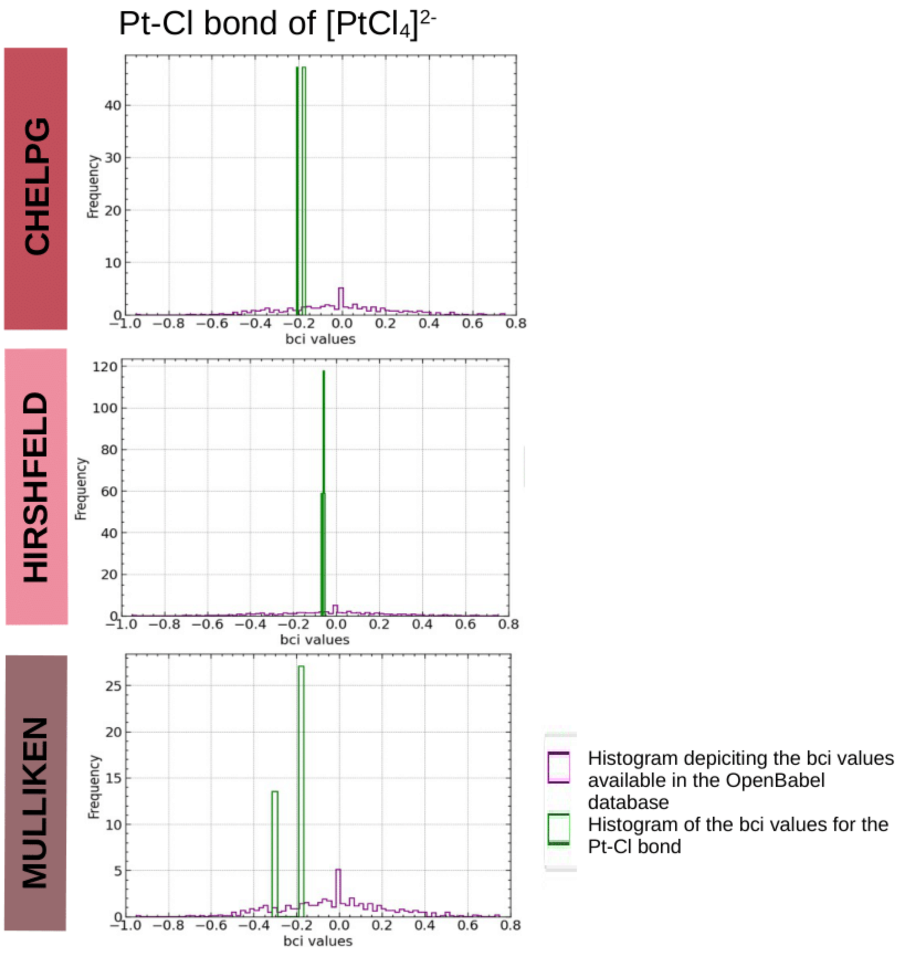

Figure S21: Computed bci histograms for the Pt-Cl bond of $[PtCl_4]^{2-}$ in the gas phase across different basis sets. The bci (e) are categorized by the charge methods used to derive them (CHELPG, Hirshfeld, and Mulliken).

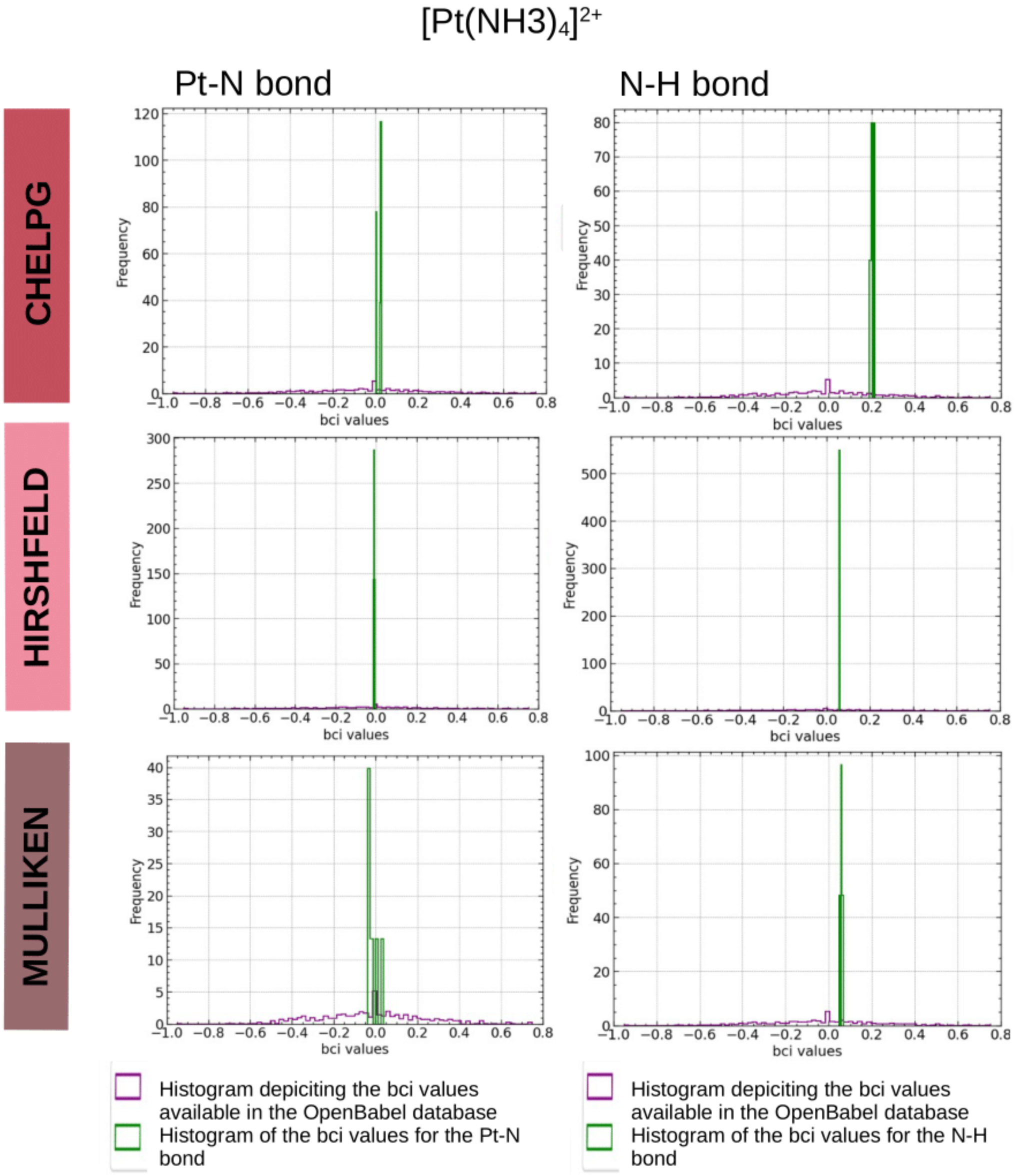

Figure S22: Computed bci histograms for the Pt-N, and N-H bonds of $[Pt(NH_3)_4]^{2+}$ in the gas phase across different basis sets. The bci (e) are categorized by the charge methods used to derive them (CHELPG, Hirshfeld, and Mulliken).

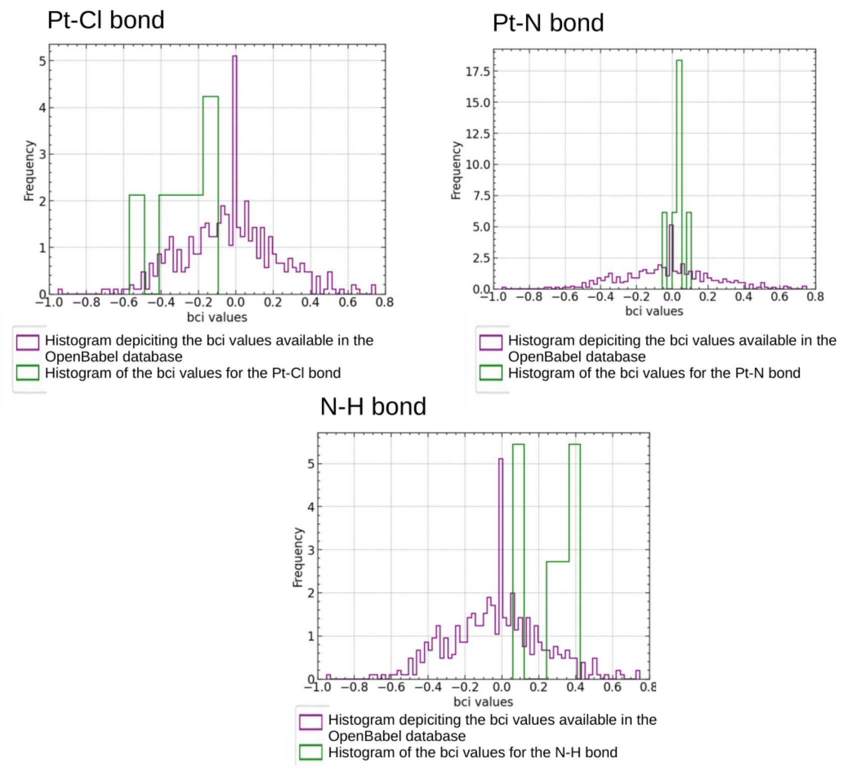

Figure S23: Computed bci histograms for the Pt-Cl, Pt-N, and N-H bonds of the α-cisplatin and β-cisplatin in the solid phase.

Additional examples for calculated bci

Methane

We present below a labeled methane molecule, where we convention that atoms are enumerated starting from 0.

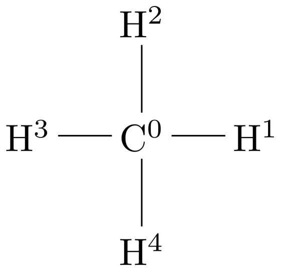

Figure S24: Enumerated methane molecule

Therefore, the adjacency matrix for the methane molecule labeled as above is precisely:

$$\begin{bmatrix} 0 & 1 & 1 & 1 & 1 \\ 1 & 0 & 0 & 0 & 0 \\ 1 & 0 & 0 & 0 & 0 \\ 1 & 0 & 0 & 0 & 0 \\ 1 & 0 & 0 & 0 & 0 \end{bmatrix}$$

Which is implemented as a numpy array.

The central carbon atom for this molecule has its MMFF94 atom type equal to 1 according to[11] and all of its neighbouring hydrogen atoms have atom type equaol to 5, we specify this by the following array:

$$\begin{bmatrix} 1 & 5 & 5 & 5 & 5 \end{bmatrix}$$

Which is constructed so that the entry corresponding to the 0th column has the same atom type as the 0th atom in the label, in this case being equal to 1, the 1st column having an entry value corresponding to the atom type of the 1st labeled atom, in this case being equal to 5, with this pattern repeating for the other entries.

The system of equations which relates the bci values and the atom charges for this molecule - for a given set of atom charges $\{q_0, q_1, q_2, q_3, q_4\}$ and formal charges $\{q_1^0, q_5^0\}$ (associated to atoms of MMFF94 atom type 1 and 5 respectively) - is given by:

$$\begin{cases} q_0 - q_1^0 = -4\,\omega_{1,5} \\ q_1 - q_5^0 = \omega_{1,5} \\ q_2 - q_5^0 = \omega_{1,5} \\ q_3 - q_5^0 = \omega_{1,5} \\ q_4 - q_5^0 = \omega_{1,5} \end{cases}$$

Which can be rewritten in matrix notation as:

$$\begin{bmatrix} q_0 - q_1^0 \\ q_1 - q_5^0 \\ q_2 - q_5^0 \\ q_3 - q_5^0 \\ q_4 - q_5^0 \end{bmatrix} = \begin{bmatrix} -4 \\ 1 \\ 1 \\ 1 \\ 1 \end{bmatrix} \cdot \omega_{1,5}$$

Ethane

For the ethane molecule:

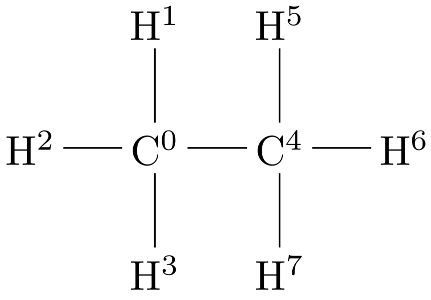

Figure S25: Enumerated ethane molecule

we have the following adjacency matrix:

$$\begin{bmatrix} 0 & 1 & 1 & 1 & 1 & 0 & 0 & 0 \\ 1 & 0 & 0 & 0 & 0 & 0 & 0 & 0 \\ 1 & 0 & 0 & 0 & 0 & 0 & 0 & 0 \\ 1 & 0 & 0 & 0 & 0 & 0 & 0 & 0 \\ 1 & 0 & 0 & 0 & 0 & 1 & 1 & 1 \\ 0 & 0 & 0 & 0 & 1 & 0 & 0 & 0 \\ 0 & 0 & 0 & 0 & 1 & 0 & 0 & 0 \\ 0 & 0 & 0 & 0 & 1 & 0 & 0 & 0 \end{bmatrix}$$

The following array of atom types:

$$\begin{bmatrix} 1 & 5 & 5 & 5 & 1 & 5 & 5 & 5 \end{bmatrix}$$

And associated system of linear equations given by:

$$\begin{bmatrix} q_0 - q_1^0 \\ q_1 - q_5^0 \\ q_2 - q_5^0 \\ q_3 - q_5^0 \\ q_4 - q_1^0 \\ q_5 - q_5^0 \\ q_6 - q_5^0 \\ q_7 - q_5^0 \end{bmatrix} = \begin{bmatrix} -3 \\ 1 \\ 1 \\ 1 \\ -3 \\ 1 \\ 1 \\ 1 \end{bmatrix} \cdot \omega_{1,5}$$

***tert*-Butyl alcohol**

For the *tert*-Butyl alcohol molecule:

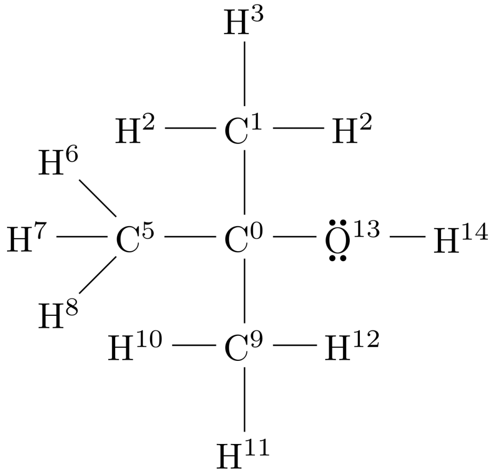

Figure S26: Enumerated *ter*-butyl-alcohol molecule

Its adjacency matrix is given by: .

$$\begin{bmatrix}
0&1&0&0&0&1&0&0&0&1&0&0&0&1&0\\
1&0&1&1&1&0&0&0&0&0&0&0&0&0&0\\
0&1&0&0&0&0&0&0&0&0&0&0&0&0&0\\
0&1&0&0&0&0&0&0&0&0&0&0&0&0&0\\
0&1&0&0&0&0&0&0&0&0&0&0&0&0&0\\
1&0&0&0&0&0&1&1&1&0&0&0&0&0&0\\
0&0&0&0&0&1&0&0&0&0&0&0&0&0&0\\
0&0&0&0&0&1&0&0&0&0&0&0&0&0&0\\
0&0&0&0&0&1&0&0&0&0&0&0&0&0&0\\
1&0&0&0&0&0&0&0&0&0&1&1&1&0&0\\
0&0&0&0&0&0&0&0&0&1&0&0&0&0&0\\
0&0&0&0&0&0&0&0&0&1&0&0&0&0&0\\
0&0&0&0&0&0&0&0&0&1&0&0&0&0&0\\
1&0&0&0&0&0&0&0&0&0&0&0&0&0&1\\
0&0&0&0&0&0&0&0&0&0&0&0&0&1&0
\end{bmatrix}$$

Its array of atom types is given by:

$$\begin{bmatrix}29 & 1 & 5 & 5 & 5 & 1 & 5 & 5 & 5 & 1 & 5 & 5 & 5 & 6 & 21\end{bmatrix}$$

The unknown bci values are $\omega_{1,5}, \omega_{1,29}, \omega_{6,21}$ and $\omega_{6,29}$. The associated system of linear

equations is:

$$\begin{bmatrix} q_0 - q_{29}^0 \\ q_1 - q_1^0 \\ q_2 - q_5^0 \\ q_3 - q_5^0 \\ q_4 - q_5^0 \\ q_5 - q_1^0 \\ q_6 - q_5^0 \\ q_7 - q_5^0 \\ q_8 - q_5^0 \\ q_9 - q_1^0 \\ q_{10} - q_5^0 \\ q_{11} - q_5^0 \\ q_{12} - q_5^0 \\ q_{13} - q_6^0 \\ q_{14} - q_{21}^0 \end{bmatrix} = \begin{bmatrix} 0 & 3 & 0 & 1 \\ -3 & -1 & 0 & 0 \\ 1 & 0 & 0 & 0 \\ 1 & 0 & 0 & 0 \\ 1 & 0 & 0 & 0 \\ -3 & -1 & 0 & 0 \\ 1 & 0 & 0 & 0 \\ 1 & 0 & 0 & 0 \\ 1 & 0 & 0 & 0 \\ -3 & -1 & 0 & 0 \\ 1 & 0 & 0 & 0 \\ 1 & 0 & 0 & 0 \\ 1 & 0 & 0 & 0 \\ 0 & 0 & -1 & -1 \\ 0 & 0 & 1 & 0 \end{bmatrix} \cdot \begin{bmatrix} \omega_{1,5} \\ \omega_{1,29} \\ \omega_{6,21} \\ \omega_{6,29} \end{bmatrix}$$

Electronic Properties of Platinum derivatives in the gas phase

Dipole moments and molecular polarizabilities are summarized in **Figure S27** and **Table S18**. In our research, no experimental values were found; consequently, only theoretical values were reported and compared with references from the literature. In general, the standard MMFF94 force field (HF/LANL2DZ/6-31G(d)) overestimates the dipole moments of cisplatin and the diatomic compounds (PtCl, and PtH) compared to the density functional methods used in this study. However, the ab initio method underestimated the molecular polarizability of square-planar Pt(II) molecules ($[PtCl_4]^{2-}$, $[Pt(NH_3)_4]^{2+}$, and cisplatin).

Figure S27 shows a direct relationship between molecular polarizability and molecular

sized, while no such trend is observed for dipole moment. The descending order of polarizability is: $[PtCl_4]^{2-}$ (78.7956-102.2066 $a.u^3$) > cisplatin (64.4803-85.300 $a.u^3$) > $[Pt(NH_3)_4]^{2+}$ (53.7194-58.4521 $a.u^3$) > PtCl (29.0499-68.3524 $a.u^3$) > PtH (22.2064-41.0316 $a.u^3$).

For dipole moments, the descending order is: cisplatin (10.1216-11.3988 D) > PtCl (2.6240-4.0868 D) > PtH (1.1188-1.4423 D) > $[Pt(NH_3)_4]^{2+}$ ≈ $[PtCl_4]^{2-}$ (≈ 0 D).

Our dipole moment values for cisplatin which align with literature reported values using different theoretical methods, such as 10.5840 D (CAM-B3LYP/DKH2/DZP-DKH),[12] 11.866 D (PBE0-D3BJ/def2-TZVP/aug-cc-pVDZ),[13] and 12.640 D (HF/6-31**).[14]

Additionally, our results for the molecular polarizability of cisplatin are lower than other theoretical values, such as 88.54 $a.u^3$ (CAM-B3LYP/DKH2/DZP-DKH),[12] and 58.02 $a.u^3$ (HF/6-31**).[14]

As shown in **Table S18** the dipole moment of PtCl is more than double that of PtH, attributed to the higher electronegativity of the chlorine atom compared to hydrogen atom.[15] Theoretical literature reports dipole moment of 1.194 D for PtH and 3.042 D for PtCl.[15] Our calculations slightly overestimated and underestimated these values, depending on the computational approach used.

The molecular polarizability of PtH calculated with the range-separated hybrid functional CAM-B3LYP is 41.0316 $a.u^3$ using the RECP basis set (LANL2TZ/def2-TZVP). This result is consistent with the values of 40.97 $a.u^3$ reported by De Berredo and colleagues,[12] obtained with the scalar relativistic all-electron basis set (DKH2/DZP-DKH) using the same functional.

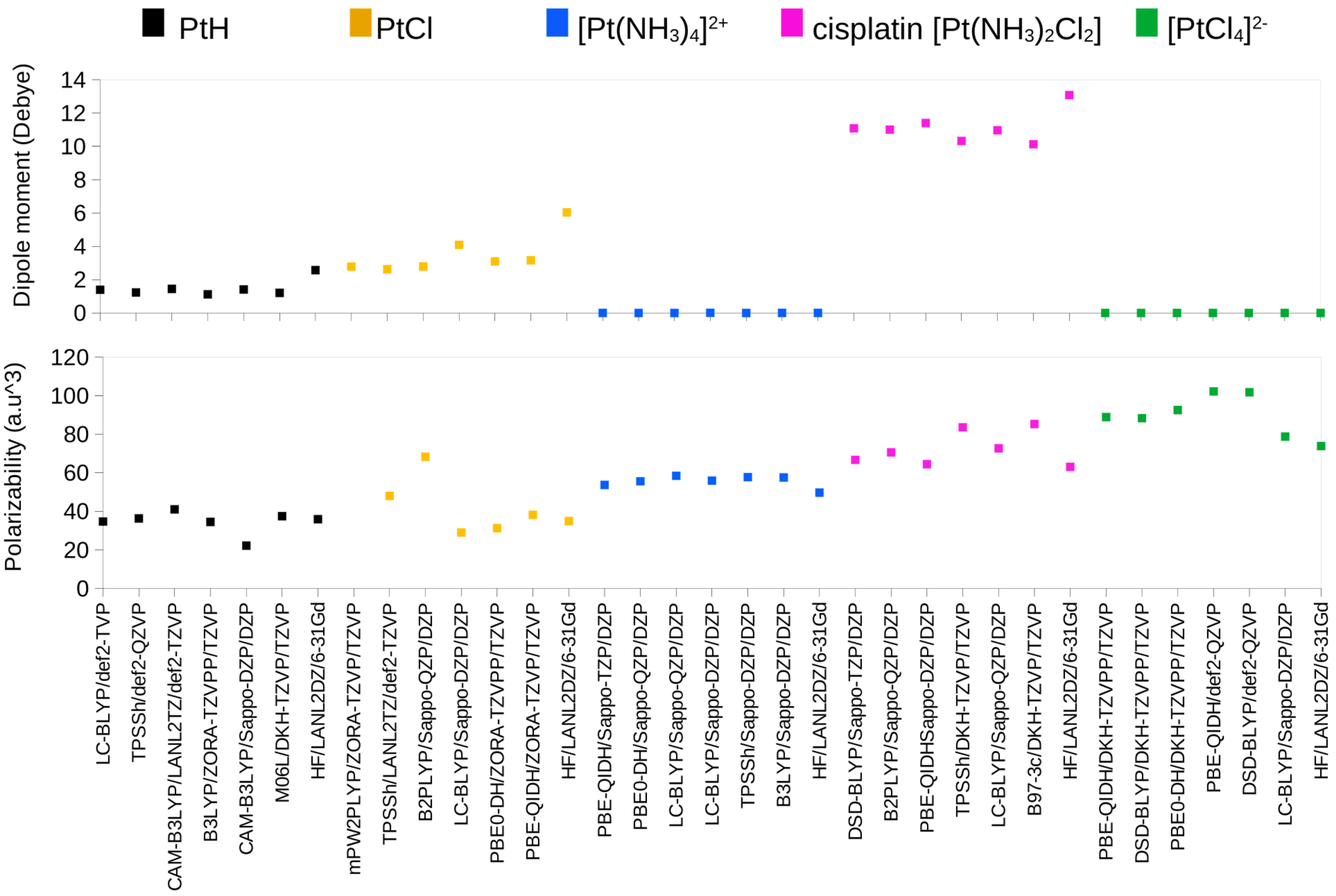

Figure S27: Calculated electronic properties: dipole moment (Debye), and molecular polarizability ($a.u^3$) of PtH, PtCl, $[PtCl_4]^{2-}$, $[Pt(NH_3)_4]^{2+}$, and cisplatin $[Pt(NH_3)_2Cl_2]$, in the gas phase. The selected levels of theory correspond to the three best predictors of structural parameters and the three for harmonic vibrational frequencies. The level of theory used in the standard MMFF94 force field (HF/LANL2DZ/...) is included. Abbreviated names for the basis sets are used here, with their full names listed in Table 1.

Table S18: Calculated dipole moment (D) and molecular polarizability ($a.u^3$) of PtH, PtCl, $[PtCl_4]^{2-}$, $[Pt(NH_3)_4]^{2+}$ and *cis*-$[Pt(NH_3)_2Cl_2]$ in the gas phase. The selected levels of theory correspond to the three best predictors of structural parameters and the three for harmonic vibrational frequencies. The level of theory used in the standard MMFF94 force field (HF/LANL2DZ/...) is included. Abbreviated names for the basis sets are used here, with their full names listed in Table 1. n.a: no applied.

Compound	Level of theory	Dipole moment	Polarizab.
**PtH**	**Best predicting for structural parameters**		
	LC-BLYP/def2-TVP	1.39053	34.7172
	TPSSh/def2-QZVP	1.22820	36.3125
	CAM-B3LYP/LANL2TZ/def2-TZVP	1.44230	41.0316
	**Best predicting for frequencies**		
	B3LYP/ZORA-TZVPP/TZVP	1.11883	34.5266
	CAM-B3LYP/Sappo-DZP/DZP	1.41016	22.2064
	M06L/DKH-TZVP/TZVP	1.20869	37.5372
	HF/LANL2DZ/6-31G(d)	2.57129	35.9160
**PtCl**	**Best predicting for structural parameters**		
	mPW2PLYP/ZORA-TZVP/TZVP	2.78399	n.a
	TPSSh/LANL2TZ/def2-TZVP	2.62400	48.0177
	B2PLYP/Sappo-QZP/DZP	2.78972	68.3524
	**Best predicting for frequencies**		
	LC-BLYP/Sappo-DZP/DZP	4.08680	29.0499
	PBE0-DH/ZORA-TZVPP/TZVP	3.09009	31.2653
	PBE-QIDH/ZORA-TZVP/TZVP	3.15938	38.2059
	HF/LANL2DZ/6-31G(d)	6.03683	34.9622
$\mathbf{[Pt(NH_3)_4]^{2+}}$	**Best predicting for structural parameters**		
	PBE-QIDH/Sappo-TZP/DZP	0.00496	53.7194
	PBE0-DH/Sappo-QZP/DZP	0.00113	55.6002
	LC-BLYP/Sappo-QZP/DZP	0.00255	58.4521
	**Best predicting for frequencies**		
	LC-BLYP/Sappo-DZP/DZP	0.00714	55.9408
	TPSSh/Sappo-DZP/DZP	0.00274	57.7441
	B3LYP/Sappo-DZP/DZP	0.00340	57.5732
	HF/LANL2DZ/6-31G(d)	0.00457	49.7400
***cis*-$\mathbf{[Pt(NH_3)_2Cl_2]}$**	**Best predicting for structural parameters**		
	DSD-BLYP/Sappo-TZP/DZP	11.08221	66.7177
	B2PLYP/Sappo-QZP/DZP	11.00250	70.5960
	PBE-QIDHSappo-DZP/DZP	11.39877	64.4803
	**Best predicting for frequencies**		
	TPSSh/DKH-TZVP/TZVP	10.32347	83.5926
	LC-BLYP/Sappo-QZP/DZP	10.96500	72.7093
	B97-3c/DKH-TZVP/TZVP	10.12164	85.3001
	HF/LANL2DZ/6-31G(d)	13.07642	63.0902
$\mathbf{[PtCl_4]^{2-}}$	**Best predicting for structural parameters**		
	PBE-QIDH/DKH-TZVPP/TZVP	0.00005	88.8938
	DSD-BLYP/DKH-TZVPP/TZVP	0.00008	88.3191
	PBE0-DH/DKH-TZVPP/TZVP	0.00006	92.5682
	**Best predicting for frequencies**		
	PBE-QIDH/def2-QZVP	0.00005	102.2066
	DSD-BLYP/def2-QZVP	0.00003	101.7483
	LC-BLYP/Sappo-DZP/DZP	0.00015	78.7956
	HF/LANL2DZ/6-31G(d)	0.00005	73.8892

References

(1) Gustafsson, G.; Scullman, R. Rotational analysis of the 23/2-X23/2 and 27/2-X25/2 sub-systems of PtD. *Molecular Physics* **1989**, *67*, 981–988.

(2) Okabayashi, T.; Kurahara, T.; Okabayashi, E. Y.; Tanimoto, M. Microwave spec-

troscopy of platinum monofluoride and platinum monochloride in the X 2 3/2 states. *Journal of Chemical Physics* **2012**, *136*.

(3) Ayala, R.; Marcos, E. S.; Díaz-Moreno, S.; Solé, V. A.; Oz-Páez, A. M. Geometry and Hydration Structure of Pt(II) Square Planar Complexes $[Pt(H_2O)_4]^{2+}$ and $[PtCl_4]^{2-}$ as Studied by X-ray Absorption Spectroscopies and Quantum-Mechanical Computations. *J. Phys. Chem. B* **2001**, *105*, 7588–7593.

(4) Brieger, L.; Henke, S.; Mohamed, A. S.; Jourdain, I.; Knorr, M.; Strohmann, C. Redetermination of the crystal structure of tetrammineplatinum(II) dichloride – A microporous hydrogen-bonded 3D network exhibiting a temperature-dependent order-disorder phase transition. *Inorganica Chimica Acta* **2019**, *495*, 119002.

(5) Ting, V. P.; Schmidtmann, M.; Wilson, C. C.; Weller, M. T. Cisplatin: polymorphism and structural insights into an important chemotherapeutic drug. *Angewandte Chemie* **2010**, *122*, 9598–9601.

(6) Bridgeman, A. J.; an Cavigliasso, G.; Harris, N.; Young, N. A. A matrix isolation and DFT study of the generation and characterization of monomeric vapour phase platinum chlorides. *Chemical Physics Letters* **2002**, *351*, 319–326.

(7) Hendra, P. J. The Raman Spectra of Complex Anions of Formula ${MX_4}^{n-}$, where M is Au^{III}, Pt^{II}, or Pd^{II}, and X is a Halogen Atom. *J. Chem. Soc. A* **1967**, *16*, 1298–1301.

(8) Malik, M.; Michalska, D. Assessment of new DFT methods for predicting vibrational spectra and structure of cisplatin: Which density functional should we choose for studying platinum(II) complexes? *Spectrochimica Acta - Part A: Molecular and Biomolecular Spectroscopy* **2014**, *125*, 431–439.

(9) Bci Solver Github Page. Accessed: 2024-03-04.

(10) Anastasi, A. E.; Deeth, R. J. Capturing the trans influence in low-spin d8 square-planar platinum(II) systems using molecular mechanics. *Journal of Chemical Theory and Computation* **2009**, *5*, 2339–2352.

(11) Halgren, T. A. Merck molecular force field. 1. Basis, form, scope, parameterization, and performance of MMFF94. *J. Comput. Chem.* **1996**, *17*, 490–519.

(12) de Berrêdo, R. C.; Jorge, F. E.; Jorge, S. S.; Centoducatte, R. An augmented Gaussian basis set for calculations of molecular polarizabilities on platinum compounds. *Computational and Theoretical Chemistry* **2011**, *965*, 236–239.

(13) Cheng, X.; Ye, Y. L.; Zhang, L.; Zheng, K. W.; Li, X. H.; Sun, W. M. A theoretical study of the mono-substituent effect of superhalogens on the geometric structure, electronic properties, and hydrolysis of cisplatin. *Chemical Physics* **2022**, *555*.

(14) Alexander, C.; Nithyakumar, A.; Paul, M. W. B.; Samy, N. A. Platinum(II) complexes of imidazophenanthroline-based polypyridine ligands as potential anticancer agents: synthesis, characterization, in vitro cytotoxicity studies and a comparative ab initio, and DFT studies with cisplatin, carboplatin, and oxaliplatin. *Journal of Biological Inorganic Chemistry* **2018**, *23*, 833–848.

(15) Deng, D.; Lian, Y.; Zou, W. Permanent electric dipole moments of PtX (X = H, F, Cl, Br, and I) by the composite approach. *Chemical Physics Letters* **2017**, *688*, 33–36.
